# From Toxoplasmosis to Schizophrenia *via* NMDA Dysfunction: Peptide Overlap between *Toxoplasma gondii* and *N*-Methyl-d-Aspartate Receptors As a Potential Mechanistic Link

**DOI:** 10.3389/fpsyt.2017.00037

**Published:** 2017-03-15

**Authors:** Guglielmo Lucchese

**Affiliations:** ^1^Brain Language Laboratory, Freie Universtät Berlin, Berlin, Germany

**Keywords:** *Toxoplasma gondii*, *N*-methyl-d-aspartate receptors, NMDA 2D, peptide commonality, immune cross-reactivity, schizophrenia, parvalbumin-positive interneurons, gamma oscillations

## Abstract

The present work aims at investigating how *Toxoplasma gondii* (*T. gondii*) infection may be linked to *N*-methyl-d-aspartate receptor (NMDAR) dysfunction in schizophrenia and related disorders and puts forward the hypothesis that immune responses against *T. gondii* may involve NMDARs. Indeed, the analysis of the protozoan proteome and NMDAR subunits for peptide commonalities shows a massive peptide overlap and supports the possibility that anti-*T. gondii* immune responses raised during active protozoan infection may cross-react with host NMDARs, determining disruption of neural circuits and cognitive deficits. In particular, the NMDA 2D subunit, which is mainly expressed in parvalbumin-positive interneurons, appears to be a hotspot for potential *T. gondii*-induced cross-reactive immune attacks.

## Introduction

Schizophrenia is a multifaceted syndrome characterized by distinctive behavioral symptoms, cognitive deficits, and a complex etiopathogenesis, which seems to involve neurodevelopmental anomalies and a combination of genetic and environmental factors (1, 2). Among the environmental factors, *Toxoplasma gondii* (*T. gondii*) is gaining increasing attention, and a causal association between the protozoan infection and schizophrenia has been repeatedly suggested (3–9). Over the last decades, studies on *T. gondii* antibodies (Abs) in patients with schizophrenia revealed higher levels of anti-*T. gondii* Abs in the affected persons when compared to controls (8, 10–12). Interestingly, higher anti-*T. gondii* Ab levels were also found in mothers of offspring who later developed schizophrenia (13) and in newborns who later were diagnosed with the disease, as compared to controls (6, 14). This suggests that toxoplasmosis in early life might affect neurodevelopment and contribute to later onset of schizophrenia. However, the molecular determinants and mechanisms by which *T. gondii* infection might contribute to the pathophysiology of the disease remain unclear.

One major pathophysiological mechanism underlying development of schizophrenia seems to be *N*-methyl-d-aspartate glutamate receptor (NMDAR) dysfunction (15–17). The NMDA model of schizophrenia originated from the observation that NMDA antagonists, like ketamine or phencyclidine (PCP), transiently induce symptoms that mimic psychotic episodes (18–21). Following these initial observations, a large body of genetic and molecular evidence has accumulated in the last three decades indicating NMDA dysfunction as a convergence point in the development of schizophrenia (22–27). NMDA dysfunction not only can provide a satisfactory explanation of behavioral and cognitive symptoms of schizophrenia but is also consistent with the neurodevelopmental aspect of the disease, given that early NMDA aberrations/damage can translate into clinical onset later in life (22, 28, 29).

In summary, the NMDA model of schizophrenia seems then to be the common pathway of different etiological factors and is characterized by an early-damage late-onset temporal pattern, which is consistent with findings on increased risk of schizophrenia after early-life *T. gondii* infection. It is therefore reasonable to hypothesize that *T. gondii* can affect NMDAR function and glutamatergic neuronal circuits.

On this basis, the present work examines the hypothesis that immune responses to *T. gondii* may relate to NMDAR dysfunction by way of cross-reactive mechanisms and anti-NMDAR Abs. The rationale is that when a pathogen has sequence/structure similarity with human proteins, then anti-pathogen immune responses may cross-react with human proteins that share sequences/structures with the pathogen, thus triggering autoimmunity (30, 31). Such a hypothesis originates form the observations that (1) anti-*T. gondii* Ab levels are, as discussed above, higher in schizophrenic patients (8, 10–12), thus suggesting that immune responses following *T. gondii* active infection might play a role in the association of the parasite with the disease and (2) NMDAR blocking Abs are present in subjects with schizophrenia, schizoaffective, bipolar, and major depressive disorders (32–37), thus suggesting a role of anti-NMDAR immunoreactivity in the genesis of NMDA dysfunction in schizophrenia and other neuropsychiatric disorders. Moreover, a direct effect of early toxoplasmosis on behavioral anomalies and elevation of anti-NMDAR autoantibodies was found in a recent study on mice (38).

In light of this immunologic context, *T. gondii* proteome and the seven NMDAR subunit proteins (NMDA 1, 2A, 2B, 2C, 2D, 3A, and 3B) were searched for common peptides that might underlie immune cross-reactions between the protozoan and the human host. Data are reported showing that the *T. gondii* proteome and NMDAR subunits share a vast epitopic peptide platform that is centered on the 2D subunit and appears to be potentially significant to schizophrenia pathogenesis.

## Methods

The seven human NMDAR subunit aminoacidic sequence analyzed in this study were retrieved from the UniProt database^1^ (39), and are listed with their alternative names in parentheses, followed by amino acids (aa) length: NMDA 1 (GluN1, NMDZ1), 938; NMDA 2A (GluN2A, NMDE1), 1,464; NMDA 2B (GluN2B, NMDE2), 1,484; NMDA 2C (GluN2C, NMDE3), 1,233; NMDA 2D (GluN2D, NMDE4), 1,336; NMDA 3A (GluN3A, NMD3A), 1,115; and NMDA 3B (GluN3B, NMD3B), 1,043.

The protein sequence of each NMDA subunit was dissected into sequential hexapeptides that overlapped each other by five aa (for example, MSTMRL, STMRLL, TMRLLT, MRLLTL, and so forth). This procedure produced a library consisting of 8,578 NMDAR subunit hexapeptides. Each NMDAR hexapeptide was used as a probe to search the entire *T. gondii* proteome for occurrences of the same hexapeptide using the Pir Peptide Match program^2^ (40).

*Toxoplasma gondii* (strain VEG, NCBI Tax ID: 432359) was investigated. The *T. gondii* proteome consists of 8,404 proteins (Uniprot proteome: UP000002226). The protozoan *Entamoeba histolytica* (NCBI Tax ID: 5759; 7,959 proteins; Uniprot proteome: UP000001926) was used as a control.

The Immune Epitope DataBase^3^ (IEDB) (41) was searched for epitopes containing (or corresponding to) NMDAR hexapeptide(s) shared with *T. gondii* and experimentally validated as immunopositive in humans. Details, references, and immunoassay type for each epitope reported in the present study are available at http://www.iedb.org/advancedQueryEpitope.php.

## Results

Sequence-matching analyses were carried out at the 6-mer level since a grouping of 5–6 aa represents the minimal immune unit able to induce specific Abs and to determine specific antigen–antibody recognition (42–44).

The hexapeptide sharing between NMDAR subunits and *T. gondii* proteins and its immunological potential is quantified in Table 1 and detailed in Table S1 in the Supplementary Material. Table 1 shows that the seven NMDAR subunit proteins share a high number of hexapeptides with the protozoan proteome. On the whole, 2,215 out of the 8,578 hexapeptides composing the NMDAR library repeatedly occur in the protozoan proteome, for a total of 5,802 multiple occurrences. Theoretically, such an impressive level of peptide sharing equates to a vast source of potential cross-reactions in case of active toxoplasmosis and, indeed, NMDAR hexapeptides shared with the *T. gondii* proteome are also present in immunopositive epitopes (Table 1, last column).

**Table 1:** ***N*-methyl-d-aspartate receptor (NMDAR) hexapeptide sharing with *Toxoplasma gondii* proteome**.

NMDAR subunit	Total number of hexapeptides	Hexapeptides shared with *T. gondii* proteome	Hexapeptides shared with *T. gondii* proteome (including multiple occurrences)	Hexapeptides shared with *T. gondii* proteome and present in immunopositive epitopes
1	933	180	504	10
2A	1,459	254	488	16
2B	1,479	258	400	7
2C	1,228	374	947	22
2D	1,331	496	1,463	58
3A	1,110	262	977	8
3B	1,038	391	1,023	44

**Total:**	8,578	2,215	5,802	165

In order to define the immunologic potential of the hexapeptide commonality between NMDARs and *T. gondii*, the shared 2,215 hexapeptides were analyzed using the IEDB, an immune epitope catalog resource, in search of epitopes experimentally validated as immunopositive in the human host, and containing (or corresponding to) hexapeptides shared between *T. gondii* and human NMDAR proteins. One hundred sixty out of the 2,215 hexapeptides shared between the 7 human NMDARs and *T. gondii* were found to be disseminated through hundreds of IEDB epitopes that have been validated as immunopositive in humans. The 160 epitopic NMDAR hexapeptides and the IEDB epitopes are described in Tables 2 and 3, respectively.

**Table 2:** **Epitopic hexapeptides shared between the seven human *N*-methyl-d-aspartate receptor (NMDAR) subunit proteins and *T. gondii* proteome and present in epitopes experimentally validated as immunopositive in humans**.^a,b^

NMDA 1	NMDA 2A	NMDA 2B	NMDA 2C	NMDA 2D	NMDA 3A	NMDA 3B
AGGIVA	AAAEKG	AAPVAV	AGVSSS	AAAATA	APRAAS	AALARA
EEEEED	**ALSLIT**	**ALSLIT**	ASPPRQ	AAPPPA	**EELSGI**	AAPAEA
ELEARV	APSAAA	LAVLAV	FLDLPL	AATAVG	LLEKIA	AEAEAA
ELLEKE	EELETL	**LKTGKL**	GPALLL	AGGAGG	NFSLLL	ALLPRA
GRGALQ	ISLKDR	LRLLRT	GPGGPR	AGGGGS	PFSSPS	ALLSSL
LVAGGI	KGPPAL	QEAIAQ	LGPALL	APGPAP	PPGSRK	APRLPH
RGALQN	LEARVR	QKEEAA	LLLTSL	APPAAA	SELEKQ	APVPAA
SPGSPR	**LKTGKL**		LLTSLF	APPPPP	VPSSSS	ARAAPA
SVARAA	LPALLV		LSLRQK	APRGAA		ARARAR
TLASSF	LRSTAS		**LTVATL**	APRPAP		ARPPPP
	RELDLS		PALLLT	AVAAAV		ATLDAL
	SLEARV		PGGPRA	AVGPPL		EAPVPA
	SRDSRG		PPPSPC	AVVARG		**EELSGI**
	SRPSRS		QASPDL	GAAGRP		EGSKEE
	SRSISL		RSVEDA	GAALVL		ELVVAA
	SSVILL		SASERP	GAGEAV		EQQQQQ
			SFSPGG	GAGGAG		ERLRQA
			SLASPP	GAGGGA		GEAPVP
			SSVAEA	GAGGPG		GGLVAL
			TAGVSS	GAPAAP		GLALAL
			TDAPPA	GASLGG		GLLALG
			**TVATLE**	GGAGGG		GRPPAA
				GGAGGP		GSALLS
				GGGGSG		GVAALL
				GGGLGG		GVLARL
				GGPGGG		LARAAL
				GKKIDG		LEHPFV
				GLGLGL		LERRIE
				KPPPPP		LGSALL
				LAGGGG		LLAQLG
				LELLPP		LLQARA
				LLLLAL		LQARAA
				LLSGLR		PEADAE
				LPPPAP		PGVAAL
				**LTVATL**		PPGVAA
				PAAAAP		PPPPQG
				PAAAAT		PRAPLA
				PAEPPA		PTGAPQ
				PAPPAA		QELERR
				PGPGGA		QQQQQQ
				PLPSPA		RARAAL
				PPAAAA		RARARA
				PPPPPQ		RLRQAL
				PRGAAG		VLSLLR
				PSPPAP		
				PVALVL		
				RAAPPP		
				RAPAVA		
				RGAGGP		
				RGAQAL		
				RGPRGP		
				SGPAYA		
				SLELLP		
				SPPAPA		
				TPAAAA		
				**TVATLE**		
				VRPVAL		
				VSAQIR		

*^a^Hexapeptides common to the seven isoforms of NMDA 1 are given underlined*.

*^b^The five hexapeptides present in more than one subunit are in bold*.

**Table 3:** **Immunopositive epitopes containing hexapeptides shared between the seven human *N*-methyl-d-aspartate receptor (NMDAR) subunit proteins and *T. gondii* proteome**.

IEDB ID^a^	Epitope^b^	IEDB ID^a^	Epitope^b^	IEDB ID^a^	Epitope^b^	IEDB ID^a^	Epitope^b^	IEDB ID^a^	Epitope^b^
364	aapLPPPAPd	178516	ALLPRAgaaaaaalp	243209	rvLRLLRTlrplrvi	446380	lprALLSSL	460038	tpAVGPPL
1432	aGAGGGAGGAGag	179362	QASPDLlrgllstfi	255107	apakaaAPPAAArsa	446610	miRAAPPPl	460172	vagLAVLAV
11210	eavesTVATLEd	179712	ylglevltRARAALt	260421	avGVAALLplptvva	446684	mPPPPPQGv	460179	vAPPPPPvev
16552	FLDLPL	180820	wlvhrqwFLDLPLpw	265472	ddddddepeEGSKEE	447353	rgsLARAAL	460604	VRPVALVL
16553	FLDLPLpwl	189280	slyLTVATL	275388	edEEEEEDEEEEEDe	447526	rpaLPALLV	460633	vtlasGGLVAL
16554	FLDLPLpwt	193670	aLLSGLRea	348447	pakaaAPPAAArsae	448486	sPGGPRAav	460697	wlknGAALVL
19674	gGAGGAGGaGAGGGA	193930	iiNFSLLLv	348613	papakaaAPPAAArs	448497	SPGSPRpal	463472	APPPPPppv
34978	laTAGVSSSdslvsp	194050	kliEELETL	359598	qekkeEEEEEDgieq	451566	aaaPAPPAA	463529	APRPAPvaqppaaa
46489	nvsVPSSSStplly	194088	klPPPPPQa	375698	sapakaaAPPAAArs	451598	aaPAAAAPa	463617	APVPAAaav
48720	pnvsVPSSSStplly	196338	APPPPPppp	410921	vsapakaaAPPAAAr	451603	aaPAPPAAA	465860	glsgSGPAYA
51177	QKEEAAicgqmdls	196592	KPPPPPppp	418132	fqinqdEEEEEDed	451607	aAPGPAPl	466021	gpPSPPAPvm
67823	vaytlaTAGVSSSds	198666	AEAEAAsvrm	418885	sprrSRSISL	451608	aAPPAAAAa	466282	hlwtgevsAAPPPA
68358	vesTVATLEd	199105	aeprPAEPPAw	419522	apqAPPPPPk	451658	AEAEAAvgl	466749	ipLLLTSL
69908	vlyspnvsVPSSSS	202838	atASPPRQk	420032	pvRAPAVAv	452024	aEQQQQQmy	466793	iprgpPSPPAPv
73319	yAALARAAL	210095	gLLEKIATpk	420626	kLVAGGInav	452378	ALLSSLarc	466794	iprgpPSPPAPvm
77946	LGPALLLll	217152	qlkLKTGKL	423463	prppplgrgRGAGGP	452567	aPAAAAPaa	469288	ppAPPPPPv
94735	VPSSSStpl	217651	RELDLSgqgf	423802	AAAATAdvtly	452663	apgKGPPAL	470036	rpikGAAGRPlel
103165	feetfevtaAAPVAV	219411	spaSRSISL	424158	avSSVAEAy	452666	APGPAPsql	470660	slmaelGEAPVPAsv
103645	tgGGGGSGfsnsgsg	222405	GEAPVPAsv	424351	ffGAGGAGy	452751	APPPPPkal	474124	yTDAPPAysel
113351	eaGAGGGA	224924	pAPPPPPpp	424829	GASLGGiiy	452752	APPPPPtsm	474710	AEAEAAavhga
114666	vrSRPSRSrssrser	227016	aGAGGGAGAGGAGGa	424842	gffGAGGAGy	452779	aprelGLGLGL	474785	aeAPPPPPp
115985	sTVATLEdsp	227017	AGAGGGAGAGGGAgg	426558	nlLQARAAlqtay	452781	APRGAAGl	475138	aelGEAPVP
118616	svsyddwdySLEARV	227018	AGAGGGAGGAGaggg	427326	SLELLPPp	452792	APRPAPvaq	475252	aeqepELEARVa
121117	adGKKIDG	227019	aGAGGGAGGAGGaga	433554	yTDAPPAysely	452810	APSAAAlpa	476614	avrPPAAAAak
121776	RARARARa	227020	AGAGGGAGGAGGagg	435140	grlPLPSPAl	453128	dqvgGVLARL	477189	eaPAAAATA
121811	rRARARAR	227021	aggaGAGGAGaggag	435175	grSSVILLty	454126	gLAVLAVvv	477192	eAPPPPPpp
122052	ykhadGKKIDGrrvl	227023	aggaGAGGGAgagga	435395	krQEAIAQnr	454159	GLLALGdymnv	477741	eLGSALLSl
127989	ggaGAGGGAgagg	227024	aggaGAGGGAGGAGG	437234	fELLEKEvgl	454273	GPGGPRnl	479011	gveGAPAAP
132330	GGGLGGtrrg	227025	aggGAGGAGAGGGAg	437708	gPPPPPQGgrpp	454675	ikGAAGRPlel	482100	lqkLKTGKL
132548	prrlGGGLGG	227188	gaGAGGGAGGAGagg	437719	gptslgGGAGGPll	455937	lAAPPPApa	482881	paaAPPAAA
132613	rlGGGLGGtr	227189	GAGGAGAGGGAGGAG	438549	kEQQQQQmw	455970	lAPPPPPaa	483053	qeAPPPPPp
138044	SRDSRGkpgy	227204	ggGAGGAGAGGGAgg	438691	klqELEARV	456697	lsrlPALLLTg	486281	spaSRSISLl
142502	aAGGGGStdnlsy	235218	lGVAALLfgfpiffd	439841	refPEADAEkl	457321	paAAAATAl	487382	tevAPPPPP
144908	iepRGAQAL	236707	aeLRSTASll	440445	SELEKQdnsw	457322	PAAAAPaaa	492366	iRAAPPPlfll
146892	epeAEAEAAagpgp	239263	AATAVGggflll	441412	vLLAQLGpqpg	457324	paaPAAAAPgy	492772	krGLALALf
150802	iRAAPPPlf	239392	alLAGGGGppak	442395	amPPPPPQGv	457338	papARAAPA	493404	mrpGPALLLlgv
156427	qqgrQQQQQQQgqqg	239445	aprelglGLGLGL	442558	appERLRQAL	457425	PPGVAALsi	495305	srTPAAAAam
161826	aegGRPPAA	239460	aprpaAAAATAl	442568	APRAAStesl	457922	rARPPPPstl	504497	APGPAPtrc
162831	LEHPFVssi	239463	APRPAPvaqppa	442615	APRLPHsvtc	458285	rpaGPALLL	504498	APGPAPtrcl
169260	maiakaAAAEKGvpl	239473	aprtPGPGGArl	442702	apsSPGSPRpal	458546	rpyLGPALL	505654	gpgifPPPPPQp
176712	VSAQIRknf	239607	eestgLLSGLRiw	443010	AVVARGttilak	458547	rpyLGPALLL	505667	gPPPPQGkp
176870	AEAEAAavhgarf	240452	SPPAPAgsratl	444271	glaAGGIVAv	458577	RSVEDAqaw	507174	miRAAPPPlf
176959	agytpatpAAPAEAa	240535	stAPPPPPllle	444325	gLVAGGIiga	459541	sprpaLPALLV	507502	pgaRGPRGPp
177000	eaqkaakPAAAATAt	240571	tgggGGGGSGgtrm	444514	grlPLPSPAley	459542	sprPFSSPSm	507608	qgPPPPQGkpq
177124	kaakPAAAATAtata	240573	tkeAVAAAVaav	445222	iRAAPPPlfl	459704	sTLASSFk	509894	LPPPAPaev
177139	kPAAAATAtataavg	242669	ilgmlrvLRLLRTlr	446022	ktlLLTSLF	459875	tgAPGPAPp	510025	ndAPRAASi

*^a^Immunopositive epitopes containing hexapeptides shared between T. gondii and NMDAR subunits are listed according to IEDB ID number. Only epitopes ≤15 aa are reported. Details and references are available at www.immuneepitope.org/*.

*^b^Peptide sequences shared between NMDAR subunit proteins and T. gondii proteome are given in capital letters*.

Control analyses using the protozoan *E. histolytica*, a human pathogen associated with intestinal and extraintestinal infections (45), highlight a lower extent of peptide sharing (Table 4), thus indicating that the intensity of the hexapeptide sharing between *T. gondii* and the NMDAR subunits—in particular, NMDA 2D and 3B—is specific (Tables 1 and 2). The detailed description of the peptide sharing between the NMDAR subunits and the protozoan *E. histolytica* is reported in Tables S2 and S3 in Supplementary Material.

**Table 4:** ***N*-methyl-d-aspartate receptor (NMDAR) hexapeptide sharing with *Entamoeba histolytica* proteome**.

NMDAR subunit	Total number of hexapeptides	Hexapeptides shared with *E. histolytica* proteome	Hexapeptides shared with *E. histolytica* proteome (including multiple occurrences)	Hexapeptides shared with *E. histolytica* proteome and present in immunopositive epitopes
1	933	100	240	13
2A	1,459	134	227	10
2B	1,479	160	241	9
2C	1,228	92	141	5
2D	1,331	109	192	15
3A	1,110	101	211	3
3B	1,038	86	142	10

**Total:**	8,578	782	1,394	65

## Discussion

### The Spatiotemporal NMDAR Subunit Expression May Shape the Potential Cross-reactions between *T. gondii* and NMDARs

Tables 2 and 3 indicate that *T. gondii* active infection might induce immune reactions able to cross-react to different extents with the seven NMDAR subunits. In particular, analysis of Table 2 shows that epitopic hexapeptides shared with *T. gondii* are mostly allocated in the NMDA 2D, 3B, and 2C subunits (58, 44, and 22 epitopic hexapeptides, respectively), whereas a relatively lower level of hexapeptide sharing characterizes NMDA 2A, 1, 3B, and 3A (16, 10, 8, and 7 epitopic hexapeptides, respectively).

This appears to be relevant in light of the fact that NMDARs consist of four subunits, two of which invariably are NMDA 1 subunits that can associate with NMDA 2 subunits or a combination of NMDA 2 and NMDA 3 subunits. Such a variable composition results in a large number of NMDAR subtypes, which present different spatiotemporal patterns of expression during neurodevelopment and in the young/adult life (46). For instance, NMDA 2B and NMDA 2D are expressed during embryonic development, whereas NMDA 2A and NMDA 2C gene expression starts after birth; NMDA 2A and NMDA 2B are highly expressed in the cortex and hippocampus and NMDA 2C in the cerebellum of adult rodents (47–52). Likewise, NMDA 3A and NMDA 3B genes have specific developmental patterns of expression (53).

Even the NMDA 1 subunit, which is a constitutive component of all NMDARs, presents a heterogenous distribution and a varying immunoreactivity potential when its seven isoforms are analyzed (54). For example, only immune cross-reactions against 6 out of the 10 epitopic hexapeptides present in all NMDA 1 isoforms (Table 2)—namely, AGGIVA, EEEEED, ELEARV, ELLEKE, SPGSPR, and SVARAA—should be able to produce a diffuse immunoreactivity in the brain.

Hence, *T. gondii*-induced immune cross-reactions might have different outcomes depending on the targeted NMDAR subunit and might target different subunits depending on the timing of exposure to the protozoan. Indeed, the timing of exposure to *T. gondii* is a crucial factor in generating different specific anti-NMDAR Abs and, consequently, different associated neurobehavioral disorders (38).

### NMDA 2D Is the Main Potential Target of Anti-*T. gondii* Immune Response

NMDA 2D exemplifies the potential relationship between toxoplasmosis-induced immunoreactivity, the spatiotemporal expression profile of NMDAR subunits, and different potential outcomes. Indeed, NMDA 2D contains 496 hexapeptides in common with the protozoan (Table 1), 58 of which are present in validated immunopositive epitopes (Table 1). Therefore, NMDA 2D might be the main target of the immune cross-reactivity potentially associated with *T. gondii* infection. Studies in animals [(48–52) and more references therein] showed that NMDA 2D gene expression:

– is high in the midbrain, the diencephalon, and the spinal cord before birth;
– is abundant around birth in thalamic and hypothalamic nuclei and in the brainstem;
– reaches a peak around 1 week after birth; and
– subsequently declines and persists mainly in the hippocampal interneurons, most of which are somatostatin (SOM)- and parvalbumin (PV)-positive cells.

Translating data from animal models to the human brain, it is logical to presume that immune cross-reactions involving NMDA 2D in the fetus and the newborn can extensively occur throughout the brain, whereas secondary immune responses after early sensitivization might target hippocampal PV- and SOM-positive interneurons in the young/adult. Indeed, alterations in the hippocampal PV- and SOM-positive interneurons have been repeatedly related to the hippocampal hyperactivity that characterizes schizophrenia (55–57).

### NMDARs vs *T. gondii* Epitopic Peptide Overlap and the NMDA Model of Schizophrenia

The vast epitopic peptide sharing between *T. gondii* and the seven NMDAR subunits (see Tables 2 and 3) suggests that anti-*T. gondii* immune responses cross-reacting with NMDARs might lead to NMDAR damage and dysfunction, targeting in particular interneurons expressing NMDA 2D. Crucially, this hypothesis, which links mechanistically toxoplasmosis and schizophrenia by way of peptide sharing with NMDARs, is consistent with the well-established NMDA dys/hypofunction model of schizophrenia (15–17). As mentioned in the Introduction, the NMDA model is based on the observation that NMDA antagonists, like PCP, Ketamine, and MK801, induce symptoms that resemble schizophrenia (2, 17), and it seems to be able to provide a good account of some aspects of the complex symptomatology of the disease, including cognitive symptoms (17). Remarkably, both of these two fundamental aspects of the NMDA model are related to the NMDA 2D subunit type. First, targeting NMDA 2D appears to be a major mechanism in the pharmacodynamics of MK801 (58), ketamine (59, 60), and PCP (61, 62). Second, 2D-containing NMDARs are typically expressed in GABAergic interneurons, where they largely contribute to excitatory post-synaptic potentials (49, 51, 63). Consequently, NMDAR dysfunction in these cells would translate into reduced GABAergic activity and consequent reduced inhibitory control of pyramidal cell activity (2, 64). The excitatory–inhibitory balance in cortical networks is crucial for generating high-frequency (gamma) oscillatory activity (65, 66), and it appears that the disruption of gamma-band oscillations (GBOs) might indeed underlie cognitive deficits in schizophrenia (67–70). On the one hand, GBOs are well known to be physiologically related to higher cognitive functions (71–74), on the other hand, they are typically altered in schizophrenic patients (75–77). Moreover, ketamine alters gamma oscillatory activity by targeting NMDA 2D (59). It appears then that NMDA 2D damage can be directly related to cognitive deficits in schizophrenia.

In summary, in the complex and articulated picture that connects PV interneurons, brain oscillation, and cognition [Ref. (78, 79) for review], a large body of evidence from pharmacological, genetic, electrophysiological, and clinical research converges on a critical role of NMDA 2D in cognition within the context of the NMDA model of schizophrenia. It is likely then that *T. gondii*-induced anti-NMDA 2D cross-reactivity might, among other different mechanisms triggered by both genetic and environmental factors, play a role in contributing to NMDA dysfunction and GABA hypofunction, thus resulting in cortical circuitry disequilibrium and, potentially, in disruption of brain oscillation and cognitive processes.

## Conclusion

This work presents and examines the hypothesis that the relationship between *T. gondii* and schizophrenia might be explained by way of shared peptides (as molecular determinants) and immune cross-reactivity (as biological mechanism) between *T. gondii* proteins and the NMDAR subunits. The high and specific peptide commonality with the NMDARs shown by *T. gondii*, as compared to the control, supports the possibility that the infection might induce anti-NMDAR immune responses in the human host through cross-reactivity (Table 1) and more so in light of the epitopic nature of many of the shared peptides (Tables 2 and 3). Such a hypothesis is consistent, on the one hand, with previous studies describing the potential neuropsychiatric relevance of the vast peptide commonality existing between infectious agents and the human host (80–82) and, on the other hand, with the well-established NMDA dysfunction model of schizophrenia. Hence, a possible scenario unfolds, where the differential spatiotemporal patterns of expression of the NMDAR subunits might generate the diversity of neuropathological outcomes. In this regard, immune attacks on NMDA 2D, a main potential target of *T. gondii*-induced cross-reactions, may represent a mechanistic link between *T. gondii* infection and NMDAR dysfunction in neuropsychiatric disorders.

In summary, immune cross-reactions with NMDARs following *T. gondii* infection might be one of the factors contributing to the pathophysiology of schizophrenia and associated disorders, and NMDAR subunit composition could relate to the timing and the targets of the neuropathologic sequela of the exposure to *T. gondii*. The hypothesis presented here might help to address aspects of the complex and multifactorial etiopathogenesis of schizophrenia in future clinical and basic research.

## Author Contributions

GL designed the study, performed the analyses, and wrote the manuscript.

## Conflict of Interest Statement

The author declares that the research was conducted in the absence of any commercial or financial relationships that could be construed as a potential conflict of interest.

## Footnotes

^1^www.uniprot.org.

^2^http://research.bioinformatics.udel.edu/peptidematch/index.jsp.

^3^www.immuneepitope.org.
