# Supplementary material for: From Toxoplasmosis to Schizophrenia via NMDA Dysfunction: Peptide Overlap between Toxoplasma gondii and N-Methyl-d-Aspartate Receptors As a Potential Mechanistic Link
Source: Front Psychiatry. 2017 Mar 15;8:37. doi: 10.3389/fpsyt.2017.00037 (PMC5350139; doi:10.3389/fpsyt.2017.00037)
Supplement: Supplementary file 1 [file Table_1.DOC]

**Supplementary Table S1. Distribution of NMDAR hexapeptides (5802 including multiple occurrences) throughout the *T. gondii*  proteome**

N° NMDAR *T. gondii* *T. gondii* *T. gondii* Protein Name Protein Matched Range(s)

Peptide Protein AC Protein ID length (aa)

**NMDA 1**

1. STMRLL B9Q6I9 B9Q6I9_TOXGO Uncharacterized protein 5651 2352-2357
2. STMRLL B9Q6I9 B9Q6I9_TOXGO Uncharacterized protein 5651 2352-2357
3. TMRLLT Q1JTI3 Q1JTI3_TOXGO Ubiquitin-protein ligase 1, putative 8112 2785-2790
4. TMRLLT V4Z553 V4Z553_TOXGO HECT-domain (Ubiquitin-transferase) domain-containing protein 8007 2785-2790
5. RLLTLA V4YIP2 V4YIP2_TOXGO Uncharacterized protein 1604 853-858
6. RLLTLA V4Z3C9 V4Z3C9_TOXGO Uncharacterized protein 1899 1056-1061
7. RLLTLA V4ZT82 V4ZT82_TOXGO Uncharacterized protein 3407 59-64
8. LLTLAL B6KU18 B6KU18_TOXGO 3-ketoacyl-(Acyl-carrier-protein) reductase 376 21-26
9. LLTLAL B9Q7J7 B9Q7J7_TOXGO DNA-directed RNA polymerase III POLR3C 823 514-519
10. LLTLAL Q1JT96 Q1JT96_TOXGO Putative uncharacterized protein precursor 1412 26-31
11. LLTLAL V4YLT3 V4YLT3_TOXGO Putative transmembrane protein 1521 26-31
12. LLTLAL V4Z6H7 V4Z6H7_TOXGO UvrD/REP helicase domain-containing protein 3190 1332-1337
13. LLTLAL V4Z8G3 V4Z8G3_TOXGO RNA-dependent RNA polymerase RDP 2894 627-632
14. LLTLAL V4ZT82 V4ZT82_TOXGO Uncharacterized protein 3407 60-65
15. LTLALL B9PUE2 B9PUE2_TOXGO Uncharacterized protein 224 169-174
16. LTLALL Q1JT96 Q1JT96_TOXGO Putative uncharacterized protein precursor 1412 27-32
17. LTLALL V4YLT3 V4YLT3_TOXGO Putative transmembrane protein 1521 27-32
18. LTLALL V4YQZ6 V4YQZ6_TOXGO Putative ribosomal protein RPS14 318 14-19
19. LTLALL V4Z6H7 V4Z6H7_TOXGO UvrD/REP helicase domain-containing protein 3190 1333-1338
20. LTLALL V4ZDX1 V4ZDX1_TOXGO Uncharacterized protein 475 158-163
21. TLALLF Q1JST6 Q1JST6_TOXGO Uncharacterized protein 1030 648-653
22. TLALLF V4YQZ6 V4YQZ6_TOXGO Putative ribosomal protein RPS14 318 15-20
23. TLALLF V4Z1S4 V4Z1S4_TOXGO HEAT repeat-containing protein 1450 1059-1064
24. TLALLF V4ZDH8 V4ZDH8_TOXGO ALG6, ALG8 glycosyltransferase family protein 1585 1006-1011
25. TLALLF V4ZKX2 V4ZKX2_TOXGO Uncharacterized protein 1348 950-955
26. TLALLF V5B555 V5B555_TOXGO Putative AAA family domain ATPase 2965 1729-1734
27. LALLFS A3FKK1 A3FKK1_TOXGO PMCA-like calcium ATPase A1 1405 169-174
28. LALLFS A5Y4G3 A5Y4G3_TOXGO SET domain-containing protein 8 1893 557-562
29. LALLFS B6K8Q8 B6K8Q8_TOXGO Plasma membrane-type Ca(2+)-ATPase A1 PMCAA1 1822 586-591
30. LALLFS B9QI05 B9QI05_TOXGO Histone lysine methyltransferase SET8 1906 570-575
31. LALLFS Q9N694 Q9N694_TOXGO Ca2+-ATPase 1405 169-174
32. LALLFS V4Z6B3 V4Z6B3_TOXGO Putative transmembrane protein 462 14-19
33. LALLFS V4ZFI3 V4ZFI3_TOXGO Uncharacterized protein 3747 2272-2277
34. LALLFS V5B555 V5B555_TOXGO Putative AAA family domain ATPase 2965 1730-1735
35. LFSCSV B6KTA3 B6KTA3_TOXGO Formin 3 2849 1030-1035
36. LFSCSV V4ZEF9 V4ZEF9_TOXGO Formin FRM3 2847 1030-1035
37. SVARAA B9QCQ8 B9QCQ8_TOXGO AP2 domain transcription factor AP2XII-8 1679 246-251
38. SVARAA B9QIL7 B9QIL7_TOXGO Uncharacterized protein 1172 23-28
39. SVARAA Q1JSA7 Q1JSA7_TOXGO Uncharacterized protein 2639 635-640
40. SVARAA V4YMT2 V4YMT2_TOXGO Uncharacterized protein 2796 635-640
41. SVARAA V4YXQ9 V4YXQ9_TOXGO Uncharacterized protein 2949 1624-1629
42. SVARAA V4Z4M8 V4Z4M8_TOXGO Amine-terminal region of chorein, A TM vesicle-mediated sorter 13455 1165-1170
43. SVARAA V4ZSF1 V4ZSF1_TOXGO Transporter, major facilitator family protein 2200 1239-1244
44. AACDPK V4YY91 V4YY91_TOXGO Uncharacterized protein 2624 1587-1592
45. AVLSTR V4Z952 V4Z952_TOXGO Uncharacterized protein 4210 882-887
46. AVLSTR V4ZLN6 V4ZLN6_TOXGO Uncharacterized protein 654 473-478
47. TRKHEQ V4ZP14 V4ZP14_TOXGO RIC1 protein 3789 2844-2849
48. REAVNQ V4ZPM1 V4ZPM1_TOXGO Uncharacterized protein 2234 414-419
49. NQANKR V4ZF87 V4ZF87_TOXGO AP2 domain transcription factor AP2X-6 4495 3979-3984
50. QLNATS V4ZKM7 V4ZKM7_TOXGO Uncharacterized protein 1278 1170-1175
51. VSHPPT B9PSC0 B9PSC0_TOXGO AGC kinase 514 68-73
52. RIPVLG B9PHW6 B9PHW6_TOXGO Putative gamma-glutamyl phosphate reductase 502 264-269
53. SIHLSF V4Z034 V4Z034_TOXGO Uncharacterized protein 1634 779-784
54. LSFLRT V4ZAD4 V4ZAD4_TOXGO Isoleucyl-tRNA synthetase family protein 1242 105-110
55. PYSHQS B6KAM0 AMA1_TOXGO Apical membrane antigen 1 precursor 569 17-22
56. LLVSDD V4Z0T8 V4Z0T8_TOXGO Uncharacterized protein 481 87-92
57. LLVSDD V4Z875 V4Z875_TOXGO Actin-like family protein 528 474-479
58. LLVSDD V5BM36 V5BM36_TOXGO Poly(ADP-ribose) polymerase catalytic domain-containing protein 1012 323-328
59. EGRAAQ B6KP57 B6KP57_TOXGO Protein phosphatase 2C domain-containing protein 703 52-57
60. EGRAAQ Q1JSQ6 Q1JSQ6_TOXGO Protein phosphatase 2c, putative 709 52-57
61. GRAAQK V4YQ73 V4YQ73_TOXGO MIF4G domain-containing protein 3756 1721-1726
62. AAQKRL V4Z704 V4Z704_TOXGO Uncharacterized protein 538 402-407
63. AQKRLE B9PTW7 B9PTW7_TOXGO Ubiquinol-cytochrome c reductase 234 215-220
64. AQKRLE V4ZAS4 V4ZAS4_TOXGO Mediator complex subunit MED4 519 114-119
65. AQKRLE V4ZL97 V4ZL97_TOXGO Uncharacterized protein 7954 3107-3112
66. KRLETL B9Q1H5 B9Q1H5_TOXGO Uncharacterized protein 477 154-159
67. KRLETL V5BBA8 V5BBA8_TOXGO Hydrolase CocE/NonD family protein 1260 243-248
68. KRLETL V5BKT3 V5BKT3_TOXGO Uncharacterized protein 976 616-621
69. KRLETL V5BL43 V5BL43_TOXGO Uncharacterized protein 2687 675-680
70. RLETLL V5BL43 V5BL43_TOXGO Uncharacterized protein 2687 676-681
71. LETLLE V4ZF56 V4ZF56_TOXGO Uncharacterized protein 1083 612-617
72. LETLLE V5BDC0 V5BDC0_TOXGO Uncharacterized protein 1391 305-310
73. ETLLEE B9QGP5 B9QGP5_TOXGO Serine/threonine-protein phosphatase 2A activator 561 364-369
74. ETLLEE Q1JST6 Q1JST6_TOXGO Uncharacterized protein 1030 1015-1020
75. ETLLEE V4Z1S4 V4Z1S4_TOXGO HEAT repeat-containing protein 1450 1435-1440
76. ETLLEE V4ZCT3 V4ZCT3_TOXGO Tubulin/FtsZ family, GTPase domain-containing protein 300 23-28
77. TLLEER V4YYU7 V4YYU7_TOXGO Putative glyoxalase 4813 4243-4248
78. TLLEER V4ZCD0 V4ZCD0_TOXGO Putative trichohyalin 497 344-349
79. TLLEER V5BDD5 V5BDD5_TOXGO CPSF A subunit region protein 2077 78-83
80. LLEERE Q6JD66 Q6JD66_TOXGO Eukaryotic initiation factor-2 alpha kinase-A 5072 1808-1813
81. LLEERE V4Z5I1 V4Z5I1_TOXGO Uncharacterized protein 3696 3159-3164
82. LLEERE V4ZU83 V4ZU83_TOXGO eIF2 kinase IF2K-A (Incomplete catalytic triad) 4638 1374-1379
83. LEERES B6KBH5 B6KBH5_TOXGO Putative C2H2 type zinc-finger protein 397 45-50
84. LEERES B6KK36 B6KK36_TOXGO Triose-phosphate isomerase TPI-II 375 252-257
85. LEERES Q1KSE3 Q1KSE3_TOXGO Triosephosphate isomerase 374 252-257
86. LEERES V4Z7J7 V4Z7J7_TOXGO Uncharacterized protein 1162 732-737
87. LEERES V4ZDM0 V4ZDM0_TOXGO HEAT repeat-containing protein 4132 1812-1817
88. EERESK V4Z801 V4Z801_TOXGO Uncharacterized protein 2495 1447-1452
89. EERESK V4ZJ30 V4ZJ30_TOXGO Uncharacterized protein 1104 553-558
90. ERESKA B6KFL3 B6KFL3_TOXGO Putative transmembrane protein 193 77-82
91. ERESKA V4Z611 V4Z611_TOXGO Putative transmembrane protein 1126 569-574
92. ERESKA V4ZJ30 V4ZJ30_TOXGO Uncharacterized protein 1104 554-559
93. ERESKA V5AWP5 V5AWP5_TOXGO HEAT repeat-containing protein 1766 1541-1546
94. RESKAE V5B344 V5B344_TOXGO AP2 domain transcription factor AP2III-1 1959 226-231
95. ESKAEK A5YVK6 A5YVK6_TOXGO Regulator of chromosome condensation 1 1155 1087-1092
96. ESKAEK B9QGB2 B9QGB2_TOXGO Regulator of chromosome condensation RCC1 1156 1088-1093
97. ESKAEK V4YTZ2 V4YTZ2_TOXGO Putative long-chain fatty acid CoA ligase 758 248-253
98. AEKVLQ V4ZMD3 V4ZMD3_TOXGO Uncharacterized protein 912 8-13
99. NVTALL V4YQ17 V4YQ17_TOXGO Putative transmembrane protein 534 374-379
100. ALLMEA V4Z4U3 V4Z4U3_TOXGO ABC transporter, ATP-binding domain-containing protein 1885 1614-1619
101. EAKELE V4YTX2 V4YTX2_TOXGO Uncharacterized protein 2251 974-979
102. AKELEA V4Z0B0 V4Z0B0_TOXGO Uncharacterized protein 676 165-170
103. KELEAR B9QJ77 B9QJ77_TOXGO Uncharacterized protein 2002 675-680
104. KELEAR V4ZAT6 V4ZAT6_TOXGO DEAD/DEAH box helicase domain-containing protein 1773 1199-1204
105. ELEARV V4Z6Q7 V4Z6Q7_TOXGO AP2 domain transcription factor AP2VIII-2 2503 1745-1750
106. ELEARV V4ZAT6 V4ZAT6_TOXGO DEAD/DEAH box helicase domain-containing protein 1773 1200-1205
107. IILSAS Q45WA6 Q45WA6_TOXGO Rhoptry protein 14 1061 524-529
108. LSASED V4Z739 V4Z739_TOXGO Uncharacterized protein 1139 579-584
109. SASEDD V4Z129 V4Z129_TOXGO Sterol-sensing domain of SREBP cleavage-activation domain- protein 1535 48-53
110. ASEDDA V4ZVI6 V4ZVI6_TOXGO SufB/sufD domain-containing protein 1860 1255-1260
111. ASEDDA V4ZWR3 V4ZWR3_TOXGO Uncharacterized protein 97 55-60
112. SEDDAA V4ZI56 V4ZI56_TOXGO Uncharacterized protein 2083 446-451
113. SEDDAA V5AYL8 V5AYL8_TOXGO Uncharacterized protein 1236 1215-1220
114. SEDDAA V5BII2 V5BII2_TOXGO Uncharacterized protein 462 187-192
115. DDAATV B6K980 B6K980_TOXGO Uncharacterized protein 379 168-173
116. DDAATV V4Z0W0 V4Z0W0_TOXGO Putative elongation factor TS 806 619-624
117. DAATVY B6KTY3 B6KTY3_TOXGO REJ domain protein 740 553-558
118. VYRAAA B9QF05 B9QF05_TOXGO Uncharacterized protein 1594 467-472
119. RAAAML V4Z525 V4Z525_TOXGO Uncharacterized protein 250 193-198
120. AAAMLN V5BJY3 V5BJY3_TOXGO HEAT repeat-containing protein 2699 234-239
121. TGSGYV V4YW85 V4YW85_TOXGO EF hand domain-containing protein 716 607-612
122. LVGERE V5BN33 V5BN33_TOXGO WD domain, G-beta repeat-containing protein 948 236-241
123. VGEREI V4Z8T5 V4Z8T5_TOXGO Uncharacterized protein 3210 154-159
124. EREISG B9QPF5 B9QPF5_TOXGO BT1 family protein 994 891-896
125. ISGNAL Q45W09 Q45W09_TOXGO Putative ATP-binding cassette protein 1179 88-93
126. ISGNAL V4YUB6 V4YUB6_TOXGO Uncharacterized protein 1153 745-750
127. ISGNAL V4ZFH0 V4ZFH0_TOXGO ABC transporter, ATP-binding domain-containing protein 1445 356-361
128. LRYAPD Q962X0 Q962X0_TOXGO Peroxidoxin 2 224 216-221
129. LRYAPD V5BHL2 V5BHL2_TOXGO Putative peroxiredoxin 6 402 394-399
130. LGLQLI V4ZPP7 V4ZPP7_TOXGO Putative transmembrane protein 285 195-200
131. NGKNES B9PZT1 B9PZT1_TOXGO WWE domain-containing protein 756 495-500
132. NGKNES B9Q1Y2 B9Q1Y2_TOXGO Lysine decarboxylase family protein 361 100-105
133. GKNESA B9QEQ3 B9QEQ3_TOXGO G-protein beta WD-40 repeat containing protein 607 266-271
134. GKNESA V4YNU1 V4YNU1_TOXGO Putative transmembrane protein 3158 2927-2932
135. GKNESA V4ZHE1 V4ZHE1_TOXGO ATG C terminal domain-containing protein 8079 2497-2502
136. SAHISD Q1JTI3 Q1JTI3_TOXGO Ubiquitin-protein ligase 1, putative 8112 2628-2633
137. SAHISD V4Z553 V4Z553_TOXGO HECT-domain (Ubiquitin-transferase) domain-containing protein 8007 2628-2633
138. ISDAVG B9QPF5 B9QPF5_TOXGO BT1 family protein 994 360-365
139. AVGVVA V5B368 V5B368_TOXGO Transporter, major facilitator family protein 760 550-555
140. GVVAQA B6KA81 B6KA81_TOXGO Spc97 / Spc98 family protein 1427 378-383
141. GVVAQA V4ZHD9 V4ZHD9_TOXGO Putative transmembrane protein 1265 8-13
142. AQAVHE B9QEX3 B9QEX3_TOXGO Bromodomain-containing protein 1354 686-691
143. AVHELL V4ZHU0 V4ZHU0_TOXGO ATPase, AAA family protein 1031 625-630
144. VHELLE Q1JTI2 Q1JTI2_TOXGO Sulfite oxidase, putative precursor 434 107-112
145. VHELLE V4Z5A0 V4Z5A0_TOXGO Putative sulfite oxidase 845 453-458
146. ELLEKE B9Q083 B9Q083_TOXGO Uncharacterized protein 192 108-113
147. ELLEKE Q4FAB5 Q4FAB5_TOXGO ATP-binding cassette sub-family B member 4 718 685-690
148. ELLEKE V4Z852 V4Z852_TOXGO ABC transporter family protein 1127 1094-1099
149. ELLEKE V5B9Z5 V5B9Z5_TOXGO Uncharacterized protein 681 312-317
150. ELLEKE V5BLK1 V5BLK1_TOXGO Tumor suppressor mitostatin 245 96-101
151. PRGCVG Q1JTK5 Q1JTK5_TOXGO Putative uncharacterized protein precursor 406 188-193
152. MSSKYA B9PVW1 B9PVW1_TOXGO Uncharacterized protein 388 133-138
153. GVTGRV B6KGA3 B6KGA3_TOXGO Replication factor a protein 3 protein 111 23-28
154. EDGDRK V4YNF3 V4YNF3_TOXGO Uncharacterized protein 1631 648-653
155. EDGDRK V4Z8K1 V4Z8K1_TOXGO RNA polymerase Rpb1 C-terminal repeat-containing protein 2419 226-231
156. LQNRKL V4ZE60 V4ZE60_TOXGO Internal kinesin motor domain protein 1360 208-213
157. QNRKLV V4ZE60 V4ZE60_TOXGO Internal kinesin motor domain protein 1360 209-214
158. PGGETE V4ZJN8 V4ZJN8_TOXGO Putative clumping factor B 1057 807-812
159. GGETEK B6KFK7 B6KFK7_TOXGO 3'5'-cyclic nucleotide phosphodiesterase domain-containing protein 1281 1259-1264
160. GGETEK B9Q0S6 B9Q0S6_TOXGO Ubiquitin carboxyl-terminal hydrolase UCHL3 264 37-42
161. GGETEK B9Q307 B9Q307_TOXGO Putative transmembrane protein 399 392-397
162. GGETEK Q1JSM5 Q1JSM5_TOXGO Ubiquitin-transferase, putative precursor 12269 1786-1791
163. GGETEK Q6JD66 Q6JD66_TOXGO Eukaryotic initiation factor-2 alpha kinase-A 5072 3921-3926
164. GGETEK V4Z1Z4 V4Z1Z4_TOXGO HECT-domain (Ubiquitin-transferase) domain-containing protein 12299 1845-1850
165. GGETEK V4ZU83 V4ZU83_TOXGO eIF2 kinase IF2K-A (Incomplete catalytic triad) 4638 3487-3492
166. GETEKP B6K9N7 B6K9N7_TOXGO Uncharacterized protein 796 183-188
167. ETEKPR V4Z7U1 V4Z7U1_TOXGO HECT-domain (Ubiquitin-transferase) domain-containing protein 1978 100-105
168. HQEPFV V4YXP4 V4YXP4_TOXGO Dishevelled/Egl-10/leckstrin domain protein 1425 691-696
169. VKPTLS Q1JT06 Q1JT06_TOXGO Uncharacterized protein 1979 1168-1173
170. VKPTLS V4ZPA4 V4ZPA4_TOXGO Uncharacterized protein 102 2-7
171. KPTLSD V4YS05 V4YS05_TOXGO Putative transmembrane protein 2088 1298-1303
172. KPTLSD V4ZAU1 V4ZAU1_TOXGO Putative activating signal cointegrator 1 complex subunit 3 family 1 2207 1060-1065
173. TLSDGT V5BJR6 V5BJR6_TOXGO Pyridine nucleotide-disulfide oxidoreductase domain-containing protein 788 502-507
174. DPVKKV V4ZGE3 V4ZGE3_TOXGO Uncharacterized protein 1241 220-225
175. TSPGSP V4Z0F7 V4Z0F7_TOXGO Putative transmembrane protein 1146 964-969
176. TSPGSP V5BAA4 V5BAA4_TOXGO Zinc finger (CCCH type) motif-containing protein 1934 1512-1517, 1603-1608
177. SPGSPR V4YXH1 V4YXH1_TOXGO Uncharacterized protein 1191 347-352
178. SPGSPR V4YY91 V4YY91_TOXGO Uncharacterized protein 2624 70-75
179. SPGSPR V4Z0F7 V4Z0F7_TOXGO Putative transmembrane protein 1146 965-970
180. SPGSPR V4Z6M7 V4Z6M7_TOXGO Uncharacterized protein 2234 714-719
181. SPGSPR V4ZQK3 V4ZQK3_TOXGO DNA-directed RNA polymerase alpha chain rpoA 1269 159-164
182. SPGSPR V5BL77 V5BL77_TOXGO Inorganic anion transporter, sulfate permease (SulP) family protein 1497 122-127
183. GSPRHT B9Q843 B9Q843_TOXGO Histone lysine methyltransferase SET1 7555 5016-5021
184. PRHTVP V5B323 V5B323_TOXGO Uncharacterized protein 4188 1273-1278
185. HTVPQC V4ZHT6 V4ZHT6_TOXGO Uncharacterized protein 488 268-273
186. VHLVAD B9QGA4 B9QGA4_TOXGO Uncharacterized protein 1572 245-250
187. GTQERV V5B0L4 V5B0L4_TOXGO Thrombospondin type 1 domain-containing protein 876 416-421
188. GELLSG B6KT39 B6KT39_TOXGO EF hand domain-containing protein 3700 3371-3376
189. GELLSG Q2QDG8 Q2QDG8_TOXGO Mitogen-activated protein kinase 683 212-217
190. GELLSG V4Z6R8 V4Z6R8_TOXGO Mitogen-activated protein kinase 671 212-217
191. LLSGQA V4Z685 V4Z685_TOXGO Uncharacterized protein 698 398-403
192. LSGQAD V4YK78 V4YK78_TOXGO Uncharacterized protein 4948 2534-2539
193. ADMIVA V5BCU2 V5BCU2_TOXGO Putative transmembrane protein 1507 1488-1493
194. VAPLTI V4Z814 V4Z814_TOXGO Uncharacterized protein 423 220-225
195. AQYIEF B6KVW8 B6KVW8_TOXGO Ribonucleoside-diphosphate reductase small subunit 391 323-328
196. VKKEIP D0V3Y0 D0V3Y0_TOXGO Formin 1 5051 4315-4320
197. VKKEIP V4YZ27 V4YZ27_TOXGO Formin FRM1 5048 4312-4317
198. PRSTLD B9Q487 B9Q487_TOXGO SWI2/SNF2 ISWI-like (AT hook) 1551 256-261
199. PRSTLD V5AYU3 V5AYU3_TOXGO Transporter, small conductance mechanosensitive ion channel protein 2793 1863-1868
200. RSTLDS V4YN30 V4YN30_TOXGO Putative vacuolar protein sorting-associated protein 8650 5501-5506
201. RSTLDS V4Z9D4 V4Z9D4_TOXGO Putative dynein light chain roadblock-type 2 125 31-36
202. LWLLVG B9PYA4 B9PYA4_TOXGO DEAD/DEAH box helicase domain-containing protein 2434 64-69
203. LWLLVG Q1JTA7 Q1JTA7_TOXGO Dead/deah box helicase, putative 2471 64-69
204. LWLLVG V4ZSD8 V4ZSD8_TOXGO Uncharacterized protein 1556 630-635
205. LWLLVG V5B535 V5B535_TOXGO ImpB/MucB/SamB family protein 1253 470-475
206. LLVGLS B9QG55 B9QG55_TOXGO Putative kynurenine 3-monooxygenase (ISS) protein 1761 1108-1113
207. LLVGLS V4Z225 V4Z225_TOXGO Uncharacterized protein 865 486-491
208. LLVGLS V4ZG45 V4ZG45_TOXGO Putative fatty acyl-CoA desaturase 1042 704-709
209. LVGLSV V5AY70 V5AY70_TOXGO Putative transmembrane protein 2314 1549-1554
210. LVGLSV V5BL34 V5BL34_TOXGO Putative alanine racemase 877 522-527
211. SVHVVA V5BJM2 V5BJM2_TOXGO Putative DNA ligase (NAD+) 1975 1136-1141
212. SPFGRF V4Z4C0 V4Z4C0_TOXGO Uncharacterized protein 2996 807-812
213. VNSEEE B6K8R8 B6K8R8_TOXGO Serine/threonine protein phosphatase 548 42-47
214. SEEEEE B6KBU5 B6KBU5_TOXGO Transcriptional elongation factor FACT80 539 508-513
215. SEEEEE B6KH13 B6KH13_TOXGO Phospholipid-translocating P-type ATPase, flippase subfamily protein 1871 1848-1853
216. SEEEEE B9PTZ1 B9PTZ1_TOXGO NOL1/NOP2/sun family protein 804 145-150
217. SEEEEE B9Q6W4 B9Q6W4_TOXGO Uncharacterized protein 1708 1574-1579
218. SEEEEE B9QGZ2 B9QGZ2_TOXGO Peptidyl-prolyl cis-trans isomerase 426 124-129
219. SEEEEE B9QJX3 B9QJX3_TOXGO Uncharacterized protein 4533 3546-3551
220. SEEEEE B9QK58 B9QK58_TOXGO Translation initiation factor eIF3 subunit 135 4956 1801-1806
221. SEEEEE B9QQ05 B9QQ05_TOXGO DnaJ domain-containing protein 697 308-313
222. SEEEEE Q1JST9 Q1JST9_TOXGO Uncharacterized protein precursor 839 131-136
223. SEEEEE V4YYZ6 V4YYZ6_TOXGO Ribosome biogenesis protein BOP1 homolog 1049 134-139
224. SEEEEE V4Z3P1 V4Z3P1_TOXGO SNF2 family N-terminal domain-containing protein 3406 81-86
225. SEEEEE V4Z3X0 V4Z3X0_TOXGO HEAT repeat-containing protein 1010 453-458
226. SEEEEE V4Z5R3 V4Z5R3_TOXGO HIT zinc finger protein 588 98-103
227. SEEEEE V4Z5W3 V4Z5W3_TOXGO OTU family cysteine protease 988 253-258
228. SEEEEE V4ZA89 V4ZA89_TOXGO Uncharacterized protein 1651 1197-1202
229. SEEEEE V4ZDU9 V4ZDU9_TOXGO Putative transmembrane protein 3661 3613-3618
230. SEEEEE V4ZDY2 V4ZDY2_TOXGO Ribonuclease type III Dicer 4343 4190-4195
231. SEEEEE V4ZED3 V4ZED3_TOXGO Glycosyltransferase family protein 1094 213-218
232. SEEEEE V4ZEM5 V4ZEM5_TOXGO Uncharacterized protein 220 43-48
233. SEEEEE V4ZES6 V4ZES6_TOXGO Transporter, small conductance mechanosensitive ion channel protein 3400 2804-2809
234. SEEEEE V4ZI56 V4ZI56_TOXGO Uncharacterized protein 2083 1390-1395
235. SEEEEE V4ZJY9 V4ZJY9_TOXGO Pre-rRNA processing protein 2863 2415-2420
236. SEEEEE V4ZMH1 V4ZMH1_TOXGO Uncharacterized protein 4312 4128-4133, 4141-4146
237. SEEEEE V4ZPX5 V4ZPX5_TOXGO Surp module domain-containing protein 658 388-393
238. SEEEEE V5AXL6 V5AXL6_TOXGO Tyrosine kinase-like (TKL) protein 3571 956-961
239. SEEEEE V5B0F5 V5B0F5_TOXGO Uncharacterized protein 839 131-136
240. SEEEEE V5B566 V5B566_TOXGO Casein kinase substrate phosphoprotein PP28 176 51-56, 85-90
241. SEEEEE V5B8F2 V5B8F2_TOXGO Peptidase family c50 protein 6890 2567-2572
242. SEEEEE V5BDX3 V5BDX3_TOXGO DNA-directed RNA polymerase III RPC5 827 90-95
243. SEEEEE V5BEA7 V5BEA7_TOXGO Uncharacterized protein 510 288-293
244. SEEEEE V5BFX9 V5BFX9_TOXGO MIF4G domain-containing protein 1433 1203-1208
245. EEEEED B6K8M3 B6K8M3_TOXGO Leucine rich repeat-containing protein 1710 1454-1459
246. EEEEED B6K901 B6K901_TOXGO Nop14-like family protein 1204 484-489, 499-504
247. EEEEED B6K917 B6K917_TOXGO YL1 nuclear protein C-terminal domain-containing protein 737 48-53
248. EEEEED B6KBU5 B6KBU5_TOXGO Transcriptional elongation factor FACT80 539 509-514
249. EEEEED B6KDA4 B6KDA4_TOXGO Putative lanp 283 238-243
250. EEEEED B6KGJ8 B6KGJ8_TOXGO WD domain, G-beta repeat-containing protein 683 394-399
251. EEEEED B6KH13 B6KH13_TOXGO Phospholipid-translocating P-type ATPase, flippase subfamily protein 1871 1849-1854
252. EEEEED B6KJ88 B6KJ88_TOXGO Uncharacterized protein 1230 593-598
253. EEEEED B6KJA4 B6KJA4_TOXGO Acetyltransferase, GNAT family protein 333 316-321
254. EEEEED B6KVB4 B6KVB4_TOXGO B-box zinc finger domain-containing protein 750 724-729, 733-738
255. EEEEED B9PII8 B9PII8_TOXGO Eukaryotic initiation factor-2, alpha subunit 347 315-320
256. EEEEED B9PJK1 B9PJK1_TOXGO Ribosomal protein RPP2 113 100-105
257. EEEEED B9PTZ1 B9PTZ1_TOXGO NOL1/NOP2/sun family protein 804 146-151
258. EEEEED B9PYG5 B9PYG5_TOXGO DEAD/DEAH box helicase domain-containing protein 698 127-132
259. EEEEED B9PZK4 B9PZK4_TOXGO Micro-fibrillar-associated protein 1 438 158-163
260. EEEEED B9PZN1 B9PZN1_TOXGO Uncharacterized protein 195 89-94
261. EEEEED B9Q2S0 B9Q2S0_TOXGO Uncharacterized protein 518 19-24
262. EEEEED B9Q487 B9Q487_TOXGO SWI2/SNF2 ISWI-like (AT hook) 1551 66-71
263. EEEEED B9Q751 B9Q751_TOXGO Subtilisin SUB8 1366 245-250
264. EEEEED B9Q8D8 B9Q8D8_TOXGO Chloroquine resistance marker 3946 1113-1118
265. EEEEED B9QHU8 B9QHU8_TOXGO RIO1 family protein 1008 751-756
266. EEEEED B9QNU4 B9QNU4_TOXGO Hepatocellular carcinoma-associated antigen 59 455 272-277
267. EEEEED B9QPR3 B9QPR3_TOXGO SWI2/SNF2 SRCAP/Ino80 2924 995-1000
268. EEEEED B9QR67 B9QR67_TOXGO Putative at hook motif protein 1282 447-452
269. EEEEED Q1JSP7 Q1JSP7_TOXGO Uncharacterized protein precursor 362 29-34
270. EEEEED Q1JST4 Q1JST4_TOXGO Putative rRNA methyltransferase 981 266-271
271. EEEEED Q1JTB5 Q1JTB5_TOXGO Putative uncharacterized protein 816 493-498, 502-507, 520-525, 533-538
272. EEEEED Q1JTC8 Q1JTC8_TOXGO Eukaryotic translation initiation factor 3 subunit G 295 135-140
273. EEEEED Q1JTF7 Q1JTF7_TOXGO ATP-dependent RNA helicase, putative 574 67-72
274. EEEEED Q1JTI3 Q1JTI3_TOXGO Ubiquitin-protein ligase 1, putative 8112 7521-7526
275. EEEEED Q38LF0 Q38LF0_TOXGO Eukaryotic translation initiation factor 3 subunit G 277 117-122
276. EEEEED Q6JD67 Q6JD67_TOXGO Eukaryotic initiation factor-2 alpha subunit 347 315-320
277. EEEEED Q7Z2C2 Q7Z2C2_TOXGO Snf2-related chromatin remodeling factor SRCAP 2924 995-1000
278. EEEEED V4YLV7 V4YLV7_TOXGO SDA1-like protein 889 590-595, 599-604, 614-619, 627-632
279. EEEEED V4YRP3 V4YRP3_TOXGO Uncharacterized protein 374 315-320, 323-328, 331-336
280. EEEEED V4YWX4 V4YWX4_TOXGO Ubiquitin carboxyl-terminal hydrolase 1029 796-801
281. EEEEED V4YYZ6 V4YYZ6_TOXGO Ribosome biogenesis protein BOP1 homolog 1049 108-113
282. EEEEED V4YZS8 V4YZS8_TOXGO Uncharacterized protein 2525 1044-1049
283. EEEEED V4Z0S4 V4Z0S4_TOXGO Methionine aminopeptidase 2 480 43-48
284. EEEEED V4Z0Z2 V4Z0Z2_TOXGO Uncharacterized protein 1456 1086-1091
285. EEEEED V4Z1T8 V4Z1T8_TOXGO Uncharacterized protein 4885 2030-2035, 2040-2045, 2068-2073, 2078-2083
286. EEEEED V4Z3Q3 V4Z3Q3_TOXGO Uncharacterized protein 111 84-89
287. EEEEED V4Z3X0 V4Z3X0_TOXGO HEAT repeat-containing protein 1010 455-460
288. EEEEED V4Z553 V4Z553_TOXGO HECT-domain (Ubiquitin-transferase) domain-containing protein 8007 7523-7528
289. EEEEED V4Z5P5 V4Z5P5_TOXGO Uncharacterized protein 5771 4343-4348
290. EEEEED V4Z5R3 V4Z5R3_TOXGO HIT zinc finger protein 588 100-105
291. EEEEED V4Z6J1 V4Z6J1_TOXGO Phospholipase, patatin family protein 665 583-588
292. EEEEED V4Z702 V4Z702_TOXGO Sas10 C-terminal domain-containing protein 800 116-121
293. EEEEED V4Z796 V4Z796_TOXGO Uncharacterized protein 593 101-106
294. EEEEED V4Z7I4 V4Z7I4_TOXGO 26S proteasome regulatory subunit, S6a family AAA ATpase 427 89-94
295. EEEEED V4Z801 V4Z801_TOXGO Uncharacterized protein 2495 1063-1068
296. EEEEED V4Z9L5 V4Z9L5_TOXGO Putative transmembrane protein 407 358-363
297. EEEEED V4ZAU1 V4ZAU1_TOXGO Putative activating signal cointegrator 1 complex subunit 3 family 1 ASCC3L1 2207 208-213, 226-231, 232-237
298. EEEEED V4ZBH1 V4ZBH1_TOXGO Uncharacterized protein 3736 2462-2467
299. EEEEED V4ZCB0 V4ZCB0_TOXGO Histone lysine-specific demethylase 3802 1736-1741
300. EEEEED V4ZDY2 V4ZDY2_TOXGO Ribonuclease type III Dicer 4343 3684-3689, 4336-4341
301. EEEEED V4ZE96 V4ZE96_TOXGO 3'5'-cyclic nucleotide phosphodiesterase domain-containing protein 609 148-153
302. EEEEED V4ZED3 V4ZED3_TOXGO Glycosyltransferase family protein 1094 215-220
303. EEEEED V4ZER7 V4ZER7_TOXGO Uncharacterized protein 3307 192-197
304. EEEEED V4ZFP2 V4ZFP2_TOXGO IgA-specific metalloendopeptidase 1814 1581-1586, 1593-1598
305. EEEEED V4ZHB9 V4ZHB9_TOXGO NAD(+)/NADH kinase domain-containing protein 2147 166-171
306. EEEEED V4ZHI4 V4ZHI4_TOXGO Putative SWI2/SNF2 Brahma 2667 2192-2197
307. EEEEED V4ZHU4 V4ZHU4_TOXGO Splicing factor U2AF protein 770 465-470
308. EEEEED V4ZI56 V4ZI56_TOXGO Uncharacterized protein 2083 1391-1396
309. EEEEED V4ZJU6 V4ZJU6_TOXGO S1 RNA binding domain-containing protein 2395 1914-1919
310. EEEEED V4ZJZ9 V4ZJZ9_TOXGO ATPase, AAA family protein 3910 573-578
311. EEEEED V4ZMH1 V4ZMH1_TOXGO Uncharacterized protein 4312 4120-4125, 4142-4147
312. EEEEED V4ZP24 V4ZP24_TOXGO Putative DNA mismatch repair protein MSH6-1 1567 222-227
313. EEEEED V4ZP54 V4ZP54_TOXGO Uncharacterized protein 2445 2116-2121
314. EEEEED V4ZSE4 V4ZSE4_TOXGO WD domain, G-beta repeat-containing protein 4664 4558-4563
315. EEEEED V4ZSF0 V4ZSF0_TOXGO Transporter, major facilitator family protein 719 449-454
316. EEEEED V4ZSF1 V4ZSF1_TOXGO Transporter, major facilitator family protein 2200 4-9
317. EEEEED V4ZVZ1 V4ZVZ1_TOXGO Uncharacterized protein 2363 1771-1776
318. EEEEED V4ZW48 V4ZW48_TOXGO Uncharacterized protein 1243 639-644
319. EEEEED V5AXL6 V5AXL6_TOXGO Tyrosine kinase-like (TKL) protein 3571 828-833, 835-840, 881-886, 959-964, 972-977
320. EEEEED V5AY89 V5AY89_TOXGO Putative CD2 antigen cytoplasmic tail-binding protein 2 565 256-261
321. EEEEED V5AYC6 V5AYC6_TOXGO Putative replication licensing factor 972 92-97
322. EEEEED V5AYN9 V5AYN9_TOXGO Cyclic nucleotide-binding domain-containing protein 2723 905-910
323. EEEEED V5B4G9 V5B4G9_TOXGO Uncharacterized protein 2072 1283-1288
324. EEEEED V5B566 V5B566_TOXGO Casein kinase substrate phosphoprotein PP28 176 52-57
325. EEEEED V5B7T9 V5B7T9_TOXGO Uncharacterized protein 838 734-739
326. EEEEED V5BDX3 V5BDX3_TOXGO DNA-directed RNA polymerase III RPC5 827 96-101
327. EEEEED V5BLF5 V5BLF5_TOXGO Uncharacterized protein 703 482-487
328. EEEEDA B6KFS2 B6KFS2_TOXGO XRN 5'-3' exonuclease N-terminus protein 2042 682-687
329. EEEEDA B6KJ88 B6KJ88_TOXGO Uncharacterized protein 1230 594-599
330. EEEEDA B9QHU8 B9QHU8_TOXGO RIO1 family protein 1008 752-757
331. EEEEDA V4YWX4 V4YWX4_TOXGO Ubiquitin carboxyl-terminal hydrolase 1029 780-785
332. EEEEDA V4Z0C3 V4Z0C3_TOXGO Myb family DNA-binding domain-containing protein 1913 1159-1164
333. EEEEDA V4Z6U9 V4Z6U9_TOXGO Phosphoglycerate mutase family protein 1971 1934-1939
334. EEEEDA V4Z702 V4Z702_TOXGO Sas10 C-terminal domain-containing protein 800 117-122
335. EEEEDA V4Z801 V4Z801_TOXGO Uncharacterized protein 2495 1064-1069
336. EEEEDA V4ZAU1 V4ZAU1_TOXGO Putative activating signal cointegrator 1 complex subunit 3 family 1 ASCC3L1 2207 209-214, 233-238
337. EEEEDA V4ZM65 V4ZM65_TOXGO Methyltransferase 1270 993-998
338. EEEEDA V4ZW70 V4ZW70_TOXGO Uncharacterized protein 5655 3219-3224, 3225-3230
339. EEEEDA V5B4U0 V5B4U0_TOXGO Uncharacterized protein 325 238-243
340. EEEEDA V5B9Z0 V5B9Z0_TOXGO Folate receptor family protein 526 315-320
341. EEEDAL B6K9Y2 B6K9Y2_TOXGO Transcription elongation factor SPT6 3132 317-322
342. EEEDAL V4Z845 V4Z845_TOXGO CW-type Zinc Finger protein 1673 199-204
343. EEEDAL V5AY44 V5AY44_TOXGO Putative transmembrane protein 7450 5601-5606
344. EEDALT V5BKE2 V5BKE2_TOXGO Uncharacterized protein 886 320-325
345. EDALTL V5BKE2 V5BKE2_TOXGO Uncharacterized protein 886 321-326
346. ALTLSS B9PY21 B9PY21_TOXGO ACR-like protein 1530 890-895
347. ALTLSS V5B2B4 V5B2B4_TOXGO Phosphoserine phosphatase 1766 1391-1396
348. LTLSSA Q1JSA7 Q1JSA7_TOXGO Uncharacterized protein 2639 586-591
349. LTLSSA V4YMT2 V4YMT2_TOXGO Uncharacterized protein 2796 586-591
350. LTLSSA V4ZSE8 V4ZSE8_TOXGO DUF803 domain-containing protein 814 627-632
351. FSWGVL V4ZDU9 V4ZDU9_TOXGO Putative transmembrane protein 3661 381-386
352. SGIGEG B9PUQ4 B9PUQ4_TOXGO Uncharacterized protein 288 118-123
353. GIGEGA B9PUQ4 B9PUQ4_TOXGO Uncharacterized protein 288 119-124
354. GIGEGA B9QQF5 B9QQF5_TOXGO Uncharacterized protein 976 25-30
355. GEGAPR B9QJI0 B9QJI0_TOXGO Histone lysine acetyltransferase GCN5-B 1032 846-851
356. GEGAPR Q5EK48 Q5EK48_TOXGO GNAT family histone acetyltransferase GCN5-B 1032 846-851
357. GEGAPR V4YT41 V4YT41_TOXGO Protein kinase domain protein 6052 2881-2886
358. GEGAPR V4Z251 V4Z251_TOXGO Uncharacterized protein 3633 3447-3452
359. EGAPRS V5BES6 V5BES6_TOXGO Uncharacterized protein 1006 216-221
360. GAPRSF B9PSX5 B9PSX5_TOXGO Uncharacterized protein 599 12-17
361. APRSFS B6KR18 B6KR18_TOXGO Putative cell-cycle-associated protein kinase CDK 1372 64-69
362. APRSFS V4ZN45 V4ZN45_TOXGO Subtilisin SUB3 969 829-834
363. PRSFSA B6KR18 B6KR18_TOXGO Putative cell-cycle-associated protein kinase CDK 1372 65-70
364. PRSFSA V4Z2H4 V4Z2H4_TOXGO Methionine aminopeptidase 697 330-335
365. PRSFSA V4ZG34 V4ZG34_TOXGO Polycystin cation channel protein 1261 559-564
366. PRSFSA V5BBQ8 V5BBQ8_TOXGO Leucine rich repeat-containing protein 4458 1423-1428
367. RSFSAR B6K8I0 B6K8I0_TOXGO Zinc finger (CCCH type) motif-containing protein 920 611-616
368. RSFSAR V4ZM71 V4ZM71_TOXGO Putative autophagy-related cysteine peptidase atg4 3747 3428-3433
369. SARILG V4ZS03 V4ZS03_TOXGO Oxidoreductase, aldo/keto reductase family protein 1485 862-867
370. YTANLA B9QM08 B9QM08_TOXGO Zinc finger (CCCH type) motif-containing protein 1199 827-832
371. TANLAA B9QM08 B9QM08_TOXGO Zinc finger (CCCH type) motif-containing protein 1199 828-833
372. ANLAAF V4ZTQ1 V4ZTQ1_TOXGO DEAD/DEAH box helicase domain-containing protein 1991 304-309
373. NLAAFL V4ZTQ1 V4ZTQ1_TOXGO DEAD/DEAH box helicase domain-containing protein 1991 305-310
374. LAAFLV V4YQA3 V4YQA3_TOXGO Serine esterase (DUF676) protein 1565 654-659
375. LAAFLV V5B190 V5B190_TOXGO Rhoptry kinase family protein ROP26 (Incomplete catalytic triad) 435 241-246
376. AAFLVL A7UDC8 A7UDC8_TOXGO Secretory rhoptry protein 4 578 18-23
377. AAFLVL B6E132 B6E132_TOXGO ROP7 protein 575 18-23
378. AAFLVL B9QL29 B9QL29_TOXGO Putative rhoptry protein 102 18-23
379. AAFLVL G4XMX9 G4XMX9_TOXGO Rhoptry protein 7 575 18-23
380. AAFLVL G4XMY0 G4XMY0_TOXGO Rhoptry protein 7 575 18-23
381. AAFLVL G4XMY1 G4XMY1_TOXGO Rhoptry protein 7 575 18-23
382. AAFLVL G4XMY3 G4XMY3_TOXGO Rhoptry protein 7 575 18-23
383. AAFLVL G4XMY5 G4XMY5_TOXGO Rhoptry protein 7 575 18-23
384. AAFLVL G4XMY7 G4XMY7_TOXGO Rhoptry protein 7 575 18-23
385. AAFLVL G4XMZ0 G4XMZ0_TOXGO Rhoptry protein 7 575 18-23
386. AAFLVL G4XMZ1 G4XMZ1_TOXGO Rhoptry protein 7 575 18-23
387. AAFLVL G4XMZ2 G4XMZ2_TOXGO Rhoptry protein 7 575 18-23
388. AAFLVL G4XMZ3 G4XMZ3_TOXGO Rhoptry protein 7 575 18-23
389. AAFLVL G4XMZ4 G4XMZ4_TOXGO Rhoptry protein 7 575 18-23
390. AAFLVL G4XMZ5 G4XMZ5_TOXGO Rhoptry protein 7 575 18-23
391. AAFLVL G4XMZ6 G4XMZ6_TOXGO Rhoptry protein 7 575 18-23
392. AAFLVL G4XMZ7 G4XMZ7_TOXGO Rhoptry protein 7 575 18-23
393. AAFLVL Q1JTG7 Q1JTG7_TOXGO Rhoptry protein 4 precursor 575 18-23
394. AAFLVL Q2PB65 Q2PB65_TOXGO Rhoptry protein 7 575 18-23
395. AAFLVL Q5Y808 ROP4_TOXGO Rhoptry protein 4 precursor 578 18-23
396. AAFLVL V4Z117 V4Z117_TOXGO Rhoptry protein ROP4 365 18-23
397. AAFLVL V5B6C6 V5B6C6_TOXGO Uncharacterized protein 1034 311-316
398. AFLVLD B9QHE7 B9QHE7_TOXGO Putative transmembrane protein 536 25-30
399. FLVLDR B9PN86 B9PN86_TOXGO Uncharacterized protein 449 188-193
400. FLVLDR V4ZBH7 V4ZBH7_TOXGO Uncharacterized protein 389 294-299
401. DRPEER B9QIL7 B9QIL7_TOXGO Uncharacterized protein 1172 435-440
402. DRPEER V4Z1P8 V4Z1P8_TOXGO PRPF39, related protein 2831 1212-1217
403. DPRLRN V5BDM3 V5BDM3_TOXGO Sec7 domain-containing protein 3987 2700-2705
404. TVKQSS B9PYE4 B9PYE4_TOXGO Putative type I fatty acid synthase 10021 4912-4917, 6663-6668
405. TVKQSS Q1JTE1 Q1JTE1_TOXGO Type I fatty acid synthase, putative 9940 4831-4836, 6582-6587
406. VKQSSV B9QH93 B9QH93_TOXGO Putative ppg3 1271 160-165
407. FRRQVE V4ZWS2 V4ZWS2_TOXGO Uncharacterized protein 4834 1457-1462
408. RRQVEL V4ZFD9 V4ZFD9_TOXGO RAP domain-containing protein 1669 1604-1609
409. LSTMYR B9QBY4 B9QBY4_TOXGO Zinc finger (CCCH type) motif-containing protein 899 336-341
410. ESAAEA V4YQJ6 V4YQJ6_TOXGO Uncharacterized protein 2197 1223-1228
411. AAEAIQ V5B7V4 V5B7V4_TOXGO tRNA pseudouridine synthase D 1489 870-875
412. AEAIQA V4Z254 V4Z254_TOXGO Uncharacterized protein 2395 2323-2328
413. AIQAVR V5BDB0 V5BDB0_TOXGO Putative tRNA (Guanine(26)-N(2))-dimethyltransferase 697 323-328
414. IWDSAV V4ZHW7 V4ZHW7_TOXGO Uncharacterized protein 822 322-327
415. DSAVLE V4ZGU8 V4ZGU8_TOXGO Zinc finger in N-recognin protein 4383 3670-3675
416. SAVLEF B9QMH6 B9QMH6_TOXGO RNA pseudouridine synthase superfamily protein 6535 4980-4985
417. AVLEFE B6KFN1 B6KFN1_TOXGO B-box zinc finger domain-containing protein 1073 220-225
418. SQKCDL V4ZJP8 V4ZJP8_TOXGO Amine-terminal region of chorein, A TM vesicle-mediated sorter 12207 4522-4527
419. VTTGEL V4YIL6 V4YIL6_TOXGO Toxoplasma gondii family B protein 258 113-118
420. VTTGEL V5B884 V5B884_TOXGO SWI2/SNF2-containing protein RAD26 1590 1520-1525
421. TGELFF V4ZDK9 V4ZDK9_TOXGO Uncharacterized protein 2679 1103-1108
422. GELFFR V4Z8Z4 V4Z8Z4_TOXGO Putative vacuolar protein sorting-associated protein 26 368 62-67
423. GFGIGM B9PIV3 B9PIV3_TOXGO Putative pyruvate dehydrogenase E1 component, beta subunit 423 163-168
424. QNVSLS V4YQT3 V4YQT3_TOXGO Putative transmembrane protein 3329 2931-2936
425. QNVSLS V4Z9H2 V4Z9H2_TOXGO Uncharacterized protein 824 362-367
426. QNVSLS V5AY44 V5AY44_TOXGO Putative transmembrane protein 7450 1965-1970
427. SRSNAP V4ZDY3 V4ZDY3_TOXGO DNA-directed DNA polymerase 1221 294-299
428. LVAGGI V4ZTH9 V4ZTH9_TOXGO Putative transmembrane protein 1278 939-944
429. AGGIVA B9Q479 B9Q479_TOXGO WD domain, G-beta repeat-containing protein 3633 3303-3308
430. GGIVAG A4GT85 A4GT85_TOXGO Sugar transporter 689 347-352
431. GGIVAG V4ZIE7 V4ZIE7_TOXGO Uncharacterized protein 742 499-504
432. GGIVAG V5BMB1 V5BMB1_TOXGO Protein phosphatase 2C domain-containing protein 1432 565-570
433. GIVAGI V5AXR6 V5AXR6_TOXGO Uncharacterized protein 1481 889-894
434. GIVAGI V5BBP7 V5BBP7_TOXGO Putative transmembrane protein 1377 1065-1070
435. DARRKQ V4YNF3 V4YNF3_TOXGO Uncharacterized protein 1631 657-662
436. MQLAFA V4ZJH2 V4ZJH2_TOXGO RNA recognition motif-containing protein 2070 617-622
437. QLAFAA V4ZG01 V4ZG01_TOXGO Uncharacterized protein 1001 225-230
438. LAFAAV B9QG55 B9QG55_TOXGO Putative kynurenine 3-monooxygenase and-related flavoprotein monooxygenase family (ISS) protein 1761 1427-1432
439. AFAAVN V4ZDV1 V4ZDV1_TOXGO Uncharacterized protein 815 443-448
440. DRKSGR B6KRG7 B6KRG7_TOXGO Protein phosphatase 2C domain-containing protein 554 537-542
441. KSGRAE B6KH95 B6KH95_TOXGO Uncharacterized protein 3071 1032-1037
442. KSGRAE V4ZRG6 V4ZRG6_TOXGO SWI2/SNF2-containing protein 1224 707-712
443. SGRAEP V4ZMP4 V4ZMP4_TOXGO Uncharacterized protein 1583 1183-1188
444. GRAEPD B9PQV4 B9PQV4_TOXGO Putative transporter 728 712-717
445. GRAEPD V4ZBY4 V4ZBY4_TOXGO Uncharacterized protein 1239 974-979
446. TSTLAS V4ZDG6 V4ZDG6_TOXGO WD domain, G-beta repeat-containing protein 1944 493-498
447. TSTLAS V4ZNP4 V4ZNP4_TOXGO Putative GRIP domain protein 921 85-90
448. TSTLAS V4ZW70 V4ZW70_TOXGO Uncharacterized protein 5655 2496-2501
449. STLASS B6KHE4 B6KHE4_TOXGO DNA repair protein rad10 subfamily protein 409 99-104, 111-116, 119-124, 127-132, 135-140
450. STLASS V4ZAC6 V4ZAC6_TOXGO Vps52 / Sac2 family protein 789 381-386
451. STLASS V4ZBH2 V4ZBH2_TOXGO DnaJ domain-containing protein 645 335-340
452. STLASS V4ZC76 V4ZC76_TOXGO Phosphofructokinase domain-containing protein 3001 794-799
453. STLASS V5B9K8 V5B9K8_TOXGO Uncharacterized protein 577 334-339
454. STLASS V5BHA1 V5BHA1_TOXGO Uncharacterized protein 5632 2989-2994
455. TLASSF V4YNL5 V4YNL5_TOXGO Uncharacterized protein 2072 1586-1591
456. TLASSF V4Z2V1 V4Z2V1_TOXGO Fumble protein 1226 804-809
457. TLASSF V4ZE02 V4ZE02_TOXGO Dynein heavy chain family protein 3987 535-540
458. SFKRRR Q1JSF7 Q1JSF7_TOXGO Uncharacterized protein 928 601-606
459. SFKRRR V5B0Y5 V5B0Y5_TOXGO RNA recognition motif-containing protein 589 262-267
460. KRRRSS B9Q591 B9Q591_TOXGO Putative transmembrane protein 7354 6351-6356
461. KRRRSS D3XAQ2 D3XAQ2_TOXGO Methionyl-tRNA formyltransferase 885 838-843
462. KRRRSS Q1JSM5 Q1JSM5_TOXGO Ubiquitin-transferase, putative precursor 12269 648-653
463. KRRRSS V4Z1Z4 V4Z1Z4_TOXGO HECT-domain (Ubiquitin-transferase) domain-containing protein 12299 648-653
464. KRRRSS V4Z826 V4Z826_TOXGO Dual specificity phosphatase, catalytic domain-containing protein 897 784-789
465. KRRRSS V4ZDV4 V4ZDV4_TOXGO HEAT repeat-containing protein 2373 1235-1240
466. KRRRSS V4ZIX0 V4ZIX0_TOXGO Galactosyltransferase amine-terminal domain protein 1109 848-853
467. KRRRSS V4ZLP1 V4ZLP1_TOXGO Uncharacterized protein 272 210-215
468. KRRRSS V5B5B5 V5B5B5_TOXGO Formyl transferase domain-containing protein 885 838-843
469. RRRSSK Q1JSY4 Q1JSY4_TOXGO Uncharacterized protein 3344 2025-2030
470. RRRSSK V4Z1R2 V4Z1R2_TOXGO GCC2 and GCC3 domain-containing protein 5081 3794-3799
471. SSKDTS B9Q6I9 B9Q6I9_TOXGO Uncharacterized protein 5651 1383-1388
472. TSTGGG V4Z893 V4Z893_TOXGO Uncharacterized protein 740 189-194
473. STGGGR B9QCR4 B9QCR4_TOXGO Pentatricopeptide repeat domain-containing protein 2141 74-79
474. STGGGR B9QMH6 B9QMH6_TOXGO RNA pseudouridine synthase superfamily protein 6535 618-623
475. STGGGR V4Z9F5 V4Z9F5_TOXGO Cullin family protein 2442 1983-1988
476. STGGGR V4ZHU0 V4ZHU0_TOXGO ATPase, AAA family protein 1031 774-779
477. TGGGRG B9PY86 B9PY86_TOXGO Uncharacterized protein 2407 424-429
478. TGGGRG B9QAM8 B9QAM8_TOXGO Uncharacterized protein 2194 1575-1580
479. TGGGRG Q1JT89 Q1JT89_TOXGO Putative uncharacterized protein 2393 410-415
480. TGGGRG V4Z254 V4Z254_TOXGO Uncharacterized protein 2395 111-116
481. TGGGRG V4Z774 V4Z774_TOXGO Putative glycosylphosphatidylinositol anchor attachment protein 1 1011 462-467
482. TGGGRG V4Z845 V4Z845_TOXGO CW-type Zinc Finger protein 1673 473-478
483. GGGRGA B6KGF8 B6KGF8_TOXGO Carrier superfamily protein 850 16-21
484. GGGRGA B9PXE5 B9PXE5_TOXGO tRNA-splicing ligase RtcB homolog 519 66-71
485. GGGRGA B9PY86 B9PY86_TOXGO Uncharacterized protein 2407 425-430
486. GGGRGA B9QCL2 B9QCL2_TOXGO Ubiquitin-conjugating enzyme subfamily protein 275 6-11
487. GGGRGA Q1JT89 Q1JT89_TOXGO Putative uncharacterized protein 2393 411-416
488. GGGRGA V4YPS5 V4YPS5_TOXGO Uncharacterized protein 1776 1117-1122
489. GGGRGA V4YV01 V4YV01_TOXGO Putative transmembrane protein 1346 634-639
490. GGGRGA V4Z845 V4Z845_TOXGO CW-type Zinc Finger protein 1673 474-479
491. GGGRGA V4Z8J6 V4Z8J6_TOXGO Uncharacterized protein 319 93-98
492. GGGRGA V4ZD39 V4ZD39_TOXGO Uncharacterized protein 3436 792-797
493. GRGALQ V4ZPY4 V4ZPY4_TOXGO RAVE 1 carboxy-terminal protein 6665 4851-4856
494. RGALQN B6K917 B6K917_TOXGO YL1 nuclear protein C-terminal domain-containing protein 737 662-667
495. RGALQN V4Z3I5 V4Z3I5_TOXGO Transporter, major facilitator family protein 636 516-521
496. TVLPRR V4ZDQ0 V4ZDQ0_TOXGO Glycine cleavage T-protein domain-containing protein 1807 5-10
497. VLPRRA B9Q562 B9Q562_TOXGO Non-specific serine/threonine protein kinase 8428 693-698
498. RRAIER V4Z766 V4Z766_TOXGO Uncharacterized protein 1905 866-871
499. RRAIER V5BIK7 V5BIK7_TOXGO Putative exosomal 3'-5' exoribonuclease complex subunit 458 220-225
500. EREEGQ B9Q5K9 B9Q5K9_TOXGO Uncharacterized protein 1656 553-558
501. EREEGQ V4Z3B0 V4Z3B0_TOXGO Uncharacterized protein 271 82-87
502. EREEGQ V4Z8H3 V4Z8H3_TOXGO Pyridoxal-dependent decarboxylase domain protein 970 319-324
503. EREEGQ V5B6J0 V5B6J0_TOXGO RNA polymerase I specific transcription initiation factor RRN3 3150 777-782
504. EEGQLQ V4YP36 V4YP36_TOXGO Uncharacterized protein 2331 1556-1561

**NMDA 2A**

1. WTLLVL Q1JTL1 Q1JTL1_TOXGO Putative uncharacterized protein 1525 420-425
2. WTLLVL V5AZJ8 V5AZJ8_TOXGO Vps54 family protein 1983 915-920
3. LLVLPA B9Q562 B9Q562_TOXGO Non-specific serine/threonine protein kinase 8428 5917-5922
4. LLVLPA K7WFS5 K7WFS5_TOXGO Palmitoyltransferase 371 85-90
5. LLVLPA V5BH73 V5BH73_TOXGO Elongation factor Tu GTP binding domain-containing protein 2560 1430-1435
6. LVLPAL B9Q562 B9Q562_TOXGO Non-specific serine/threonine protein kinase 8428 5918-5923
7. LVLPAL B9QNR2 B9QNR2_TOXGO Uncharacterized protein 3029 1556-1561
8. VLPALL B6K8J1 B6K8J1_TOXGO Putative transmembrane protein 4690 1969-1974
9. VLPALL B9Q562 B9Q562_TOXGO Non-specific serine/threonine protein kinase 8428 5919-5924
10. VLPALL V4Z5P5 V4Z5P5_TOXGO Uncharacterized protein 5771 3310-3315
11. LPALLV B6K8Q8 B6K8Q8_TOXGO Plasma membrane-type Ca(2+)-ATPase A1 PMCAA1 1822 228-233
12. LPALLV B9QB74 B9QB74_TOXGO NOL1/NOP2/Sun family protein 919 484-489
13. ALLVWR V4ZFZ2 V4ZFZ2_TOXGO Sec7 domain-containing protein 3546 2041-2046
14. LLVWRG V4YY04 V4YY04_TOXGO Uncharacterized protein 129 80-85
15. PAPSAA A8CBF6 A8CBF6_TOXGO Delta-aminolevulinic acid synthetase 584 15-20
16. PAPSAA B6KH95 B6KH95_TOXGO Uncharacterized protein 3071 1236-1241
17. PAPSAA B6KQK7 B6KQK7_TOXGO Cyclin protein 2572 853-858
18. PAPSAA B9QQ97 B9QQ97_TOXGO Uncharacterized protein 1284 317-322
19. PAPSAA Q1JSA3 Q1JSA3_TOXGO Uncharacterized protein 648 493-498
20. PAPSAA Q1JT27 Q1JT27_TOXGO Putative uncharacterized protein 2626 907-912
21. PAPSAA V4Z138 V4Z138_TOXGO Gamma interferon inducible lysosomal thiol reductase (GILT) protein 380 175-180
22. PAPSAA V4Z4P9 V4Z4P9_TOXGO Uncharacterized protein 2174 864-869
23. PAPSAA V4Z4S7 V4Z4S7_TOXGO Uncharacterized protein 1150 291-296
24. PAPSAA V4Z843 V4Z843_TOXGO Uncharacterized protein 1132 347-352
25. PAPSAA V4ZIT3 V4ZIT3_TOXGO 5-aminolevulinic acid synthase domain-containing protein 752 78-83
26. PAPSAA V5B115 V5B115_TOXGO Ubiquitin-like protein ATG12 681 493-498
27. APSAAA A8CBF6 A8CBF6_TOXGO Delta-aminolevulinic acid synthetase 584 16-21
28. APSAAA B6KH95 B6KH95_TOXGO Uncharacterized protein 3071 1237-1242
29. APSAAA B9PQM3 B9PQM3_TOXGO Ran binding protein 367 200-205
30. APSAAA B9QFR6 B9QFR6_TOXGO NUC173 domain protein 1915 1166-1171
31. APSAAA V4Z138 V4Z138_TOXGO Gamma interferon inducible lysosomal thiol reductase (GILT) protein 380 176-181
32. APSAAA V4Z704 V4Z704_TOXGO Uncharacterized protein 538 264-269
33. APSAAA V4ZCX8 V4ZCX8_TOXGO Uncharacterized protein 418 163-168
34. APSAAA V4ZD18 V4ZD18_TOXGO Uncharacterized protein 3221 952-957
35. APSAAA V4ZIT3 V4ZIT3_TOXGO 5-aminolevulinic acid synthase domain-containing protein 752 79-84
36. APSAAA V4ZN18 V4ZN18_TOXGO RbAp48 914 428-433
37. APSAAA V4ZTH9 V4ZTH9_TOXGO Putative transmembrane protein 1278 979-984
38. APSAAA V5B919 V5B919_TOXGO DEAD/DEAH box helicase domain-containing protein 1841 450-455
39. APSAAA V5BDC0 V5BDC0_TOXGO Uncharacterized protein 1391 1226-1231
40. PSAAAE B9QIT9 B9QIT9_TOXGO Glycosyl hydrolase, family 31 protein 1618 1433-1438
41. PSAAAE B9QMG4 B9QMG4_TOXGO Putative transmembrane protein 1215 632-637
42. PSAAAE V4Z2V1 V4Z2V1_TOXGO Fumble protein 1226 1154-1159
43. PSAAAE V4Z718 V4Z718_TOXGO Concanavalin A-like lectin/glucanase family protein 1494 1266-1271
44. PSAAAE V4ZGA9 V4ZGA9_TOXGO GTP1/Obg protein 1917 1820-1825
45. SAAAEK B9Q1V7 B9Q1V7_TOXGO Putative integral membrane protein 302 122-127
46. SAAAEK B9Q7W5 B9Q7W5_TOXGO Uncharacterized protein 2115 1032-1037
47. SAAAEK B9QER6 B9QER6_TOXGO Uncharacterized protein 1233 439-444
48. SAAAEK B9QKQ0 B9QKQ0_TOXGO SNF2 family amine-terminal domain protein 1465 183-188
49. SAAAEK V4ZDG3 V4ZDG3_TOXGO Uncharacterized protein 855 18-23
50. AAAEKG A8CBF8 A8CBF8_TOXGO Delta-aminolevulinic acid dehydratase 497 442-447
51. AAAEKG A8CBG0 A8CBG0_TOXGO Delta-aminolevulinic acid dehydratase 407 352-357
52. AAAEKG A8CBG3 A8CBG3_TOXGO Delta-aminolevulinic acid dehydratase 693 638-643
53. AAAEKG B9PQ71 B9PQ71_TOXGO Eukaryotic translation initiation factor 3 subunit M 463 86-91
54. AAAEKG B9Q4U1 B9Q4U1_TOXGO Amine-terminal region of chorein, A TM vesicle-mediated sorter 10329 8551-8556
55. AAAEKG B9QJ12 B9QJ12_TOXGO Delta-aminolevulinic acid dehydratase 662 607-612
56. AAAEKG V4Z2X9 V4Z2X9_TOXGO Pseudouridine synthase 640 428-433
57. AAAEKG V4ZL93 V4ZL93_TOXGO Uncharacterized protein 1323 390-395
58. AAEKGP V4ZAZ4 V4ZAZ4_TOXGO AP2 domain transcription factor AP2X-7 1869 149-154
59. AAEKGP V4ZHG0 V4ZHG0_TOXGO GDA1/CD39 (Nucleoside phosphatase) family protein 696 638-643
60. KGPPAL V4YY91 V4YY91_TOXGO Uncharacterized protein 2624 346-351
61. KGPPAL V4ZAJ9 V4ZAJ9_TOXGO Putative transmembrane protein 617 129-134
62. TERELR Q1JTC9 Q1JTC9_TOXGO Rhomboid protease ROM5 841 22-27
63. TERELR Q6GV23 RHBL5_TOXGO Rhomboid-like protease 5 841 22-27
64. TERELR V4ZGA9 V4ZGA9_TOXGO GTP1/Obg protein 1917 1656-1661
65. ERELRT V4YXP4 V4YXP4_TOXGO Dishevelled/Egl-10/leckstrin domain protein 1425 242-247
66. ERELRT V4ZL39 V4ZL39_TOXGO Zn-finger in ubiquitin-hydrolases domain-containing protein 2582 1970-1975
67. RELRTL V4ZBJ2 V4ZBJ2_TOXGO Tyrosine kinase-like (TKL) protein 1673 1400-1405
68. GPEQAA V4Z4Q5 V4Z4Q5_TOXGO Uncharacterized protein 850 55-60
69. GPEQAA V4ZIP7 V4ZIP7_TOXGO Eukaryotic initiation factor 4E 2044 271-276
70. PEQAAG V5B8L5 V5B8L5_TOXGO IQ calmodulin-binding motif domain-containing protein 1179 195-200
71. EQAAGL Q1JSU8 Q1JSU8_TOXGO Uncharacterized protein 320 287-292
72. EQAAGL V5B0A7 V5B0A7_TOXGO Putative transmembrane protein 1740 1375-1380
73. QAAGLP V4YUG0 V4YUG0_TOXGO Glutamate-tRNA ligase 828 511-516
74. AAGLPL Q1JT47 Q1JT47_TOXGO MoeA N-terminal region (Domain I and II) domain-containing protein 1255 363-368
75. AAGLPL V4ZKE7 V4ZKE7_TOXGO AP2 domain transcription factor AP2X-1 1292 498-503
76. AAGLPL V5BDN4 V5BDN4_TOXGO General transcription factor IIH polypeptide 3 GTF2H3 549 255-260
77. AGLPLD V4ZH73 V4ZH73_TOXGO Uncharacterized protein 5847 1698-1703
78. ALLMNR B9QM39 B9QM39_TOXGO Kinase, pfkB family protein 439 85-90
79. RTDPKS B9Q7H9 B9Q7H9_TOXGO Uncharacterized protein 2408 222-227
80. TDPKSL V5BG60 V5BG60_TOXGO Putative transmembrane protein 351 228-233
81. ARIHGL B9PTG1 B9PTG1_TOXGO WD-40 repeat protein 521 332-337
82. DTDQEA V4Z4I1 V4Z4I1_TOXGO PLU-1 family protein 8088 995-1000
83. TDQEAV V4YYQ2 V4YYQ2_TOXGO Protein phosphatase 2C domain-containing protein 548 504-509
84. AVAQML B6KNZ2 B6KNZ2_TOXGO Rhoptry kinase family protein ROP36 (Incomplete catalytic triad) 624 161-166
85. AVAQML Q1JSY1 Q1JSY1_TOXGO Uncharacterized protein precursor 624 161-166
86. LDFISS V4ZBJ7 V4ZBJ7_TOXGO DNA polymerase 2975 616-621
87. DFISSH V4ZI18 V4ZI18_TOXGO Zinc finger (CCCH type) motif-containing protein 1570 202-207
88. FISSHT V4ZI18 V4ZI18_TOXGO Zinc finger (CCCH type) motif-containing protein 1570 203-208
89. VPILGI B9QFS2 B9QFS2_TOXGO p-aminobenzoic acid synthase 988 109-114
90. ASIQQQ B9QEC7 B9QEC7_TOXGO CCR4-Not complex component, Not1 protein 2562 391-396
91. VFSLVT V4Z894 V4Z894_TOXGO ATPase family associated with various cellular activities (AAA) subfamily protein 9030 4834-4839
92. FSLVTT V4YPN9 V4YPN9_TOXGO ATPase, AAA family protein 1299 665-670
93. GYREFI B9PSM1 B9PSM1_TOXGO Putative transmembrane protein 319 276-281
94. REFISF V4Z034 V4Z034_TOXGO Uncharacterized protein 1634 124-129
95. ISFVKT V4ZS91 V4ZS91_TOXGO Inositol polyphosphate kinase 1573 288-293
96. VKTTVD B9QEB0 B9QEB0_TOXGO 8-amino-7-oxononanoate synthase 577 558-563
97. EDAKTQ Q1JSC0 Q1JSC0_TOXGO Uncharacterized protein precursor 181 52-57
98. EDAKTQ V5B135 V5B135_TOXGO Uncharacterized protein 1054 925-930
99. LKKIHS V5BDB0 V5BDB0_TOXGO Putative tRNA (Guanine(26)-N(2))-dimethyltransferase 697 183-188
100. SSVILL Q1JSM9 Q1JSM9_TOXGO Uncharacterized protein 3444 1635-1640
101. SSVILL Q1JTI3 Q1JTI3_TOXGO Ubiquitin-protein ligase 1, putative 8112 759-764
102. SSVILL V4Z553 V4Z553_TOXGO HECT-domain (Ubiquitin-transferase) domain-containing protein 8007 759-764
103. DEAVLI B9QP58 B9QP58_TOXGO Putative electron transfer flavoprotein subunit beta 275 86-91
104. AVLILS B6KBH4 B6KBH4_TOXGO Nucleoporin autopeptidase 2894 2710-2715
105. AVLILS V5B3V7 V5B3V7_TOXGO Putative transmembrane protein 256 120-125
106. LSEARS V4ZNF6 V4ZNF6_TOXGO Putative glutamic acid-rich protein 2698 2543-2548
107. SEARSL A4L9T5 A4L9T5_TOXGO Seryl-tRNA synthase 2 918 26-31
108. SEARSL V4Z7K9 V4Z7K9_TOXGO Serine--tRNA ligase 367 26-31
109. EARSLG B6K9J1 B6K9J1_TOXGO Inner centromere protein, ARK-binding region protein 1331 1245-1250
110. EARSLG B9Q751 B9Q751_TOXGO Subtilisin SUB8 1366 24-29
111. ARSLGL B9Q751 B9Q751_TOXGO Subtilisin SUB8 1366 25-30
112. RSLGLT V4ZBQ6 V4ZBQ6_TOXGO Putative transmembrane domain protein 878 847-852
113. SLGLTG B9Q836 B9Q836_TOXGO Aconitate hydratase ACN/IRP 1055 985-990
114. SLGLTG Q6DUK1 Q6DUK1_TOXGO Iron regulatory protein-like protein 1055 985-990
115. SLGLTG V4ZIY3 V4ZIY3_TOXGO Rhoptry kinase family protein ROP27 975 417-422
116. SLGLTG V4ZJ87 V4ZJ87_TOXGO Putative transmembrane protein 471 353-358
117. SLGLTG V5B2X9 V5B2X9_TOXGO CAAX metallo endopeptidase 232 161-166
118. LGLTGY B9QAR8 B9QAR8_TOXGO Uncharacterized protein 173 18-23
119. LTGYDF V4Z4W1 V4Z4W1_TOXGO Putative target of rapamycin (TOR) 4922 4883-4888
120. IVPSLV Q1JSG4 Q1JSG4_TOXGO Uncharacterized protein 288 75-80
121. VPSLVS B9Q792 B9Q792_TOXGO Uncharacterized protein 4118 118-123
122. VPSLVS B9QF55 B9QF55_TOXGO AP2 domain transcription factor AP2VIIa-3 998 624-629
123. VPSLVS Q1JSF3 Q1JSF3_TOXGO Uncharacterized protein 4600 3143-3148
124. VPSLVS V4Z6H5 V4Z6H5_TOXGO Putative glutamic acid-rcih protein 4436 2668-2673
125. VPSLVS V4ZER8 V4ZER8_TOXGO Uncharacterized protein 4402 2287-2292
126. VPSLVS V4ZJA8 V4ZJA8_TOXGO Uncharacterized protein 145 6-11
127. VPSLVS V4ZNF6 V4ZNF6_TOXGO Putative glutamic acid-rich protein 2698 121-126
128. PSLVSG B9QGW3 B9QGW3_TOXGO Patched family protein 1933 1544-1549
129. PSLVSG V4Z880 V4Z880_TOXGO Uncharacterized protein 4668 4077-4082
130. PSLVSG V4Z8S4 V4Z8S4_TOXGO AP2 domain transcription factor AP2III-4 1645 300-305
131. PSLVSG V4ZBA9 V4ZBA9_TOXGO Putative transmembrane protein 684 459-464
132. PSLVSG V4ZC85 V4ZC85_TOXGO Phosphohistidine phosphatase 386 157-162
133. PSLVSG V4ZDF9 V4ZDF9_TOXGO Putative GTP-binding protein engB 1027 400-405
134. PSLVSG V4ZER8 V4ZER8_TOXGO Uncharacterized protein 4402 2288-2293
135. SLVSGN B9QGW3 B9QGW3_TOXGO Patched family protein 1933 1545-1550
136. SLVSGN Q1JT79 Q1JT79_TOXGO Putative uncharacterized protein precursor 1342 156-161
137. SLVSGN V4Z1C2 V4Z1C2_TOXGO Uncharacterized protein 2290 21-26
138. LVSGNT V4Z6D3 V4Z6D3_TOXGO Putative ribonuclease z 1062 980-985
139. SLEARV V4ZMY4 V4ZMY4_TOXGO Uncharacterized protein 1350 682-687
140. LEARVR B6KAF6 B6KAF6_TOXGO AP2 domain transcription factor AP2XI-2 2243 1994-1999
141. LEARVR V4Z7N4 V4Z7N4_TOXGO Uncharacterized protein 2114 293-298
142. LEARVR V5BGS1 V5BGS1_TOXGO Zinc finger (CCCH type) motif-containing protein 3460 2623-2628
143. EARVRD B9Q2S0 B9Q2S0_TOXGO Uncharacterized protein 518 264-269
144. ARVRDG V4YQA3 V4YQA3_TOXGO Serine esterase (DUF676) protein 1565 685-690
145. ARVRDG V4Z303 V4Z303_TOXGO DnaJ domain-containing protein 2010 257-262
146. IGILTT B9Q309 B9Q309_TOXGO Putative transmembrane protein 389 283-288
147. GILTTA B9Q309 B9Q309_TOXGO Putative transmembrane protein 389 284-289
148. GILTTA B9QDN3 B9QDN3_TOXGO ABC1 family protein 1900 533-538
149. LTTAAS B9QF45 B9QF45_TOXGO Putative transmembrane protein 1306 311-316
150. LTTAAS V4ZNI0 V4ZNI0_TOXGO Putative transmembrane protein 6079 5486-5491
151. TTAASS B9PU35 B9PU35_TOXGO Putative transmembrane protein 877 51-56
152. TTAASS B9QPH0 B9QPH0_TOXGO Uncharacterized protein 1128 241-246
153. TTAASS K7WFT3 K7WFT3_TOXGO DHHC16 1047 412-417
154. TTAASS V4YY19 V4YY19_TOXGO Uncharacterized protein 411 301-306
155. TTAASS V4YZ95 V4YZ95_TOXGO Kinesin motor domain-containing protein 2394 1467-1472
156. TTAASS V4ZBW1 V4ZBW1_TOXGO Uncharacterized protein 99 90-95
157. TTAASS V4ZI37 V4ZI37_TOXGO DHHC zinc finger domain-containing protein 1047 412-417
158. TTAASS V4ZNI0 V4ZNI0_TOXGO Putative transmembrane protein 6079 5487-5492
159. TTAASS V5B4D7 V5B4D7_TOXGO Spc97/Spc98 family protein 2870 2089-2094
160. TAASSM V4ZJP8 V4ZJP8_TOXGO Amine-terminal region of chorein, A TM vesicle-mediated sorter 12207 5258-5263
161. AASSML B9PRR9 B9PRR9_TOXGO Uncharacterized protein 282 10-15
162. AASSML V4Z7F3 V4Z7F3_TOXGO TBC domain-containing protein 2116 694-699
163. AASSML V4ZCB0 V4ZCB0_TOXGO Histone lysine-specific demethylase 3802 838-843
164. MLEKFS B6KGA7 B6KGA7_TOXGO Putative transmembrane protein 349 269-274
165. GKDLSF V4ZES6 V4ZES6_TOXGO Transporter, small conductance mechanosensitive ion channel (MscS) family protein 3400 1810-1815
166. SFTEEG B9QND0 B9QND0_TOXGO ENTH domain-containing protein 634 132-137
167. SFTEEG V5BE69 V5BE69_TOXGO Putative ATP-dependent hsl protease ATP-binding subunit hslU 938 665-670
168. QVHPRL V5BGS1 V5BGS1_TOXGO Zinc finger (CCCH type) motif-containing protein 3460 3391-3396
169. HTLSLR V5B468 V5B468_TOXGO Uncharacterized protein 2811 2403-2408
170. TLSLRH V4Z1S4 V4Z1S4_TOXGO HEAT repeat-containing protein 1450 354-359
171. TLSLRH V4ZR02 V4ZR02_TOXGO SAM-dependent methyltransferase 760 184-189
172. LSLRHA V4ZBR9 V4ZBR9_TOXGO Protein kinase 1763 13-18
173. VTLEEA V4ZEZ5 V4ZEZ5_TOXGO AMP-binding enzyme 1075 217-222
174. TLEEAP B6KN45 B6KN45_TOXGO Elongation factor 1-alpha 448 372-377
175. EEAPFV B9QFF8 B9QFF8_TOXGO PHD-finger domain-containing protein 2138 286-291
176. TETCVR V4YMY1 V4YMY1_TOXGO Tetratricopeptide repeat-containing protein 1008 119-124
177. TVPCRK V4ZC24 V4ZC24_TOXGO Uncharacterized protein 817 445-450
178. RKFVKI V4ZBS5 V4ZBS5_TOXGO RAP domain-containing protein 2755 1647-1652
179. IDILKK V4ZHN6 V4ZHN6_TOXGO Flagellar associated protein 222 154-159
180. IDILKK V5BCB9 V5BCB9_TOXGO Putative autoantigen, coiled-coil vesicle tethering subfamily A protein 1 431 395-400
181. LKKLSR B9PZ51 B9PZ51_TOXGO Toxoplasma gondii family B protein 150 61-66
182. KKLSRT B9PZ51 B9PZ51_TOXGO Toxoplasma gondii family B protein 150 62-67
183. LSRTVK B9Q7W6 B9Q7W6_TOXGO Putative F-box protein 1461 186-191
184. LVTNGK V4ZFK1 V4ZFK1_TOXGO Erythronate-4-phosphate dehydrogenase domain-containing protein 382 146-151
185. RAVMAV B6KB67 B6KB67_TOXGO Rhoptry kinase family protein ROP28 694 213-218
186. MAVGSL V4YRC2 V4YRC2_TOXGO Putative transmembrane protein 4703 3052-3057
187. AVGSLT B9QFN5 B9QFN5_TOXGO Dip2/Utp12 family protein 774 658-663
188. SLTINE V4YWD3 V4YWD3_TOXGO Putative transmembrane protein 1502 1404-1409
189. LTINEE V5AXU2 V5AXU2_TOXGO Uncharacterized protein 929 910-915
190. EERSEV B9QIK5 B9QIK5_TOXGO Uncharacterized protein 1006 880-885
191. VVDFSV V4ZH83 V4ZH83_TOXGO Ulp1 protease family, C-terminal catalytic domain-containing protein 1760 172-177
192. VDFSVP V4ZTC0 V4ZTC0_TOXGO Queuine trna-ribosyltransferase domain-containing protein 747 23-28
193. DFSVPF V4YXD3 V4YXD3_TOXGO Glycosyltransferase, group 1 family protein 462 278-283
194. FSVPFV A2AXH5 A2AXH5_TOXGO Pdx1 protein 307 130-135
195. FSVPFV V4YXD3 V4YXD3_TOXGO Glycosyltransferase, group 1 family protein 462 279-284
196. FSVPFV V5B5T4 V5B5T4_TOXGO Putative ethylene inducible protein 307 130-135
197. PFVETG V4ZFC8 V4ZFC8_TOXGO Leucyl aminopeptidase LAP 781 60-65
198. NGTVSP V4ZWK4 V4ZWK4_TOXGO Myosin-light-chain kinase 1794 1341-1346
199. GTVSPS B6K9Q1 B6K9Q1_TOXGO Uncharacterized protein 655 365-370
200. GTVSPS V4YNZ5 V4YNZ5_TOXGO Putative glycogen synthase 3004 2460-2465
201. GTVSPS V4ZAK3 V4ZAK3_TOXGO Uncharacterized protein 3378 1993-1998
202. GTVSPS V4ZWK4 V4ZWK4_TOXGO Myosin-light-chain kinase 1794 1342-1347
203. TVSPSA M9T1W1 M9T1W1_TOXGO ATG4 3753 1317-1322
204. TVSPSA V4Z7Q1 V4Z7Q1_TOXGO Zinc finger domain, LSD1 subclass domain-containing protein 1059 30-35
205. TVSPSA V4ZBG2 V4ZBG2_TOXGO 3'5'-cyclic nucleotide phosphodiesterase domain-containing protein 3476 579-584
206. TVSPSA V4ZWU1 V4ZWU1_TOXGO Uncharacterized protein 1719 201-206
207. TVSPSA V5B196 V5B196_TOXGO Uncharacterized protein 2539 1545-1550
208. VSPSAF V4ZBG2 V4ZBG2_TOXGO 3'5'-cyclic nucleotide phosphodiesterase domain-containing protein 3476 580-585
209. VSPSAF V4ZES6 V4ZES6_TOXGO Transporter, small conductance mechanosensitive ion channel (MscS) family protein 3400 822-827
210. VSPSAF V4ZIG9 V4ZIG9_TOXGO Putative transmembrane protein 1334 1004-1009
211. VSPSAF V5BBD9 V5BBD9_TOXGO Elongation factor Tu GTP binding domain-containing protein 2366 2047-2052
212. SPSAFL B9QCQ8 B9QCQ8_TOXGO AP2 domain transcription factor AP2XII-8 1679 1296-1301
213. SPSAFL B9QCW2 B9QCW2_TOXGO Uncharacterized protein 754 416-421
214. SPSAFL V4YJB7 V4YJB7_TOXGO Protein kinase domain-containing protein 1290 989-994
215. SPSAFL V4YKA0 V4YKA0_TOXGO Uncharacterized protein 559 67-72
216. SPSAFL V4ZD37 V4ZD37_TOXGO eIF2 kinase IF2K-C 3270 450-455
217. SPSAFL V4ZHE1 V4ZHE1_TOXGO ATG C terminal domain-containing protein 8079 1584-1589
218. SPSAFL V4ZJ80 V4ZJ80_TOXGO Amine-terminal region of chorein, A TM vesicle-mediated sorter 5383 661-666
219. SPSAFL V4ZMC8 V4ZMC8_TOXGO Dual specificity phosphatase, catalytic domain-containing protein 812 613-618
220. SPSAFL V5BIW0 V5BIW0_TOXGO Uncharacterized protein 858 357-362
221. SPSAFL X2F4W3 X2F4W3_TOXGO GCN2-like eIF2 alpha kinase 3269 450-455
222. PSAFLE B6KRY2 B6KRY2_TOXGO Uncharacterized protein 1745 791-796
223. PSAFLE V4YJB7 V4YJB7_TOXGO Protein kinase domain-containing protein 1290 990-995
224. SAFLEP B6KRY2 B6KRY2_TOXGO Uncharacterized protein 1745 792-797
225. SAFLEP V4ZLU8 V4ZLU8_TOXGO SWI2/SNF2-containing protein 3776 1100-1105
226. PFSASV B9Q4U1 B9Q4U1_TOXGO Amine-terminal region of chorein, A TM vesicle-mediated sorter 10329 1318-1323
227. PFSASV V4ZEL7 V4ZEL7_TOXGO CPSF A subunit region protein 2847 150-155
228. PFSASV V5BBQ8 V5BBQ8_TOXGO Leucine rich repeat-containing protein 4458 2014-2019, 2032-2037
229. FSPVGY V4ZJP8 V4ZJP8_TOXGO Amine-terminal region of chorein, A TM vesicle-mediated sorter 12207 9657-9662
230. VGYNRN V5B3Z0 V5B3Z0_TOXGO FATC domain-containing protein 6012 4592-4597
231. GKAPHG V5BLE9 V5BLE9_TOXGO AP2 domain transcription factor AP2VIII-4 3417 2570-2575
232. HGPSFT V4YIJ9 V4YIJ9_TOXGO Uncharacterized protein 221 138-143
233. AIWLLW Q8MUM2 Q8MUM2_TOXGO Facilitative glucose transporter 568 233-238
234. LLWGLV B9QG55 B9QG55_TOXGO Putative kynurenine 3-monooxygenase and-related flavoprotein monooxygenase family (ISS) protein 1761 735-740
235. AVIFLA V4Z520 V4Z520_TOXGO Cytochrome p450 superfamily protein 553 509-514
236. VIFLAS B9Q0Y3 B9Q0Y3_TOXGO Uncharacterized protein 170 144-149
237. LASYTA V4ZGD0 V4ZGD0_TOXGO Citrate synthase 509 137-142
238. MIQEEF V4ZP41 V4ZP41_TOXGO Uncharacterized protein 625 424-429
239. VDQVTG V5BAM1 V5BAM1_TOXGO Uncharacterized protein 957 248-253
240. QVTGLS V4ZBR9 V4ZBR9_TOXGO Protein kinase 1763 1356-1361
241. YSPPFR V4ZWZ5 V4ZWZ5_TOXGO 'chromo' (CHRromatin Organization MOdifier) domain-containing protein 1408 526-531
242. SPPFRF B9QPP3 B9QPP3_TOXGO HECT-domain (Ubiquitin-transferase) domain protein 389 148-153
243. PPFRFG V4ZB34 V4ZB34_TOXGO Putative transmembrane protein 142 76-81
244. VPNGST B9QEL6 B9QEL6_TOXGO Rhoptry kinase family protein ROP41 490 356-361
245. PNGSTE B9QEL6 B9QEL6_TOXGO Rhoptry kinase family protein ROP41 490 357-362
246. NGSTER V4ZF03 V4ZF03_TOXGO Dynein, axonemal, heavy chain 2 family protein 4547 23-28
247. KGVEDA B6K9A1 B6K9A1_TOXGO Bradyzoite rhoptry protein BRP1 162 71-76
248. KGVEDA V4Z445 V4Z445_TOXGO Histone acetyltransferase TAF1/250 2775 2318-2323
249. KGVEDA V4ZIB5 V4ZIB5_TOXGO Uncharacterized protein 2104 795-800
250. GVEDAL V4Z445 V4Z445_TOXGO Histone acetyltransferase TAF1/250 2775 2319-2324
251. VEDALV V4ZB09 V4ZB09_TOXGO Putative hypoxia-inducible factor prolyl hydroxylase (Phd2) 577 118-123
252. ALVSLK B6KLP1 RON22_TOXGO Rhoptry neck protein 2-like protein 2 precursor 1167 919-924
253. ALVSLK V4ZEK4 V4ZEK4_TOXGO Putative rhoptry neck protein 1167 919-924
254. LKTGKL V4YVE3 V4YVE3_TOXGO RNB family domain-containing protein 1150 339-344
255. FIYDAA B9Q7I5 B9Q7I5_TOXGO Putative aminopeptidase n 970 532-537
256. YDAAVL V5BMD9 V5BMD9_TOXGO UBA/TS-N domain-containing protein 7817 38-43
257. AGRDEG B9PY95 B9PY95_TOXGO Arginine N-methyltransferase, putative 660 633-638
258. AGRDEG Q2VTP8 Q2VTP8_TOXGO Protein arginine methyltransferase 441 414-419
259. AGRDEG V4Z754 V4Z754_TOXGO AAA domain protein 2605 2126-2131
260. AGRDEG V4ZFN7 V4ZFN7_TOXGO Uncharacterized protein 676 381-386
261. FATTGY B9PTL3 B9PTL3_TOXGO Uncharacterized protein 610 597-602
262. GSPWKR V4YZ84 V4YZ84_TOXGO Putative divalent metal transporter 1232 203-208
263. SPWKRQ V4YZ84 V4YZ84_TOXGO Putative divalent metal transporter 1232 204-209
264. DLALLQ B9Q853 B9Q853_TOXGO Trypsin-like peptidase domain protein 992 610-615
265. ALLQFV B6K9Y2 B6K9Y2_TOXGO Transcription elongation factor SPT6 3132 2266-2271
266. LLQFVG V4ZBS8 V4ZBS8_TOXGO ABC transporter transmembrane region domain-containing protein 1951 397-402
267. GDGEME V4ZJP8 V4ZJP8_TOXGO Amine-terminal region of chorein, A TM vesicle-mediated sorter 12207 5571-5576
268. DGEMEE V4YUW7 V4YUW7_TOXGO Uncharacterized protein 622 281-286
269. EELETL V4ZBN2 V4ZBN2_TOXGO Patched family protein 2498 2123-2128
270. EELETL V4ZNI0 V4ZNI0_TOXGO Putative transmembrane protein 6079 4173-4178
271. EELETL V4ZVJ1 V4ZVJ1_TOXGO Putative dynein heavy chain 2299 864-869
272. FYMLAA V5B8W0 V5B8W0_TOXGO Folate/biopterin transporter subfamily protein 1057 107-112
273. MLAAAM B9QH00 B9QH00_TOXGO Putative RNA methyltransferase 833 203-208
274. LAAAMA B9QFB6 B9QFB6_TOXGO PGAP1 family protein 2110 1924-1929
275. LAAAMA B9QH00 B9QH00_TOXGO Putative RNA methyltransferase 833 204-209
276. LAAAMA V4ZKI0 V4ZKI0_TOXGO Uncharacterized protein 964 334-339
277. AAMALS V4ZA14 V4ZA14_TOXGO Intraflagellar transport 80 family protein 442 287-292
278. ALSLIT Q9BJM5 Q9BJM5_TOXGO Acetyl-CoA carboxylase 2 723 696-701
279. ALSLIT V4YZX5 V4YZX5_TOXGO Acetyl-coA carboxylase ACC2 3400 3373-3378
280. FTGVCS B9Q3U4 B9Q3U4_TOXGO Putative transmembrane protein 217 176-181
281. TGVCSD B9Q843 B9Q843_TOXGO Histone lysine methyltransferase SET1 7555 808-813
282. DRPGLL V4YNS8 V4YNS8_TOXGO Uncharacterized protein 3987 3833-3838
283. LLFSIS V4ZP18 V4ZP18_TOXGO Putative transmembrane protein 2590 2184-2189
284. IEEKKK B9Q8G8 B9Q8G8_TOXGO Uncharacterized protein 484 325-330
285. EEKKKS B6KGA9 B6KGA9_TOXGO Uncharacterized protein 1548 1187-1192
286. EEKKKS B9QNW5 B9QNW5_TOXGO Putative trichohyalin 394 276-281
287. EEKKKS K7WFS9 K7WFS9_TOXGO DHHC11 944 646-651
288. EEKKKS V4Z994 V4Z994_TOXGO DHHC zinc finger domain-containing protein 951 653-658
289. DFNLTG B9Q595 B9Q595_TOXGO Ck2 beta subunit 315 84-89
290. LTGSQS B6KNV6 B6KNV6_TOXGO C3orf15 protein 586 303-308
291. LTGSQS V4YZE7 V4YZE7_TOXGO Uncharacterized protein 1048 125-130
292. LKLLRS B9QI06 B9QI06_TOXGO SART-1 family protein 861 584-589
293. KLLRSA V4Z0A1 V4Z0A1_TOXGO Uncharacterized protein 607 537-542
294. LRSAKN B9QMU8 B9QMU8_TOXGO Putative transmembrane protein 2220 966-971
295. RSAKNI V4Z3T3 V4Z3T3_TOXGO Intraflagellar transport 52 (Protein NGD5 ) family protein 519 480-485
296. RAADFI B9PMC6 B9PMC6_TOXGO Putative transmembrane protein 691 82-87
297. FIQRGS B6KAN6 B6KAN6_TOXGO Uncharacterized protein 278 260-265
298. FIQRGS V4Z8G0 V4Z8G0_TOXGO Guanylyl cyclase 4367 330-335
299. SDNRSF V4ZH67 V4ZH67_TOXGO Uncharacterized protein 3076 1999-2004
300. MNELQT V5BE19 V5BE19_TOXGO Uncharacterized protein 1214 236-241
301. VAVSTE B9QHR3 B9QHR3_TOXGO Putative armadillo/beta-catenin-like repeat protein 1297 480-485
302. AVSTES B9PUC7 B9PUC7_TOXGO Metallo-beta-lactamase domain-containing protein 798 173-178
303. STESKA Q1JTA6 Q1JTA6_TOXGO Putative uncharacterized protein 304 28-33
304. STESKA V5B047 V5B047_TOXGO Putative transmembrane protein 345 69-74
305. SKANSR B9QE42 B9QE42_TOXGO Putative proteophosphoglycan protein ppg4 1290 716-721
306. SKANSR B9QEX3 B9QEX3_TOXGO Bromodomain-containing protein 1354 291-296
307. SRPRQL B9QB70 B9QB70_TOXGO Helicase associated domain (Ha2) protein 1277 121-126
308. RPRQLW V4Z3C9 V4Z3C9_TOXGO Uncharacterized protein 1899 446-451
309. WKKSVD B6KQU6 B6KQU6_TOXGO Cytoadherence-linked asexual protein 1490 1166-1171
310. WKKSVD Q1JTB2 Q1JTB2_TOXGO Putative uncharacterized protein precursor 1453 1129-1134
311. SIRQDS V5BDM3 V5BDM3_TOXGO Sec7 domain-containing protein 3987 2730-2735
312. RQDSLS Q1JSF7 Q1JSF7_TOXGO Uncharacterized protein 928 84-89
313. RQDSLS V4Z2A3 V4Z2A3_TOXGO RNA recognition motif-containing protein 667 479-484
314. SQRDEA V4ZKR1 V4ZKR1_TOXGO Aminotransferase class IV 687 229-234
315. RDEATA V4ZC84 V4ZC84_TOXGO Kinesin motor domain-containing protein 1249 1119-1124
316. DEATAE B6KHD9 B6KHD9_TOXGO Uncharacterized protein 271 108-113
317. ATAENR V5BB80 V5BB80_TOXGO Uncharacterized protein 200 11-16
318. THSLKS V4ZMT1 V4ZMT1_TOXGO Uncharacterized protein 1015 347-352
319. RYLPEE B6KGI0 B6KGI0_TOXGO Uncharacterized protein 1265 494-499
320. RYLPEE V4ZLI6 V4ZLI6_TOXGO N-acetylgalactosaminyl transferase 865 690-695
321. DISETS B9QP43 B9QP43_TOXGO Putative NAD-dependent epimerase/dehydratase 836 110-115
322. ETSNRA V4ZCJ9 V4ZCJ9_TOXGO Ankyrin repeat-containing protein 836 517-522
323. ETSNRA V5B468 V5B468_TOXGO Uncharacterized protein 2811 1197-1202
324. SKNHKT V4ZCL8 V4ZCL8_TOXGO Uncharacterized protein 785 585-590
325. KRSVAS V4Z9V6 V4Z9V6_TOXGO Uncharacterized protein 1571 40-45
326. RSVASK V4YPS5 V4YPS5_TOXGO Uncharacterized protein 1776 1476-1481
327. SVASKY V4ZI01 V4ZI01_TOXGO Uncharacterized protein 532 59-64
328. CSEVER V5BN33 V5BN33_TOXGO WD domain, G-beta repeat-containing protein 948 385-390
329. SEVERT V5BN33 V5BN33_TOXGO WD domain, G-beta repeat-containing protein 948 386-391
330. EVERTY V4Z0C3 V4Z0C3_TOXGO Myb family DNA-binding domain-containing protein 1913 1350-1355
331. RTYLKT V4ZVK3 V4ZVK3_TOXGO Uncharacterized protein 2214 1704-1709
332. KSSSPR V4Z6H5 V4Z6H5_TOXGO Putative glutamic acid-rcih protein 4436 1004-1009
333. SSSPRD B6KSW5 B6KSW5_TOXGO Zinc finger, C3HC4 type (RING finger) domain-containing protein 1027 692-697
334. SSSPRD B9QB36 B9QB36_TOXGO Acetyltransferase, GNAT family protein 757 192-197
335. SSSPRD Q86PI1 Q86PI1_TOXGO Jlp2 550 165-170 139750
336. SSSPRD V4YJ14 V4YJ14_TOXGO Uncharacterized protein 1223 1060-1065
337. SSSPRD V4Z7A0 V4Z7A0_TOXGO Dense granule protein GRA15 635 166-171
338. SSSPRD V4Z8G0 V4Z8G0_TOXGO Guanylyl cyclase 4367 1038-1043
339. SSSPRD V4ZBB6 V4ZBB6_TOXGO Uncharacterized protein 3325 719-724
340. SSSPRD V4ZW70 V4ZW70_TOXGO Uncharacterized protein 5655 4536-4541
341. TIDGEK V4ZDT2 V4ZDT2_TOXGO Putative transmembrane protein 930 534-539
342. DGEKEP B9QL14 B9QL14_TOXGO Inositol polyphosphate kinase 2851 2730-2735
343. DGEKEP Q1JSS7 Q1JSS7_TOXGO Inositol hexaphosphate kinase 3, putative 1842 1721-1726
344. GEKEPG V4Z409 V4Z409_TOXGO HECT-domain (Ubiquitin-transferase) domain-containing protein 1709 916-921
345. GEKEPG V4ZEU4 V4ZEU4_TOXGO Putative transmembrane protein 1002 367-372
346. EPGFHL B9QKB8 B9QKB8_TOXGO Uncharacterized protein 2225 1391-1396
347. PYQDPS V4Z5J3 V4Z5J3_TOXGO Uncharacterized protein 149 75-80
348. YQDPSE V4Z5J3 V4Z5J3_TOXGO Uncharacterized protein 149 76-81
349. RKGDST V4ZIJ3 V4ZIJ3_TOXGO Ulp1 protease family, C-terminal catalytic domain-containing protein 3028 2713-2718
350. RKGDST V4ZJH9 V4ZJH9_TOXGO NIMA-related protein kinase NIMA1 1609 1422-1427
351. GDSTLP V4ZFK6 V4ZFK6_TOXGO ER-trafficking TRAPP I complex 85 kDa subunit 3377 1299-1304
352. GDSTLP V4ZIU9 V4ZIU9_TOXGO Uncharacterized protein 1263 1159-1164
353. GDSTLP V4ZJ80 V4ZJ80_TOXGO Amine-terminal region of chorein, A TM vesicle-mediated sorter 5383 4358-4363
354. STLPMN B9Q616 B9Q616_TOXGO Uncharacterized protein 460 109-114
355. NPLHNE B9PYV2 B9PYV2_TOXGO Importin-beta N-terminal domain-containing protein 1147 486-491
356. PLHNEE V4YYX8 V4YYX8_TOXGO EF-hand protein 1299 531-536
357. LHNEEG V4YYX8 V4YYX8_TOXGO EF-hand protein 1299 532-537
358. HNEEGL V4YYX8 V4YYX8_TOXGO EF-hand protein 1299 533-538
359. NNDQYK Q9BKE2 Q9BKE2_TOXGO Glyceraldehyde-3-phosphate dehydrogenase 340 136-141
360. NNDQYK V5BE77 V5BE77_TOXGO Glyceraldehyde-3-phosphate dehydrogenase 489 285-290
361. LKDKGS B6KRG1 B6KRG1_TOXGO Putative late embryogenesis abundant domain protein 517 215-220
362. GSPHSE V4ZEZ0 V4ZEZ0_TOXGO Histone lysine demethylase JMJD4 1662 970-975
363. PHSETS V4ZF91 V4ZF91_TOXGO SWI2/SNF2-containing protein 2045 1565-1570
364. SETSER Q1JT85 Q1JT85_TOXGO Putative uncharacterized protein 663 436-441
365. SETSER V4YLR5 V4YLR5_TOXGO YrdC domain-containing protein 828 575-580
366. CRSCLS B9QJX3 B9QJX3_TOXGO Uncharacterized protein 4533 3460-3465
367. RSCLSN V4Z8M6 V4Z8M6_TOXGO Putative 30S ribosomal protein S12 594 53-58
368. LQETGN B9Q7R9 B9Q7R9_TOXGO Uncharacterized protein 504 265-270
369. ETGNPA V4Z5A6 V4Z5A6_TOXGO Uncharacterized protein 1868 1403-1408
370. TGNPAT V4Z5A6 V4Z5A6_TOXGO Uncharacterized protein 1868 1404-1409
371. GNPATG B9PFU2 B9PFU2_TOXGO Phosphoglycerate mutase family protein 307 90-95
372. NPATGE B9PFU2 B9PFU2_TOXGO Phosphoglycerate mutase family protein 307 91-96
373. PATGEQ B9QIQ4 B9QIQ4_TOXGO Uncharacterized protein 615 173-178
374. NNALQL B9QJ77 B9QJ77_TOXGO Uncharacterized protein 2002 603-608
375. LQLQKN V4ZLF7 V4ZLF7_TOXGO Putative O-acetylserine (Thiol) lyase 2 585 79-84
376. VDKPRE V4YXU2 V4YXU2_TOXGO Dynein heavy chain family protein 4551 1725-1730
377. KPRELD B6KJ47 B6KJ47_TOXGO Uncharacterized protein 3460 3035-3040
378. RELDLS V4Z8N7 V4Z8N7_TOXGO Uncharacterized protein 3607 409-414
379. LDLSRP B9PY06 B9PY06_TOXGO Endonuclease/exonuclease/phosphatase family protein 682 550-555
380. DLSRPS B9Q8D8 B9Q8D8_TOXGO Chloroquine resistance marker 3946 714-719
381. LSRPSR B9Q792 B9Q792_TOXGO Uncharacterized protein 4118 1923-1928
382. LSRPSR V5AWW8 V5AWW8_TOXGO Putative transmembrane protein 4107 3775-3780
383. SRPSRS B9PSR7 B9PSR7_TOXGO Uncharacterized protein 417 256-261
384. SRPSRS V4YR36 V4YR36_TOXGO Uncharacterized protein 562 401-406
385. SRPSRS V4YXQ4 V4YXQ4_TOXGO Ubiquitin carboxyl-terminal hydrolase 3144 2336-2341
386. SRPSRS V4Z3T3 V4Z3T3_TOXGO Intraflagellar transport 52 (Protein NGD5 ) family protein 519 26-31
387. SRPSRS V4Z9U0 V4Z9U0_TOXGO Uncharacterized protein 5083 1180-1185
388. PSRSIS M9T1W1 M9T1W1_TOXGO ATG4 3753 16-21
389. PSRSIS V4ZM71 V4ZM71_TOXGO Putative autophagy-related cysteine peptidase atg4 3747 16-21
390. SRSISL V4Z819 V4Z819_TOXGO DEAD/DEAH box helicase domain-containing protein 1850 640-645
391. SRSISL V4ZWU6 V4ZWU6_TOXGO Leucine rich repeat-containing protein 1024 967-972
392. RSISLK V4Z3T5 V4Z3T5_TOXGO Putative LMBR1 family region protein 656 151-156
393. ISLKDR B6KHU2 B6KHU2_TOXGO Putative subunit of proteaseome activator complex 269 45-50
394. LKDRER V4ZDB4 V4ZDB4_TOXGO Uncharacterized protein 1981 157-162
395. DRERLL Q9NGR7 Q9NGR7_TOXGO Proliferating cell nuclear antigen 316 63-68
396. DRERLL V4ZDF1 V4ZDF1_TOXGO Uncharacterized protein 1485 413-418
397. DRERLL V5B9N6 V5B9N6_TOXGO Nucleolar GTP-binding protein 1 922 865-870
398. RERLLE B9Q0I8 B9Q0I8_TOXGO RecF/RecN/SMC N terminal domain-containing protein 1418 942-947
399. RERLLE Q1JSI5 Q1JSI5_TOXGO Uncharacterized protein 3127 2351-2356
400. RERLLE V4YL69 V4YL69_TOXGO Rft protein 1197 533-538
401. RERLLE V4Z515 V4Z515_TOXGO ERCC4 domain-containing protein 2053 192-197
402. RERLLE V4Z6D7 V4Z6D7_TOXGO Uncharacterized protein 2936 2160-2165
403. RERLLE V5BMJ1 V5BMJ1_TOXGO 'chromo' (CHRromatin Organization MOdifier) domain-containing protein 1808 506-511
404. ERLLEG Q1JSI5 Q1JSI5_TOXGO Uncharacterized protein 3127 2352-2357
405. ERLLEG V4Z6D7 V4Z6D7_TOXGO Uncharacterized protein 2936 2161-2166
406. ERLLEG V4ZEH7 V4ZEH7_TOXGO Uncharacterized protein 1252 716-721
407. ERLLEG V5B8F2 V5B8F2_TOXGO Peptidase family c50 protein 6890 1848-1853
408. NFYGSL V4ZQ64 V4ZQ64_TOXGO Zinc finger, C3HC4 type (RING finger) domain-containing protein 794 601-606
409. GSLFSV B9PYD7 B9PYD7_TOXGO Uncharacterized protein 173 74-79
410. GSLFSV V5AXG5 V5AXG5_TOXGO FYVE zinc finger domain-containing protein 518 146-151
411. SLFSVP Q1JTH5 Q1JTH5_TOXGO Putative uncharacterized protein precursor 2837 2296-2301
412. SLFSVP V5AY44 V5AY44_TOXGO Putative transmembrane protein 7450 4436-4441
413. SLFSVP V5AZM0 V5AZM0_TOXGO Cyclic nucleotide-binding domain-containing protein 3062 2521-2526
414. LFSVPS V4ZK17 V4ZK17_TOXGO Uncharacterized protein 2660 499-504
415. LFSVPS V5BE34 V5BE34_TOXGO Uncharacterized protein 1239 155-160
416. FSVPSS B9QHR3 B9QHR3_TOXGO Putative armadillo/beta-catenin-like repeat protein 1297 101-106
417. FSVPSS B9QR17 B9QR17_TOXGO Uncharacterized protein 6038 2659-2664
418. FSVPSS V4Z1H5 V4Z1H5_TOXGO Ribosomal protein RPS27 151 47-52
419. FSVPSS V4Z5P5 V4Z5P5_TOXGO Uncharacterized protein 5771 3506-3511
420. FSVPSS V4ZKD8 V4ZKD8_TOXGO Uncharacterized protein 11926 1184-1189
421. SVPSSK V5BBF3 V5BBF3_TOXGO Putative transmembrane protein 1927 1353-1358
422. PSSKLS Q1JSC8 Q1JSC8_TOXGO Uncharacterized protein 734 30-35
423. PSSKLS V4ZB21 V4ZB21_TOXGO Uncharacterized protein 741 30-35
424. PSSKLS V4ZQP4 V4ZQP4_TOXGO PAN domain-containing protein 1966 1634-1639
425. PSSKLS V5BBS6 V5BBS6_TOXGO Ribosome biogenesis GTPase Der protein 1125 166-171
426. SSKLSG V4ZI10 V4ZI10_TOXGO Uncharacterized protein 767 309-314
427. LSGKKS V4Z3B0 V4Z3B0_TOXGO Uncharacterized protein 271 31-36
428. SGKKSS B6KAP3 B6KAP3_TOXGO Uncharacterized protein 305 141-146
429. SGKKSS V4Z0X2 V4Z0X2_TOXGO Chloride transporter, chloride channel (ClC) family protein 1597 162-167
430. SGKKSS V4ZJZ9 V4ZJZ9_TOXGO ATPase, AAA family protein 3910 3713-3718
431. KKSSLF Q9MTD7 Q9MTD7_TOXGO Clp 765 345-350
432. SLFPQG V4ZJW0 V4ZJW0_TOXGO Exostosin family protein 1327 345-350
433. LFPQGL V4YW89 V4YW89_TOXGO Sushi domain (Scr repeat) domain-containing protein 4752 1603-1608
434. QGLEDS V4YQJ6 V4YQJ6_TOXGO Uncharacterized protein 2197 697-702
435. GLEDSK V5BDM3 V5BDM3_TOXGO Sec7 domain-containing protein 3987 2582-2587
436. SKRSKS B6KP82 B6KP82_TOXGO SAG-related sequence SRS11 190 91-96
437. SKRSKS Q9XYH0 Q9XYH0_TOXGO SAG2 related antigen SAG2B 190 91-96
438. SKRSKS R4JAM5 R4JAM5_TOXGO Surface antigen 2B 130 31-36
439. RSKSLL B9QGP5 B9QGP5_TOXGO Serine/threonine-protein phosphatase 2A activator 561 274-279
440. RSKSLL V4Z9J2 V4Z9J2_TOXGO Uncharacterized protein 1923 1248-1253
441. SKSLLP V4Z9J2 V4Z9J2_TOXGO Uncharacterized protein 1923 1249-1254
442. SKSLLP V5BI89 V5BI89_TOXGO Dullard family phosphatase domain-containing protein 644 269-274
443. FLHSHR V4Z5W3 V4Z5W3_TOXGO OTU family cysteine protease 988 836-841
444. RLVIGR Q45W18 Q45W18_TOXGO ATP-binding cassette, sub-family G, member 1 794 295-300
445. RLVIGR V4Z8I8 V4Z8I8_TOXGO ATP-binding cassette G family transporter ABCG87 794 295-300
446. RLVIGR V4ZKD4 V4ZKD4_TOXGO PPIC-type PPIASE domain-containing protein 914 213-218
447. PSDPYK V4YX08 V4YX08_TOXGO Sec23/Sec24 trunk domain-containing protein 1540 152-157
448. YKHSLP V4Z1Z1 V4Z1Z1_TOXGO FATC domain-containing protein 2244 1364-1369
449. KHSLPS V4Z1Z1 V4Z1Z1_TOXGO FATC domain-containing protein 2244 1365-1370
450. SLPSQA B9QFP6 B9QFP6_TOXGO Rad17 cell cycle checkpoint protein 1867 1145-1150
451. SLPSQA Q1JSU6 Q1JSU6_TOXGO Transcription factor IIIb subunit, putative 644 269-274
452. SLPSQA V4YM08 V4YM08_TOXGO Transcription initiation factor TFIIIB 813 407-412
453. SLPSQA V4YXY1 V4YXY1_TOXGO Uncharacterized protein 1476 1323-1328
454. SLPSQA V4Z3J2 V4Z3J2_TOXGO Uncharacterized protein 623 512-517
455. SLPSQA V4ZEZ2 V4ZEZ2_TOXGO Fe-S protein assembly co-chaperone HscB protein 466 64-69
456. LRSSLR Q1JSM5 Q1JSM5_TOXGO Ubiquitin-transferase, putative precursor 12269 2781-2786
457. LRSSLR V4Z1Z4 V4Z1Z4_TOXGO HECT-domain (Ubiquitin-transferase) domain-containing protein 12299 2837-2842
458. LRSSLR V4Z585 V4Z585_TOXGO ThiF family protein 2933 333-338
459. LRSSLR V4Z5U2 V4Z5U2_TOXGO TBC domain-containing protein 2711 1126-1131
460. LRSSLR V4ZAY9 V4ZAY9_TOXGO Uncharacterized protein 2286 45-50
461. LRSSLR V4ZTH9 V4ZTH9_TOXGO Putative transmembrane protein 1278 245-250
462. LRSSLR V5B6X5 V5B6X5_TOXGO Ubiquitin family protein 607 474-479
463. RSSLRS B6KRA7 B6KRA7_TOXGO ATP-dependent DNA helicase, RecQ family protein 1626 53-58
464. RSSLRS B9QAD0 B9QAD0_TOXGO tRNA methyl transferase 1596 314-319
465. RSSLRS B9QB75 B9QB75_TOXGO Tetratricopeptide repeat-containing protein 3326 364-369
466. RSSLRS Q1JSM5 Q1JSM5_TOXGO Ubiquitin-transferase, putative precursor 12269 2782-2787
467. RSSLRS V4Z1Z4 V4Z1Z4_TOXGO HECT-domain (Ubiquitin-transferase) domain-containing protein 12299 2838-2843
468. RSSLRS V4Z7M9 V4Z7M9_TOXGO Uncharacterized protein 2297 289-294
469. RSSLRS V4ZJJ7 V4ZJJ7_TOXGO Zinc finger, C3HC4 type (RING finger) domain-containing protein 1822 1231-1236
470. SSLRST Q1JSZ3 Q1JSZ3_TOXGO Uncharacterized protein 394 294-299
471. SSLRST V4Z2G8 V4Z2G8_TOXGO Uncharacterized protein 394 302-307
472. SSLRST V4ZFY9 V4ZFY9_TOXGO Uncharacterized protein 108 92-97
473. SSLRST V4ZGJ7 V4ZGJ7_TOXGO Uncharacterized protein 2378 1657-1662
474. SSLRST V5AX84 V5AX84_TOXGO Uncharacterized protein 749 302-307
475. SSLRST V5BG16 V5BG16_TOXGO Uncharacterized protein 1767 503-508
476. LRSTAS B9QIU4 B9QIU4_TOXGO Putative transmembrane protein 519 266-271
477. LRSTAS V4YZ80 V4YZ80_TOXGO HECT-domain (Ubiquitin-transferase) domain-containing protein 15897 5605-5610
478. LRSTAS V4ZJ61 V4ZJ61_TOXGO Uncharacterized protein 627 11-16
479. LRSTAS V5BM94 V5BM94_TOXGO Uncharacterized protein 3520 2079-2084
480. SRDSRG B6KRE3 B6KRE3_TOXGO Phosphotransferase enzyme family protein 547 293-298
481. SRDSRG B9PZ67 B9PZ67_TOXGO Ethanolamine kinase 547 293-298
482. SRDSRG B9QB62 B9QB62_TOXGO Metal cation transporter, ZIP family protein 1067 595-600
483. SRDSRG V4Z7B8 V4Z7B8_TOXGO Alpha/beta hydrolase family protein 3457 1042-1047
484. SRDSRG V4ZHQ4 V4ZHQ4_TOXGO Putative isoleucyl-tRNA synthetase 2447 454-459
485. SRDSRG V4ZTS0 V4ZTS0_TOXGO Uncharacterized protein 2211 1719-1724
486. SRDSRG V5B1K2 V5B1K2_TOXGO Uncharacterized protein 1673 910-915
487. SRDSRG V5BDM3 V5BDM3_TOXGO Sec7 domain-containing protein 3987 3072-3077
488. VLNSCS V5B823 V5B823_TOXGO Spc97 / Spc98 family protein 1877 991-996

**NMDA 2B**

1. LVLAVL B6KAG9 B6KAG9_TOXGO U3 small nucleolar RNA-associated protein 10 3738 1730-1735
2. LVLAVL B6KT39 B6KT39_TOXGO EF hand domain-containing protein 3700 1096-1101
3. LVLAVL B9PU87 B9PU87_TOXGO Uncharacterized protein 268 227-232
4. LVLAVL B9Q7M6 B9Q7M6_TOXGO Putative transmembrane protein 1759 1603-1608
5. LVLAVL Q1JTI3 Q1JTI3_TOXGO Ubiquitin-protein ligase 1, putative 8112 106-111
6. LVLAVL V4YYU7 V4YYU7_TOXGO Putative glyoxalase 4813 3004-3009
7. LVLAVL V4Z0S5 V4Z0S5_TOXGO HEAT repeat-containing protein 2505 1426-1431
8. LVLAVL V4Z553 V4Z553_TOXGO HECT-domain (Ubiquitin-transferase) domain-containing protein 8007 106-111
9. VLAVLA B9QH98 B9QH98_TOXGO Uncharacterized protein 1410 985-990
10. VLAVLA V5BJP3 V5BJP3_TOXGO ALG6, ALG8 glycosyltransferase family protein 896 355-360
11. LAVLAV B9Q309 B9Q309_TOXGO Putative transmembrane protein 389 119-124
12. LAVLAV B9QPM1 B9QPM1_TOXGO Q-cell neuroblast polarisation protein 1157 362-367
13. LAVLAV V4Z2U4 V4Z2U4_TOXGO Uncharacterized protein 1386 757-762
14. LAVLAV V4Z8N7 V4Z8N7_TOXGO Uncharacterized protein 3607 1160-1165
15. AVLAVS B6KA99 B6KA99_TOXGO WD domain, G-beta repeat-containing protein 744 312-317
16. AVLAVS B9PLW7 B9PLW7_TOXGO Putative transmembrane protein 470 361-366
17. AVLAVS V4ZBJ0 V4ZBJ0_TOXGO Uncharacterized protein 3206 126-131
18. VLAVSG B9PLW7 B9PLW7_TOXGO Putative transmembrane protein 470 362-367
19. LAVSGS V4YMZ0 V4YMZ0_TOXGO Sushi domain (Scr repeat) domain-containing protein 3916 3232-3237
20. LAVSGS V4ZEU8 V4ZEU8_TOXGO PAN domain-containing protein 1860 1689-1694
21. LAVSGS V4ZQF7 V4ZQF7_TOXGO Transporter, major facilitator family protein 547 449-454
22. AVSGSR B6KAF6 B6KAF6_TOXGO AP2 domain transcription factor AP2XI-2 2243 1426-1431
23. AVSGSR V4ZDM0 V4ZDM0_TOXGO HEAT repeat-containing protein 4132 924-929
24. VSGSRA V4YIS8 V4YIS8_TOXGO Uncharacterized protein 1194 975-980
25. VSGSRA V4Z6K2 V4Z6K2_TOXGO A C2HC-type zinc-finger protein 2752 1320-1325
26. VSGSRA V4ZIG9 V4ZIG9_TOXGO Putative transmembrane protein 1334 76-81
27. SGSRAR V4Z3K3 V4Z3K3_TOXGO PIK3R4 kinase-related protein (Incomplete catalytic triad) 3028 2674-2679
28. GSRARS V4ZBT5 V4ZBT5_TOXGO Uncharacterized protein 356 19-24
29. GSRARS V5B1X7 V5B1X7_TOXGO Glycosyl hydrolases family 35 protein 1876 955-960
30. GSRARS V5B509 V5B509_TOXGO Uncharacterized protein 938 81-86
31. RARSQK V4YMX1 V4YMX1_TOXGO Uncharacterized protein 982 927-932
32. SIGIAV Q1JSV5 Q1JSV5_TOXGO Uncharacterized protein 846 76-81
33. SIGIAV V4ZAB1 V4ZAB1_TOXGO GCC2 and GCC3 domain-containing protein 2914 644-649
34. AVILVG B9PUT8 B9PUT8_TOXGO BT1 family protein 834 545-550
35. VGTSDE B6KAR1 B6KAR1_TOXGO WD domain, G-beta repeat-containing protein 845 548-553
36. GTSDEV B6K9U3 B6K9U3_TOXGO 1,4-alpha-glucan-branching enzyme 972 92-97
37. GTSDEV Q5IXJ1 Q5IXJ1_TOXGO Putative 1,4-alpha-glucan branching enzyme 1 983 92-97
38. TSDEVA V4Z5T1 V4Z5T1_TOXGO Uncharacterized protein 1825 519-524
39. SDEVAI B3VQI5 B3VQI5_TOXGO Micronemal protein 16 668 114-119
40. SDEVAI B9QE22 B9QE22_TOXGO Microneme protein MIC16 668 114-119
41. DEVAIK B9PPN2 B9PPN2_TOXGO Casein kinase I 324 33-38
42. DEVAIK Q6QNM1 KC1_TOXGO Casein kinase I 324 33-38
43. AHEKDD B6K8J1 B6K8J1_TOXGO Putative transmembrane protein 4690 1142-1147
44. VPRVEL B9PGI1 B9PGI1_TOXGO Brix domain-containing protein 401 289-294
45. PRVELV B9PGI1 B9PGI1_TOXGO Brix domain-containing protein 401 290-295
46. LMSDRK B9QM89 B9QM89_TOXGO Putative nucleolar gtp-binding protein 2 641 336-341
47. VVFADD B6KAU3 B6KAU3_TOXGO Chaperone dnaJ, related protein 804 155-160
48. VFADDT B6KAU3 B6KAU3_TOXGO Chaperone dnaJ, related protein 804 156-161
49. QEAIAQ V4ZFU3 V4ZFU3_TOXGO 14-3-3 superfamily protein 422 375-380
50. QILDFI V4ZE21 V4ZE21_TOXGO BTB/POZ domain-containing protein 357 230-235
51. EQQASV B9QLS7 B9QLS7_TOXGO WD domain, G-beta repeat-containing protein 2649 2551-2556
52. EQQASV V5BLA3 V5BLA3_TOXGO Putative transmembrane protein 2165 2141-2146
53. IFSIVT V4Z9R9 V4Z9R9_TOXGO Uncharacterized protein 79 23-28
54. DFVNKI V4ZFH5 V4ZFH5_TOXGO Translin family protein 273 200-205
55. VNKIRS V4ZF01 V4ZF01_TOXGO Putative RNA methylase 213 54-59
56. GWELEE V4YQX6 V4YQX6_TOXGO Uncharacterized protein 847 188-193
57. ELEEVL B9QFI2 B9QFI2_TOXGO Putative viral A-type inclusion repeat protein 951 76-81
58. LEEVLL B8Y892 B8Y892_TOXGO Choline kinase 630 425-430
59. LEEVLL B9QIG6 B9QIG6_TOXGO Phosphotransferase enzyme family protein 630 425-430
60. EEVLLL B9PNQ3 B9PNQ3_TOXGO Bystin protein 453 215-220
61. EEVLLL B9QN49 B9QN49_TOXGO Serine/threonine-protein phosphatase 552 235-240
62. EVLLLD V4ZSE4 V4ZSE4_TOXGO WD domain, G-beta repeat-containing protein 4664 1699-1704
63. LDMSLD B9PTB9 B9PTB9_TOXGO RNA recognition motif-containing protein 228 6-11
64. LDMSLD B9PZC9 B9PZC9_TOXGO RNA recognition motif-containing protein 372 13-18
65. DMSLDD B9PTB9 B9PTB9_TOXGO RNA recognition motif-containing protein 228 7-12
66. SLDDGD V4ZH21 V4ZH21_TOXGO GTPase 1352 969-974
67. LDDGDS V4YP36 V4YP36_TOXGO Uncharacterized protein 2331 2195-2200
68. DDGDSK B9PHR9 B9PHR9_TOXGO Josephin protein 412 224-229
69. NQLKKL V4Z808 V4Z808_TOXGO Uncharacterized protein 490 274-279
70. QLKKLQ Q1JT45 Q1JT45_TOXGO DNA ligase IV, putative 1023 962-967
71. QLKKLQ V4Z188 V4Z188_TOXGO ATP-dependent DNA ligase domain-containing protein 1385 1195-1200
72. QLKKLQ V4Z5X1 V4Z5X1_TOXGO Inner membrane protein 790 582-587
73. TKEEAT B9QB74 B9QB74_TOXGO NOL1/NOP2/Sun family protein 919 691-696
74. ANSVGL B6KK00 B6KK00_TOXGO Putative deoxyuridine 5'-triphosphate nucleotidohydrolase 170 86-91
75. VPSLVA V4Z7B8 V4Z7B8_TOXGO Alpha/beta hydrolase family protein 3457 823-828
76. VPSLVA V4ZGG2 V4ZGG2_TOXGO Uncharacterized protein 3620 891-896
77. PSLVAG V4Z3B4 V4Z3B4_TOXGO Transporter, solute:sodium symporter (SSS) family protein 617 461-466
78. SLVAGD Q1JTI4 Q1JTI4_TOXGO Methyltransferase, putative 541 483-488
79. SLVAGD V4ZJT3 V4ZJT3_TOXGO Uncharacterized protein 1521 423-428
80. GDTDTV B6KPA5 B6KPA5_TOXGO PAN domain-containing protein 438 187-192
81. GDTDTV Q1JSK6 Q1JSK6_TOXGO Micronemal protein 4, putative precursor 438 187-192
82. GDTDTV V4ZIV1 V4ZIV1_TOXGO Putative transmembrane protein 916 825-830
83. TDTVPA B9PMV8 B9PMV8_TOXGO Phosphorylated CTD-interacting factor 1 722 27-32
84. TDTVPA V4ZG98 V4ZG98_TOXGO RNB family domain-containing protein 1165 1121-1126
85. DTVPAE Q1JTE9 Q1JTE9_TOXGO Putative uncharacterized protein 514 403-408
86. DTVPAE V4YLJ7 V4YLJ7_TOXGO Uncharacterized protein 894 783-788
87. TVPAEF Q1JTE9 Q1JTE9_TOXGO Putative uncharacterized protein 514 404-409
88. TVPAEF V4YLJ7 V4YLJ7_TOXGO Uncharacterized protein 894 784-789
89. TVPAEF V4YV42 V4YV42_TOXGO Elongation factor G, mitochondrial 878 788-793
90. VPAEFP A3FKK0 A3FKK0_TOXGO Golgi-ER-type, P-type ATPase 1484 608-613
91. VPAEFP B9QI36 B9QI36_TOXGO Uncharacterized protein 1484 608-613
92. TGLISV B9QLW3 B9QLW3_TOXGO Uncharacterized protein 968 520-525
93. TGLISV V5B4U7 V5B4U7_TOXGO Peptidase M20D, amidohydrolase 514 329-334
94. SVSYDE V4ZFP2 V4ZFP2_TOXGO IgA-specific metalloendopeptidase 1814 1611-1616
95. DYGLPA V4ZCD1 V4ZCD1_TOXGO Zinc finger (CCCH type) motif-containing protein 1298 1011-1016
96. GLPARV V4YZA9 V4YZA9_TOXGO 3'5'-cyclic nucleotide phosphodiesterase domain-containing protein 1656 387-392
97. GLPARV V4Z8X8 V4Z8X8_TOXGO Uncharacterized protein 1707 533-538
98. ITTAAS V4ZCX5 V4ZCX5_TOXGO Toxoplasma gondii family B protein 208 93-98
99. NTHEKR V5B1X7 V5B1X7_TOXGO Glycosyl hydrolases family 35 protein 1876 1313-1318
100. EGRNLS V4ZLA3 V4ZLA3_TOXGO Uncharacterized protein 1205 376-381
101. NLSFSE V5BJC6 V5BJC6_TOXGO EF hand domain-containing protein 6368 5617-5622
102. LSFSED B6KVC2 B6KVC2_TOXGO Uncharacterized protein 1113 219-224
103. LSFSED V4ZGQ4 V4ZGQ4_TOXGO Uncharacterized protein 2272 2061-2066
104. LLNKER B6KGA9 B6KGA9_TOXGO Uncharacterized protein 1548 1451-1456
105. ETEEQE V4Z2Z1 V4Z2Z1_TOXGO Uncharacterized protein 1862 1285-1290
106. ETEEQE V5BI59 V5BI59_TOXGO WD domain, G-beta repeat-containing protein 3142 2139-2144
107. TEEQED Q2PP52 RHBL6_TOXGO Rhomboid-like protease 6 531 264-269
108. TEEQED V4ZAG4 V4ZAG4_TOXGO 3'5'-cyclic nucleotide phosphodiesterase domain-containing protein 2346 1318-1323
109. TEEQED V4ZJR6 V4ZJR6_TOXGO Rhomboid protease ROM6 588 264-269
110. EEQEDD B9QCD4 B9QCD4_TOXGO GTP-binding protein 428 159-164
111. EEQEDD V4Z3T6 V4Z3T6_TOXGO Uncharacterized protein 1806 396-401
112. EEQEDD V5B6A8 V5B6A8_TOXGO Uncharacterized protein 1142 846-851
113. IVESVD V4ZLU8 V4ZLU8_TOXGO SWI2/SNF2-containing protein 3776 3277-3282
114. SVDPLS V4ZI56 V4ZI56_TOXGO Uncharacterized protein 2083 567-572
115. KRIVTE B6KGK1 B6KGK1_TOXGO Uncharacterized protein 504 215-220
116. KTDEEP V4ZAM3 V4ZAM3_TOXGO Kazal-type serine protease inhibitor domain-containing protein 2035 1971-1976
117. TDEEPG D3XD37 D3XD37_TOXGO ATP-binding cassette transporter G family ABCG-84 protein 766 472-477
118. TDEEPG V4ZAM3 V4ZAM3_TOXGO Kazal-type serine protease inhibitor domain-containing protein 2035 1972-1977
119. ILKKIS V5B218 V5B218_TOXGO ATPase/histidine kinase/DNA gyrase B/HSP90 domain-containing protein 1440 1395-1400
120. KKISKS B6KJJ4 B6KJJ4_TOXGO Uncharacterized protein 178 165-170
121. VVMKRA Q1JTB7 Q1JTB7_TOXGO Dynein heavy chain, putative 4991 3162-3167
122. VVMKRA V4Z5R6 V4Z5R6_TOXGO Dynein heavy chain 4974 3145-3150
123. LIVSAV V4ZFJ2 V4ZFJ2_TOXGO Putative transmembrane protein 344 37-42
124. IVSAVA Q70GH0 Q70GH0_TOXGO Aquaglyceroporin 234 23-28
125. IVSAVA Q86C53 Q86C53_TOXGO Aquaporin 263 52-57
126. VSAVAV B6K8Q8 B6K8Q8_TOXGO Plasma membrane-type Ca(2+)-ATPase A1 PMCAA1 1822 133-138
127. VSAVAV Q1JT48 Q1JT48_TOXGO Putative transmembrane protein 521 119-124
128. VSAVAV V4ZMT5 V4ZMT5_TOXGO Nucleoside diphosphate kinase 602 125-130
129. VSAVAV V4ZRZ5 V4ZRZ5_TOXGO Uncharacterized protein 603 67-72
130. AVAVFV V5BG40 V5BG40_TOXGO Zinc finger, C3HC4 type (RING finger) domain-containing protein 304 113-118
131. RCLADG V5BA36 V5BA36_TOXGO DnaJ domain-containing protein 383 177-182
132. LADGRE V4ZTQ1 V4ZTQ1_TOXGO DEAD/DEAH box helicase domain-containing protein 1991 1701-1706
133. LADGRE V5BLE9 V5BLE9_TOXGO AP2 domain transcription factor AP2VIII-4 3417 2728-2733
134. ADGREP B9QMG4 B9QMG4_TOXGO Putative transmembrane protein 1215 1067-1072
135. DGREPG B6KGR7 B6KGR7_TOXGO Uncharacterized protein 979 412-417
136. DGREPG B9QGE1 B9QGE1_TOXGO ABC1 family protein 1917 518-523
137. VSGLSD V4Z880 V4Z880_TOXGO Uncharacterized protein 4668 4080-4085
138. VSGLSD V4ZS76 V4ZS76_TOXGO Uncharacterized protein 555 193-198
139. FSPPFR B9QE30 B9QE30_TOXGO AP2 domain transcription factor AP2IX-5 2282 1737-1742
140. VDDALL Q1JSD3 Q1JSD3_TOXGO Mannosyltransferase, putative 768 614-619
141. VDDALL V4ZB26 V4ZB26_TOXGO Putative mannosyltransferase 768 614-619
142. VDDALL V5BLD5 V5BLD5_TOXGO Adaptin c-terminal domain-containing protein 1672 1423-1428
143. DDALLS B9Q6B7 B9Q6B7_TOXGO EF hand domain-containing protein 297 67-72
144. DDALLS B9QJ49 B9QJ49_TOXGO Uncharacterized protein 1851 897-902
145. DDALLS V4Z9J7 V4Z9J7_TOXGO Microneme protein MIC7 340 321-326
146. DALLSL B9QEV3 B9QEV3_TOXGO Zinc finger, C3HC4 type (RING finger) domain-containing protein 3872 1866-1871
147. DALLSL B9QJ49 B9QJ49_TOXGO Uncharacterized protein 1851 898-903
148. DALLSL V4Z3P9 V4Z3P9_TOXGO Putative Tbc domain,related protein 3378 3093-3098
149. DALLSL V4Z9P5 V4Z9P5_TOXGO Microneme-like protein 759 44-49
150. DALLSL V4ZI56 V4ZI56_TOXGO Uncharacterized protein 2083 594-599
151. DALLSL V4ZIE9 V4ZIE9_TOXGO HEAT repeat-containing protein 3443 589-594
152. DALLSL V5B4G9 V5B4G9_TOXGO Uncharacterized protein 2072 980-985
153. DALLSL V5BB89 V5BB89_TOXGO Putative transmembrane protein 3658 2625-2630
154. ALLSLK Q1JTF3 Q1JTF3_TOXGO Putative uncharacterized protein 665 255-260
155. ALLSLK V4Z3S3 V4Z3S3_TOXGO DEAD/DEAH box helicase domain-containing protein 2095 1160-1165
156. ALLSLK V4Z5B0 V4Z5B0_TOXGO Uncharacterized protein 519 109-114
157. GSGKVF V4YXF7 V4YXF7_TOXGO Calcium dependent protein kinase CDPK7 2133 782-787
158. KVFAST B9Q0K8 B9Q0K8_TOXGO Tetratricopeptide repeat-containing protein 560 102-107
159. GWKRQV V4ZBW3 V4ZBW3_TOXGO Translation initiation factor eIF3 subunit 135 2322 694-699
160. LFGDGE B9PYE4 B9PYE4_TOXGO Putative type I fatty acid synthase 10021 3674-3679
161. LFGDGE B9QJP2 B9QJP2_TOXGO RNA polymerase-associated protein RTF1 783 603-608
162. LFGDGE Q1JTE1 Q1JTE1_TOXGO Type I fatty acid synthase, putative 9940 3593-3598
163. MEELEA Q1JSX6 Q1JSX6_TOXGO Kinesin-like protein 716 461-466
164. MEELEA V4YM26 V4YM26_TOXGO Kinesin motor domain-containing protein 825 513-518
165. EELEAL A7LNF0 A7LNF0_TOXGO ATP citrate lyase 1281 242-247
166. EELEAL B6K8T3 B6K8T3_TOXGO Putative IMPACT 482 37-42
167. EELEAL B9Q479 B9Q479_TOXGO WD domain, G-beta repeat-containing protein 3633 928-933
168. EELEAL B9Q7D3 B9Q7D3_TOXGO Putative ATP-citrate lyase 1281 242-247
169. EELEAL V4YRC2 V4YRC2_TOXGO Putative transmembrane protein 4703 3961-3966
170. EELEAL V4YZY2 V4YZY2_TOXGO ATPase (DUF699) protein 1247 254-259
171. EELEAL V4Z0W4 V4Z0W4_TOXGO Poly(A) polymerase, Cid1 family protein 425 400-405
172. EELEAL V4ZDH8 V4ZDH8_TOXGO ALG6, ALG8 glycosyltransferase family protein 1585 1423-1428
173. EELEAL V5AW67 V5AW67_TOXGO Putative methyltransferase 481 62-67
174. ELEALW V4ZD50 V4ZD50_TOXGO Putative type I fatty acid synthase 1643 860-865
175. LEALWL V4ZPY4 V4ZPY4_TOXGO RAVE 1 carboxy-terminal protein 6665 1019-1024
176. LEALWL V5B535 V5B535_TOXGO ImpB/MucB/SamB family protein 1253 467-472
177. VFYMLG B6KJK9 B6KJK9_TOXGO Uncharacterized protein 571 280-285
178. LGAAMA V5B8H8 V5B8H8_TOXGO Proteasome/cyclosome repeat-containing protein 167 18-23
179. ITFICE V4Z0C3 V4Z0C3_TOXGO Myb family DNA-binding domain-containing protein 1913 1386-1391
180. TFICEH V4Z0C3 V4Z0C3_TOXGO Myb family DNA-binding domain-containing protein 1913 1387-1392
181. AIEERQ V4ZAA2 V4ZAA2_TOXGO Uncharacterized protein 2719 2647-2652
182. AIEERQ V4ZCZ6 V4ZCZ6_TOXGO AP2 domain transcription factor AP2VIIa-4 3431 2368-2373
183. SNILRL B6KH85 B6KH85_TOXGO Uncharacterized protein 229 172-177
184. NILRLL B9PMD7 B9PMD7_TOXGO DNA-directed RNA polymerase II RPB1 1892 986-991
185. ILRLLR B9Q6I9 B9Q6I9_TOXGO Uncharacterized protein 5651 3908-3913
186. ILRLLR B9QR17 B9QR17_TOXGO Uncharacterized protein 6038 2628-2633
187. ILRLLR V4YZ80 V4YZ80_TOXGO HECT-domain (Ubiquitin-transferase) domain-containing protein 15897 1779-1784
188. LRLLRT Q1JSI2 Q1JSI2_TOXGO Uncharacterized protein precursor 1033 403-408
189. LRLLRT V4Z239 V4Z239_TOXGO WD domain, G-beta repeat-containing protein 2341 655-660
190. LRLLRT V4ZC68 V4ZC68_TOXGO Uncharacterized protein 257 224-229
191. LRLLRT V4ZD09 V4ZD09_TOXGO WD domain, G-beta repeat-containing protein 752 394-399
192. LRLLRT V4ZF78 V4ZF78_TOXGO Haloacid dehalogenase family hydrolase domain-containing protein 2473 768-773
193. LRLLRT V4ZHF5 V4ZHF5_TOXGO Uncharacterized protein 134 87-92
194. RLLRTA V4ZC68 V4ZC68_TOXGO Uncharacterized protein 257 225-230
195. RLLRTA V4ZCD1 V4ZCD1_TOXGO Zinc finger (CCCH type) motif-containing protein 1298 434-439
196. RLLRTA V4ZF78 V4ZF78_TOXGO Haloacid dehalogenase family hydrolase domain-containing protein 2473 769-774
197. RLLRTA V4ZP96 V4ZP96_TOXGO Putative transmembrane protein 907 731-736
198. NGSPQS V4ZHD9 V4ZHD9_TOXGO Putative transmembrane protein 1265 128-133
199. GSPQSA V4ZHD9 V4ZHD9_TOXGO Putative transmembrane protein 1265 129-134
200. SPQSAL B9QI26 B9QI26_TOXGO Pumilio-family RNA binding repeat-containing protein 1913 650-655
201. SPQSAL V4Z7U1 V4Z7U1_TOXGO HECT-domain (Ubiquitin-transferase) domain-containing protein 1978 439-444
202. SALDFI Q86PI1 Q86PI1_TOXGO Jlp2 550 223-228
203. SALDFI V4Z7A0 V4Z7A0_TOXGO Dense granule protein GRA15 635 224-229
204. LDFIRR V5BKJ8 V5BKJ8_TOXGO Suppressor of forked protein SUF 1295 541-546
205. RRESSV B9PK14 B9PK14_TOXGO Uncharacterized protein 386 262-267
206. RRESSV V4ZH67 V4ZH67_TOXGO Uncharacterized protein 3076 899-904
207. EENLFS V5BJX1 V5BJX1_TOXGO Uncharacterized protein 1332 757-762
208. ENLFSD V5BJX1 V5BJX1_TOXGO Uncharacterized protein 1332 758-763
209. FSDYIS V4YW85 V4YW85_TOXGO EF hand domain-containing protein 716 281-286
210. ISEVER V4ZVH8 V4ZVH8_TOXGO Uncharacterized protein 1104 883-888
211. LQLKDS V4YJP8 V4YJP8_TOXGO RecF/RecN/SMC N terminal domain-containing protein 1588 122-127
212. QLKDSN V4Z4U3 V4Z4U3_TOXGO ABC transporter, ATP-binding domain-containing protein 1885 267-272
213. SIGSAS V4ZCZ6 V4ZCZ6_TOXGO AP2 domain transcription factor AP2VIIa-4 3431 2829-2834
214. IGSASS V4Z3I2 V4Z3I2_TOXGO SCY kinase-related protein (Incomplete catalytic triad) 1065 1046-1051
215. IGSASS V4ZCZ6 V4ZCZ6_TOXGO AP2 domain transcription factor AP2VIIa-4 3431 2830-2835
216. GSASSI Q1PA41 Q1PA41_TOXGO Micronemal protein 15 2909 739-744
217. GSASSI V4ZMJ1 V4ZMJ1_TOXGO Uncharacterized protein 4969 4270-4275
218. GSASSI V5BFN0 V5BFN0_TOXGO Microneme protein MIC15 2924 754-759
219. SASSID B9QEZ9 B9QEZ9_TOXGO PUL domain-containing protein 898 454-459
220. SSIDGL B9QJM1 B9QJM1_TOXGO DEAD/DEAH box helicase domain-containing protein 1001 205-210
221. SSIDGL V4Z845 V4Z845_TOXGO CW-type Zinc Finger protein 1673 65-70
222. IDGLYD B6KFK3 B6KFK3_TOXGO C2 domain protein 453 201-206
223. NPPFTT V4YX42 V4YX42_TOXGO Rhoptry kinase family protein ROP30 470 350-355
224. QSRSIS B9Q266 B9Q266_TOXGO Endopeptidase, putative precursor 1038 88-93
225. SRSISK M9T1W1 M9T1W1_TOXGO ATG4 3753 17-22
226. SRSISK V4ZM71 V4ZM71_TOXGO Putative autophagy-related cysteine peptidase atg4 3747 17-22
227. DIGLPS V4ZAD9 V4ZAD9_TOXGO Protein kinase domain protein 789 650-655
228. GLPSSK V4Z9G4 V4Z9G4_TOXGO Putative cytochrome C family protein 183 170-175
229. HSQLSD Q1JT94 Q1JT94_TOXGO Putative uncharacterized protein 709 664-669
230. HSQLSD V4ZGA9 V4ZGA9_TOXGO GTP1/Obg protein 1917 442-447
231. HSQLSD V5B030 V5B030_TOXGO Uncharacterized protein 709 664-669
232. SQLSDL B6KGQ1 B6KGQ1_TOXGO Putative zinc finger protein 993 803-808
233. SQLSDL V4ZGA9 V4ZGA9_TOXGO GTP1/Obg protein 1917 443-448
234. QLSDLY V4ZF80 V4ZF80_TOXGO Uncharacterized protein 481 299-304
235. LSDLYG B6KGE9 B6KGE9_TOXGO Uncharacterized protein 2533 2373-2378
236. GKFSFK V4ZGI1 V4ZGI1_TOXGO Calcium-dependent protein kinase 2228 2214-2219
237. SDRYSG B6K922 B6K922_TOXGO Putative WD-40 repeat protein 437 82-87
238. RYSGHD B9Q5A1 B9Q5A1_TOXGO Tetratricopeptide repeat-containing protein 1031 491-496
239. DDLIRS B6K9L7 B6K9L7_TOXGO Putative vacuolar ATP synthase subunit C 404 63-68
240. RSDVSD V4ZFZ6 V4ZFZ6_TOXGO Ribosomal protein RPS6 993 726-731
241. RSDVSD V4ZLA6 V4ZLA6_TOXGO Putative transmembrane protein 1396 695-700
242. SDVSDI B9QMJ7 B9QMJ7_TOXGO Putative guanine nucleotide-binding protein 705 626-631
243. VSDIST V4ZFQ3 V4ZFQ3_TOXGO AP2 domain transcription factor AP2X-9 1876 69-74
244. SDISTH B6KK43 B6KK43_TOXGO Putative transmembrane protein 225 11-16
245. SDISTH Q0PH68 Q0PH68_TOXGO Dense granule protein GRA8 246 182-187
246. SDISTH Q9GSE9 Q9GSE9_TOXGO P35 surface antigen 378 198-203, 309-314
247. SDISTH Q9U4T9 Q9U4T9_TOXGO Dense granule protein GRA8 267 198-203
248. SDISTH V4Z4U6 V4Z4U6_TOXGO Dense granule protein GRA8 244 175-180
249. AAKRRK V4Z5W3 V4Z5W3_TOXGO OTU family cysteine protease 988 34-39
250. AAKRRK V4Z772 V4Z772_TOXGO AP2 domain transcription factor AP2X-11 1372 338-343
251. KRRKQQ V4ZPM1 V4ZPM1_TOXGO Uncharacterized protein 2234 1657-1662
252. DSLKKR B9PK30 B9PK30_TOXGO Mago binding protein 250 190-195
253. DSLKKR V4Z4I4 V4Z4I4_TOXGO Molybdopterin guanine dinucleotide synthesis protein B 2440 2248-2253
254. KKRPAS Q1JT85 Q1JT85_TOXGO Putative uncharacterized protein 663 88-93
255. KKRPAS V4YLR5 V4YLR5_TOXGO YrdC domain-containing protein 828 227-232
256. KKRPAS V4ZP24 V4ZP24_TOXGO Putative DNA mismatch repair protein MSH6-1 1567 280-285
257. KRPASA B9Q5E6 B9Q5E6_TOXGO Putative high molecular mass nuclear antigen 949 231-236
258. KRPASA V4YT41 V4YT41_TOXGO Protein kinase domain protein 6052 3190-3195
259. KRPASA V4ZN39 V4ZN39_TOXGO Uncharacterized protein 1167 423-428
260. KRPASA V4ZVZ1 V4ZVZ1_TOXGO Uncharacterized protein 2363 762-767
261. RPASAK V4YUP1 V4YUP1_TOXGO Uncharacterized protein 1171 137-142
262. PASAKS V4YUP1 V4YUP1_TOXGO Uncharacterized protein 1171 138-143
263. PASAKS V4Z3K0 V4Z3K0_TOXGO tRNA binding domain-containing protein 472 91-96
264. ASAKSR V4YSI1 V4YSI1_TOXGO Thiamin pyrophosphokinase, catalytic domain-containing protein 813 769-774
265. SAKSRR B6KRJ1 B6KRJ1_TOXGO Uncharacterized protein 2272 1762-1767
266. SAKSRR B9QFP6 B9QFP6_TOXGO Rad17 cell cycle checkpoint protein 1867 730-735
267. AKSRRE B6KHL3 B6KHL3_TOXGO Putative transmembrane protein 263 44-49
268. AKSRRE B9QFP6 B9QFP6_TOXGO Rad17 cell cycle checkpoint protein 1867 731-736
269. REFDEI V5BG16 V5BG16_TOXGO Uncharacterized protein 1767 1251-1256
270. EIELAY B9QJB7 B9QJB7_TOXGO Iron only hydrogenase large subunit, c-terminal domain-containing protein 981 894-899
271. IELAYR B9QJB7 B9QJB7_TOXGO Iron only hydrogenase large subunit, c-terminal domain-containing protein 981 895-900
272. IELAYR V4ZB23 V4ZB23_TOXGO Uncharacterized protein 909 527-532
273. AYRRRP B6KQU6 B6KQU6_TOXGO Cytoadherence-linked asexual protein 1490 1464-1469
274. AYRRRP Q1JTB2 Q1JTB2_TOXGO Putative uncharacterized protein precursor 1453 1427-1432
275. RRRPPR Q1JSA2 Q1JSA2_TOXGO Pre-mrna splicing factor protein, putative 429 172-177
276. RRRPPR V4YKM6 V4YKM6_TOXGO Zinc finger, C3HC4 type (RING finger) protein 1815 770-775
277. RRRPPR V4YMS8 V4YMS8_TOXGO Prp18 domain-containing protein 426 172-177
278. RRRPPR V4Z5U2 V4Z5U2_TOXGO TBC domain-containing protein 2711 1137-1142
279. RRRPPR V4Z6F3 V4Z6F3_TOXGO Protein phosphatase 2C domain-containing protein 886 581-586
280. RRRPPR V5B196 V5B196_TOXGO Uncharacterized protein 2539 2112-2117
281. RPPRSP B6KT44 B6KT44_TOXGO FUSE-binding protein 2 / KH-type splicing regulatory protein 941 640-645
282. RPPRSP V4YID7 V4YID7_TOXGO Uncharacterized protein 383 373-378
283. RPPRSP V4ZAE4 V4ZAE4_TOXGO Uncharacterized protein 217 207-212
284. PPRSPD V4ZTS0 V4ZTS0_TOXGO Uncharacterized protein 2211 1415-1420
285. PPRSPD V5BLE9 V5BLE9_TOXGO AP2 domain transcription factor AP2VIII-4 3417 2856-2861
286. LRDFYL V4Z6S4 V4Z6S4_TOXGO Ubiquitin carboxyl-terminal hydrolase 3600 896-901
287. EHVDLT V4ZBH2 V4ZBH2_TOXGO DnaJ domain-containing protein 645 326-331
288. SVSGGG B9QMX4 B9QMX4_TOXGO Toxoplasma gondii family A protein 612 263-268
289. SVSGGG V4YST6 V4YST6_TOXGO Uncharacterized protein 974 527-532
290. SVSGGG V4ZK17 V4ZK17_TOXGO Uncharacterized protein 2660 313-318
291. SVSGGG V4ZKG2 V4ZKG2_TOXGO Uncharacterized protein 2258 949-954
292. SVSGGG V5BGS1 V5BGS1_TOXGO Zinc finger (CCCH type) motif-containing protein 3460 420-425
293. VSGGGP V4ZIA7 V4ZIA7_TOXGO Uncharacterized protein 814 412-417, 442-447
294. VSGGGP V4ZS14 V4ZS14_TOXGO Putative acyl-CoA carboxyltransferase beta chain 812 264-269
295. KHGVVS V4YJS1 V4YJS1_TOXGO Phosphatidylinositol-4-phosphate 5-Kinase 4165 2921-2926
296. GVVSGV B9PY50 B9PY50_TOXGO 3-oxoacyl-[acyl-carrier-protein] synthase i/ii,putative precursor 551 136-141
297. GVVSGV B9QJ27 B9QJ27_TOXGO Optic atrophy 3 protein (Opa3) protein 352 195-200
298. VVSGVP V4ZWS2 V4ZWS2_TOXGO Uncharacterized protein 4834 3461-3466
299. VSGVPA B6KQJ2 B6KQJ2_TOXGO Uncharacterized protein 2318 1640-1645
300. VSGVPA Q1JT13 Q1JT13_TOXGO Putative uncharacterized protein 2318 1640-1645
301. VSGVPA V4ZRB7 V4ZRB7_TOXGO Toxoplasma gondii family A protein 384 269-274
302. SGVPAP B9Q700 B9Q700_TOXGO Nuclear fragile X mental retardation-interacting protein 1 830 468-473
303. SGVPAP Q1JTB6 Q1JTB6_TOXGO Methyl transferase, putative 842 11-16
304. SGVPAP V4ZRB7 V4ZRB7_TOXGO Toxoplasma gondii family A protein 384 270-275
305. SGVPAP V5B055 V5B055_TOXGO NOL1/NOP2/sun family protein 970 11-16
306. VEWEDR B9Q8D8 B9Q8D8_TOXGO Chloroquine resistance marker 3946 3877-3882
307. EWEDRS B9Q8D8 B9Q8D8_TOXGO Chloroquine resistance marker 3946 3878-3883
308. EDRSGG B6KHI3 B6KHI3_TOXGO Putative ARM repeat protein 891 176-181
309. EDRSGG Q1JSK4 Q1JSK4_TOXGO Uncharacterized protein 753 524-529
310. EDRSGG V4ZAI7 V4ZAI7_TOXGO Uncharacterized protein 753 524-529
311. SCPSKL B9QFF8 B9QFF8_TOXGO PHD-finger domain-containing protein 2138 357-362
312. STTVTG V4Z3N7 V4Z3N7_TOXGO Alpha/beta hydrolase family protein 1235 190-195
313. STTVTG V4ZRJ9 V4ZRJ9_TOXGO Putative transmembrane protein 2062 949-954
314. TGQNSG V5AZ15 V5AZ15_TOXGO Amine-terminal region of chorein, A TM vesicle-mediated sorter 8847 3307-3312
315. GQNSGR V4Z9J4 V4Z9J4_TOXGO PT repeat protein 1391 851-856
316. NSGRQA V4ZCU5 V4ZCU5_TOXGO Phosphatidylinositol 3-and 4-kinase 1228 666-671
317. SGRQAC V4YKM6 V4YKM6_TOXGO Zinc finger, C3HC4 type (RING finger) protein 1815 839-844
318. QACIRC B9PRH6 B9PRH6_TOXGO Rieske [2fe-2s] domain-containing protein 155 70-75
319. CEACKK V4Z845 V4Z845_TOXGO CW-type Zinc Finger protein 1673 537-542
320. EDNSLQ B9PUV4 B9PUV4_TOXGO Zinc finger protein 603 80-85
321. LDQPAA V4ZCV3 V4ZCV3_TOXGO Amine-terminal region of chorein, A TM vesicle-mediated sorter 9741 5987-5992
322. PAAPVA B9PHQ4 B9PHQ4_TOXGO Uncharacterized protein 223 39-44
323. PAAPVA B9PSH4 B9PSH4_TOXGO CHCH domain-containing protein 149 90-95
324. PAAPVA V4YJP8 V4YJP8_TOXGO RecF/RecN/SMC N terminal domain-containing protein 1588 743-748
325. PAAPVA V4Z9E6 V4Z9E6_TOXGO Uncharacterized protein 2263 2057-2062
326. PAAPVA V5B7N3 V5B7N3_TOXGO Uncharacterized protein 943 479-484
327. AAPVAV B6KL12 B6KL12_TOXGO RNA recognition motif-containing protein 705 5-10
328. AAPVAV Q5PY68 Q5PY68_TOXGO Nucleolar protein 534 5-10
329. AAPVAV V4Z2Q0 V4Z2Q0_TOXGO Uncharacterized protein 1684 8-13
330. APVAVT V4Z3A3 V4Z3A3_TOXGO Putative transmembrane protein 388 294-299
331. PVAVTS B9PN71 B9PN71_TOXGO Phosphatidylethanolamine-binding protein 281 105-110
332. PVAVTS V4ZCL5 V4ZCL5_TOXGO Uncharacterized protein 845 786-791
333. VAVTSN B9Q161 B9Q161_TOXGO Putative duplicated carbonic anhydrase 519 29-34
334. VAVTSN V4Z0W0 V4Z0W0_TOXGO Putative elongation factor TS 806 66-71
335. AVTSNA V4ZLJ6 V4ZLJ6_TOXGO MIZ/SP-RING zinc finger domain-containing protein 1779 1194-1199
336. TSNAST V4ZPQ9 V4ZPQ9_TOXGO Uncharacterized protein 1528 1275-1280
337. PTNSKA B9Q585 B9Q585_TOXGO Elongation factor Tu GTP binding domain-containing protein 1697 471-476
338. AQKKNR Q1JT06 Q1JT06_TOXGO Uncharacterized protein 1979 820-825
339. QKKNRN Q1JSE4 Q1JSE4_TOXGO Uncharacterized protein 363 203-208
340. KLRRQH B9Q6D5 B9Q6D5_TOXGO Uncharacterized protein 234 140-145
341. DTFVDL V4YXU2 V4YXU2_TOXGO Dynein heavy chain family protein 4551 1291-1296
342. DLQKEE B9Q6W4 B9Q6W4_TOXGO Uncharacterized protein 1708 610-615
343. QKEEAA B9QQE9 B9QQE9_TOXGO SAG-related sequence SRS47D 376 114-119
344. QKEEAA V4ZJ82 V4ZJ82_TOXGO Uncharacterized protein 1881 1011-1016
345. EEAALA K7WFT3 K7WFT3_TOXGO DHHC16 1047 465-470
346. EEAALA V4YMZ6 V4YMZ6_TOXGO Putative origin recognition complex subunit 878 508-513
347. EEAALA V4Z5Y2 V4Z5Y2_TOXGO Lecithin retinol acyltransferase 1008 744-749
348. EEAALA V4ZI37 V4ZI37_TOXGO DHHC zinc finger domain-containing protein 1047 465-470
349. EEAALA V4ZLT7 V4ZLT7_TOXGO Uncharacterized protein 1920 1373-1378
350. EEAALA V4ZX10 V4ZX10_TOXGO Putative transmembrane protein 3484 1135-1140
351. EAALAP V4YMY1 V4YMY1_TOXGO Tetratricopeptide repeat-containing protein 1008 922-927
352. EAALAP V4ZLT7 V4ZLT7_TOXGO Uncharacterized protein 1920 1374-1379
353. AALAPR B6KAF6 B6KAF6_TOXGO AP2 domain transcription factor AP2XI-2 2243 1606-1611
354. AALAPR V4Z9X5 V4Z9X5_TOXGO Uncharacterized protein 4983 1342-1347
355. ALAPRS B9Q325 B9Q325_TOXGO Putative DNA repair protein RecA 481 158-163
356. ALAPRS B9Q7H9 B9Q7H9_TOXGO Uncharacterized protein 2408 798-803
357. ALAPRS V4YKP3 V4YKP3_TOXGO Putative transmembrane protein 1321 604-609
358. APRSVS B9QI17 B9QI17_TOXGO Transcription initiation factor TFIID subunit TAF5 1095 736-741
359. APRSVS B9QQJ8 B9QQJ8_TOXGO Uncharacterized protein 1182 743-748
360. APRSVS Q1JSC7 Q1JSC7_TOXGO Uncharacterized protein 1182 743-748
361. APRSVS V4YM72 V4YM72_TOXGO Uncharacterized protein 2067 1991-1996
362. APRSVS V4Z5Y9 V4Z5Y9_TOXGO Putative transmembrane protein 635 267-272
363. PRSVSL B6KGZ6 B6KGZ6_TOXGO Putative transmembrane protein 256 216-221
364. PRSVSL B9QIX9 B9QIX9_TOXGO Uncharacterized protein 1904 139-144
365. PRSVSL V5AYI0 V5AYI0_TOXGO NEK kinase 2906 1249-1254
366. PRSVSL V5BDT5 V5BDT5_TOXGO Phosphatidylinositol 3-and 4-kinase 8859 6104-6109
367. SVSLKD V4ZG55 V4ZG55_TOXGO CBS domain-containing protein 924 755-760
368. SLKDKG B6KRG1 B6KRG1_TOXGO Putative late embryogenesis abundant domain protein 517 214-219
369. SAGEST B6K8M3 B6K8M3_TOXGO Leucine rich repeat-containing protein 1710 1242-1247
370. SAGEST B6KAF6 B6KAF6_TOXGO AP2 domain transcription factor AP2XI-2 2243 1237-1242
371. SAGEST V4YZG2 V4YZG2_TOXGO AP2 domain-containing protein 968 429-434
372. SAGEST V4Z0C3 V4Z0C3_TOXGO Myb family DNA-binding domain-containing protein 1913 122-127
373. SAGEST V4ZJ76 V4ZJ76_TOXGO Non-specific serine/threonine protein kinase 3401 2870-2875
374. SSVPTA B9QE30 B9QE30_TOXGO AP2 domain transcription factor AP2IX-5 2282 1302-1307
375. HHHNNP B9PXF8 B9PXF8_TOXGO Putative suppressor of kinetochore protein 1 170 68-73
376. NPGGGY V4Z7A4 V4Z7A4_TOXGO Histone lysine-specific demethylase LSD1/BHC110/KDMA1A 2947 1551-1556
377. RQPTVA V4ZPH3 V4ZPH3_TOXGO Protein kinase domain protein 2329 1678-1683
378. QPTVAG V5BLY3 V5BLY3_TOXGO DALR anticodon binding domain-containing protein 1307 1045-1050
379. PTVAGA B9PYA4 B9PYA4_TOXGO DEAD/DEAH box helicase domain-containing protein 2434 517-522
380. PTVAGA Q1JTA7 Q1JTA7_TOXGO Dead/deah box helicase, putative 2471 517-522
381. TVAGAS B6KRE5 B6KRE5_TOXGO Nucleoporin FG repeat region protein 698 484-489
382. TVAGAS V4ZS36 V4ZS36_TOXGO Uncharacterized protein 2025 1633-1638
383. AGASKA B6KJA3 B6KJA3_TOXGO Putative 50S ribosomal protein L3 514 149-154
384. AGASKA B6KUG4 B6KUG4_TOXGO Uncharacterized protein 1979 383-388
385. AGASKA B9Q5H6 B9Q5H6_TOXGO MIF4G domain-containing protein 2668 651-656
386. AGASKA Q1JTK6 Q1JTK6_TOXGO Putative uncharacterized protein precursor 1701 176-181
387. AGASKA V4ZPZ2 V4ZPZ2_TOXGO ARID/BRIGHT DNA binding domain-containing protein 2713 2525-2530
388. ASKARP V4Z8D2 V4Z8D2_TOXGO Uncharacterized protein 709 331-336
389. ASKARP V4ZJH9 V4ZJH9_TOXGO NIMA-related protein kinase NIMA1 1609 498-503
390. RPDFRA V4Z445 V4Z445_TOXGO Histone acetyltransferase TAF1/250 2775 1531-1536
391. RALVTN Q45W18 Q45W18_TOXGO ATP-binding cassette, sub-family G, member 1 794 300-305
392. RALVTN V4Z8I8 V4Z8I8_TOXGO ATP-binding cassette G family transporter ABCG87 794 300-305
393. PVVSAL B9Q562 B9Q562_TOXGO Non-specific serine/threonine protein kinase 8428 4496-4501
394. ALHGAV B6KJX9 B6KJX9_TOXGO V-type proton ATPase subunit a 909 493-498
395. GAVPAR V4ZQD7 V4ZQD7_TOXGO Dynein light intermediate chain 697 132-137
396. KNPRAF V4Z9G4 V4Z9G4_TOXGO Putative cytochrome C family protein 183 68-73
397. RAFNGS B9PQR4 B9PQR4_TOXGO Putative vacuolar ATP synthase subunit b 505 113-118
398. RAFNGS Q86N78 Q86N78_TOXGO Putative vacuolar H+ ATPase subunit B 409 113-118
399. YEKLSS V4Z6D9 V4Z6D9_TOXGO Subtilisin SUB11 1213 1099-1104
400. SSIESD Q1JSF3 Q1JSF3_TOXGO Uncharacterized protein 4600 4181-4186

**NMDA 2C**

1. MGGALG Q695T8 RHBL4_TOXGO Rhomboid-like protease 4 641 461-466
2. MGGALG V4Z6C0 V4Z6C0_TOXGO LsmAD domain-containing protein 1524 922-927
3. MGGALG V4Z806 V4Z806_TOXGO Rhomboid protease ROM4 665 485-490
4. GGALGP V4Z4I1 V4Z4I1_TOXGO PLU-1 family protein 8088 7788-7793
5. GGALGP V4ZGB0 V4ZGB0_TOXGO Zinc finger (CCCH type) motif-containing protein 1513 1237-1242
6. GGALGP V4ZMF4 V4ZMF4_TOXGO Uncharacterized protein 1585 651-656
7. GGALGP V4ZMJ1 V4ZMJ1_TOXGO Uncharacterized protein 4969 4790-4795
8. GALGPA V4YZG7 V4YZG7_TOXGO MCM2/3/5 family protein 1028 215-220
9. GALGPA V4ZMF4 V4ZMF4_TOXGO Uncharacterized protein 1585 652-657
10. ALGPAL V4ZG50 V4ZG50_TOXGO Uncharacterized protein 1834 125-130
11. LGPALL V4Z3A4 V4Z3A4_TOXGO Putative transmembrane protein 424 330-335
12. GPALLL V4Z3A4 V4Z3A4_TOXGO Putative transmembrane protein 424 331-336
13. GPALLL V4ZJ62 V4ZJ62_TOXGO Putative transmembrane protein 689 615-620
14. PALLLT V4Z8Q6 V4Z8Q6_TOXGO Uncharacterized protein 1568 144-149
15. PALLLT V4ZKA4 V4ZKA4_TOXGO Putative transmembrane protein 859 459-464
16. ALLLTS V4ZBN2 V4ZBN2_TOXGO Patched family protein 2498 1448-1453
17. ALLLTS V5B724 V5B724_TOXGO DEAD/DEAH box helicase domain-containing protein 1454 1202-1207
18. LLLTSL B9QR92 B9QR92_TOXGO Putative transmembrane protein 203 19-24
19. LLLTSL V4ZAH2 V4ZAH2_TOXGO Putative transmembrane protein 633 133-138
20. LLLTSL V4ZBH1 V4ZBH1_TOXGO Uncharacterized protein 3736 2272-2277
21. LLLTSL V4ZBN2 V4ZBN2_TOXGO Patched family protein 2498 1438-1443
22. LLLTSL V4ZGG2 V4ZGG2_TOXGO Uncharacterized protein 3620 2264-2269
23. LLLTSL V4ZSL1 V4ZSL1_TOXGO Putative transmembrane protein 1892 724-729
24. LLTSLF B6E3J1 B6E3J1_TOXGO ATP-binding cassette sub-family C member 1 1883 181-186
25. LLTSLF B6E3J4 B6E3J4_TOXGO ATP-binding cassette sub-family C member 1 1883 181-186
26. LLTSLF D0EP42 D0EP42_TOXGO ATP-binding cassette protein subfamily C member 1 1883 181-186
27. LLTSLF Q45W20 Q45W20_TOXGO ABC transporter transmembrane region domain-containing protein 1883 181-186
28. LLTSLF V4YP36 V4YP36_TOXGO Uncharacterized protein 2331 1677-1682
29. TSLFGA B6KBH4 B6KBH4_TOXGO Nucleoporin autopeptidase 2894 743-748
30. TSLFGA B6KRE5 B6KRE5_TOXGO Nucleoporin FG repeat region protein 698 88-93
31. TSLFGA V5BCF9 V5BCF9_TOXGO Putative proteophosphoglycan 5, related protein 1474 1332-1337
32. TSLFGA V5BLS9 V5BLS9_TOXGO Nucleoporin 723 420-425
33. GAWAGL V4ZIM8 V4ZIM8_TOXGO Alpha/beta hydrolase family protein 1781 1041-1046
34. WAGLGP B6KQK7 B6KQK7_TOXGO Cyclin protein 2572 475-480
35. WAGLGP Q1JT27 Q1JT27_TOXGO Putative uncharacterized protein 2626 529-534
36. AGLGPG B9Q8F4 B9Q8F4_TOXGO Uncharacterized protein 386 347-352
37. AGLGPG V4ZE60 V4ZE60_TOXGO Internal kinesin motor domain protein 1360 566-571
38. AGLGPG V4ZPZ2 V4ZPZ2_TOXGO ARID/BRIGHT DNA binding domain-containing protein 2713 244-249
39. GPGQGE V4ZLR9 V4ZLR9_TOXGO GYF domain-containing protein 1821 1248-1253
40. PGQGEQ B9Q465 B9Q465_TOXGO AP2 domain transcription factor AP2VIII-3 2084 2011-2016
41. GMTVAV V4ZSF6 V4ZSF6_TOXGO Cytidine and deoxycytidylate deaminase zinc-binding region domain-containing protein 514 506-511
42. TVAVVF A4UUQ4 A4UUQ4_TOXGO Dense granule antigen 229 21-26
43. TVAVVF A4UUQ8 A4UUQ8_TOXGO Dense granule antigen 230 21-26
44. TVAVVF A4UUQ9 A4UUQ9_TOXGO Dense granule antigen GRA6 230 21-26
45. TVAVVF A4UUR1 A4UUR1_TOXGO Dense granule antigen 225 21-26
46. TVAVVF A4UUR4 A4UUR4_TOXGO Dense granule antigen 230 21-26
47. TVAVVF A4UUR6 A4UUR6_TOXGO Dense granule antigen 230 21-26
48. TVAVVF A4UUR8 A4UUR8_TOXGO Dense granule antigen 230 21-26
49. TVAVVF A4UUS0 A4UUS0_TOXGO Dense granule antigen 230 21-26
50. TVAVVF A4UUS4 A4UUS4_TOXGO Dense granule antigen GRA6 229 21-26
51. TVAVVF A4UUS5 A4UUS5_TOXGO Dense granule antigen GRA6 230 21-26
52. TVAVVF A4UUS6 A4UUS6_TOXGO Dense granule antigen GRA6 230 21-26
53. TVAVVF A4UUT1 A4UUT1_TOXGO Dense granule antigen GRA6 230 21-26
54. TVAVVF A4UUT3 A4UUT3_TOXGO Dense granule antigen GRA6 230 21-26
55. TVAVVF A4UUU8 A4UUU8_TOXGO Dense granule antigen GRA6 229 21-26
56. TVAVVF A5JVX9 A5JVX9_TOXGO GRA6 73 21-26
57. TVAVVF A5JVY1 A5JVY1_TOXGO GRA6 73 21-26
58. TVAVVF A5JVY8 A5JVY8_TOXGO GRA6 73 21-26
59. TVAVVF D2Y4V7 D2Y4V7_TOXGO Dense granule antigen protein 6 222 19-24
60. TVAVVF D2Y4V8 D2Y4V8_TOXGO Dense granule antigen protein 6 211 19-24
61. TVAVVF D7PBL9 D7PBL9_TOXGO Dense granule antigen 6 77 21-26
62. TVAVVF D7PBM0 D7PBM0_TOXGO Dense granule antigen 6 77 21-26
63. TVAVVF D7PBM1 D7PBM1_TOXGO Dense granule antigen 6 77 21-26
64. TVAVVF D7PBM2 D7PBM2_TOXGO Dense granule antigen 6 77 21-26
65. TVAVVF F8TD24 F8TD24_TOXGO Granule antigen protein 6 126 21-26
66. TVAVVF F8TD25 F8TD25_TOXGO Granule antigen protein 6 126 21-26
67. TVAVVF F8TD26 F8TD26_TOXGO Granule antigen protein 6 126 21-26
68. TVAVVF F8TD27 F8TD27_TOXGO Granule antigen protein 6 126 21-26
69. TVAVVF G8HWZ7 G8HWZ7_TOXGO Dense granule protein 230 21-26
70. TVAVVF G8HWZ8 G8HWZ8_TOXGO Dense granule protein 230 21-26
71. TVAVVF I7CL27 I7CL27_TOXGO Dense granule antigen 230 21-26
72. TVAVVF Q19PW5 Q19PW5_TOXGO Dense granule antigen 230 21-26
73. TVAVVF Q1RS38 Q1RS38_TOXGO Dense granule antigen 230 21-26
74. TVAVVF Q1RS39 Q1RS39_TOXGO Granule antigen protein 230 21-26
75. TVAVVF Q1RS40 Q1RS40_TOXGO Granule antigen protein 224 21-26
76. TVAVVF Q25C67 Q25C67_TOXGO Dense granule antigen GRA6 224 21-26
77. TVAVVF Q25C68 Q25C68_TOXGO Dense granule antigen GRA6 230 21-26
78. TVAVVF Q25C69 Q25C69_TOXGO Dense granule antigen 230 21-26
79. TVAVVF Q25C71 Q25C71_TOXGO Dense granule antigen GRA6 230 21-26
80. TVAVVF Q25C72 Q25C72_TOXGO Dense granule antigen 224 21-26
81. TVAVVF Q25C73 Q25C73_TOXGO Dense granule antigen GRA6 230 21-26
82. TVAVVF Q25C76 Q25C76_TOXGO Dense granule antigen GRA6 224 21-26
83. TVAVVF Q27003 GRA6_TOXGO Dense granule protein 6 230 21-26
84. VAVVFS B9Q846 B9Q846_TOXGO SAG-related sequence SRS67 210 194-199
85. VAVVFS V4Z6I2 V4Z6I2_TOXGO Putative DP-fucose transporter 394 299-304
86. VVFSSS B6KU03 B6KU03_TOXGO Putative 50S ribosomal protein L17 552 246-251
87. VVFSSS B9QKE6 B9QKE6_TOXGO Ribulose 5-phosphate isomerase 259 146-151
88. FSSSGP B6KK02 B6KK02_TOXGO Homeodomain-like domain protein 1874 826-831
89. FSSSGP B9QGZ1 B9QGZ1_TOXGO RAP domain-containing protein 571 386-391
90. FSSSGP V4ZHS6 V4ZHS6_TOXGO Putative transmembrane protein 889 563-568
91. SSSGPP B9PY21 B9PY21_TOXGO ACR-like protein 1530 250-255
92. SSSGPP B9QPK1 B9QPK1_TOXGO Histone deacetylase HDAC1 2383 1680-1685
93. SSSGPP V4ZC41 V4ZC41_TOXGO Uncharacterized protein 496 399-404
94. SSSGPP V4ZMH1 V4ZMH1_TOXGO Uncharacterized protein 4312 2407-2412
95. SSGPPQ B9PVZ8 B9PVZ8_TOXGO Putative transmembrane protein 360 18-23
96. SGPPQA V4Z7K0 V4Z7K0_TOXGO Uncharacterized protein 1512 441-446
97. RARLTP V4ZC76 V4ZC76_TOXGO Phosphofructokinase domain-containing protein 3001 2955-2960
98. RLTPQS B9QFV9 B9QFV9_TOXGO 3'5'-cyclic nucleotide phosphodiesterase domain-containing protein 1676 1245-1250
99. RLTPQS V4Z8N7 V4Z8N7_TOXGO Uncharacterized protein 3607 2532-2537
100. QSFLDL V4Z9X6 V4Z9X6_TOXGO Pumilio-family RNA binding repeat-containing protein 1676 239-244
101. FLDLPL B9Q2T3 B9Q2T3_TOXGO Putative elongation factor 1-gamma 394 21-26
102. FLDLPL V4ZIR0 V4ZIR0_TOXGO Ubiquitin carboxyl-terminal hydrolase 2294 1021-1026
103. TTNPSS V4Z1Y1 V4Z1Y1_TOXGO Regulator of chromosome condensation (RCC1) repeat-containing protein 1761 1146-1151
104. TNPSSL V4Z1Y1 V4Z1Y1_TOXGO Regulator of chromosome condensation (RCC1) repeat-containing protein 1761 1147-1152
105. NPSSLL V4Z7B4 V4Z7B4_TOXGO PEK kinase 564 159-164
106. NPSSLL V4ZJ56 V4ZJ56_TOXGO Uncharacterized protein 1480 1087-1092
107. PSSLLT B6K9V5 B6K9V5_TOXGO Uncharacterized protein 1315 318-323
108. PSSLLT V4YJ42 V4YJ42_TOXGO Leucine rich repeat-containing protein 912 870-875
109. PSSLLT V4ZAZ4 V4ZAZ4_TOXGO AP2 domain transcription factor AP2X-7 1869 1111-1116
110. PSSLLT V4ZIK3 V4ZIK3_TOXGO Eukaryotic aspartyl protease superfamily protein 921 326-331
111. PSSLLT V4ZJH9 V4ZJH9_TOXGO NIMA-related protein kinase NIMA1 1609 1573-1578
112. PSSLLT V4ZL32 V4ZL32_TOXGO Uncharacterized protein 1689 1078-1083
113. PSSLLT V5B6D1 V5B6D1_TOXGO Penicillin amidase 1149 414-419
114. PSSLLT V5BHN2 V5BHN2_TOXGO Transmembrane amino acid transporter 2130 936-941
115. SSLLTQ B9QP66 B9QP66_TOXGO VMA21-like domain protein 185 43-48
116. SSLLTQ V4Z3G1 V4Z3G1_TOXGO Dynein heavy chain family protein 4610 4491-4496
117. SSLLTQ V4Z7Q3 V4Z7Q3_TOXGO Uncharacterized protein 2521 1818-1823
118. SSLLTQ V4ZIK3 V4ZIK3_TOXGO Eukaryotic aspartyl protease superfamily protein 921 327-332
119. SSLLTQ V5B872 V5B872_TOXGO Myb family DNA-binding domain-containing protein 1755 678-683
120. LLTQIC V4ZGL5 V4ZGL5_TOXGO RAP domain-containing protein 1856 1195-1200
121. CGLLGA V5B8R6 V5B8R6_TOXGO SacI-like domain protein 2122 374-379
122. GLLGAA B6KBH4 B6KBH4_TOXGO Nucleoporin autopeptidase 2894 666-671
123. GLLGAA V4Z2F1 V4Z2F1_TOXGO Dense granule protein GRA11 471 293-298
124. GLLGAA V4Z8H6 V4Z8H6_TOXGO Uncharacterized protein 656 266-271
125. GLLGAA V4ZPZ4 V4ZPZ4_TOXGO Uncharacterized protein 837 56-61
126. NVDTEA B6KFG2 B6KFG2_TOXGO Sec1 family protein 633 467-472
127. VDTEAV V4ZWM5 V4ZWM5_TOXGO Putative blood stage antigen 41-3 441 265-270
128. DTEAVA Q1JSD2 Q1JSD2_TOXGO Uncharacterized protein 1409 1358-1363
129. DTEAVA V4Z6L1 V4Z6L1_TOXGO Protein kinase domain protein 1375 1324-1329
130. VAQILD B9Q560 B9Q560_TOXGO Transport protein Trs120 2958 1046-1051
131. SISGGS V4ZUZ7 V4ZUZ7_TOXGO Uncharacterized protein 1822 1628-1633
132. SISGGS V5BGY6 V5BGY6_TOXGO Nuclear protein Es2 799 737-742
133. ISGGSA V4Z4F5 V4Z4F5_TOXGO Uncharacterized protein 2203 799-804
134. ISGGSA V4ZUZ7 V4ZUZ7_TOXGO Uncharacterized protein 1822 1629-1634
135. SGGSAV V4YY19 V4YY19_TOXGO Uncharacterized protein 411 118-123
136. SGGSAV V4Z4K9 V4Z4K9_TOXGO Putative nucleoporin FG repeat region protein 5638 3200-3205
137. GGSAVV A8WAD2 A8WAD2_TOXGO ATP-binding cassette protein subfamily B member 1 1345 610-615
138. GGSAVV Q4FCM2 Q4FCM2_TOXGO ATP-binding cassette protein subfamily B member 1 1345 610-615
139. GSAVVL B9QGG5 B9QGG5_TOXGO Uncharacterized protein 247 189-194
140. SAVVLT V4ZIE0 V4ZIE0_TOXGO DNA polymerase 4247 2212-2217
141. AVVLTP V4ZW36 V4ZW36_TOXGO Putative adenylate cyclase 2040 1118-1123
142. LTPKEP V4ZKZ6 V4ZKZ6_TOXGO Histidine acid phosphatase superfamily protein 875 267-272
143. PKEPGS V4ZCA5 V4ZCA5_TOXGO Methyltransferase TRM13 923 515-520
144. PGSAFL B6K8P4 B6K8P4_TOXGO Uncharacterized protein 3213 1693-1698
145. PGSAFL B6KRE0 B6KRE0_TOXGO Uncharacterized protein 835 100-105
146. PGSAFL B9Q5J3 B9Q5J3_TOXGO Uncharacterized protein 1001 780-785
147. PGSAFL Q1JSI5 Q1JSI5_TOXGO Uncharacterized protein 3127 461-466
148. PGSAFL V4Z6D7 V4Z6D7_TOXGO Uncharacterized protein 2936 315-320
149. SAFLQL B9PPS0 B9PPS0_TOXGO Thioredoxin domain-containing protein 312 255-260
150. SAFLQL B9Q7T1 B9Q7T1_TOXGO Putative COP9 signalosome complex subunit 3 579 200-205
151. SAFLQL V4ZIP7 V4ZIP7_TOXGO Eukaryotic initiation factor 4E 2044 366-371
152. LQLGVS V4ZD79 V4ZD79_TOXGO Utp14 1028 356-361
153. QLGVSL V4ZQQ5 V4ZQQ5_TOXGO Sad1 / UNC family C-terminal protein 796 229-234
154. LGVSLE B6KRJ8 B6KRJ8_TOXGO Uncharacterized protein 2419 1383-1388
155. LGVSLE B9Q479 B9Q479_TOXGO WD domain, G-beta repeat-containing protein 3633 1105-1110
156. LGVSLE V4Z6F0 V4Z6F0_TOXGO Putative DUF775 domain protein 188 107-112
157. LGVSLE V4ZKD8 V4ZKD8_TOXGO Uncharacterized protein 11926 3312-3317
158. GVSLEQ V4ZPD2 V4ZPD2_TOXGO Uncharacterized protein 903 736-741
159. VSLEQQ V4YZC1 V4YZC1_TOXGO Uncharacterized protein 3026 1436-1441
160. LEQQLQ V4ZJY0 V4ZJY0_TOXGO Uncharacterized protein 2547 735-740
161. LEQQLQ V5BHS4 V5BHS4_TOXGO Putative guanylate-binding protein 1396 1318-1323
162. EQQLQV V4YMZ0 V4YMZ0_TOXGO Sushi domain (Scr repeat) domain-containing protein 3916 1304-1309
163. EQQLQV V4ZJY0 V4ZJY0_TOXGO Uncharacterized protein 2547 736-741
164. PGHALF B6KGY9 B6KGY9_TOXGO Uncharacterized protein 2303 1626-1631
165. HALFLE B9QPC3 B9QPC3_TOXGO Putative transmembrane protein 316 17-22
166. HALFLE V4ZR42 V4ZR42_TOXGO Uncharacterized protein 1471 909-914
167. LEGVRA B6KB04 B6KB04_TOXGO Uncharacterized protein 621 256-261
168. LEGVRA B6KFK2 B6KFK2_TOXGO Peptidyl-prolyl cis-trans isomerase 575 315-320
169. LEGVRA V4ZLU0 V4ZLU0_TOXGO Phospholipase, patatin family protein 656 559-564
170. EGVRAV V4ZB19 V4ZB19_TOXGO Uncharacterized protein 386 330-335
171. EGVRAV V4ZJ55 V4ZJ55_TOXGO Enoyl-CoA hydratase/isomerase family protein 585 539-544
172. RAVADA V4ZBY1 V4ZBY1_TOXGO Putative transmembrane protein 3864 469-474
173. AVADAS B9PRV7 B9PRV7_TOXGO Zn-finger in Ran binding protein and others domain-containing protein 1258 867-872
174. AVADAS B9Q751 B9Q751_TOXGO Subtilisin SUB8 1366 1142-1147
175. AVADAS V4Z435 V4Z435_TOXGO Putative histone lysine methyltransferase, SET 1798 486-491
176. AVADAS V5AX26 V5AX26_TOXGO Uncharacterized protein 1105 542-547
177. DASHVS V4ZJU2 V4ZJU2_TOXGO Putative transmembrane protein 2174 1734-1739
178. WRLLDV V4Z5Y4 V4Z5Y4_TOXGO CRAL/TRIO domain protein 1773 983-988
179. RLLDVV B9QP11 B9QP11_TOXGO CMGC kinase, CK2 family 539 290-295
180. RLLDVV V4ZEW9 V4ZEW9_TOXGO CorA family Mg2+ transporter protein 1190 454-459
181. DVVTLE A1E140 A1E140_TOXGO Rhoptry protein 18 586 359-364
182. DVVTLE B9PXV0 B9PXV0_TOXGO Uncharacterized protein 451 234-239
183. DVVTLE I7CBH8 I7CBH8_TOXGO Rhoptry kinase family protein 556 329-334
184. DVVTLE I7CQ43 I7CQ43_TOXGO Rhoptry kinase family protein 556 329-334
185. DVVTLE Q0GBR6 Q0GBR6_TOXGO Secretory serine-threonine protein kinase ROP18-III 541 314-319
186. DVVTLE Q1JSH6 Q1JSH6_TOXGO Uncharacterized protein 384 167-172
187. VTLELG V4Z3T0 V4Z3T0_TOXGO Aldehyde dehydrogenase 497 254-259
188. VTLELG V4Z4T2 V4Z4T2_TOXGO Putative dynein gamma chain, flagellar outer arm 2639 627-632
189. LELGPG V4Z3X9 V4Z3X9_TOXGO Uncharacterized protein 2041 972-977
190. LGPGGP B9QPP0 B9QPP0_TOXGO Uncharacterized protein 2806 622-627
191. LGPGGP V4ZIE7 V4ZIE7_TOXGO Uncharacterized protein 742 531-536
192. LGPGGP V5AZX8 V5AZX8_TOXGO Uncharacterized protein 713 10-15
193. GPGGPR B6KA08 B6KA08_TOXGO TAF7-like RNA polymerase II TAF7L 728 509-514
194. PGGPRA B9QR17 B9QR17_TOXGO Uncharacterized protein 6038 222-227
195. GGPRAR B9QR17 B9QR17_TOXGO Uncharacterized protein 6038 223-228
196. GGPRAR V4ZBG2 V4ZBG2_TOXGO 3'5'-cyclic nucleotide phosphodiesterase domain-containing protein 3476 2707-2712
197. RARTQR Q1JTI3 Q1JTI3_TOXGO Ubiquitin-protein ligase 1, putative 8112 1705-1710
198. RARTQR V4Z553 V4Z553_TOXGO HECT-domain (Ubiquitin-transferase) domain-containing protein 8007 1705-1710
199. ARTQRL B9QFW3 B9QFW3_TOXGO AP2 domain transcription factor AP2VIIa-7 3112 641-646
200. ARTQRL V4ZB93 V4ZB93_TOXGO Putative ATP-dependent hsl protease ATP-binding subunit hslU 672 39-44
201. ARTQRL V5B7I4 V5B7I4_TOXGO Cysteine dioxygenase type i protein 214 63-68
202. RTQRLL V5B7I4 V5B7I4_TOXGO Cysteine dioxygenase type i protein 214 64-69
203. TQRLLR B6KFK7 B6KFK7_TOXGO 3'5'-cyclic nucleotide phosphodiesterase domain-containing protein 1281 1146-1151
204. TQRLLR V4ZIV1 V4ZIV1_TOXGO Putative transmembrane protein 916 348-353
205. QRLLRQ B6KP01 B6KP01_TOXGO Rhoptry kinase family protein ROP22 (Incomplete catalytic triad) 611 560-565
206. QRLLRQ B9Q072 B9Q072_TOXGO RNA recognition motif-containing protein 274 60-65
207. QRLLRQ Q1JSX3 Q1JSX3_TOXGO Uncharacterized protein precursor 611 560-565
208. RLLRQL V4YNF3 V4YNF3_TOXGO Uncharacterized protein 1631 1089-1094
209. RLLRQL V4ZCV5 V4ZCV5_TOXGO Polo kinase 697 280-285
210. RLLRQL V4ZER7 V4ZER7_TOXGO Uncharacterized protein 3307 1814-1819
211. RLLRQL V4ZJ32 V4ZJ32_TOXGO Uncharacterized protein 1284 319-324
212. LLRQLD B9QGQ9 B9QGQ9_TOXGO Leucine rich repeat-containing protein 644 101-106
213. LLRQLD V4YZ95 V4YZ95_TOXGO Kinesin motor domain-containing protein 2394 468-473
214. LRQLDA V4ZAN5 V4ZAN5_TOXGO Serine/threonine specific protein phosphatase 2462 1744-1749
215. LDAPVF V5B7K5 V5B7K5_TOXGO Putative 2-oxoglutarate dehydrogenase e1 component, mitochondrial 1116 540-545
216. DAPVFV B9Q7B0 B9Q7B0_TOXGO Uncharacterized protein 700 81-86
217. APVFVA B9QGJ2 B9QGJ2_TOXGO Plasma-membrane choline transporter 1060 988-993
218. FVAYCS B6KKY8 B6KKY8_TOXGO Putative MYND finger protein 1059 1035-1040
219. AYCSRE V4Z8S4 V4Z8S4_TOXGO AP2 domain transcription factor AP2III-4 1645 285-290
220. CSREEA B9QFR7 B9QFR7_TOXGO Uncharacterized protein 2197 1082-1087
221. SREEAE B6KRY2 B6KRY2_TOXGO Uncharacterized protein 1745 1222-1227
222. SREEAE B9Q5X2 B9Q5X2_TOXGO Putative transmembrane protein 347 96-101
223. SREEAE B9QLY4 B9QLY4_TOXGO RNA methyltransferase, TrmH family protein 2898 152-157
224. SREEAE V4YTX2 V4YTX2_TOXGO Uncharacterized protein 2251 347-352
225. SREEAE V4ZFK6 V4ZFK6_TOXGO ER-trafficking TRAPP I complex 85 kDa subunit 3377 2890-2895
226. SREEAE V4ZL10 V4ZL10_TOXGO Putative transmembrane protein 2026 1267-1272
227. SREEAE V4ZLM2 V4ZLM2_TOXGO Polynucleotide adenylyltransferase 2477 1433-1438
228. SREEAE V4ZRH7 V4ZRH7_TOXGO Uncharacterized protein 808 197-202
229. SREEAE V4ZV34 V4ZV34_TOXGO Inositol monophosphatase family protein 1032 266-271
230. REEAEV B9QHU8 B9QHU8_TOXGO RIO1 family protein 1008 500-505
231. REEAEV H3K408 H3K408_TOXGO CaMK-related kinase 3196 1073-1078
232. REEAEV V4Z7U1 V4Z7U1_TOXGO HECT-domain (Ubiquitin-transferase) domain-containing protein 1978 277-282
233. REEAEV V4ZAJ4 V4ZAJ4_TOXGO Histone lysine demethylase JMJD5 1087 707-712
234. REEAEV V4ZHL8 V4ZHL8_TOXGO HECT-domain (Ubiquitin-transferase) domain protein 762 715-720
235. REEAEV V4ZRP3 V4ZRP3_TOXGO RecF/RecN/SMC N terminal domain-containing protein 2097 1048-1053
236. EEAEVL V4Z7U1 V4Z7U1_TOXGO HECT-domain (Ubiquitin-transferase) domain-containing protein 1978 278-283
237. LFAEAA B9PY64 B9PY64_TOXGO DHHC zinc finger domain-containing protein 971 119-124
238. LFAEAA B9QNC9 B9QNC9_TOXGO SAG-related sequence SRS46 376 22-27
239. LFAEAA Q1JSJ8 Q1JSJ8_TOXGO Uncharacterized protein 2342 1088-1093
240. LFAEAA V4YQH8 V4YQH8_TOXGO DnaJ domain-containing protein 397 120-125
241. LFAEAA V4ZCP1 V4ZCP1_TOXGO OTU family cysteine protease 1395 654-659
242. LFAEAA V4ZQZ0 V4ZQZ0_TOXGO Organic solute transporter ostalpha protein 684 551-556
243. LFAEAA V4ZUU4 V4ZUU4_TOXGO UBA/TS-N domain-containing protein 1868 210-215
244. LFAEAA V5B0W4 V5B0W4_TOXGO Uncharacterized protein 1806 1088-1093
245. FAEAAQ Q1JSJ8 Q1JSJ8_TOXGO Uncharacterized protein 2342 1089-1094
246. FAEAAQ V5B0W4 V5B0W4_TOXGO Uncharacterized protein 1806 1089-1094
247. AEAAQA A4GT85 A4GT85_TOXGO Sugar transporter 689 539-544
248. AEAAQA B6KAZ3 B6KAZ3_TOXGO Putative proteophosphoglycan 5, related protein 2250 2048-2053
249. AEAAQA B9QHU8 B9QHU8_TOXGO RIO1 family protein 1008 302-307
250. AEAAQA B9QQK5 B9QQK5_TOXGO CS domain protein 232 227-232
251. AEAAQA Q0PW51 Q0PW51_TOXGO P23 co-chaperone 226 221-226
252. AEAAQA Q1JSB8 Q1JSB8_TOXGO p23-like protein 232 227-232
253. AEAAQA Q1JSJ8 Q1JSJ8_TOXGO Uncharacterized protein 2342 1090-1095
254. AEAAQA Q1JTB4 Q1JTB4_TOXGO Putative uncharacterized protein 503 425-430
255. AEAAQA V4Z1G9 V4Z1G9_TOXGO Programmed cell death protein 2, c-terminal domain-containing protein 584 506-511
256. AEAAQA V4Z3S3 V4Z3S3_TOXGO DEAD/DEAH box helicase domain-containing protein 2095 834-839
257. AEAAQA V4Z685 V4Z685_TOXGO Uncharacterized protein 698 550-555
258. AEAAQA V4Z7A4 V4Z7A4_TOXGO Histone lysine-specific demethylase LSD1/BHC110/KDMA1A 2947 868-873
259. AEAAQA V5B0W4 V5B0W4_TOXGO Uncharacterized protein 1806 1090-1095
260. EAAQAG V5B3X2 V5B3X2_TOXGO GATA zinc finger domain-containing protein 2012 452-457
261. QAGLVG V4Z532 V4Z532_TOXGO Lipoyl(Octanoyl) transferase 910 707-712
262. AGLVGP B6K9V8 B6K9V8_TOXGO RNA pseudouridine synthase superfamily protein 2780 1175-1180
263. AGLVGP B9QJF2 B9QJF2_TOXGO Toxoplasma gondii family A protein 416 274-279
264. LALGST B9QN40 B9QN40_TOXGO Carbamoylphosphate synthetase 1699 798-803
265. LALGST Q8T9L7 Q8T9L7_TOXGO Carbamoyl phosphate synthetase II 1687 786-791
266. LALGST V4Z5I7 V4Z5I7_TOXGO Rtr1/RPAP2 family protein 1653 362-367
267. LALGST V4ZB30 V4ZB30_TOXGO Uncharacterized protein 204 108-113
268. ALGSTD A8CBF6 A8CBF6_TOXGO Delta-aminolevulinic acid synthetase 584 29-34
269. ALGSTD V4ZIT3 V4ZIT3_TOXGO 5-aminolevulinic acid synthase domain-containing protein 752 92-97
270. LGSTDA A8CBF6 A8CBF6_TOXGO Delta-aminolevulinic acid synthetase 584 30-35
271. LGSTDA V4ZIT3 V4ZIT3_TOXGO 5-aminolevulinic acid synthase domain-containing protein 752 93-98
272. GSTDAP V4Z8W6 V4Z8W6_TOXGO Zinc knuckle domain-containing protein 218 96-101
273. STDAPP Q1JT23 Q1JT23_TOXGO Putative uncharacterized protein 1439 1005-1010
274. STDAPP V4Z164 V4Z164_TOXGO Uncharacterized protein 1439 1005-1010
275. STDAPP V5AWX9 V5AWX9_TOXGO Uncharacterized protein 1272 956-961
276. TDAPPA B9PTF5 B9PTF5_TOXGO Hydrolase, NUDIX family protein 300 148-153
277. TDAPPA V4ZE20 V4ZE20_TOXGO AP2 domain transcription factor AP2V-2 3456 1027-1032
278. TDAPPA V5BI30 V5BI30_TOXGO Uncharacterized protein 187 134-139
279. PPATFP V4ZPP7 V4ZPP7_TOXGO Putative transmembrane protein 285 90-95
280. TFPVGL V5AZ15 V5AZ15_TOXGO Amine-terminal region of chorein, A TM vesicle-mediated sorter 8847 4315-4320
281. ESWRLS V4ZBB6 V4ZBB6_TOXGO Uncharacterized protein 3325 424-429
282. RLSLRQ Q86GL5 Q86GL5_TOXGO Peroxiredoxin 3 283 9-14
283. RLSLRQ V4Z316 V4Z316_TOXGO Uncharacterized protein 1343 340-345
284. RLSLRQ V5BDH0 V5BDH0_TOXGO Tsp1 domain TSP12 (Precursor),related protein 1329 31-36
285. LSLRQK V4Z4H9 V4Z4H9_TOXGO Uncharacterized protein 1019 273-278
286. SLRQKV V4Z4H9 V4Z4H9_TOXGO Uncharacterized protein 1019 274-279
287. LRQKVR B6KAZ7 B6KAZ7_TOXGO Myosin J 2532 1346-1351
288. KVRDGV V4ZG90 V4ZG90_TOXGO Sin3-associated polypeptide SAP18 868 129-134
289. GVAILA V4ZLW4 V4ZLW4_TOXGO SAG-related sequence SRS55F 383 366-371
290. GVAILA V4ZRM7 V4ZRM7_TOXGO SAG-related sequence SRS55A 374 357-362
291. AILALG V4Z1J8 V4Z1J8_TOXGO Uncharacterized protein 2920 1165-1170
292. AILALG V4Z377 V4Z377_TOXGO Ribosomal protein L15 protein 1395 1175-1180
293. QHGTLP V4ZBM6 V4ZBM6_TOXGO Uncharacterized protein 1503 825-830
294. HGTLPA V4Z2F7 V4Z2F7_TOXGO Putative transmembrane protein 2862 197-202
295. TLPAPA Q2LKW8 Q2LKW8_TOXGO Transcriptional co-activator ADA2-B 2697 2420-2425
296. TLPAPA V4ZHY8 V4ZHY8_TOXGO AP2 domain transcription factor APVIIb-1/ADA2-B 2728 2451-2456
297. LPAPAG V4YYJ7 V4YYJ7_TOXGO Uncharacterized protein 333 2-7
298. LPAPAG V4ZPY4 V4ZPY4_TOXGO RAVE 1 carboxy-terminal protein 6665 4839-4844
299. PAPAGD B9PUI7 B9PUI7_TOXGO Uncharacterized protein 364 40-45
300. APAGDC Q1JSY4 Q1JSY4_TOXGO Uncharacterized protein 3344 625-630
301. APAGDC V4YR59 V4YR59_TOXGO Uncharacterized protein 1342 609-614
302. APAGDC V4Z1R2 V4Z1R2_TOXGO GCC2 and GCC3 domain-containing protein 5081 2394-2399
303. VHPGPV V4YWW3 V4YWW3_TOXGO AP2 domain transcription factor AP2X-2 1254 1209-1214
304. PGPVSP B9Q641 B9Q641_TOXGO Uncharacterized protein 1011 232-237
305. PGPVSP B9QEJ9 B9QEJ9_TOXGO Putative transmembrane protein 1300 793-798
306. PGPVSP Q6JD66 Q6JD66_TOXGO Eukaryotic initiation factor-2 alpha kinase-A 5072 3670-3675
307. PGPVSP V4Z893 V4Z893_TOXGO Uncharacterized protein 740 529-534
308. PGPVSP V4ZB93 V4ZB93_TOXGO Putative ATP-dependent hsl protease ATP-binding subunit hslU 672 382-387
309. PGPVSP V4ZU83 V4ZU83_TOXGO eIF2 kinase IF2K-A (Incomplete catalytic triad) 4638 3236-3241
310. GPVSPA B9QEJ9 B9QEJ9_TOXGO Putative transmembrane protein 1300 794-799
311. PVSPAR B9Q7N4 B9Q7N4_TOXGO Uncharacterized protein 154 88-93
312. PVSPAR V4ZPR1 V4ZPR1_TOXGO Putative transmembrane protein 2050 1450-1455
313. SPAREA B9Q591 B9Q591_TOXGO Putative transmembrane protein 7354 5490-5495
314. PAREAF V4Z498 V4Z498_TOXGO Uncharacterized protein 1765 1243-1248
315. PAREAF V4Z7C5 V4Z7C5_TOXGO Queuine tRNA ribosyl transferase 951 680-685
316. REAFYR V5AYN9 V5AYN9_TOXGO Cyclic nucleotide-binding domain-containing protein 2723 1138-1143
317. EAFYRH B9QHE7 B9QHE7_TOXGO Putative transmembrane protein 536 488-493
318. DFSFSP V5BFD3 V5BFD3_TOXGO Dipeptidyl peptidase iv (Dpp iv) n-terminal region domain-containing protein 1883 536-541
319. SFSPGG V5BMQ4 V5BMQ4_TOXGO AP2 domain transcription factor AP2VIII-7 870 479-484
320. SPGGYL V4YZI6 V4YZI6_TOXGO Putative tRNA synthetase 2557 44-49
321. VIALNR V5BAU3 V5BAU3_TOXGO Putative transmembrane protein 1011 918-923
322. RYSASL B9Q4Z1 B9Q4Z1_TOXGO Phospholipase, patatin family protein 2904 80-85
323. SASLQP B9Q7W7 B9Q7W7_TOXGO Putative collagen alpha-1(II) chain 1259 1230-1235
324. SASLQP V4ZIA3 V4ZIA3_TOXGO EF hand family protein 296 45-50
325. SASLQP V5BHA1 V5BHA1_TOXGO Uncharacterized protein 5632 2527-2532
326. ASLQPV V4YR23 V4YR23_TOXGO Non-specific serine/threonine protein kinase 855 583-588
327. ASLQPV V4Z2V1 V4Z2V1_TOXGO Fumble protein 1226 900-905
328. ASLQPV V4Z754 V4Z754_TOXGO AAA domain protein 2605 1051-1056
329. ASLQPV V5AY44 V5AY44_TOXGO Putative transmembrane protein 7450 6658-6663
330. PVVDSR B9QG15 B9QG15_TOXGO Aspartyl protease ASP1 619 445-450
331. LTVATL B6KP74 B6KP74_TOXGO Uncharacterized protein 160 12-17
332. LTVATL B9QPM2 B9QPM2_TOXGO 3'5'-cyclic nucleotide phosphodiesterase domain-containing protein 1085 470-475
333. TVATLE B9QDW5 B9QDW5_TOXGO AP2 domain transcription factor AP2IX-4 951 53-58
334. TVATLE V4Z9U4 V4Z9U4_TOXGO Surface antigen repeat-containing protein 6661 3335-3340
335. VATLEE V4Z9U4 V4Z9U4_TOXGO Surface antigen repeat-containing protein 6661 3336-3341
336. ATLEER B6K973 B6K973_TOXGO Zinc finger, C3HC4 type (RING finger) domain-containing protein 2190 1793-1798
337. PGTGGC V4ZHU4 V4ZHU4_TOXGO Splicing factor U2AF protein 770 759-764
338. GTGGCV B5AIC8 B5AIC8_TOXGO Oocyst wall protein OWP1 499 343-348
339. GTGGCV B6KH70 B6KH70_TOXGO Putative ubiquitin 216 99-104
340. GTGGCV Q6S5C5 Q6S5C5_TOXGO Putative oocyst wall protein COWP 499 343-348
341. GCVPNT V4YNH7 V4YNH7_TOXGO SAG-related sequence SRS16A 396 329-334
342. GCVPNT V5B1U2 V5B1U2_TOXGO SAG-related sequence SRS16B 398 327-332
343. VPNTVP V4Z5L5 V4Z5L5_TOXGO Uncharacterized protein 622 461-466
344. PCRRQS V4Z3P9 V4Z3P9_TOXGO Putative Tbc domain,related protein 3378 1534-1539
345. NHTFSS B6KRH0 B6KRH0_TOXGO Tetratricopeptide repeat-containing protein 1779 636-641
346. TFSSGD V4ZEQ3 V4ZEQ3_TOXGO Not1 amine-terminal domain, CCR4-NOT complex component 1754 987-992
347. SSGDVA V4ZI73 V4ZI73_TOXGO Uncharacterized protein 4519 2489-2494
348. SGDVAP B6KP07 B6KP07_TOXGO DnaJ C terminal region domain-containing protein 1519 856-861
349. SGDVAP Q1JSW6 Q1JSW6_TOXGO Uncharacterized protein 1064 856-861
350. SGDVAP V4ZI73 V4ZI73_TOXGO Uncharacterized protein 4519 2490-2495
351. PYTKLC B9Q562 B9Q562_TOXGO Non-specific serine/threonine protein kinase 8428 7110-7115
352. ILKKLA V4ZWZ5 V4ZWZ5_TOXGO 'chromo' (CHRromatin Organization MOdifier) domain-containing protein 1408 688-693
353. LKKLAR B9PRV7 B9PRV7_TOXGO Zn-finger in Ran binding protein and others domain-containing protein 1258 82-87
354. KKLARV V4ZL97 V4ZL97_TOXGO Uncharacterized protein 7954 5166-5171
355. LARVVK V4ZML5 V4ZML5_TOXGO SAG-related sequence SRS38D 389 24-29
356. KHGKRV B6KP49 B6KP49_TOXGO Putative capping alpha-like subunit 424 344-349
357. KHGKRV Q1JSR5 Q1JSR5_TOXGO Capping protein alpha-like subunit 424 344-349
358. KHGKRV Q5XP13 Q5XP13_TOXGO Capping protein alpha-like subunit 424 344-349
359. KRVRGV B9PGJ4 B9PGJ4_TOXGO 18S rRNA biogenesis protein RCL1 protein 442 208-213
360. RVRGVW B9Q4U1 B9Q4U1_TOXGO Amine-terminal region of chorein, A TM vesicle-mediated sorter 10329 7591-7596
361. EERSEI V4ZE20 V4ZE20_TOXGO AP2 domain transcription factor AP2V-2 3456 684-689
362. SEIVDF V5AYN9 V5AYN9_TOXGO Cyclic nucleotide-binding domain-containing protein 2723 2125-2130
363. SEIVDF V5BFJ6 V5BFJ6_TOXGO Putative transmembrane protein 752 268-273
364. EIVDFS V4Z308 V4Z308_TOXGO Uncharacterized protein 2775 550-555
365. EIVDFS V5BFJ6 V5BFJ6_TOXGO Putative transmembrane protein 752 269-274
366. VMVARS V4ZHF6 V4ZHF6_TOXGO Uncharacterized protein 772 361-366
367. AFLEPY V4Z106 V4Z106_TOXGO Uncharacterized protein 2597 1648-1653
368. VVAITV V5B4P3 V5B4P3_TOXGO NifU family domain-containing protein 370 321-326
369. LTRGKK V5BCR0 V5BCR0_TOXGO Isovaleryl-CoA dehydrogenase 404 207-212
370. TRGKKS Q1JSY4 Q1JSY4_TOXGO Uncharacterized protein 3344 2610-2615
371. TRGKKS V4ZKP9 V4ZKP9_TOXGO Uncharacterized protein 320 136-141
372. TRGKKS V5BKV1 V5BKV1_TOXGO Protein phosphatase 2C domain-containing protein 4071 1923-1928
373. RGKKSG V4Z2U4 V4Z2U4_TOXGO Uncharacterized protein 1386 634-639
374. GKKSGG B6KB91 B6KB91_TOXGO Putative RNA recognition motif protein 578 427-432
375. GKKSGG V4Z2U4 V4Z2U4_TOXGO Uncharacterized protein 1386 635-640
376. GKKSGG V4Z7D4 V4Z7D4_TOXGO Uncharacterized protein 389 52-57
377. GKKSGG V4ZIJ3 V4ZIJ3_TOXGO Ulp1 protease family, C-terminal catalytic domain-containing protein 3028 1135-1140
378. GKKSGG V4ZN07 V4ZN07_TOXGO DNA topoisomerase 2 1657 26-31
379. GKKSGG V5B996 V5B996_TOXGO Endonuclease/exonuclease/phosphatase family protein 1558 1131-1136
380. SGGPAF B9QQU3 B9QQU3_TOXGO Putative transmembrane protein 2122 1695-1700
381. LLWALV B9QNR2 B9QNR2_TOXGO Uncharacterized protein 3029 2511-2516
382. LLWALV V4Z3C9 V4Z3C9_TOXGO Uncharacterized protein 1899 1328-1333
383. ENPRGT Q1JSH3 Q1JSH3_TOXGO Uncharacterized protein precursor 1012 729-734
384. ENPRGT V4Z222 V4Z222_TOXGO RNA-directed DNA polymerase 4625 4347-4352
385. ENPRGT V5BAX7 V5BAX7_TOXGO Uncharacterized protein 1633 857-862
386. DTVSGL V4Z4M8 V4Z4M8_TOXGO Amine-terminal region of chorein, A TM vesicle-mediated sorter 13455 4495-4500
387. TVSGLS V4YVM3 V4YVM3_TOXGO Uncharacterized protein 2336 1112-1117
388. RSVEDA V4Z4F5 V4Z4F5_TOXGO Uncharacterized protein 2203 1486-1491
389. RSVEDA V4Z9U4 V4Z9U4_TOXGO Surface antigen repeat-containing protein 6661 4637-4642
390. RSVEDA V4ZS58 V4ZS58_TOXGO Uncharacterized protein 121 54-59
391. AGKDEG V4Z7J4 V4Z7J4_TOXGO Ras-associated protein Rap1 isoform 1 family protein 761 259-264
392. AGKDEG V4ZMH1 V4ZMH1_TOXGO Uncharacterized protein 4312 2652-2657
393. KRAIDL V4Z4I1 V4Z4I1_TOXGO PLU-1 family protein 8088 1636-1641
394. LGDGET V4ZKG2 V4ZKG2_TOXGO Uncharacterized protein 2258 625-630
395. LGDGET V5AYU8 V5AYU8_TOXGO Uncharacterized protein 207 109-114
396. GDGETQ V4Z5J8 V4Z5J8_TOXGO Condensin complex subunit 1 2466 2078-2083
397. GDGETQ V4Z882 V4Z882_TOXGO Putative helicase 1355 802-807
398. GDGETQ V4ZHD6 V4ZHD6_TOXGO Putative oxidoreductase 503 117-122
399. DGETQK B6KBH4 B6KBH4_TOXGO Nucleoporin autopeptidase 2894 2359-2364
400. GLALLV V4Z7D1 V4Z7D1_TOXGO Putative ARM repeat protein 518 354-359
401. GLALLV V5BH90 V5BH90_TOXGO Diaminopimelate decarboxylase 686 601-606
402. LALLVF V4YXY6 V4YXY6_TOXGO GCC2 and protein GCC3 921 19-24
403. LALLVF V4ZIN3 V4ZIN3_TOXGO Uncharacterized protein 2277 570-575
404. ALLVFA V5BE57 V5BE57_TOXGO Chloride transporter, chloride channel (ClC) family protein 2052 273-278
405. LVFAWE V5BL77 V5BL77_TOXGO Inorganic anion transporter, sulfate permease (SulP) family protein 1497 1215-1220
406. PNSSQL V5B468 V5B468_TOXGO Uncharacterized protein 2811 1558-1563
407. NSSQLD V4YRP9 V4YRP9_TOXGO Peptidase M16 inactive domain-containing protein 1692 1303-1308
408. SSQLDF V5AZ15 V5AZ15_TOXGO Amine-terminal region of chorein, A TM vesicle-mediated sorter 8847 23-28
409. LDFLLA A4L9T5 A4L9T5_TOXGO Seryl-tRNA synthase 2 918 633-638
410. LDFLLA B9Q4U1 B9Q4U1_TOXGO Amine-terminal region of chorein, A TM vesicle-mediated sorter 10329 7242-7247
411. LDFLLA B9QH98 B9QH98_TOXGO Uncharacterized protein 1410 1143-1148
412. LDFLLA V4ZM20 V4ZM20_TOXGO Serine--tRNA ligase 559 265-270
413. LDFLLA V5BLD5 V5BLD5_TOXGO Adaptin c-terminal domain-containing protein 1672 1513-1518
414. FLLAFS B9QPB2 B9QPB2_TOXGO 3'5'-cyclic nucleotide phosphodiesterase domain-containing protein 1065 214-219
415. FLLAFS V4YT35 V4YT35_TOXGO Protein kinase (Incomplete catalytic triad) 1611 534-539
416. FLLAFS V5BK29 V5BK29_TOXGO Transporter, major facilitator family protein 847 386-391
417. LLAFSR V4ZIS0 V4ZIS0_TOXGO Putative transmembrane protein 850 737-742
418. LLAFSR V5BB23 V5BB23_TOXGO Uncharacterized protein 1094 271-276
419. FSRGIY V4ZPP0 V4ZPP0_TOXGO Transaldolase 384 170-175
420. CFSGVQ V4Z4Q0 V4Z4Q0_TOXGO Uncharacterized protein 864 312-317
421. SGVQSL V4ZCV3 V4ZCV3_TOXGO Amine-terminal region of chorein, A TM vesicle-mediated sorter 9741 8991-8996
422. VQSLAS V4ZBS7 V4ZBS7_TOXGO B-block-binding subunit of tfiiic protein 4096 1644-1649
423. QSLASP Q1PCQ8 Q1PCQ8_TOXGO Mitochondrial type I phosphatidylserine decarboxylase 427 203-208
424. QSLASP V4ZB70 V4ZB70_TOXGO Phosphatidylserine decarboxylase 427 203-208
425. SLASPP B6K8H8 B6K8H8_TOXGO Transporter, cation channel family protein 1515 11-16
426. SLASPP B6KAC8 B6KAC8_TOXGO Heat shock factor binding protein 1 protein 188 109-114
427. SLASPP B6KJI1 B6KJI1_TOXGO Arv1, related protein 859 398-403
428. SLASPP B9Q843 B9Q843_TOXGO Histone lysine methyltransferase SET1 7555 984-989
429. SLASPP B9QH66 B9QH66_TOXGO Uncharacterized protein 1346 474-479
430. SLASPP B9QLB6 B9QLB6_TOXGO Putative transmembrane protein 124 81-86
431. SLASPP B9QPR3 B9QPR3_TOXGO SWI2/SNF2 SRCAP/Ino80 2924 2412-2417
432. SLASPP Q7Z2C2 Q7Z2C2_TOXGO Snf2-related chromatin remodeling factor SRCAP 2924 2412-2417
433. SLASPP V4YQJ6 V4YQJ6_TOXGO Uncharacterized protein 2197 270-275
434. SLASPP V4Z308 V4Z308_TOXGO Uncharacterized protein 2775 1893-1898
435. SLASPP V4Z3K3 V4Z3K3_TOXGO PIK3R4 kinase-related protein (Incomplete catalytic triad) 3028 739-744
436. SLASPP V4Z5Y9 V4Z5Y9_TOXGO Putative transmembrane protein 635 289-294
437. SLASPP V4Z6T1 V4Z6T1_TOXGO Dopey, N-terminal domain-containing protein 3103 2382-2387
438. SLASPP V4ZER7 V4ZER7_TOXGO Uncharacterized protein 3307 1503-1508
439. SLASPP V4ZKD8 V4ZKD8_TOXGO Uncharacterized protein 11926 7238-7243
440. SLASPP V4ZMH1 V4ZMH1_TOXGO Uncharacterized protein 4312 2194-2199
441. SLASPP V4ZVH8 V4ZVH8_TOXGO Uncharacterized protein 1104 305-310
442. SLASPP V4ZVK3 V4ZVK3_TOXGO Uncharacterized protein 2214 487-492
443. SLASPP V5B2M2 V5B2M2_TOXGO Uncharacterized protein 1373 1127-1132
444. LASPPR B6KB97 B6KB97_TOXGO Uncharacterized protein 1714 1691-1696
445. LASPPR V4Z4K9 V4Z4K9_TOXGO Putative nucleoporin FG repeat region protein 5638 1280-1285
446. LASPPR V4ZDH1 V4ZDH1_TOXGO Putative transmembrane protein 669 222-227
447. LASPPR V4ZMF4 V4ZMF4_TOXGO Uncharacterized protein 1585 119-124
448. LASPPR V5AY00 V5AY00_TOXGO Uncharacterized protein 357 59-64
449. ASPPRQ V4Z581 V4Z581_TOXGO Protein SEY1 homolog 893 835-840
450. SPPRQA B9QI88 B9QI88_TOXGO Putative alpha-tubulin N-acetyltransferase 907 522-527
451. SPPRQA V4ZM77 V4ZM77_TOXGO Putative transmembrane protein 1060 756-761
452. PPRQAS B9QI88 B9QI88_TOXGO Putative alpha-tubulin N-acetyltransferase 907 523-528
453. PRQASP B9QAD0 B9QAD0_TOXGO tRNA methyl transferase 1596 25-30
454. PRQASP V4Z581 V4Z581_TOXGO Protein SEY1 homolog 893 881-886
455. QASPDL V4ZKL1 V4ZKL1_TOXGO NLI interacting factor family phosphatase 1624 336-341
456. SPDLTA B9QCW2 B9QCW2_TOXGO Uncharacterized protein 754 357-362
457. PDLTAS V4Z3Y5 V4Z3Y5_TOXGO TBC domain-containing protein 3409 685-690
458. LTASSA B9Q4W9 B9Q4W9_TOXGO Uncharacterized protein 1232 385-390
459. LTASSA M9T1W1 M9T1W1_TOXGO ATG4 3753 569-574
460. LTASSA V4ZM71 V4ZM71_TOXGO Putative autophagy-related cysteine peptidase atg4 3747 569-574
461. TASSAQ B9QRA7 B9QRA7_TOXGO Enoyl-CoA hydratase/isomerase family protein 371 106-111
462. TASSAQ V4ZFZ2 V4ZFZ2_TOXGO Sec7 domain-containing protein 3546 961-966
463. ASSAQA B6KH74 B6KH74_TOXGO Nucleoporin complex subunit 54 346 32-37
464. ASSAQA V4ZCK5 V4ZCK5_TOXGO Uncharacterized protein 1023 907-912
465. ASSAQA V4ZKN4 V4ZKN4_TOXGO Putative prenylcysteine lyase protein 719 642-647
466. ASSAQA V5BBU1 V5BBU1_TOXGO Carrier superfamily protein 682 340-345
467. SSAQAS B6KGY9 B6KGY9_TOXGO Uncharacterized protein 2303 2099-2104
468. SSAQAS B9Q479 B9Q479_TOXGO WD domain, G-beta repeat-containing protein 3633 1595-1600
469. SSAQAS Q1JT09 Q1JT09_TOXGO Putative uncharacterized protein precursor 2213 359-364
470. SSAQAS V4Z0Q7 V4Z0Q7_TOXGO Uncharacterized protein 930 266-271
471. SSAQAS V4Z9X5 V4Z9X5_TOXGO Uncharacterized protein 4983 715-720
472. SSAQAS V4ZA72 V4ZA72_TOXGO Uncharacterized protein 2746 348-353
473. SAQASV B9QMH6 B9QMH6_TOXGO RNA pseudouridine synthase superfamily protein 6535 3823-3828
474. SAQASV V4YPD7 V4YPD7_TOXGO Uncharacterized protein 875 177-182
475. SAQASV V4ZNM3 V4ZNM3_TOXGO NEK kinase 4925 4126-4131
476. AQASVL V4ZTB4 V4ZTB4_TOXGO ULK kinase 2097 2041-2046
477. KMLQAA B9PMC6 B9PMC6_TOXGO Putative transmembrane protein 691 547-552
478. LQAARD B9QFP6 B9QFP6_TOXGO Rad17 cell cycle checkpoint protein 1867 1783-1788
479. LQAARD V5B2X1 V5B2X1_TOXGO Uncharacterized protein 4035 1977-1982
480. QAARDM B9QFP6 B9QFP6_TOXGO Rad17 cell cycle checkpoint protein 1867 1784-1789
481. VTTAGV B9PZ51 B9PZ51_TOXGO Toxoplasma gondii family B protein 150 87-92
482. TTAGVS B9QD38 B9QD38_TOXGO NudC family protein 384 138-143
483. TTAGVS V4YXI5 V4YXI5_TOXGO Uncharacterized protein 1320 286-291
484. TTAGVS V4Z8G0 V4Z8G0_TOXGO Guanylyl cyclase 4367 3852-3857
485. TTAGVS V5BM58 V5BM58_TOXGO AP2 domain transcription factor AP2VIII-6 1763 146-151
486. TAGVSS B9PYE4 B9PYE4_TOXGO Putative type I fatty acid synthase 10021 3485-3490
487. TAGVSS Q1JTE1 Q1JTE1_TOXGO Type I fatty acid synthase, putative 9940 3404-3409
488. AGVSSS B6KQK7 B6KQK7_TOXGO Cyclin protein 2572 1801-1806
489. AGVSSS B9Q8D8 B9Q8D8_TOXGO Chloroquine resistance marker 3946 286-291
490. AGVSSS Q1JT27 Q1JT27_TOXGO Putative uncharacterized protein 2626 1855-1860
491. AGVSSS V4YXZ8 V4YXZ8_TOXGO Uncharacterized protein 192 130-135
492. AGVSSS V4ZL97 V4ZL97_TOXGO Uncharacterized protein 7954 6691-6696
493. AGVSSS V4ZMG2 V4ZMG2_TOXGO AP2 domain transcription factor AP2XI-3 1399 92-97
494. AGVSSS V4ZNG2 V4ZNG2_TOXGO E1-E2 ATPase subfamily protein 2014 689-694
495. AGVSSS V4ZPZ2 V4ZPZ2_TOXGO ARID/BRIGHT DNA binding domain-containing protein 2713 653-658
496. AGVSSS V4ZWD1 V4ZWD1_TOXGO Putative transmembrane protein 410 233-238
497. GVSSSL B6KF96 B6KF96_TOXGO Putative transmembrane protein 1261 119-124
498. GVSSSL B9QJ14 B9QJ14_TOXGO Uncharacterized protein 887 359-364
499. GVSSSL V4YN30 V4YN30_TOXGO Putative vacuolar protein sorting-associated protein 8650 268-273, 2392-2397
500. GVSSSL V4ZD37 V4ZD37_TOXGO eIF2 kinase IF2K-C 3270 262-267
501. GVSSSL X2F4W3 X2F4W3_TOXGO GCN2-like eIF2 alpha kinase 3269 262-267
502. VSSSLD B6KR35 B6KR35_TOXGO Phospholipase, patatin family protein 2579 128-133
503. VSSSLD B9QBS0 B9QBS0_TOXGO WD domain, G-beta repeat-containing protein 1017 739-744
504. VSSSLD Q1JTK4 Q1JTK4_TOXGO Putative uncharacterized protein 834 68-73
505. VSSSLD V4YT41 V4YT41_TOXGO Protein kinase domain protein 6052 2546-2551
506. VSSSLD V4Z952 V4Z952_TOXGO Uncharacterized protein 4210 3796-3801
507. VSSSLD V4ZC01 V4ZC01_TOXGO Uncharacterized protein 563 39-44
508. VSSSLD V4ZQG9 V4ZQG9_TOXGO Uncharacterized protein 3806 2788-2793
509. VSSSLD V4ZRG6 V4ZRG6_TOXGO SWI2/SNF2-containing protein 1224 345-350
510. VSSSLD V4ZRH2 V4ZRH2_TOXGO Zinc carboxypeptidase superfamily protein 1330 158-163
511. SSSLDR V4YTX2 V4YTX2_TOXGO Uncharacterized protein 2251 1163-1168
512. SSSLDR V4ZGU8 V4ZGU8_TOXGO Zinc finger in N-recognin protein 4383 3342-3347
513. SSSLDR V4ZKE7 V4ZKE7_TOXGO AP2 domain transcription factor AP2X-1 1292 909-914
514. SSSLDR V4ZKZ1 V4ZKZ1_TOXGO Uncharacterized protein 1477 65-70
515. SSSLDR V4ZQG9 V4ZQG9_TOXGO Uncharacterized protein 3806 2789-2794
516. SSLDRA B6KAV9 B6KAV9_TOXGO FYVE zinc finger domain-containing protein 834 669-674
517. SSLDRA V5B444 V5B444_TOXGO Uncharacterized protein 2574 383-388
518. SLDRAT V4YYM9 V4YYM9_TOXGO Putative transmembrane protein 1823 978-983
519. LDRATR B9Q8F0 B9Q8F0_TOXGO Uncharacterized protein 1255 1184-1189
520. LDRATR V4ZMR4 V4ZMR4_TOXGO Uncharacterized protein 636 520-525
521. WGGGRR V4YZY7 V4YZY7_TOXGO ATP-dependent DNA helicase, RecQ family protein 1759 1417-1422
522. GGGRRA B9Q6Q1 B9Q6Q1_TOXGO WD-40 repeat protein 702 480-485
523. GGGRRA B9QEY3 B9QEY3_TOXGO Signal recognition particle receptor beta subunit protein 347 125-130
524. GGGRRA V4YY84 V4YY84_TOXGO Ribosomal RNA methyltransferase (FtsJ ) family protein 482 239-244
525. GGGRRA V4ZAN5 V4ZAN5_TOXGO Serine/threonine specific protein phosphatase 2462 337-342
526. GGGRRA V4ZHN3 V4ZHN3_TOXGO Uncharacterized protein 292 56-61
527. GGGRRA V4ZL39 V4ZL39_TOXGO Zn-finger in ubiquitin-hydrolases domain-containing protein 2582 310-315
528. GGGRRA V4ZSE4 V4ZSE4_TOXGO WD domain, G-beta repeat-containing protein 4664 4379-4384
529. GGGRRA V5BI24 V5BI24_TOXGO Putative DnaJ family chaperone 426 112-117
530. GGRRAP V5B6P7 V5B6P7_TOXGO Regulator of chromosome condensation (RCC1) repeat-containing protein 2900 910-915
531. GRRAPP B6K9Z6 B6K9Z6_TOXGO Alpha and gamma adaptin-binding protein p34 784 376-381
532. GRRAPP V5B115 V5B115_TOXGO Ubiquitin-like protein ATG12 681 234-239
533. GRRAPP V5BM48 V5BM48_TOXGO Uncharacterized protein 1186 282-287
534. RAPPPS B9QPV5 B9QPV5_TOXGO tRNA ligases class II (D, K and N) domain-containing protein 1170 843-848
535. RAPPPS Q45W11 Q45W11_TOXGO Putative ATP-binding cassette protein 307 2-7
536. RAPPPS V4Z4D5 V4Z4D5_TOXGO RNA recognition motif-containing protein 1123 587-592
537. RAPPPS V4ZGX1 V4ZGX1_TOXGO Uncharacterized protein 3917 2345-2350
538. APPPSP B6KFB3 B6KFB3_TOXGO Macro domain-containing protein 817 610-615
539. APPPSP B9Q5J0 B9Q5J0_TOXGO Uncharacterized protein 2385 811-816
540. APPPSP B9QEV3 B9QEV3_TOXGO Zinc finger, C3HC4 type (RING finger) domain-containing protein 3872 994-999
541. APPPSP D2K747 D2K747_TOXGO Proteophosphoglycan 1522 3-8
542. APPPSP Q1JSF3 Q1JSF3_TOXGO Uncharacterized protein 4600 2816-2821
543. APPPSP V4YNX0 V4YNX0_TOXGO Proteophosphoglycan PPG1 1531 3-8
544. APPPSP V4Z6H5 V4Z6H5_TOXGO Putative glutamic acid-rcih protein 4436 2341-2346
545. APPPSP V4ZEA4 V4ZEA4_TOXGO Uncharacterized protein 2622 1265-1270
546. APPPSP V4ZGE2 V4ZGE2_TOXGO Aurora kinase 2811 755-760
547. APPPSP V4ZGX1 V4ZGX1_TOXGO Uncharacterized protein 3917 2346-2351
548. APPPSP V4ZHB1 V4ZHB1_TOXGO ICE family protease (Caspase) p20 domain-containing protein 2055 257-262
549. APPPSP V4ZHN1 V4ZHN1_TOXGO Uncharacterized protein 6085 4483-4488
550. PPPSPC V5B2X1 V5B2X1_TOXGO Uncharacterized protein 4035 2332-2337
551. PPSPCP V4ZL32 V4ZL32_TOXGO Uncharacterized protein 1689 529-534
552. PPSPCP V5BKN7 V5BKN7_TOXGO Putative GCN1 3416 2962-2967
553. PSPCPT V4Z200 V4Z200_TOXGO Uncharacterized protein 1380 86-91
554. SPCPTP V5AXG1 V5AXG1_TOXGO DNA polymerase I domain-containing protein 1657 280-285
555. PCPTPR V4ZJN0 V4ZJN0_TOXGO MCM2/3/5 family protein 1238 390-395
556. PTPRSG B9PM57 B9PM57_TOXGO Alveolin domain containing intermediate filament IMC5 584 326-331
557. PTPRSG Q0GB78 Q0GB78_TOXGO Inner membrane complex associated protein 4 584 326-331
558. PTPRSG V4YQ73 V4YQ73_TOXGO MIF4G domain-containing protein 3756 1465-1470
559. PTPRSG V4YUV5 V4YUV5_TOXGO AP2 domain transcription factor AP2VIIb-2 810 530-535
560. PTPRSG V4ZNM3 V4ZNM3_TOXGO NEK kinase 4925 1929-1934
561. TPRSGP B9PM57 B9PM57_TOXGO Alveolin domain containing intermediate filament IMC5 584 327-332
562. TPRSGP Q0GB78 Q0GB78_TOXGO Inner membrane complex associated protein 4 584 327-332
563. TPRSGP V4ZCD5 V4ZCD5_TOXGO FHA domain-containing protein 1042 910-915
564. PRSGPS B9PM57 B9PM57_TOXGO Alveolin domain containing intermediate filament IMC5 584 328-333
565. PRSGPS B9PY91 B9PY91_TOXGO Putative transmembrane protein 603 122-127
566. PRSGPS B9QN11 B9QN11_TOXGO Histone arginine methyltransferase PRMT5 979 839-844
567. PRSGPS Q0GB78 Q0GB78_TOXGO Inner membrane complex associated protein 4 584 328-333
568. PRSGPS V4ZCQ1 V4ZCQ1_TOXGO CW-type Zinc Finger protein 1296 719-724
569. RSGPSP B6K9V8 B6K9V8_TOXGO RNA pseudouridine synthase superfamily protein 2780 2687-2692
570. RSGPSP B9PM57 B9PM57_TOXGO Alveolin domain containing intermediate filament IMC5 584 329-334
571. RSGPSP Q0GB78 Q0GB78_TOXGO Inner membrane complex associated protein 4 584 329-334
572. RSGPSP V4Z6U9 V4Z6U9_TOXGO Phosphoglycerate mutase family protein 1971 531-536
573. RSGPSP V4ZJD5 V4ZJD5_TOXGO Transporter 1577 1207-1212
574. RSGPSP V5AWD3 V5AWD3_TOXGO Toxoplasma gondii family E protein 362 312-317
575. RSGPSP V5BKJ8 V5BKJ8_TOXGO Suppressor of forked protein SUF 1295 1032-1037
576. PSPCLP Q1JSM5 Q1JSM5_TOXGO Ubiquitin-transferase, putative precursor 12269 7418-7423
577. PSPCLP V4Z1Z4 V4Z1Z4_TOXGO HECT-domain (Ubiquitin-transferase) domain-containing protein 12299 7474-7479
578. SPCLPT V4ZEW9 V4ZEW9_TOXGO CorA family Mg2+ transporter protein 1190 168-173
579. PTPDPP V4YN30 V4YN30_TOXGO Putative vacuolar protein sorting-associated protein 8650 7786-7791
580. DPPPEP B6KSU6 B6KSU6_TOXGO Cholinephosphate cytidylyltransferase 329 24-29
581. PPPEPS V4ZVZ6 V4ZVZ6_TOXGO Uncharacterized protein 2022 322-327
582. PPPEPS V5AYY3 V5AYY3_TOXGO Variable surface lipoprotein 876 194-199
583. PEPSPT V4Z6Z0 V4Z6Z0_TOXGO Uncharacterized protein 4064 3367-3372
584. GPPDGG B9QI99 B9QI99_TOXGO Kinesin motor domain-containing protein 1317 150-155
585. PPDGGR B9QI99 B9QI99_TOXGO Kinesin motor domain-containing protein 1317 151-156
586. PPDGGR V4ZNH6 V4ZNH6_TOXGO Putative aquarius 2250 12-17
587. PDGGRA V4Z5G1 V4Z5G1_TOXGO Putative, related protein 1658 293-298
588. DGGRAA V4YJL9 V4YJL9_TOXGO Methionine aminopeptidase 1 484 458-463
589. DGGRAA V4Z5W6 V4Z5W6_TOXGO Putative transmembrane protein 1015 625-630
590. DGGRAA V4ZBB7 V4ZBB7_TOXGO Aurora kinase(Incomplete catalytic triad) 2470 1645-1650
591. DGGRAA V4ZQR4 V4ZQR4_TOXGO Radical SAM domain-containing protein 1166 223-228
592. DGGRAA V5BD56 V5BD56_TOXGO AP2 domain transcription factor AP2VIIa-2 918 575-580
593. DGGRAA V5BDT5 V5BDT5_TOXGO Phosphatidylinositol 3-and 4-kinase 8859 943-948
594. GGRAAL V4YLV1 V4YLV1_TOXGO PP-loop domain-containing protein 1701 1260-1265
595. GGRAAL V4Z3Y5 V4Z3Y5_TOXGO TBC domain-containing protein 3409 1047-1052
596. GGRAAL V4ZNQ0 V4ZNQ0_TOXGO Uncharacterized protein 5025 10-15
597. GGRAAL V5BGB9 V5BGB9_TOXGO SNARE associated Golgi protein 394 148-153
598. GRAALV V4Z3Y5 V4Z3Y5_TOXGO TBC domain-containing protein 3409 1048-1053
599. RAALVR V4ZAY6 V4ZAY6_TOXGO Spc97 / Spc98 family protein 2023 841-846
600. RAALVR V4ZQG3 V4ZQG3_TOXGO Uncharacterized protein 1179 300-305
601. AALVRR V4Z1Y1 V4Z1Y1_TOXGO Regulator of chromosome condensation (RCC1) repeat-containing protein 1761 1398-1403
602. AALVRR V4ZB45 V4ZB45_TOXGO Putative GTP-binding protein engA 1180 56-61
603. AALVRR V4ZQG3 V4ZQG3_TOXGO Uncharacterized protein 1179 301-306
604. ALVRRA V4ZQG3 V4ZQG3_TOXGO Uncharacterized protein 1179 302-307
605. ALVRRA V5BBF9 V5BBF9_TOXGO Uncharacterized protein 1172 293-298
606. ALVRRA V5BEU4 V5BEU4_TOXGO Putative proteophosphoglycan 5, related protein 1451 1423-1428
607. RRAPQP B9QNR9 B9QNR9_TOXGO Uncharacterized protein 1185 99-104
608. RAPQPP V4ZNP7 V4ZNP7_TOXGO Uncharacterized protein 817 513-518
609. APQPPG V4ZJ82 V4ZJ82_TOXGO Uncharacterized protein 1881 264-269
610. APQPPG V5B3X2 V5B3X2_TOXGO GATA zinc finger domain-containing protein 2012 1011-1016
611. PQPPGR V5B3X2 V5B3X2_TOXGO GATA zinc finger domain-containing protein 2012 1012-1017
612. PPGRPP B6K956 B6K956_TOXGO Sel1 repeat-containing protein 1012 436-441
613. PPGRPP B6KTA3 B6KTA3_TOXGO Formin 3 2849 1368-1373
614. PPGRPP B9PSE3 B9PSE3_TOXGO Zinc finger, c2h2 type domain-containing protein 287 243-248
615. PPGRPP V4ZEF9 V4ZEF9_TOXGO Formin FRM3 2847 1366-1371
616. PPGRPP V4ZFN8 V4ZFN8_TOXGO Putative DNA replication licensing factor 1049 449-454
617. PPTPGP V4ZCC7 V4ZCC7_TOXGO Uncharacterized protein 1414 11-16
618. PPTPGP V4ZNI0 V4ZNI0_TOXGO Putative transmembrane protein 6079 424-429
619. PTPGPP V4Z6C0 V4Z6C0_TOXGO LsmAD domain-containing protein 1524 794-799
620. PTPGPP V4ZMR4 V4ZMR4_TOXGO Uncharacterized protein 636 57-62
621. PTPGPP V4ZNI0 V4ZNI0_TOXGO Putative transmembrane protein 6079 425-430
622. TPGPPL V4YXI5 V4YXI5_TOXGO Uncharacterized protein 1320 873-878
623. TPGPPL V4ZIE0 V4ZIE0_TOXGO DNA polymerase 4247 550-555
624. TPGPPL V5B9B3 V5B9B3_TOXGO Uncharacterized protein 371 134-139
625. PPLSDV V4Z0S4 V4Z0S4_TOXGO Methionine aminopeptidase 2 480 447-452
626. PLSDVS V4YLH6 V4YLH6_TOXGO Uncharacterized protein 3421 2469-2474
627. PLSDVS V4YXQ4 V4YXQ4_TOXGO Ubiquitin carboxyl-terminal hydrolase 3144 1884-1889
628. LSDVSR V4Z3E2 V4Z3E2_TOXGO Uncharacterized protein 287 78-83
629. LSDVSR V5AW93 V5AW93_TOXGO Uncharacterized protein 1683 1084-1089
630. SDVSRV B9QPV7 B9QPV7_TOXGO Putative transmembrane protein 545 10-15
631. SDVSRV V4ZJY6 V4ZJY6_TOXGO Putative transmembrane protein 875 276-281
632. DVSRVS B6KHP1 B6KHP1_TOXGO Uncharacterized protein 1156 29-34
633. DVSRVS V5B2Z9 V5B2Z9_TOXGO Putative transmembrane protein 298 93-98
634. VSRVSR V4ZAL8 V4ZAL8_TOXGO Uncharacterized protein 1098 74-79
635. VSRVSR V4ZKG2 V4ZKG2_TOXGO Uncharacterized protein 2258 633-638
636. SRVSRR B9QE62 B9QE62_TOXGO Uncharacterized protein 152 72-77
637. SRVSRR V4Z3T5 V4Z3T5_TOXGO Putative LMBR1 family region protein 656 117-122
638. SRVSRR V4Z4P2 V4Z4P2_TOXGO Uncharacterized protein 498 56-61
639. SRVSRR V4ZAL8 V4ZAL8_TOXGO Uncharacterized protein 1098 75-80
640. SRVSRR V4ZLZ2 V4ZLZ2_TOXGO Hydrolase 3142 1680-1685
641. SRVSRR V4ZNQ0 V4ZNQ0_TOXGO Uncharacterized protein 5025 983-988
642. RVSRRP V4ZAL8 V4ZAL8_TOXGO Uncharacterized protein 1098 76-81
643. RVSRRP V4ZDF9 V4ZDF9_TOXGO Putative GTP-binding protein engB 1027 615-620
644. RVSRRP V4ZHE1 V4ZHE1_TOXGO ATG C terminal domain-containing protein 8079 932-937
645. RVSRRP V4ZLZ2 V4ZLZ2_TOXGO Hydrolase 3142 1681-1686
646. VSRRPA V4Z6W2 V4Z6W2_TOXGO BRCA1 C Terminus (BRCT) domain-containing protein 1322 484-489
647. VSRRPA V4ZAL8 V4ZAL8_TOXGO Uncharacterized protein 1098 77-82
648. VSRRPA V4ZLZ2 V4ZLZ2_TOXGO Hydrolase 3142 1682-1687
649. SRRPAW V5B602 V5B602_TOXGO Tetratricopeptide repeat-containing protein 1219 612-617
650. CGRHLS B9QJE8 B9QJE8_TOXGO Uncharacterized protein 3150 757-762
651. GRHLSA B9QIG4 B9QIG4_TOXGO Uncharacterized protein 771 332-337
652. GRHLSA V4Z550 V4Z550_TOXGO Uncharacterized protein 1008 215-220
653. RHLSAS D0V3Y0 D0V3Y0_TOXGO Formin 1 5051 3570-3575
654. RHLSAS V4YZ27 V4YZ27_TOXGO Formin FRM1 5048 3528-3533
655. RHLSAS V4ZK04 V4ZK04_TOXGO Tetratricopeptide repeat-containing protein 1677 133-138
656. HLSASE B9Q562 B9Q562_TOXGO Non-specific serine/threonine protein kinase 8428 250-255
657. HLSASE B9QHY1 B9QHY1_TOXGO Uncharacterized protein 2240 1856-1861
658. HLSASE V5B6F0 V5B6F0_TOXGO Uncharacterized protein 1397 859-864
659. LSASER V4YJS1 V4YJS1_TOXGO Phosphatidylinositol-4-phosphate 5-Kinase 4165 2120-2125
660. LSASER V4Z8F3 V4Z8F3_TOXGO Uncharacterized protein 834 152-157
661. LSASER V4ZJE5 V4ZJE5_TOXGO Uncharacterized protein 914 619-624
662. SASERP Q1JTL0 Q1JTL0_TOXGO Putative uncharacterized protein 529 424-429
663. SASERP V5AZJ8 V5AZJ8_TOXGO Vps54 family protein 1983 499-504
664. SERPLS Q1JSP4 Q1JSP4_TOXGO Uncharacterized protein 352 153-158
665. SERPLS V4YU53 V4YU53_TOXGO Uncharacterized protein 2302 298-303
666. SERPLS V5B0J2 V5B0J2_TOXGO DNA/RNA non-specific endonuclease 991 135-140
667. ERPLSP V4YNV3 V4YNV3_TOXGO Putative beta-tubulin cofactor D 2041 277-282
668. ERPLSP V4YU53 V4YU53_TOXGO Uncharacterized protein 2302 1976-1981
669. ERPLSP V4ZHB5 V4ZHB5_TOXGO Uncharacterized protein 1792 312-317
670. ERPLSP V4ZMT1 V4ZMT1_TOXGO Uncharacterized protein 1015 167-172
671. RPLSPA B9QEV3 B9QEV3_TOXGO Zinc finger, C3HC4 type (RING finger) domain-containing protein 3872 3107-3112
672. RPLSPA B9QPR3 B9QPR3_TOXGO SWI2/SNF2 SRCAP/Ino80 2924 1998-2003
673. RPLSPA Q7Z2C2 Q7Z2C2_TOXGO Snf2-related chromatin remodeling factor SRCAP 2924 1998-2003
674. RPLSPA V4YZ36 V4YZ36_TOXGO HEAT repeat-containing protein 3407 598-603
675. RPLSPA V4Z3V0 V4Z3V0_TOXGO Uncharacterized protein 100 44-49
676. RPLSPA V4Z8G0 V4Z8G0_TOXGO Guanylyl cyclase 4367 1447-1452
677. RPLSPA V4ZHB5 V4ZHB5_TOXGO Uncharacterized protein 1792 313-318
678. RPLSPA V4ZL97 V4ZL97_TOXGO Uncharacterized protein 7954 5242-5247
679. RPLSPA V4ZPH3 V4ZPH3_TOXGO Protein kinase domain protein 2329 624-629
680. RPLSPA V4ZQ23 V4ZQ23_TOXGO Uncharacterized protein 932 259-264
681. PLSPAR V4Z3V0 V4Z3V0_TOXGO Uncharacterized protein 100 45-50
682. PLSPAR V4ZAW5 V4ZAW5_TOXGO Uncharacterized protein 847 647-652
683. PLSPAR V4ZL97 V4ZL97_TOXGO Uncharacterized protein 7954 5243-5248
684. PLSPAR V5AWP5 V5AWP5_TOXGO HEAT repeat-containing protein 1766 854-859
685. SSFPRA B9PI69 B9PI69_TOXGO NPL4 family protein 502 418-423
686. SSFPRA V4Z2W7 V4Z2W7_TOXGO Uncharacterized protein 1027 101-106
687. FPRADR B6KJV5 B6KJV5_TOXGO Glutamate-cysteine ligase, catalytic subunit domain-containing protein 1062 325-330
688. DRSGRP V4ZDP7 V4ZDP7_TOXGO Uncharacterized protein 103 4-9
689. RSGRPF B6KFV0 B6KFV0_TOXGO Uncharacterized protein 1266 331-336
690. RSGRPF V4ZIW9 V4ZIW9_TOXGO Putative transmembrane protein 330 177-182
691. RPFLPL V4Z509 V4Z509_TOXGO Putative rhoptry protein 1063 269-274
692. RPFLPL V4Z7L4 V4Z7L4_TOXGO FAD binding domain-containing protein 1390 1140-1145
693. PFLPLF B6KNY8 B6KNY8_TOXGO Putative transmembrane protein 667 653-658
694. PFLPLF V4Z7L4 V4Z7L4_TOXGO FAD binding domain-containing protein 1390 1141-1146
695. FLPLFP B6KB03 B6KB03_TOXGO Transporter, major facilitator family protein 504 326-331
696. FLPLFP V5BHK7 V5BHK7_TOXGO Oxidoreductase, short chain dehydrogenase/reductase family protein 837 71-76
697. FLPLFP V5BM64 V5BM64_TOXGO Putative cysteine protease domain protein 347 338-343
698. ELEDLP V4ZJ30 V4ZJ30_TOXGO Uncharacterized protein 1104 933-938
699. LEDLPL B9QHV4 B9QHV4_TOXGO Uncharacterized protein 430 289-294
700. EDLPLL V4YIZ5 V4YIZ5_TOXGO Uncharacterized protein 903 802-807
701. DLPLLG B6K8K9 B6K8K9_TOXGO Putative M phase phosphoprotein MPP10 866 571-576
702. DLPLLG V5BM33 V5BM33_TOXGO GAF domain-containing protein 826 789-794
703. LPLLGP V4ZC41 V4ZC41_TOXGO Uncharacterized protein 496 409-414
704. LPLLGP V4ZJH2 V4ZJH2_TOXGO RNA recognition motif-containing protein 2070 1261-1266
705. PLLGPE B6K9S7 B6K9S7_TOXGO Uncharacterized protein 243 166-171
706. PLLGPE B9Q3H3 B9Q3H3_TOXGO Uncharacterized protein 1569 315-320
707. PLLGPE V4ZEK9 V4ZEK9_TOXGO Putative transmembrane protein 579 387-392
708. PLLGPE V4ZJH2 V4ZJH2_TOXGO RNA recognition motif-containing protein 2070 1262-1267
709. EQLARR B9PQV4 B9PQV4_TOXGO Putative transporter 728 399-404
710. EQLARR B9QFY0 B9QFY0_TOXGO Probable tRNA N6-adenosine threonylcarbamoyltransferase 580 209-214
711. QLARRE V4ZBG2 V4ZBG2_TOXGO 3'5'-cyclic nucleotide phosphodiesterase domain-containing protein 3476 944-949
712. QLARRE V5B4Q4 V5B4Q4_TOXGO Uncharacterized protein 1046 669-674
713. LARREA B6KJR9 B6KJR9_TOXGO SWIB/MDM2 domain-containing protein 1073 849-854
714. LARREA B6KR20 B6KR20_TOXGO Guanylate-binding protein, N-terminal domain-containing protein 1281 1046-1051
715. LARREA Q1JTH3 Q1JTH3_TOXGO Putative uncharacterized protein 429 102-107
716. LARREA V4YZC1 V4YZC1_TOXGO Uncharacterized protein 3026 1120-1125
717. LARREA V4Z108 V4Z108_TOXGO EKN1, related protein 806 102-107
718. LARREA V4ZNI0 V4ZNI0_TOXGO Putative transmembrane protein 6079 2679-2684
719. ARREAL B6KR20 B6KR20_TOXGO Guanylate-binding protein, N-terminal domain-containing protein 1281 1047-1052
720. ARREAL B9QFN3 B9QFN3_TOXGO CRAL/TRIO domain-containing protein 456 417-422
721. ARREAL V4Z308 V4Z308_TOXGO Uncharacterized protein 2775 2076-2081
722. ARREAL V5B7V4 V5B7V4_TOXGO tRNA pseudouridine synthase D 1489 446-451
723. ARREAL V5BG65 V5BG65_TOXGO Uncharacterized protein 601 208-213
724. RREALL B9QFI6 B9QFI6_TOXGO Uncharacterized protein 495 177-182
725. RREALL V4Z308 V4Z308_TOXGO Uncharacterized protein 2775 2077-2082
726. RREALL V4ZDF0 V4ZDF0_TOXGO AP2 domain transcription factor AP2III-3 2330 1410-1415
727. RREALL V5B5U5 V5B5U5_TOXGO Putative alpha-glucan water dikinase 1 1552 698-703
728. RREALL V5B7V4 V5B7V4_TOXGO tRNA pseudouridine synthase D 1489 447-452
729. REALLH V4Z752 V4Z752_TOXGO Uncharacterized protein 202 10-15
730. ALLHAA B6KAG9 B6KAG9_TOXGO U3 small nucleolar RNA-associated protein 10 3738 780-785
731. ALLHAA V4ZD18 V4ZD18_TOXGO Uncharacterized protein 3221 2999-3004
732. ALLHAA V5B3Z0 V5B3Z0_TOXGO FATC domain-containing protein 6012 2594-2599
733. LHAAWA Q1JT92 Q1JT92_TOXGO Putative uncharacterized protein 479 242-247
734. LHAAWA V4Z1E0 V4Z1E0_TOXGO Enoyl-CoA hydratase/isomerase family protein 716 479-484
735. HAAWAR Q1JT92 Q1JT92_TOXGO Putative uncharacterized protein 479 243-248
736. HAAWAR V4Z1E0 V4Z1E0_TOXGO Enoyl-CoA hydratase/isomerase family protein 716 480-485
737. ARGSRP V4ZT82 V4ZT82_TOXGO Uncharacterized protein 3407 1683-1688
738. RGSRPR V5AXG1 V5AXG1_TOXGO DNA polymerase I domain-containing protein 1657 1178-1183
739. GSRPRH B9QFI8 B9QFI8_TOXGO FHA domain-containing protein 2035 1639-1644
740. GSRPRH Q1JT76 Q1JT76_TOXGO Calpain-7, putative precursor 2101 958-963
741. GSRPRH V4Z5M2 V4Z5M2_TOXGO Calpain family cysteine protease domain-containing protein 2269 958-963
742. PRHASL V4Z4T9 V4Z4T9_TOXGO Uncharacterized protein 888 860-865
743. RHASLP V4YJB7 V4YJB7_TOXGO Protein kinase domain-containing protein 1290 1012-1017
744. HASLPS V4Z6Y3 V4Z6Y3_TOXGO Component of IIS longevity pathway protein SMK-1 1896 1543-1548
745. HASLPS V4ZBR6 V4ZBR6_TOXGO Uncharacterized protein 1695 180-185
746. ASLPSS B6K9J1 B6K9J1_TOXGO Inner centromere protein, ARK-binding region protein 1331 252-257
747. ASLPSS B6K9L3 B6K9L3_TOXGO Uncharacterized protein 1292 260-265
748. ASLPSS B6KRA7 B6KRA7_TOXGO ATP-dependent DNA helicase, RecQ family protein 1626 123-128
749. ASLPSS B9PVA3 B9PVA3_TOXGO SAG-related sequence SRS17B 379 198-203
750. ASLPSS B9PY91 B9PY91_TOXGO Putative transmembrane protein 603 81-86
751. ASLPSS B9Q2S2 B9Q2S2_TOXGO MtN3/saliva family protein 666 131-136
752. ASLPSS B9Q7P3 B9Q7P3_TOXGO Uncharacterized protein 2473 984-989
753. ASLPSS B9Q8D5 B9Q8D5_TOXGO tRNA-dihydrouridine(47) synthase [NAD(P)(+)] 1220 204-209
754. ASLPSS B9QAR3 B9QAR3_TOXGO Uncharacterized protein 1303 406-411
755. ASLPSS B9QEU6 B9QEU6_TOXGO Uncharacterized protein 351 119-124
756. ASLPSS B9QG55 B9QG55_TOXGO Putative kynurenine 3-monooxygenase and-related flavoprotein monooxygenase family (ISS) protein 1761 525-530
757. ASLPSS B9QGW8 B9QGW8_TOXGO RNA cap guanine-N2 methyltransferase 1653 892-897
758. ASLPSS B9QHZ2 B9QHZ2_TOXGO tRNA pseudouridine synthase 1512 502-507
759. ASLPSS B9QMU8 B9QMU8_TOXGO Putative transmembrane protein 2220 1515-1520
760. ASLPSS B9QNS1 B9QNS1_TOXGO tRNA pseudouridine synthase 1818 986-991
761. ASLPSS B9QQB3 B9QQB3_TOXGO Uncharacterized protein 329 5-10
762. ASLPSS Q1JSH2 Q1JSH2_TOXGO Uncharacterized protein 2102 1140-1145
763. ASLPSS Q1JSI5 Q1JSI5_TOXGO Uncharacterized protein 3127 2242-2247
764. ASLPSS Q1JTI0 Q1JTI0_TOXGO Putative uncharacterized protein 1056 57-62
765. ASLPSS Q1JTJ3 Q1JTJ3_TOXGO SET-domain protein, putative 4382 1255-1260
766. ASLPSS V4YLE4 V4YLE4_TOXGO Uncharacterized protein 1818 819-824
767. ASLPSS V4YND4 V4YND4_TOXGO eIF2 kinase IF2K-D (Incomplete catalytic triad) 2866 2721-2726
768. ASLPSS V4YU53 V4YU53_TOXGO Uncharacterized protein 2302 1080-1085, 1109-1114, 1138-1143
769. ASLPSS V4YXQ4 V4YXQ4_TOXGO Ubiquitin carboxyl-terminal hydrolase 3144 1057-1062
770. ASLPSS V4Z222 V4Z222_TOXGO RNA-directed DNA polymerase 4625 2743-2748
771. ASLPSS V4Z3N7 V4Z3N7_TOXGO Alpha/beta hydrolase family protein 1235 456-461
772. ASLPSS V4Z3X6 V4Z3X6_TOXGO DNA gyrase/topoisomerase IV, A subunit domain-containing protein 1273 206-211
773. ASLPSS V4Z3X9 V4Z3X9_TOXGO Uncharacterized protein 2041 1301-1306
774. ASLPSS V4Z559 V4Z559_TOXGO Putative histone lysine methyltransferase, SET 5175 1255-1260
775. ASLPSS V4Z5A8 V4Z5A8_TOXGO Uncharacterized protein 1237 146-151
776. ASLPSS V4Z5S4 V4Z5S4_TOXGO Uncharacterized protein 1302 960-965
777. ASLPSS V4Z6D7 V4Z6D7_TOXGO Uncharacterized protein 2936 2051-2056
778. ASLPSS V4Z827 V4Z827_TOXGO Glucose inhibited division protein A subfamily protein 1512 222-227
779. ASLPSS V4Z8C7 V4Z8C7_TOXGO Uncharacterized protein 518 214-219
780. ASLPSS V4ZAE2 V4ZAE2_TOXGO Bromodomain-containing protein 1827 266-271
781. ASLPSS V4ZAG4 V4ZAG4_TOXGO 3'5'-cyclic nucleotide phosphodiesterase domain-containing protein 2346 1481-1486
782. ASLPSS V4ZE64 V4ZE64_TOXGO Uncharacterized protein 1069 917-922
783. ASLPSS V4ZER8 V4ZER8_TOXGO Uncharacterized protein 4402 4126-4131
784. ASLPSS V4ZFJ6 V4ZFJ6_TOXGO HECT-domain (Ubiquitin-transferase) domain-containing protein 4155 1120-1125
785. ASLPSS V4ZFZ2 V4ZFZ2_TOXGO Sec7 domain-containing protein 3546 3498-3503
786. ASLPSS V4ZH38 V4ZH38_TOXGO Putative transmembrane protein 701 189-194
787. ASLPSS V4ZHQ4 V4ZHQ4_TOXGO Putative isoleucyl-tRNA synthetase 2447 1690-1695
788. ASLPSS V4ZJ80 V4ZJ80_TOXGO Amine-terminal region of chorein, A TM vesicle-mediated sorter 5383 3470-3475
789. ASLPSS V4ZJY8 V4ZJY8_TOXGO Histone lysine methyltransferase SET/SUV39 1547 959-964
790. ASLPSS V4ZK59 V4ZK59_TOXGO GTPase protein HflX 1145 43-48
791. ASLPSS V4ZLH4 V4ZLH4_TOXGO DUF3228 domain-containing protein 465 98-103
792. ASLPSS V4ZM65 V4ZM65_TOXGO Methyltransferase 1270 225-230
793. ASLPSS V4ZM93 V4ZM93_TOXGO NUC153 domain-containing protein 999 678-683
794. ASLPSS V4ZNF6 V4ZNF6_TOXGO Putative glutamic acid-rich protein 2698 2099-2104
795. ASLPSS V4ZQ12 V4ZQ12_TOXGO Putative tRNA splicing endonuclease 3982 530-535, 2374-2379
796. ASLPSS V4ZRM6 V4ZRM6_TOXGO Uncharacterized protein 1430 1165-1170
797. ASLPSS V4ZSN9 V4ZSN9_TOXGO Uncharacterized protein 1444 148-153
798. ASLPSS V4ZSV5 V4ZSV5_TOXGO Uncharacterized protein 864 644-649
799. ASLPSS V4ZUH7 V4ZUH7_TOXGO Uncharacterized protein 2230 361-366
800. ASLPSS V4ZWK4 V4ZWK4_TOXGO Myosin-light-chain kinase 1794 905-910
801. ASLPSS V5AW93 V5AW93_TOXGO Uncharacterized protein 1683 48-53
802. ASLPSS V5B0G2 V5B0G2_TOXGO DNA ligase 1331 156-161
803. ASLPSS V5BK89 V5BK89_TOXGO AP2 domain transcription factor AP2VIII-1 2102 1833-1838
804. ASLPSS V5BKW3 V5BKW3_TOXGO Putative omega secalin 866 461-466
805. SLPSSV B6KRH9 B6KRH9_TOXGO Uncharacterized protein 1898 37-42
806. SLPSSV B9Q2S2 B9Q2S2_TOXGO MtN3/saliva family protein 666 132-137
807. SLPSSV B9QK58 B9QK58_TOXGO Translation initiation factor eIF3 subunit 135 4956 1317-1322
808. SLPSSV V4YJB7 V4YJB7_TOXGO Protein kinase domain-containing protein 1290 831-836
809. SLPSSV V4YND4 V4YND4_TOXGO eIF2 kinase IF2K-D (Incomplete catalytic triad) 2866 2722-2727
810. SLPSSV V4YZX5 V4YZX5_TOXGO Acetyl-coA carboxylase ACC2 3400 2076-2081
811. SLPSSV V4Z5A8 V4Z5A8_TOXGO Uncharacterized protein 1237 147-152
812. SLPSSV V4Z5W3 V4Z5W3_TOXGO OTU family cysteine protease 988 930-935
813. SLPSSV V4ZAP5 V4ZAP5_TOXGO Zinc finger, C3HC4 type (RING finger) domain-containing protein 3893 2832-2837
814. SLPSSV V4ZDM0 V4ZDM0_TOXGO HEAT repeat-containing protein 4132 290-295
815. SLPSSV V4ZFJ6 V4ZFJ6_TOXGO HECT-domain (Ubiquitin-transferase) domain-containing protein 4155 711-716
816. SLPSSV V4ZN83 V4ZN83_TOXGO Ribosomal protein RPL22 595 256-261
817. SLPSSV V4ZT18 V4ZT18_TOXGO General transcription factor IIIC polypeptide 3 GTF3C3 1487 494-499
818. SLPSSV V5AX01 V5AX01_TOXGO Putative transmembrane protein 657 589-594
819. SLPSSV V5B468 V5B468_TOXGO Uncharacterized protein 2811 1102-1107
820. SLPSSV V5BKQ9 V5BKQ9_TOXGO Exonuclease 1889 1831-1836
821. LPSSVA B9QEX3 B9QEX3_TOXGO Bromodomain-containing protein 1354 1123-1128
822. LPSSVA V4YJB7 V4YJB7_TOXGO Protein kinase domain-containing protein 1290 832-837
823. LPSSVA V4YXQ9 V4YXQ9_TOXGO Uncharacterized protein 2949 2662-2667
824. LPSSVA V4ZFK6 V4ZFK6_TOXGO ER-trafficking TRAPP I complex 85 kDa subunit 3377 227-232
825. LPSSVA V4ZHM3 V4ZHM3_TOXGO Regulator of chromosome condensation (RCC1) repeat-containing protein 1933 1117-1122
826. LPSSVA V4ZKZ1 V4ZKZ1_TOXGO Uncharacterized protein 1477 118-123
827. PSSVAE B9QQ26 B9QQ26_TOXGO Serine/threonine-protein phosphatase 898 193-198
828. PSSVAE V4YRU8 V4YRU8_TOXGO Putative transmembrane protein 235 211-216
829. PSSVAE V4YYG6 V4YYG6_TOXGO Putative zinc finger protein 2469 1556-1561
830. PSSVAE V5AWI1 V5AWI1_TOXGO Putative transmembrane protein 359 335-340
831. SSVAEA B9QFW3 B9QFW3_TOXGO AP2 domain transcription factor AP2VIIa-7 3112 1569-1574
832. SSVAEA V4ZFE3 V4ZFE3_TOXGO Uncharacterized protein 940 556-561
833. SSVAEA V4ZHH3 V4ZHH3_TOXGO Toxolysin TLN4 2341 33-38
834. SVAEAF B9QFW3 B9QFW3_TOXGO AP2 domain transcription factor AP2VIIa-7 3112 1570-1575
835. VAEAFA B9PW73 B9PW73_TOXGO Uncharacterized protein 704 191-196
836. VAEAFA B9QFW3 B9QFW3_TOXGO AP2 domain transcription factor AP2VIIa-7 3112 1571-1576
837. VAEAFA B9QGY1 B9QGY1_TOXGO 6,7-dihydropteridine reductase 319 21-26
838. AEAFAR V4ZE47 V4ZE47_TOXGO DNA mismatch repair protein, C-terminal domain-containing protein 1329 1254-1259
839. AEAFAR V4ZTB4 V4ZTB4_TOXGO ULK kinase 2097 103-108
840. FARPSS V4ZHB5 V4ZHB5_TOXGO Uncharacterized protein 1792 465-470
841. FARPSS V4ZWS2 V4ZWS2_TOXGO Uncharacterized protein 4834 449-454
842. ARPSSL Q1JT30 Q1JT30_TOXGO Putative uncharacterized protein precursor 1580 1181-1186
843. ARPSSL V4YLL3 V4YLL3_TOXGO Putative transmembrane protein 1857 1454-1459
844. ARPSSL V4YMZ6 V4YMZ6_TOXGO Putative origin recognition complex subunit 878 270-275
845. ARPSSL V4Z9L6 V4Z9L6_TOXGO Uncharacterized protein 663 238-243
846. ARPSSL V4ZEL7 V4ZEL7_TOXGO CPSF A subunit region protein 2847 39-44
847. ARPSSL V4ZT82 V4ZT82_TOXGO Uncharacterized protein 3407 2702-2707
848. ARPSSL V5AWP5 V5AWP5_TOXGO HEAT repeat-containing protein 1766 858-863
849. RPSSLP B6KHS4 B6KHS4_TOXGO Uncharacterized protein 1731 1130-1135
850. RPSSLP V4YU53 V4YU53_TOXGO Uncharacterized protein 2302 716-721
851. RPSSLP V4Z639 V4Z639_TOXGO Putative transmembrane protein 264 126-131
852. RPSSLP V4Z9F1 V4Z9F1_TOXGO Zinc finger, C3HC4 type (RING finger) domain-containing protein 1284 586-591
853. RPSSLP V4ZHN1 V4ZHN1_TOXGO Uncharacterized protein 6085 2778-2783
854. RPSSLP V4ZKB2 V4ZKB2_TOXGO Uncharacterized protein 605 172-177
855. RPSSLP V4ZKU2 V4ZKU2_TOXGO WD domain, G-beta repeat-containing protein 1632 1110-1115
856. RPSSLP V4ZSD8 V4ZSD8_TOXGO Uncharacterized protein 1556 92-97
857. RPSSLP V5AWP5 V5AWP5_TOXGO HEAT repeat-containing protein 1766 859-864
858. RPSSLP V5BGZ2 V5BGZ2_TOXGO tRNA synthetases class I family protein 540 188-193
859. PSSLPA B6KR18 B6KR18_TOXGO Putative cell-cycle-associated protein kinase CDK 1372 259-264
860. PSSLPA B9PY86 B9PY86_TOXGO Uncharacterized protein 2407 364-369
861. PSSLPA B9PYH2 B9PYH2_TOXGO ATP-dependent RNA helicase, putative 1603 1046-1051
862. PSSLPA B9PZ67 B9PZ67_TOXGO Ethanolamine kinase 547 116-121
863. PSSLPA B9Q4T1 B9Q4T1_TOXGO Zinc finger, C3HC4 type (RING finger) domain-containing protein 603 57-62, 63-68, 69-74
864. PSSLPA B9QHQ6 B9QHQ6_TOXGO Trehalose-phosphatase 1222 179-184
865. PSSLPA K7X7G8 K7X7G8_TOXGO Palmitoyltransferase 510 67-72
866. PSSLPA Q1JT89 Q1JT89_TOXGO Putative uncharacterized protein 2393 350-355
867. PSSLPA V4YLG5 V4YLG5_TOXGO Helicase associated domain (Ha2) protein 1261 704-709
868. PSSLPA V4YMZ0 V4YMZ0_TOXGO Sushi domain (Scr repeat) domain-containing protein 3916 2867-2872
869. PSSLPA V4YRN2 V4YRN2_TOXGO Uncharacterized protein 720 228-233
870. PSSLPA V4YXF7 V4YXF7_TOXGO Calcium dependent protein kinase CDPK7 2133 1958-1963
871. PSSLPA V4Z2X5 V4Z2X5_TOXGO Uncharacterized protein 1443 428-433
872. PSSLPA V4Z5C3 V4Z5C3_TOXGO Helix-hairpin-helix motif domain-containing protein 1074 141-146
873. PSSLPA V4Z639 V4Z639_TOXGO Putative transmembrane protein 264 127-132
874. PSSLPA V4ZER8 V4ZER8_TOXGO Uncharacterized protein 4402 896-901
875. PSSLPA V4ZGV1 V4ZGV1_TOXGO Palmitoyltransferase 466 22-27
876. PSSLPA V4ZKD8 V4ZKD8_TOXGO Uncharacterized protein 11926 1998-2003
877. PSSLPA V4ZKT9 V4ZKT9_TOXGO ELMO/CED-12 family protein 2252 1264-1269
878. PSSLPA V4ZLP7 V4ZLP7_TOXGO Cyclic nucleotide-binding domain-containing protein 1533 763-768
879. PSSLPA V4ZSE9 V4ZSE9_TOXGO CMGC kinase, MAPK family (ERK) MAPK-1 1298 566-571
880. PSSLPA V5B857 V5B857_TOXGO Pentatricopeptide repeat domain-containing protein 2538 2293-2298
881. PSSLPA V5BMD9 V5BMD9_TOXGO UBA/TS-N domain-containing protein 7817 4607-4612
882. SSLPAG B9QI17 B9QI17_TOXGO Transcription initiation factor TFIID subunit TAF5 1095 363-368
883. SSLPAG B9QMB6 B9QMB6_TOXGO AP2 domain transcription factor AP2IX-8 1753 1274-1279
884. SSLPAG V4YRN2 V4YRN2_TOXGO Uncharacterized protein 720 229-234
885. SSLPAG V4YXF7 V4YXF7_TOXGO Calcium dependent protein kinase CDPK7 2133 1959-1964
886. SSLPAG V4Z4M5 V4Z4M5_TOXGO Alanine--tRNA ligase 1280 16-21
887. SSLPAG V4ZAZ4 V4ZAZ4_TOXGO AP2 domain transcription factor AP2X-7 1869 1533-1538
888. SSLPAG V4ZD65 V4ZD65_TOXGO Uncharacterized protein 1913 1427-1432
889. SSLPAG V5B4V7 V5B4V7_TOXGO Uncharacterized protein 2975 175-180
890. SSLPAG V5B7V4 V5B7V4_TOXGO tRNA pseudouridine synthase D 1489 830-835
891. SLPAGC B9QMB6 B9QMB6_TOXGO AP2 domain transcription factor AP2IX-8 1753 1275-1280
892. LPAGCT V4Z9X5 V4Z9X5_TOXGO Uncharacterized protein 4983 4222-4227
893. ARPDGH V4Z5J8 V4Z5J8_TOXGO Condensin complex subunit 1 2466 252-257
894. PDGHSA I7F9D1 I7F9D1_TOXGO Inner membrane complex sub-compartment protein 4 181 168-173
895. PDGHSA V5BCQ4 V5BCQ4_TOXGO IMC subcompartment protein ISP4 181 168-173
896. GHSACR V4ZNM3 V4ZNM3_TOXGO NEK kinase 4925 3310-3315
897. HSACRR V4ZNM3 V4ZNM3_TOXGO NEK kinase 4925 3311-3316
898. SACRRL K4HQR5 K4HQR5_TOXGO Apicoplast Tic22 536 90-95
899. SACRRL V4Z435 V4Z435_TOXGO Putative histone lysine methyltransferase, SET 1798 96-101
900. SACRRL V4ZBX8 V4ZBX8_TOXGO Phosphatidylinositol 3-and 4-kinase 6746 2217-2222
901. SACRRL V5B4E7 V5B4E7_TOXGO Tic22-like family protein 536 90-95
902. ACRRLA K4HQR5 K4HQR5_TOXGO Apicoplast Tic22 536 91-96
903. ACRRLA V4YU53 V4YU53_TOXGO Uncharacterized protein 2302 2252-2257
904. ACRRLA V5B4E7 V5B4E7_TOXGO Tic22-like family protein 536 91-96
905. CRRLAQ B9QPR3 B9QPR3_TOXGO SWI2/SNF2 SRCAP/Ino80 2924 598-603
906. CRRLAQ Q7Z2C2 Q7Z2C2_TOXGO Snf2-related chromatin remodeling factor SRCAP 2924 598-603
907. CRRLAQ V4ZIN3 V4ZIN3_TOXGO Uncharacterized protein 2277 586-591
908. RRLAQA B9PWV5 B9PWV5_TOXGO Radial spoke protein 3 protein 426 244-249
909. RRLAQA V4YI07 V4YI07_TOXGO Trypsin domain-containing protein 741 128-133
910. RRLAQA V5BKN7 V5BKN7_TOXGO Putative GCN1 3416 1650-1655
911. RLAQAQ B9PWV5 B9PWV5_TOXGO Radial spoke protein 3 protein 426 245-250
912. LPIYRE V4ZJY6 V4ZJY6_TOXGO Putative transmembrane protein 875 869-874
913. QEGEQA V4Z994 V4Z994_TOXGO DHHC zinc finger domain-containing protein 951 612-617
914. QEGEQA V4ZNI2 V4ZNI2_TOXGO WW domain protein 2115 1900-1905
915. EGEQAG V4Z265 V4Z265_TOXGO Putative named for A Pro-Trp-Trp-Pro motif protein 1235 711-716
916. EGEQAG V4ZKG4 V4ZKG4_TOXGO Zinc finger (CCCH type) motif-containing protein 816 608-613
917. GEQAGA B9QNA9 B9QNA9_TOXGO Putative transmembrane protein 490 411-416
918. QAGAPA B6KA08 B6KA08_TOXGO TAF7-like RNA polymerase II TAF7L 728 535-540
919. CLHAHA B9QMF6 B9QMF6_TOXGO CAM kinase, CDPK family 1388 1374-1379
920. AHAHLP V4YYG6 V4YYG6_TOXGO Putative zinc finger protein 2469 2288-2293
921. LPPCAS V4Z2Y1 V4Z2Y1_TOXGO Uncharacterized protein 3246 567-572
922. LPPCAS V4ZGI1 V4ZGI1_TOXGO Calcium-dependent protein kinase 2228 1045-1050
923. LPPCAS V5BIM4 V5BIM4_TOXGO Uncharacterized protein 1813 1135-1140
924. PCASHG V4ZVI6 V4ZVI6_TOXGO SufB/sufD domain-containing protein 1860 1238-1243
925. LGHRGR B9QK58 B9QK58_TOXGO Translation initiation factor eIF3 subunit 135 4956 2300-2305
926. LGLGTG V4Z1N3 V4Z1N3_TOXGO Uncharacterized protein 1389 355-360
927. RDSGGL V4YZ80 V4YZ80_TOXGO HECT-domain (Ubiquitin-transferase) domain-containing protein 15897 10330-10335
928. RDSGGL V4ZA17 V4ZA17_TOXGO Uncharacterized protein 2390 954-959
929. DSGGLD V4ZA17 V4ZA17_TOXGO Uncharacterized protein 2390 955-960
930. LDEISR V5BEJ4 V5BEJ4_TOXGO Ubiquitin carboxyl-terminal hydrolase 1328 433-438
931. SRVARG B9QJ03 B9QJ03_TOXGO Uncharacterized protein 1306 231-236
932. RVARGT B9PHD3 B9PHD3_TOXGO Mov34/MPN/PAD-1 family protein 343 52-57
933. VARGTQ V4ZL93 V4ZL93_TOXGO Uncharacterized protein 1323 235-240
934. ARGTQG V4Z3Y5 V4Z3Y5_TOXGO TBC domain-containing protein 3409 2271-2276
935. ARGTQG V4Z4W9 V4Z4W9_TOXGO Uncharacterized protein 2995 434-439
936. ARGTQG V4ZL93 V4ZL93_TOXGO Uncharacterized protein 1323 236-241
937. RGTQGF V4YMZ0 V4YMZ0_TOXGO Sushi domain (Scr repeat) domain-containing protein 3916 2241-2246
938. RRISSL B9QMU8 B9QMU8_TOXGO Putative transmembrane protein 2220 1810-1815
939. RRISSL Q1JT24 Q1JT24_TOXGO Putative uncharacterized protein 1511 1193-1198
940. RRISSL V4Z5G2 V4Z5G2_TOXGO Uncharacterized protein 842 718-723
941. RRISSL V5BJ10 V5BJ10_TOXGO Ribosomal l25 family protein 739 10-15
942. ISSLES B9PQP9 B9PQP9_TOXGO Zinc finger (CCCH type) motif-containing protein 595 572-577
943. SSLESE B9Q4N1 B9Q4N1_TOXGO Hsp70 interacting protein HIP 425 72-77
944. SSLESE Q0PW50 Q0PW50_TOXGO Hsp70 interacting protein 425 72-77
945. SSLESE V4ZJ50 V4ZJ50_TOXGO Oligomeric complex protein COG6 1298 519-524
946. SLESEV B9Q4N1 B9Q4N1_TOXGO Hsp70 interacting protein HIP 425 73-78
947. SLESEV Q0PW50 Q0PW50_TOXGO Hsp70 interacting protein 425 73-78

**NMDA 2D**

1. RGAGGP B9Q8F4 B9Q8F4_TOXGO Uncharacterized protein 386 269-274
2. RGAGGP Q1KSE5 Q1KSE5_TOXGO Mitochondrial putative ATP-specific succinyl-CoA synthetase beta subunit 498 204-209
3. GAGGPR B9Q8F4 B9Q8F4_TOXGO Uncharacterized protein 386 270-275
4. GAGGPR B9QFF3 B9QFF3_TOXGO Putative transmembrane protein 2209 269-274
5. GAGGPR B9QMH6 B9QMH6_TOXGO RNA pseudouridine synthase superfamily protein 6535 6333-6338
6. GAGGPR Q86PI1 Q86PI1_TOXGO Jlp2 550 537-542
7. GAGGPR V4Z7A0 V4Z7A0_TOXGO Dense granule protein GRA15 635 622-627
8. GAGGPR V5B872 V5B872_TOXGO Myb family DNA-binding domain-containing protein 1755 1318-1323
9. AGGPRG B9Q8F4 B9Q8F4_TOXGO Uncharacterized protein 386 271-276
10. AGGPRG V4Z757 V4Z757_TOXGO Putative platelet-binding protein GspB 1383 199-204
11. AGGPRG V4ZSU4 V4ZSU4_TOXGO Putative transmembrane protein 1274 365-370
12. AGGPRG V5B7E4 V5B7E4_TOXGO Putative transmembrane protein 2871 1625-1630
13. GGPRGP B6KA08 B6KA08_TOXGO TAF7-like RNA polymerase II TAF7L 728 511-516
14. GGPRGP B6KJR0 B6KJR0_TOXGO Uncharacterized protein 446 33-38
15. GGPRGP B9QN68 B9QN68_TOXGO AP2 domain transcription factor AP2X-8 3817 3702-3707
16. GGPRGP V4YQ73 V4YQ73_TOXGO MIF4G domain-containing protein 3756 44-49
17. GGPRGP V4YT92 V4YT92_TOXGO Uncharacterized protein 483 61-66
18. GGPRGP V4ZGB0 V4ZGB0_TOXGO Zinc finger (CCCH type) motif-containing protein 1513 582-587
19. GPRGPR B6KA08 B6KA08_TOXGO TAF7-like RNA polymerase II TAF7L 728 512-517
20. GPRGPR V4YR59 V4YR59_TOXGO Uncharacterized protein 1342 323-328
21. PRGPRG B6KA08 B6KA08_TOXGO TAF7-like RNA polymerase II TAF7L 728 513-518
22. PRGPRG Q1JSJ1 Q1JSJ1_TOXGO Uncharacterized protein 3577 169-174
23. PRGPRG V4YKS0 V4YKS0_TOXGO Uncharacterized protein 1393 476-481
24. PRGPRG V4Z251 V4Z251_TOXGO Uncharacterized protein 3633 169-174
25. PRGPRG V5BLE9 V5BLE9_TOXGO AP2 domain transcription factor AP2VIII-4 3417 125-130
26. RGPRGP Q1JSJ1 Q1JSJ1_TOXGO Uncharacterized protein 3577 170-175
27. RGPRGP V4Z251 V4Z251_TOXGO Uncharacterized protein 3633 170-175
28. GPRGPA B6KJR0 B6KJR0_TOXGO Uncharacterized protein 446 34-39
29. GPRGPA B9QN68 B9QN68_TOXGO AP2 domain transcription factor AP2X-8 3817 3703-3708
30. GPRGPA Q1JSJ1 Q1JSJ1_TOXGO Uncharacterized protein 3577 1164-1169
31. GPRGPA V4Z251 V4Z251_TOXGO Uncharacterized protein 3633 1164-1169
32. KMLLLL V5B4Q4 V5B4Q4_TOXGO Uncharacterized protein 1046 22-27
33. MLLLLA Q1JSG8 Q1JSG8_TOXGO Uncharacterized protein precursor 178 23-28
34. MLLLLA V4YMF8 V4YMF8_TOXGO Uncharacterized protein 201 46-51
35. LLLLAL B6K9K5 B6K9K5_TOXGO Putative transmembrane protein 187 108-113
36. LLLLAL B6KAG9 B6KAG9_TOXGO U3 small nucleolar RNA-associated protein 10 3738 1151-1156
37. LLLLAL B6KB41 B6KB41_TOXGO Putative calcium signaling protein kinase RAD53 1436 690-695
38. LLLLAL B6KHJ3 B6KHJ3_TOXGO Putative dynein heavy chain 2 4140 1685-1690
39. LLLLAL B6KHR8 B6KHR8_TOXGO Eukaryotic translation initiation factor 3 subunit H 600 43-48
40. LLLLAL B9PQJ8 B9PQJ8_TOXGO Uncharacterized protein 1162 303-308
41. LLLLAL B9Q415 B9Q415_TOXGO Putative transmembrane protein 292 239-244
42. LLLLAL F1DI03 F1DI03_TOXGO Putative metalloproteinase TLN4 2435 12-17
43. LLLLAL V4YL69 V4YL69_TOXGO Rft protein 1197 571-576
44. LLLLAL V4YZ17 V4YZ17_TOXGO Putative transmembrane protein 1298 1116-1121
45. LLLLAL V4Z0R4 V4Z0R4_TOXGO Uncharacterized protein 738 8-13
46. LLLLAL V4Z439 V4Z439_TOXGO Putative histone lysine methyltransferase, SET 1124 93-98
47. LLLLAL V4ZDF9 V4ZDF9_TOXGO Putative GTP-binding protein engB 1027 38-43
48. LLLLAL V4ZFC2 V4ZFC2_TOXGO Putative vacuolar proton translocating ATPase subunit 1066 214-219
49. LLLLAL V4ZU57 V4ZU57_TOXGO Mediator complex subunit MED14 4355 69-74
50. LLLLAL V4ZV93 V4ZV93_TOXGO Uncharacterized protein 4618 4017-4022
51. LLLLAL V5B9Q9 V5B9Q9_TOXGO Uncharacterized protein 126 72-77
52. LLLLAL V5BDZ6 V5BDZ6_TOXGO Uncharacterized protein 4591 4324-4329
53. LLLLAL V5BE34 V5BE34_TOXGO Uncharacterized protein 1239 78-83
54. LLLLAL V5BIX0 V5BIX0_TOXGO Uncharacterized protein 296 28-33
55. LLLALA B9PP41 B9PP41_TOXGO Uncharacterized protein 225 50-55
56. LLLALA B9PQJ8 B9PQJ8_TOXGO Uncharacterized protein 1162 304-309
57. LLLALA B9Q562 B9Q562_TOXGO Non-specific serine/threonine protein kinase 8428 6264-6269
58. LLLALA V4Z002 V4Z002_TOXGO Phosphotransferase enzyme family protein 827 757-762
59. LLLALA V4Z581 V4Z581_TOXGO Protein SEY1 homolog 893 781-786
60. LLLALA V4Z8T5 V4Z8T5_TOXGO Uncharacterized protein 3210 1629-1634
61. LLLALA V4ZCK1 V4ZCK1_TOXGO Uncharacterized protein 593 391-396
62. LLLALA V4ZED0 V4ZED0_TOXGO Thioredoxin domain protein 1014 14-19
63. LLLALA V5BKC0 V5BKC0_TOXGO Uncharacterized protein 2239 1943-1948
64. LLALAC V4ZJL5 V4ZJL5_TOXGO Uncharacterized protein 797 30-35
65. ALACAS V4ZEE7 V4ZEE7_TOXGO Putative transmembrane protein 794 470-475
66. ALACAS V4ZEN0 V4ZEN0_TOXGO Adaptor complexes medium subunit family protein 2062 859-864
67. LACASP B9QHY1 B9QHY1_TOXGO Uncharacterized protein 2240 386-391
68. LACASP B9QN68 B9QN68_TOXGO AP2 domain transcription factor AP2X-8 3817 166-171
69. LACASP B9QR17 B9QR17_TOXGO Uncharacterized protein 6038 4125-4130
70. LACASP V4YSI1 V4YSI1_TOXGO Thiamin pyrophosphokinase, catalytic domain-containing protein 813 167-172
71. ACASPF B9QR17 B9QR17_TOXGO Uncharacterized protein 6038 4126-4131
72. ACASPF V4ZE60 V4ZE60_TOXGO Internal kinesin motor domain protein 1360 742-747
73. ACASPF V4ZHJ4 V4ZHJ4_TOXGO Uncharacterized protein 984 563-568
74. CASPFP B9Q843 B9Q843_TOXGO Histone lysine methyltransferase SET1 7555 944-949
75. CASPFP B9QR17 B9QR17_TOXGO Uncharacterized protein 6038 4127-4132
76. CASPFP V4YY43 V4YY43_TOXGO Putative transmembrane protein 862 286-291
77. SPFPEE V4YXY6 V4YXY6_TOXGO GCC2 and protein GCC3 921 686-691
78. SPFPEE V5B333 V5B333_TOXGO Radical SAM domain-containing protein 680 524-529
79. PFPEEA V4YXY6 V4YXY6_TOXGO GCC2 and protein GCC3 921 687-692
80. PEEAPG B9QDW1 B9QDW1_TOXGO ATP-binding cassette G family transporter ABCG96 1064 9-14
81. PEEAPG B9QJL9 B9QJL9_TOXGO Uncharacterized protein 1492 285-290
82. PEEAPG D3XD36 D3XD36_TOXGO ATP-binding cassette transporter G family ABCG-96 protein 1045 9-14
83. PEEAPG Q45W16 Q45W16_TOXGO ATP-binding cassette, sub-family G, member 4 992 9-14
84. PEEAPG V4Z4K1 V4Z4K1_TOXGO Peptidase, S9A/B/C family, catalytic domain protein 2343 1528-1533
85. PEEAPG V4ZAE2 V4ZAE2_TOXGO Bromodomain-containing protein 1827 1103-1108
86. EEAPGP V4YYU7 V4YYU7_TOXGO Putative glyoxalase 4813 591-596
87. EEAPGP V5B368 V5B368_TOXGO Transporter, major facilitator family protein 760 33-38
88. EAPGPG V4Z894 V4Z894_TOXGO ATPase family associated with various cellular activities (AAA) subfamily protein 9030 68-73
89. APGPGG B9PWH3 B9PWH3_TOXGO Uncharacterized protein 173 104-109
90. APGPGG B9QND3 B9QND3_TOXGO Uncharacterized protein 1975 1385-1390
91. APGPGG V4ZL76 V4ZL76_TOXGO WD domain, G-beta repeat-containing protein 1802 902-907
92. PGPGGA B9QND3 B9QND3_TOXGO Uncharacterized protein 1975 1386-1391
93. PGPGGA Q1JTB7 Q1JTB7_TOXGO Dynein heavy chain, putative 4991 196-201
94. PGPGGA V4Z5R6 V4Z5R6_TOXGO Dynein heavy chain 4974 196-201
95. GPGGAG B9Q479 B9Q479_TOXGO WD domain, G-beta repeat-containing protein 3633 341-346
96. GPGGAG B9QEC7 B9QEC7_TOXGO CCR4-Not complex component, Not1 protein 2562 930-935
97. GPGGAG B9QN68 B9QN68_TOXGO AP2 domain transcription factor AP2X-8 3817 1569-1574
98. GPGGAG V4YR46 V4YR46_TOXGO Sec1 family protein 1046 885-890
99. GPGGAG V4Z839 V4Z839_TOXGO Uncharacterized protein 1953 663-668
100. GPGGAG V4ZEN0 V4ZEN0_TOXGO Adaptor complexes medium subunit family protein 2062 1854-1859
101. GPGGAG V4ZQG9 V4ZQG9_TOXGO Uncharacterized protein 3806 2841-2846
102. GPGGAG V5B996 V5B996_TOXGO Endonuclease/exonuclease/phosphatase family protein 1558 919-924
103. GPGGAG V5BL66 V5BL66_TOXGO KOW motif domain-containing protein 1169 731-736
104. PGGAGG B9Q4N1 B9Q4N1_TOXGO Hsp70 interacting protein HIP 425 301-306
105. PGGAGG B9QDB9 B9QDB9_TOXGO Uncharacterized protein 2034 217-222
106. PGGAGG B9QEC7 B9QEC7_TOXGO CCR4-Not complex component, Not1 protein 2562 931-936
107. PGGAGG B9QHQ3 B9QHQ3_TOXGO Uncharacterized protein 2132 732-737
108. PGGAGG Q0PW50 Q0PW50_TOXGO Hsp70 interacting protein 425 301-306
109. PGGAGG V4Z1G0 V4Z1G0_TOXGO AP2 domain transcription factor AP2XII-2 1737 260-265
110. GGAGGP V4YKX4 V4YKX4_TOXGO Serine/threonine specific protein phosphatase 2883 847-852
111. GGAGGP V4Z1I1 V4Z1I1_TOXGO Eukaryotic translation initiation factor 3 subunit C 971 905-910
112. GGAGGP V4Z4M8 V4Z4M8_TOXGO Amine-terminal region of chorein, A TM vesicle-mediated sorter 13455 10690-10695
113. GGAGGP V4ZIQ1 V4ZIQ1_TOXGO Uncharacterized protein 1557 1346-1351
114. GGAGGP V5B327 V5B327_TOXGO Putative initiation factor subunit 2 family protein 304 96-101
115. GGAGGP V5B364 V5B364_TOXGO Putative initiation factor subunit 2 family protein 219 96-101
116. GGAGGP V5B872 V5B872_TOXGO Myb family DNA-binding domain-containing protein 1755 1317-1322
117. GAGGPG B6KR53 B6KR53_TOXGO Cyclin, N-terminal domain-containing protein 1174 471-476
118. GAGGPG B9PS36 B9PS36_TOXGO Putative transport protein Sec24 1019 653-658
119. GAGGPG B9PX83 B9PX83_TOXGO Protein kinase, other 1130 642-647
120. GAGGPG B9QF05 B9QF05_TOXGO Uncharacterized protein 1594 1334-1339
121. GAGGPG B9QJP2 B9QJP2_TOXGO RNA polymerase-associated protein RTF1 783 180-185
122. GAGGPG B9QQH7 B9QQH7_TOXGO Indole-3-glycerol phosphate synthase domain-containing protein 1672 1193-1198
123. GAGGPG V4Z089 V4Z089_TOXGO Gar1/Naf1 RNA-binding region protein 989 866-871
124. GAGGPG V4Z1I1 V4Z1I1_TOXGO Eukaryotic translation initiation factor 3 subunit C 971 906-911
125. GAGGPG V4ZJ80 V4ZJ80_TOXGO Amine-terminal region of chorein, A TM vesicle-mediated sorter 5383 631-636
126. GAGGPG V4ZMA8 V4ZMA8_TOXGO Hsp90 domain-containing protein 464 278-283
127. AGGPGG B6K9V8 B6K9V8_TOXGO RNA pseudouridine synthase superfamily protein 2780 2328-2333
128. AGGPGG B6KJU7 B6KJU7_TOXGO RING finger protein 1261 1234-1239
129. AGGPGG B9Q560 B9Q560_TOXGO Transport protein Trs120 2958 2518-2523
130. AGGPGG B9Q655 B9Q655_TOXGO RNA recognition motif-containing protein 1374 652-657
131. AGGPGG Q6RYT4 Q6RYT4_TOXGO Sodium/hydrogen exchanger 1 2097 778-783
132. AGGPGG V4YVI5 V4YVI5_TOXGO Na+/H+ exchanger NHE1 1440 121-126
133. AGGPGG V4Z1I1 V4Z1I1_TOXGO Eukaryotic translation initiation factor 3 subunit C 971 907-912
134. AGGPGG V4Z5L9 V4Z5L9_TOXGO S1 RNA binding domain-containing protein 1523 521-526
135. AGGPGG V4ZGX3 V4ZGX3_TOXGO TBC domain-containing protein 2113 599-604
136. AGGPGG V4ZPY4 V4ZPY4_TOXGO RAVE 1 carboxy-terminal protein 6665 5292-5297
137. AGGPGG V4ZSE4 V4ZSE4_TOXGO WD domain, G-beta repeat-containing protein 4664 1147-1152
138. AGGPGG V5BCJ7 V5BCJ7_TOXGO Subtilisin SUB4 1242 1209-1214
139. AGGPGG V5BEQ5 V5BEQ5_TOXGO Putative sortilin 1033 908-913
140. GGPGGG B9QEC7 B9QEC7_TOXGO CCR4-Not complex component, Not1 protein 2562 1008-1013
141. GGPGGG B9QFU9 B9QFU9_TOXGO ATP-dependent metallopeptidase HflB subfamily protein 1188 608-613
142. GGPGGG B9QJI0 B9QJI0_TOXGO Histone lysine acetyltransferase GCN5-B 1032 359-364
143. GGPGGG B9QMH6 B9QMH6_TOXGO RNA pseudouridine synthase superfamily protein 6535 1125-1130, 1135-1140
144. GGPGGG Q5EK48 Q5EK48_TOXGO GNAT family histone acetyltransferase GCN5-B 1032 359-364
145. GGPGGG V4ZFL6 V4ZFL6_TOXGO DnaJ domain-containing protein 755 481-486
146. GGPGGG V4ZTV0 V4ZTV0_TOXGO Ubiquitin carboxyl-terminal hydrolase 4218 599-604
147. GPGGGL V5BE34 V5BE34_TOXGO Uncharacterized protein 1239 546-551
148. PGGGLG V4Z8X4 V4Z8X4_TOXGO Putative toxoplasma gondii family D protein 1539 49-54
149. GGGLGG B6KA11 B6KA11_TOXGO Zinc finger (CCCH type) motif-containing protein 1051 362-367
150. GGGLGG B9PXD4 B9PXD4_TOXGO Putative replication protein A2 278 16-21
151. GGGLGG B9QLW7 B9QLW7_TOXGO Ribosomal protein RPS9 271 210-215
152. GGGLGG O77216 O77216_TOXGO Ribosomal protein S9 homolog 272 211-216
153. GGGLGG Q9UBA1 Q9UBA1_TOXGO Small ribosomal protein S9 precursor 271 210-215
154. GGGLGG Q9UBA2 Q9UBA2_TOXGO Small ribosomal protein S9 precursor 267 206-211
155. GGGLGG V5AYS4 V5AYS4_TOXGO Uncharacterized protein 406 226-231
156. GGLGGA B6KU32 B6KU32_TOXGO Putative RNA-binding protein Nova-1 412 229-234
157. GGLGGA B9Q5Q9 B9Q5Q9_TOXGO Putative transmembrane protein 865 833-838
158. GGLGGA V4ZAZ4 V4ZAZ4_TOXGO AP2 domain transcription factor AP2X-7 1869 1306-1311
159. GGLGGA V4ZMK9 V4ZMK9_TOXGO NLI interacting factor family phosphatase 1248 169-174
160. GGLGGA V5BE34 V5BE34_TOXGO Uncharacterized protein 1239 593-598
161. GLGGAR V5BE34 V5BE34_TOXGO Uncharacterized protein 1239 594-599
162. GGARPL V4ZV93 V4ZV93_TOXGO Uncharacterized protein 4618 3073-3078
163. GARPLN V5AZ22 V5AZ22_TOXGO Rhoptry neck protein RON8 2980 2361-2366
164. LNVALV B6KIB2 B6KIB2_TOXGO Rhoptry kinase family protein 554 31-36
165. LNVALV F8QMD3 F8QMD3_TOXGO Rhoptry protein 18 554 31-36
166. LNVALV F8QMD4 F8QMD4_TOXGO Rhoptry kinase family protein 554 31-36
167. LNVALV F8QMD6 F8QMD6_TOXGO Rhoptry protein 18 554 31-36
168. LNVALV F8QMD7 F8QMD7_TOXGO Rhoptry protein 18 554 31-36
169. LNVALV F8QMD8 F8QMD8_TOXGO Rhoptry kinase family protein 554 31-36
170. LNVALV F8QMD9 F8QMD9_TOXGO Rhoptry protein 18 554 31-36
171. LNVALV F8QME0 F8QME0_TOXGO Rhoptry protein 18 554 31-36
172. LNVALV F8QME2 F8QME2_TOXGO Rhoptry protein 18 554 31-36
173. LNVALV F8QME5 F8QME5_TOXGO Rhoptry protein 18 554 31-36
174. LNVALV I7BEY8 I7BEY8_TOXGO Rhoptry kinase family protein 554 31-36
175. LNVALV I7CBG7 I7CBG7_TOXGO Rhoptry kinase family protein 554 31-36
176. LNVALV I7CQ33 I7CQ33_TOXGO Rhoptry kinase family protein 554 31-36
177. LNVALV Q2PAY2 Q2PAY2_TOXGO Rhoptry kinase family protein 554 31-36
178. NVALVF B6KIB2 B6KIB2_TOXGO Rhoptry kinase family protein 554 32-37
179. NVALVF F8QMD3 F8QMD3_TOXGO Rhoptry protein 18 554 32-37
180. NVALVF F8QMD4 F8QMD4_TOXGO Rhoptry kinase family protein 554 32-37
181. NVALVF F8QMD6 F8QMD6_TOXGO Rhoptry protein 18 554 32-37
182. NVALVF F8QMD7 F8QMD7_TOXGO Rhoptry protein 18 554 32-37
183. NVALVF F8QMD8 F8QMD8_TOXGO Rhoptry kinase family protein 554 32-37
184. NVALVF F8QMD9 F8QMD9_TOXGO Rhoptry protein 18 554 32-37
185. NVALVF F8QME0 F8QME0_TOXGO Rhoptry protein 18 554 32-37
186. NVALVF F8QME2 F8QME2_TOXGO Rhoptry protein 18 554 32-37
187. NVALVF F8QME3 F8QME3_TOXGO Rhoptry protein 18 554 32-37
188. NVALVF F8QME5 F8QME5_TOXGO Rhoptry protein 18 554 32-37
189. NVALVF I7BEY8 I7BEY8_TOXGO Rhoptry kinase family protein 554 32-37
190. NVALVF I7CBG7 I7CBG7_TOXGO Rhoptry kinase family protein 554 32-37
191. NVALVF I7CQ33 I7CQ33_TOXGO Rhoptry kinase family protein 554 32-37
192. NVALVF Q2PAY2 Q2PAY2_TOXGO Rhoptry kinase family protein 554 32-37
193. ALVFSG A5YVK6 A5YVK6_TOXGO Regulator of chromosome condensation 1 1155 493-498
194. ALVFSG B9QGB2 B9QGB2_TOXGO Regulator of chromosome condensation RCC1 1156 493-498
195. ALVFSG V5BA36 V5BA36_TOXGO DnaJ domain-containing protein 383 30-35
196. VFSGPA V4Z7B8 V4Z7B8_TOXGO Alpha/beta hydrolase family protein 3457 2823-2828
197. VFSGPA V5AY08 V5AY08_TOXGO Transporter, major facilitator family protein 713 159-164
198. FSGPAY B9QIC7 B9QIC7_TOXGO RNA recognition motif-containing protein 1216 968-973
199. FSGPAY V5AY08 V5AY08_TOXGO Transporter, major facilitator family protein 713 160-165
200. SGPAYA Q1JTI7 Q1JTI7_TOXGO Putative uncharacterized protein 3352 2817-2822
201. SGPAYA V4Z556 V4Z556_TOXGO Zinc finger in N-recognin protein 4439 3737-3742
202. SGPAYA V4ZFL3 V4ZFL3_TOXGO Uncharacterized protein 1163 826-831
203. SGPAYA V4ZHN1 V4ZHN1_TOXGO Uncharacterized protein 6085 4065-4070
204. PAYAAE V4Z952 V4Z952_TOXGO Uncharacterized protein 4210 93-98
205. AAEAAR B6KDL5 B6KDL5_TOXGO ABC transporter transmembrane region domain-containing protein 1163 492-497
206. AAEAAR B6KHE3 B6KHE3_TOXGO Flavodoxin domain-containing protein 1027 125-130
207. AAEAAR B6KRK8 B6KRK8_TOXGO Putative adenylosuccinate lyase 661 44-49
208. AAEAAR B9PJB3 B9PJB3_TOXGO Uncharacterized protein 139 32-37
209. AAEAAR B9PWV5 B9PWV5_TOXGO Radial spoke protein 3 protein 426 380-385
210. AAEAAR B9Q592 B9Q592_TOXGO Uncharacterized protein 252 69-74
211. AAEAAR B9QFY9 B9QFY9_TOXGO Uncharacterized protein 1148 1054-1059
212. AAEAAR B9QIT9 B9QIT9_TOXGO Glycosyl hydrolase, family 31 protein 1618 657-662
213. AAEAAR B9QJ77 B9QJ77_TOXGO Uncharacterized protein 2002 241-246
214. AAEAAR Q1JSF8 Q1JSF8_TOXGO Uncharacterized protein 844 406-411
215. AAEAAR Q4FCM0 Q4FCM0_TOXGO ATP-binding cassette protein subfamily B member 3 1040 369-374
216. AAEAAR V4YUG7 V4YUG7_TOXGO Bromodomain protein 647 162-167
217. AAEAAR V4YXI9 V4YXI9_TOXGO Putative membrane protein 355 254-259
218. AAEAAR V4Z5U2 V4Z5U2_TOXGO TBC domain-containing protein 2711 1706-1711
219. AAEAAR V4Z6G6 V4Z6G6_TOXGO Putative myosin heavy chain 844 406-411
220. AAEAAR V4Z6N1 V4Z6N1_TOXGO Uncharacterized protein 1674 1544-1549
221. AAEAAR V4ZAK3 V4ZAK3_TOXGO Uncharacterized protein 3378 2667-2672
222. AAEAAR V4ZD18 V4ZD18_TOXGO Uncharacterized protein 3221 2546-2551
223. AAEAAR V4ZFB0 V4ZFB0_TOXGO Coiled-coil domain containing 124 family protein 231 20-25
224. AAEAAR V4ZFW2 V4ZFW2_TOXGO Myb family DNA-binding domain-containing protein 888 548-553
225. AAEAAR V4ZIR0 V4ZIR0_TOXGO Ubiquitin carboxyl-terminal hydrolase 2294 460-465
226. AAEAAR V4ZNR4 V4ZNR4_TOXGO Uncharacterized protein 568 457-462
227. AAEAAR V4ZP18 V4ZP18_TOXGO Putative transmembrane protein 2590 1083-1088
228. AAEAAR V4ZW70 V4ZW70_TOXGO Uncharacterized protein 5655 441-446
229. AAEAAR V5BKE2 V5BKE2_TOXGO Uncharacterized protein 886 545-550
230. AEAARL B6KRK8 B6KRK8_TOXGO Putative adenylosuccinate lyase 661 45-50
231. AEAARL B9PJB3 B9PJB3_TOXGO Uncharacterized protein 139 33-38
232. AEAARL B9PWV5 B9PWV5_TOXGO Radial spoke protein 3 protein 426 381-386
233. AEAARL B9Q7J7 B9Q7J7_TOXGO DNA-directed RNA polymerase III POLR3C 823 773-778
234. AEAARL B9QHQ3 B9QHQ3_TOXGO Uncharacterized protein 2132 908-913
235. AEAARL B9QI99 B9QI99_TOXGO Kinesin motor domain-containing protein 1317 673-678
236. AEAARL Q1JT30 Q1JT30_TOXGO Putative uncharacterized protein precursor 1580 1399-1404
237. AEAARL V4YK78 V4YK78_TOXGO Uncharacterized protein 4948 983-988
238. AEAARL V4YS95 V4YS95_TOXGO Uncharacterized protein 742 548-553
239. AEAARL V4Z3J8 V4Z3J8_TOXGO Gtr1/RagA protein G 676 299-304
240. AEAARL V4ZAD9 V4ZAD9_TOXGO Protein kinase domain protein 789 722-727
241. AEAARL V4ZD18 V4ZD18_TOXGO Uncharacterized protein 3221 2547-2552
242. AEAARL V4ZN88 V4ZN88_TOXGO Uncharacterized protein 2946 1907-1912
243. AEAARL V4ZNF6 V4ZNF6_TOXGO Putative glutamic acid-rich protein 2698 1064-1069
244. EAARLG B9QP11 B9QP11_TOXGO CMGC kinase, CK2 family 539 248-253
245. AARLGP V4ZIB8 V4ZIB8_TOXGO Phosphodiesterase/alkaline phosphatase D family protein 762 625-630
246. RLGPAV B9QJQ0 B9QJQ0_TOXGO Uncharacterized protein 1172 429-434
247. LGPAVA B6KQU6 B6KQU6_TOXGO Cytoadherence-linked asexual protein 1490 1346-1351
248. LGPAVA B9QJQ0 B9QJQ0_TOXGO Uncharacterized protein 1172 430-435
249. LGPAVA Q1JTB2 Q1JTB2_TOXGO Putative uncharacterized protein precursor 1453 1309-1314
250. LGPAVA V4Z3C9 V4Z3C9_TOXGO Uncharacterized protein 1899 1205-1210
251. GPAVAA B6KU18 B6KU18_TOXGO 3-ketoacyl-(Acyl-carrier-protein) reductase 376 193-198
252. GPAVAA V4Z3C9 V4Z3C9_TOXGO Uncharacterized protein 1899 1206-1211
253. PAVAAA A8CBF6 A8CBF6_TOXGO Delta-aminolevulinic acid synthetase 584 424-429
254. PAVAAA B9PJ56 B9PJ56_TOXGO Calcium binding egf domain-containing protein 302 287-292
255. PAVAAA B9Q562 B9Q562_TOXGO Non-specific serine/threonine protein kinase 8428 68-73
256. PAVAAA V4YXZ7 V4YXZ7_TOXGO NLI interacting factor family phosphatase 2212 1842-1847
257. PAVAAA V4Z106 V4Z106_TOXGO Uncharacterized protein 2597 1059-1064
258. PAVAAA V4Z3C9 V4Z3C9_TOXGO Uncharacterized protein 1899 1207-1212
259. PAVAAA V4Z6D2 V4Z6D2_TOXGO Uncharacterized protein 1534 1385-1390
260. PAVAAA V4Z7C7 V4Z7C7_TOXGO Polynucleotide adenylyltransferase 835 206-211
261. PAVAAA V4ZIT3 V4ZIT3_TOXGO 5-aminolevulinic acid synthase domain-containing protein 752 487-492
262. PAVAAA V4ZIV2 V4ZIV2_TOXGO RNA pseudouridine synthase superfamily protein 1534 446-451
263. PAVAAA V5BMD9 V5BMD9_TOXGO UBA/TS-N domain-containing protein 7817 3418-3423
264. AVAAAV B9QFW3 B9QFW3_TOXGO AP2 domain transcription factor AP2VIIa-7 3112 2131-2136
265. AVAAAV B9QP50 B9QP50_TOXGO Putative transmembrane protein 340 230-235
266. AVAAAV Q1JT62 Q1JT62_TOXGO Putative uncharacterized protein precursor 1122 682-687
267. AVAAAV V4YKW6 V4YKW6_TOXGO Putative transmembrane protein 256 3-8
268. AVAAAV V4YYK4 V4YYK4_TOXGO ATP-dependent metallopeptidase HflB subfamily protein 1005 955-960
269. AVAAAV V4YZY2 V4YZY2_TOXGO ATPase (DUF699) protein 1247 346-351
270. AVAAAV V4Z0I9 V4Z0I9_TOXGO LETM1 family protein 1101 630-635
271. AVAAAV V4Z5K2 V4Z5K2_TOXGO Uncharacterized protein 1183 682-687
272. AVAAAV V4ZAN5 V4ZAN5_TOXGO Serine/threonine specific protein phosphatase 2462 2390-2395
273. AVAAAV V4ZPK3 V4ZPK3_TOXGO Uncharacterized protein 1917 534-539
274. AVAAAV V4ZPZ2 V4ZPZ2_TOXGO ARID/BRIGHT DNA binding domain-containing protein 2713 2566-2571
275. AVAAAV V5B7V9 V5B7V9_TOXGO L1P family of ribosomal protein 928 462-467
276. AVAAAV V5BGF0 V5BGF0_TOXGO Uncharacterized protein 101 36-41
277. VAAAVR Q1JTJ3 Q1JTJ3_TOXGO SET-domain protein, putative 4382 1400-1405
278. VAAAVR V4YK78 V4YK78_TOXGO Uncharacterized protein 4948 3532-3537
279. VAAAVR V4Z559 V4Z559_TOXGO Putative histone lysine methyltransferase, SET 5175 1400-1405
280. VAAAVR V4Z9X5 V4Z9X5_TOXGO Uncharacterized protein 4983 367-372
281. VAAAVR V4ZLF7 V4ZLF7_TOXGO Putative O-acetylserine (Thiol) lyase 2 585 495-500
282. AAAVRS V4ZBJ0 V4ZBJ0_TOXGO Uncharacterized protein 3206 1347-1352
283. AAAVRS V4ZIE9 V4ZIE9_TOXGO HEAT repeat-containing protein 3443 1937-1942
284. AAAVRS V4ZMH5 V4ZMH5_TOXGO Uncharacterized protein 394 215-220
285. VRSPGL B6K8Q8 B6K8Q8_TOXGO Plasma membrane-type Ca(2+)-ATPase A1 PMCAA1 1822 28-33
286. VRSPGL B6KJ35 B6KJ35_TOXGO MaoC family domain-containing protein 324 314-319
287. VRSPGL V5B555 V5B555_TOXGO Putative AAA family domain ATPase 2965 822-827
288. PGLDVR B6KPA4 B6KPA4_TOXGO Uncharacterized protein 416 250-255
289. PGLDVR Q1JSK7 Q1JSK7_TOXGO Uncharacterized protein precursor 416 250-255
290. DVRPVA V4Z7S0 V4Z7S0_TOXGO Toxoplasma gondii family E protein 530 96-101
291. VRPVAL B6K9K0 B6K9K0_TOXGO Putative saccharopine dehydrogenase 537 419-424
292. VRPVAL B6KUG4 B6KUG4_TOXGO Uncharacterized protein 1979 1824-1829
293. PVALVL V5BLY3 V5BLY3_TOXGO DALR anticodon binding domain-containing protein 1307 611-616
294. GSDPRS B9PJE6 B9PJE6_TOXGO IMC sub-compartment protein ISP3 164 96-101
295. GSDPRS V4ZBR9 V4ZBR9_TOXGO Protein kinase 1763 851-856
296. DPRSLV V4Z3A9 V4Z3A9_TOXGO Putative large protein with signal peptide cysteine-rich,threonine-rich, mucin 1092 172-177
297. LQLCDL V4ZKU2 V4ZKU2_TOXGO WD domain, G-beta repeat-containing protein 1632 537-542
298. CDLLSG V4ZIK8 V4ZIK8_TOXGO Putative acyl-CoA thioesterase 516 95-100
299. DLLSGL V4Z8L2 V4Z8L2_TOXGO Uncharacterized protein 4801 3899-3904
300. LLSGLR B6K9U0 B6K9U0_TOXGO XRN 5'-3' exonuclease N-terminus protein 2089 1229-1234
301. LLSGLR V4Z8L2 V4Z8L2_TOXGO Uncharacterized protein 4801 3900-3905
302. LLSGLR V4ZBS7 V4ZBS7_TOXGO B-block-binding subunit of tfiiic protein 4096 1163-1168
303. LLSGLR V4ZNX9 V4ZNX9_TOXGO Putative histone kinase SNF1 1947 221-226
304. LRVHGV V4ZRU8 V4ZRU8_TOXGO BTB/POZ domain-containing protein 1289 463-468
305. VHGVVF B6KAV9 B6KAV9_TOXGO FYVE zinc finger domain-containing protein 834 475-480
306. VFEDDS V4ZJY9 V4ZJY9_TOXGO Pre-rRNA processing protein 2863 2410-2415
307. EDDSRA V5B4D7 V5B4D7_TOXGO Spc97/Spc98 family protein 2870 139-144
308. SRAPAV B9Q722 B9Q722_TOXGO Apicomplexan specific, related protein 1003 828-833
309. SRAPAV B9QHZ2 B9QHZ2_TOXGO tRNA pseudouridine synthase 1512 1341-1346
310. RAPAVA V5B4T5 V5B4T5_TOXGO Surface antigen repeat-containing protein 2316 757-762
311. APAVAP B6KGG5 B6KGG5_TOXGO Diacylglycerol kinase 1841 1430-1435
312. APAVAP B9QF52 B9QF52_TOXGO Putative transmembrane protein 805 197-202
313. APAVAP V4Z316 V4Z316_TOXGO Uncharacterized protein 1343 126-131
314. APAVAP V4Z7Q1 V4Z7Q1_TOXGO Zinc finger domain, LSD1 subclass domain-containing protein 1059 439-444
315. APAVAP V4ZP03 V4ZP03_TOXGO Uncharacterized protein 1408 804-809
316. APAVAP V4ZUE4 V4ZUE4_TOXGO LNS2 (Lipin/Ned1/Smp2) protein 1575 234-239
317. LSAQTS V4ZTD6 V4ZTD6_TOXGO Kinesin motor domain-containing protein 594 483-488
318. LSAQTS V5BIT9 V5BIT9_TOXGO Uncharacterized protein 2397 1104-1109
319. SAQTSL B6KAX6 B6KAX6_TOXGO Zinc finger (CCCH type) motif-containing protein 822 500-505
320. SLPIVA V4ZLH0 V4ZLH0_TOXGO Putative transmembrane protein 1257 1251-1256
321. IVAVHG Q969A2 Q969A2_TOXGO Pyruvate kinase 531 508-513
322. AVHGGA V4YW89 V4YW89_TOXGO Sushi domain (Scr repeat) domain-containing protein 4752 3214-3219
323. VHGGAA B9Q553 B9Q553_TOXGO Uncharacterized protein 2284 1512-1517
324. VHGGAA V5BDM3 V5BDM3_TOXGO Sec7 domain-containing protein 3987 900-905
325. GGAALV B6KDK3 B6KDK3_TOXGO Radical SAM methylthiotransferase, MiaB/RimO family protein 867 21-26
326. GGAALV B9Q4U1 B9Q4U1_TOXGO Amine-terminal region of chorein, A TM vesicle-mediated sorter 10329 3834-3839
327. GGAALV V4Z611 V4Z611_TOXGO Putative transmembrane protein 1126 421-426
328. GGAALV V4ZPK3 V4ZPK3_TOXGO Uncharacterized protein 1917 887-892
329. GGAALV V5B2X1 V5B2X1_TOXGO Uncharacterized protein 4035 524-529
330. GAALVL B6KDQ2 B6KDQ2_TOXGO Putative acetyl-CoA acyltransferase B 418 276-281
331. GAALVL B9QP15 B9QP15_TOXGO UDP-galactose transporter subfamily protein 394 321-326
332. GAALVL Q1JSC8 Q1JSC8_TOXGO Uncharacterized protein 734 662-667
333. GAALVL V4YPW3 V4YPW3_TOXGO Peptidase M16 inactive domain-containing protein 1604 1047-1052
334. GAALVL V4Z7P7 V4Z7P7_TOXGO Putative transmembrane protein 217 45-50
335. AALVLT B9QP15 B9QP15_TOXGO UDP-galactose transporter subfamily protein 394 322-327
336. AALVLT V4ZJN0 V4ZJN0_TOXGO MCM2/3/5 family protein 1238 723-728
337. ALVLTP V4ZDH7 V4ZDH7_TOXGO Uncharacterized protein 5043 560-565
338. LVLTPK V4ZCV3 V4ZCV3_TOXGO Amine-terminal region of chorein, A TM vesicle-mediated sorter 9741 7047-7052
339. LTPKEK B9PLW6 B9PLW6_TOXGO Putative transmembrane protein 407 264-269
340. TPKEKG V4Z8D7 V4Z8D7_TOXGO Putative eukaryotic initiation factor-2B, gamma subunit 525 47-52
341. PKEKGS V5BM22 V5BM22_TOXGO WD domain, G-beta repeat-containing protein 2869 1782-1787
342. STFLQL A3RJI7 A3RJI7_TOXGO ATP-binding cassette protein subfamily B member 2 1407 780-785
343. STFLQL B6E3J6 B6E3J6_TOXGO ATP-binding cassette sub-family B member 2 1407 780-785
344. STFLQL D0EP43 D0EP43_TOXGO ATP-binding cassette protein subfamily B member 2 1407 780-785
345. STFLQL Q4FCM1 Q4FCM1_TOXGO ABC transporter transmembrane region domain-containing protein 1407 780-785
346. QLGSST V4ZM77 V4ZM77_TOXGO Putative transmembrane protein 1060 338-343
347. LGSSTE B6K9M7 AMA1L_TOXGO Apical membrane antigen 1-like protein precursor 651 430-435
348. LGSSTE B9PUA3 B9PUA3_TOXGO Uncharacterized protein 429 185-190
349. LGSSTE V4YWF6 V4YWF6_TOXGO SAG-related sequence SRS40F 390 144-149
350. LGSSTE V4Z2H8 V4Z2H8_TOXGO Uncharacterized protein 984 880-885
351. VIFEVL B9Q6F6 B9Q6F6_TOXGO Putative heat shock protein hslv 338 301-306
352. IFEVLE B9Q6F6 B9Q6F6_TOXGO Putative heat shock protein hslv 338 302-307
353. IFEVLE V5BMA5 V5BMA5_TOXGO Uncharacterized protein 2399 838-843
354. FEVLEE V5BMA5 V5BMA5_TOXGO Uncharacterized protein 2399 839-844
355. TSFVAV V4ZG45 V4ZG45_TOXGO Putative fatty acyl-CoA desaturase 1042 587-592
356. SFVAVT V5B211 V5B211_TOXGO Uncharacterized protein 70 48-53
357. VAVTTR V4ZL76 V4ZL76_TOXGO WD domain, G-beta repeat-containing protein 1802 1616-1621
358. AVTTRA B9QDW1 B9QDW1_TOXGO ATP-binding cassette G family transporter ABCG96 1064 25-30
359. AVTTRA V4ZER8 V4ZER8_TOXGO Uncharacterized protein 4402 3795-3800
360. VTTRAP V4YV25 V4YV25_TOXGO Putative transmembrane protein 726 493-498
361. TTRAPG B9Q661 B9Q661_TOXGO Uncharacterized protein 357 209-214
362. VLTDGS V5BD96 V5BD96_TOXGO Fumarate hydratase 776 351-356
363. LTDGSL B6KM84 B6KM84_TOXGO Deoxyhypusine synthase 457 327-332
364. TDGSLV V4YN30 V4YN30_TOXGO Putative vacuolar protein sorting-associated protein 8650 6112-6117
365. DGSLVG V4ZBW6 V4ZBW6_TOXGO WD domain, G-beta repeat-containing protein 461 381-386
366. GSLVGW V4ZBW6 V4ZBW6_TOXGO WD domain, G-beta repeat-containing protein 461 382-387
367. EHRGAL V4Z6Y4 V4Z6Y4_TOXGO SWI2/SNF2-containing protein RAD5 1748 702-707
368. EHRGAL V4ZIY3 V4ZIY3_TOXGO Rhoptry kinase family protein ROP27 975 572-577
369. RGALTL B9QQF1 B9QQF1_TOXGO Proteasome/cyclosome repeat-containing protein 3187 1350-1355
370. RGALTL V4ZSA9 V4ZSA9_TOXGO Putative transmembrane protein 648 186-191
371. GALTLD V4YN30 V4YN30_TOXGO Putative vacuolar protein sorting-associated protein 8650 8380-8385
372. PGAGEA B6K924 B6K924_TOXGO Leucine rich repeat protein 1687 139-144
373. PGAGEA B6KFK7 B6KFK7_TOXGO 3'5'-cyclic nucleotide phosphodiesterase domain-containing protein 1281 35-40
374. PGAGEA V4YUB6 V4YUB6_TOXGO Uncharacterized protein 1153 511-516
375. PGAGEA V4ZPY4 V4ZPY4_TOXGO RAVE 1 carboxy-terminal protein 6665 977-982
376. GAGEAV Q6JD66 Q6JD66_TOXGO Eukaryotic initiation factor-2 alpha kinase-A 5072 996-1001
377. GAGEAV V4YQT3 V4YQT3_TOXGO Putative transmembrane protein 3329 1383-1388
378. GAGEAV V4YUB6 V4YUB6_TOXGO Uncharacterized protein 1153 512-517
379. GAGEAV V4Z8B4 V4Z8B4_TOXGO Putative thioredoxin 711 172-177
380. GAGEAV V4ZSE9 V4ZSE9_TOXGO CMGC kinase, MAPK family (ERK) MAPK-1 1298 860-865
381. GAGEAV V4ZU83 V4ZU83_TOXGO eIF2 kinase IF2K-A (Incomplete catalytic triad) 4638 562-567
382. AGEAVL B9Q5J0 B9Q5J0_TOXGO Uncharacterized protein 2385 2330-2335
383. AGEAVL V4YN30 V4YN30_TOXGO Putative vacuolar protein sorting-associated protein 8650 3463-3468
384. GEAVLS B9Q5J0 B9Q5J0_TOXGO Uncharacterized protein 2385 2331-2336
385. GEAVLS V4Z3X0 V4Z3X0_TOXGO HEAT repeat-containing protein 1010 345-350
386. EAVLSA B9Q5R9 B9Q5R9_TOXGO RAP domain-containing protein 863 170-175
387. EAVLSA Q1JSF3 Q1JSF3_TOXGO Uncharacterized protein 4600 2756-2761
388. EAVLSA V4YLC6 V4YLC6_TOXGO Uncharacterized protein 92 5-10
389. EAVLSA V4YZC1 V4YZC1_TOXGO Uncharacterized protein 3026 1787-1792
390. EAVLSA V4Z6H5 V4Z6H5_TOXGO Putative glutamic acid-rcih protein 4436 2281-2286
391. AVLSAQ Q1JSF3 Q1JSF3_TOXGO Uncharacterized protein 4600 2757-2762
392. AVLSAQ V4Z6H5 V4Z6H5_TOXGO Putative glutamic acid-rcih protein 4436 2282-2287
393. LSAQLR V4YVZ3 V4YVZ3_TOXGO COG4 transport protein 1178 1009-1014
394. LSAQLR V4ZI21 V4ZI21_TOXGO Putative related MUS81 endonuclease 1415 843-848
395. QLRSVS V4Z8L2 V4Z8L2_TOXGO Uncharacterized protein 4801 440-445
396. LRSVSA V4YY43 V4YY43_TOXGO Putative transmembrane protein 862 849-854
397. LRSVSA V4YYG6 V4YYG6_TOXGO Putative zinc finger protein 2469 1123-1128
398. LRSVSA V4ZHW2 V4ZHW2_TOXGO Uncharacterized protein 681 296-301
399. RSVSAQ V4ZB28 V4ZB28_TOXGO Uncharacterized protein 1836 1355-1360
400. SVSAQI V5B7Q8 V5B7Q8_TOXGO Pentatricopeptide repeat domain-containing protein 1422 412-417
401. VSAQIR V5B7Q8 V5B7Q8_TOXGO Pentatricopeptide repeat domain-containing protein 1422 413-418
402. SAQIRL V5B7Q8 V5B7Q8_TOXGO Pentatricopeptide repeat domain-containing protein 1422 414-419
403. LLFCAR V5BLW2 V5BLW2_TOXGO Uncharacterized protein 1741 883-888
404. FCAREE B6KVH0 B6KVH0_TOXGO Histone methylation protein DOT1 2377 1941-1946
405. CAREEA B6KA26 B6KA26_TOXGO Adaptin n terminal region domain-containing protein 1355 127-132
406. AREEAE B9Q832 B9Q832_TOXGO Uncharacterized protein 899 606-611
407. AREEAE B9QKP8 B9QKP8_TOXGO Radical SAM domain-containing protein 619 157-162
408. AREEAE B9QNS3 B9QNS3_TOXGO Uncharacterized protein 2165 930-935
409. AREEAE Q1JSG1 Q1JSG1_TOXGO Uncharacterized protein 641 188-193
410. AREEAE Q7Z289 Q7Z289_TOXGO GAP45 245 122-127
411. AREEAE V4YZ36 V4YZ36_TOXGO HEAT repeat-containing protein 3407 841-846
412. AREEAE V4Z6T1 V4Z6T1_TOXGO Dopey, N-terminal domain-containing protein 3103 585-590
413. AREEAE V4ZHL8 V4ZHL8_TOXGO HECT-domain (Ubiquitin-transferase) domain protein 762 714-719
414. AREEAE V5B176 V5B176_TOXGO DEAD/DEAH box helicase domain-containing protein 1084 204-209
415. AREEAE V5B4T5 V5B4T5_TOXGO Surface antigen repeat-containing protein 2316 1334-1339
416. AREEAE V5BM94 V5BM94_TOXGO Uncharacterized protein 3520 784-789
417. REEAEP B9QNS3 B9QNS3_TOXGO Uncharacterized protein 2165 931-936
418. EPVFRA V4ZGX1 V4ZGX1_TOXGO Uncharacterized protein 3917 3747-3752
419. VFRAAE B6KGT6 B6KGT6_TOXGO Uncharacterized protein 502 371-376
420. FRAAEE B6KGT6 B6KGT6_TOXGO Uncharacterized protein 502 372-377
421. RAAEEA B6KB66 B6KB66_TOXGO Uncharacterized protein 538 260-265
422. RAAEEA B9QIB9 B9QIB9_TOXGO Uncharacterized protein 964 199-204
423. RAAEEA B9QMF6 B9QMF6_TOXGO CAM kinase, CDPK family 1388 745-750
424. RAAEEA V4YS05 V4YS05_TOXGO Putative transmembrane protein 2088 1900-1905
425. RAAEEA V4YZ89 V4YZ89_TOXGO Putative myosin heavy chain 508 93-98
426. RAAEEA V4Z4L9 V4Z4L9_TOXGO Uncharacterized protein 583 403-408
427. RAAEEA V4Z7X7 V4Z7X7_TOXGO HAD hydrolase, family IIID protein 661 650-655
428. RAAEEA V4Z952 V4Z952_TOXGO Uncharacterized protein 4210 311-316
429. RAAEEA V4ZC21 V4ZC21_TOXGO Uncharacterized protein 1167 1108-1113
430. RAAEEA V4ZFW7 V4ZFW7_TOXGO Uncharacterized protein 2647 2165-2170
431. RAAEEA V4ZJ82 V4ZJ82_TOXGO Uncharacterized protein 1881 897-902
432. RAAEEA V4ZJW7 V4ZJW7_TOXGO Sma protein 2746 1747-1752
433. AAEEAG K7WFS9 K7WFS9_TOXGO DHHC11 944 718-723
434. AAEEAG V4YMY1 V4YMY1_TOXGO Tetratricopeptide repeat-containing protein 1008 30-35
435. AAEEAG V4Z026 V4Z026_TOXGO Electron transfer flavoprotein-ubiquinone oxidoreductase 1035 236-241
436. AAEEAG V4Z518 V4Z518_TOXGO Acetyltransferase, GNAT family protein 519 141-146
437. AAEEAG V4Z994 V4Z994_TOXGO DHHC zinc finger domain-containing protein 951 725-730
438. AAEEAG V4ZG71 V4ZG71_TOXGO Putative transmembrane protein 699 319-324
439. AEEAGL B6K9V5 B6K9V5_TOXGO Uncharacterized protein 1315 848-853
440. AEEAGL B9Q657 B9Q657_TOXGO ATPase, AAA family protein 1600 1434-1439
441. AEEAGL B9QAU9 B9QAU9_TOXGO Gorasp2-prov protein 475 35-40
442. AEEAGL V5BCP0 V5BCP0_TOXGO Putative transmembrane protein 959 886-891
443. GLTGSG B9QJX3 B9QJX3_TOXGO Uncharacterized protein 4533 748-753
444. GLTGSG V5BBZ5 V5BBZ5_TOXGO Calcium binding egf domain-containing protein 1078 226-231
445. VGPQLA B9Q792 B9Q792_TOXGO Uncharacterized protein 4118 3113-3118
446. GPQLAG B9Q792 B9Q792_TOXGO Uncharacterized protein 4118 3114-3119
447. GPQLAG V4Z406 V4Z406_TOXGO AP2 domain transcription factor AP2XI-5 868 169-174
448. PQLAGG B9Q792 B9Q792_TOXGO Uncharacterized protein 4118 3115-3120
449. PQLAGG V5BBJ6 V5BBJ6_TOXGO Uncharacterized protein 139 45-50
450. QLAGGG V5BBJ6 V5BBJ6_TOXGO Uncharacterized protein 139 46-51
451. LAGGGG V4Z108 V4Z108_TOXGO EKN1, related protein 806 386-391
452. AGGGGS B6KTZ8 B6KTZ8_TOXGO RNA recognition motif-containing protein 351 9-14
453. AGGGGS B9Q7B5 B9Q7B5_TOXGO TLD protein 2195 540-545
454. AGGGGS B9QKB4 B9QKB4_TOXGO 5'-nucleotidase, C-terminal domain-containing protein 884 10-15
455. AGGGGS Q1JTJ7 Q1JTJ7_TOXGO Putative uncharacterized protein precursor 1839 15-20
456. AGGGGS V5AZI6 V5AZI6_TOXGO C2 domain-containing protein 1043 15-20
457. GGGGSG B6KTZ8 B6KTZ8_TOXGO RNA recognition motif-containing protein 351 10-15
458. GGGGSG B9PJF1 B9PJF1_TOXGO Putative transmembrane protein 131 112-117
459. GGGGSG B9PRZ6 B9PRZ6_TOXGO Ras family protein 257 16-21
460. GGGGSG B9PSA7 B9PSA7_TOXGO Putative kelch repeat protein 625 600-605
461. GGGGSG B9QMH6 B9QMH6_TOXGO RNA pseudouridine synthase superfamily protein 6535 289-294
462. GGGGSG V4YU53 V4YU53_TOXGO Uncharacterized protein 2302 1683-1688
463. GGGGSG V4Z1G0 V4Z1G0_TOXGO AP2 domain transcription factor AP2XII-2 1737 1397-1402
464. GGGGSG V4Z585 V4Z585_TOXGO ThiF family protein 2933 1336-1341
465. GGGGSG V4ZCQ1 V4ZCQ1_TOXGO CW-type Zinc Finger protein 1296 1220-1225
466. GGGGSG V4ZFW3 V4ZFW3_TOXGO Putative DNA methyltransferase 2 831 431-436
467. GGGSGA B6KBJ8 B6KBJ8_TOXGO SAC3/GANP family protein 975 244-249
468. GGGSGA B9PSA7 B9PSA7_TOXGO Putative kelch repeat protein 625 601-606
469. GGGSGA V4YU53 V4YU53_TOXGO Uncharacterized protein 2302 1684-1689
470. GGGSGA V4YUB6 V4YUB6_TOXGO Uncharacterized protein 1153 719-724
471. GGGSGA V4ZCQ1 V4ZCQ1_TOXGO CW-type Zinc Finger protein 1296 1221-1226
472. GGGSGA V4ZEM1 V4ZEM1_TOXGO Protein phosphatase 2C domain-containing protein 1089 1056-1061
473. GGSGAP B6KFK7 B6KFK7_TOXGO 3'5'-cyclic nucleotide phosphodiesterase domain-containing protein 1281 1173-1178
474. GGSGAP B6KJP5 B6KJP5_TOXGO Pre-mRNA processing splicing factor PRP8 2538 127-132
475. GGSGAP B9QNL6 B9QNL6_TOXGO Uncharacterized protein 560 248-253
476. GGSGAP V4ZFW7 V4ZFW7_TOXGO Uncharacterized protein 2647 888-893
477. GGSGAP V5BAB1 V5BAB1_TOXGO RNA recognition motif-containing protein 539 425-430
478. GSGAPG B6KH44 B6KH44_TOXGO Putative ribosomal RNA (Adenine(1779)-N(6)/adenine(1780)-N(6))-dimethyltransferase 494 463-468
479. GSGAPG B9PS36 B9PS36_TOXGO Putative transport protein Sec24 1019 545-550
480. GSGAPG B9QK32 B9QK32_TOXGO Putative coatomer protein complex, subunit alpha 1300 913-918
481. GSGAPG V4YKX4 V4YKX4_TOXGO Serine/threonine specific protein phosphatase 2883 355-360
482. GSGAPG V4YPC7 V4YPC7_TOXGO Putative ppg3 1940 573-578
483. GSGAPG V4Z6S4 V4Z6S4_TOXGO Ubiquitin carboxyl-terminal hydrolase 3600 829-834
484. GSGAPG V4ZMJ1 V4ZMJ1_TOXGO Uncharacterized protein 4969 4824-4829
485. GSGAPG V4ZPK7 V4ZPK7_TOXGO Sec20 protein 808 9-14
486. GSGAPG V5AXN6 V5AXN6_TOXGO Putative NAD(P) transhydrogenase subunit beta 1244 590-595
487. SGAPGE B9PY21 B9PY21_TOXGO ACR-like protein 1530 484-489
488. SGAPGE V4ZD09 V4ZD09_TOXGO WD domain, G-beta repeat-containing protein 752 405-410
489. SGAPGE V4ZEF2 V4ZEF2_TOXGO AP2 domain transcription factor AP2IX-3 3096 918-923
490. SGAPGE V4ZIP7 V4ZIP7_TOXGO Eukaryotic initiation factor 4E 2044 1848-1853
491. SGAPGE V4ZPK7 V4ZPK7_TOXGO Sec20 protein 808 10-15
492. SGAPGE V5AXN6 V5AXN6_TOXGO Putative NAD(P) transhydrogenase subunit beta 1244 591-596
493. GAPGEP V4Z9Z0 V4Z9Z0_TOXGO Uncharacterized protein 1080 908-913
494. GAPGEP V4ZEF2 V4ZEF2_TOXGO AP2 domain transcription factor AP2IX-3 3096 919-924
495. APGEPP V4YKE7 V4YKE7_TOXGO Calcium-dependent protein kinase CDPK6 1477 418-423
496. APGEPP V4ZL32 V4ZL32_TOXGO Uncharacterized protein 1689 845-850
497. EPPLLP B6KFA4 B6KFA4_TOXGO Uncharacterized protein 843 532-537
498. PPLLPG Q1JSL3 Q1JSL3_TOXGO mRNA decapping enzyme, putative precursor 512 388-393
499. PPLLPG V4YMB2 V4YMB2_TOXGO Putative mRNA decapping enzyme 400 276-281
500. PPLLPG V4YZ80 V4YZ80_TOXGO HECT-domain (Ubiquitin-transferase) domain-containing protein 15897 2834-2839
501. PPLLPG V4ZF71 V4ZF71_TOXGO Tyrosine kinase-like (TKL) protein 2021 371-376
502. PPLLPG V4ZIZ2 V4ZIZ2_TOXGO Putative collagen alpha-1(III) chain, related protein 2799 2604-2609
503. PPLLPG V4ZN59 V4ZN59_TOXGO Uncharacterized protein 361 346-351
504. PLLPGG B6K924 B6K924_TOXGO Leucine rich repeat protein 1687 96-101
505. PLLPGG Q1JSL3 Q1JSL3_TOXGO mRNA decapping enzyme, putative precursor 512 389-394
506. PLLPGG V4YMB2 V4YMB2_TOXGO Putative mRNA decapping enzyme 400 277-282
507. PLLPGG V4YMZ0 V4YMZ0_TOXGO Sushi domain (Scr repeat) domain-containing protein 3916 808-813
508. PLLPGG V4YZ80 V4YZ80_TOXGO HECT-domain (Ubiquitin-transferase) domain-containing protein 15897 2835-2840
509. PLLPGG V4Z4M8 V4Z4M8_TOXGO Amine-terminal region of chorein, A TM vesicle-mediated sorter 13455 12826-12831
510. PLLPGG V4Z8G0 V4Z8G0_TOXGO Guanylyl cyclase 4367 589-594
511. PLLPGG V4ZC18 V4ZC18_TOXGO Uncharacterized protein 2162 126-131
512. LLPGGA B6KAZ7 B6KAZ7_TOXGO Myosin J 2532 1884-1889
513. LLPGGA V4YMZ0 V4YMZ0_TOXGO Sushi domain (Scr repeat) domain-containing protein 3916 809-814
514. LLPGGA V4Z4M8 V4Z4M8_TOXGO Amine-terminal region of chorein, A TM vesicle-mediated sorter 13455 12827-12832
515. LPGGAP B9PTV9 B9PTV9_TOXGO RuvB family 2 protein 508 463-468
516. LPGGAP B9QFQ5 B9QFQ5_TOXGO Uncharacterized protein 1798 516-521
517. LPGGAP V4YND4 V4YND4_TOXGO eIF2 kinase IF2K-D (Incomplete catalytic triad) 2866 2682-2687
518. LPGGAP V4ZR42 V4ZR42_TOXGO Uncharacterized protein 1471 1175-1180
519. LPGGAP V5B4N7 V5B4N7_TOXGO Uncharacterized protein 1005 212-217
520. PGGAPL V4Z445 V4Z445_TOXGO Histone acetyltransferase TAF1/250 2775 137-142
521. PGGAPL V5B4N7 V5B4N7_TOXGO Uncharacterized protein 1005 213-218
522. GGAPLP B9PMK8 B9PMK8_TOXGO Transporter, major facilitator family protein 589 531-536
523. GGAPLP V5BDC0 V5BDC0_TOXGO Uncharacterized protein 1391 868-873
524. GAPLPA B6KRB0 B6KRB0_TOXGO RNA polymerase II accessory factor CDC73 720 160-165
525. GAPLPA V4ZIE9 V4ZIE9_TOXGO HEAT repeat-containing protein 3443 1928-1933
526. GAPLPA V4ZPH3 V4ZPH3_TOXGO Protein kinase domain protein 2329 430-435
527. GAPLPA V4ZPK3 V4ZPK3_TOXGO Uncharacterized protein 1917 75-80
528. APLPAG V4YXX0 V4YXX0_TOXGO Putative Elicitor-responsive protein 306 229-234
529. APLPAG V4Z6C0 V4Z6C0_TOXGO LsmAD domain-containing protein 1524 1129-1134
530. PLPAGL B6KDN0 B6KDN0_TOXGO Eukaryotic translation initiation factor 3 subunit E 601 280-285
531. PLPAGL V4Z3R4 V4Z3R4_TOXGO U1 zinc finger protein 567 141-146
532. PLPAGL V4ZAP5 V4ZAP5_TOXGO Zinc finger, C3HC4 type (RING finger) domain-containing protein 3893 2990-2995
533. PLPAGL V4ZKI0 V4ZKI0_TOXGO Uncharacterized protein 964 329-334
534. LPAGLF V4YQT3 V4YQT3_TOXGO Putative transmembrane protein 3329 2873-2878
535. PAGLFA V4Z8X8 V4Z8X8_TOXGO Uncharacterized protein 1707 1074-1079
536. LFAVRS B9QHE7 B9QHE7_TOXGO Putative transmembrane protein 536 349-354
537. AVRSAG B9PTV9 B9PTV9_TOXGO RuvB family 2 protein 508 475-480
538. AVRSAG B9Q553 B9Q553_TOXGO Uncharacterized protein 2284 1876-1881
539. AVRSAG V4ZBI1 V4ZBI1_TOXGO Putative GDP mannose 4,6-dehydratase 251 114-119
540. AVRSAG V5BB50 V5BB50_TOXGO Uncharacterized protein 2115 1806-1811
541. RSAGWR B6KR46 B6KR46_TOXGO Putative phospholipase 594 281-286
542. SAGWRD V4ZEI6 V4ZEI6_TOXGO Kelch repeat-containing protein 2049 646-651
543. RDDLAR V4YKJ6 V4YKJ6_TOXGO Uncharacterized protein 180 139-144
544. DLARRV B9QL03 B9QL03_TOXGO Putative myosin heavy chain 1124 686-691
545. DLARRV Q1JSR7 Q1JSR7_TOXGO Uncharacterized protein 931 493-498
546. DLARRV V4Z6H7 V4Z6H7_TOXGO UvrD/REP helicase domain-containing protein 3190 3084-3089
547. LARRVA B9Q6P1 B9Q6P1_TOXGO Putative transmembrane protein 760 636-641
548. LARRVA B9QI34 B9QI34_TOXGO Helicase associated domain (Ha2) protein 2234 359-364
549. LARRVA V4Z5A8 V4Z5A8_TOXGO Uncharacterized protein 1237 911-916
550. LARRVA V4Z6H7 V4Z6H7_TOXGO UvrD/REP helicase domain-containing protein 3190 3085-3090
551. LARRVA V5B7V4 V5B7V4_TOXGO tRNA pseudouridine synthase D 1489 1172-1177
552. ARRVAA V4Z685 V4Z685_TOXGO Uncharacterized protein 698 13-18
553. ARRVAA V4ZFR1 V4ZFR1_TOXGO HEAT repeat-containing protein 1697 1147-1152
554. ARRVAA V4ZG06 V4ZG06_TOXGO Uncharacterized protein 2320 1705-1710
555. ARRVAA V4ZH67 V4ZH67_TOXGO Uncharacterized protein 3076 1076-1081
556. ARRVAA V5B7V4 V5B7V4_TOXGO tRNA pseudouridine synthase D 1489 1173-1178
557. RRVAAG B9Q568 B9Q568_TOXGO XPG N-terminal domain-containing protein 2004 15-20
558. RVAAGV B9QHN7 B9QHN7_TOXGO Fop carboxy-terminal duplication domain protein 248 213-218
559. RVAAGV B9QLZ6 B9QLZ6_TOXGO Putative transmembrane protein 606 549-554
560. VAAGVA B6KAG9 B6KAG9_TOXGO U3 small nucleolar RNA-associated protein 10 3738 1059-1064
561. VAAGVA B6KRL4 B6KRL4_TOXGO Alpha/beta hydrolase family protein 1376 369-374
562. VAAGVA Q1JTA6 Q1JTA6_TOXGO Putative uncharacterized protein 304 270-275
563. VAAGVA V4ZJ48 V4ZJ48_TOXGO NAD/NADP octopine/nopaline dehydrogenase, alpha-helical domain-containing protein 435 7-12
564. VAAGVA V4ZL48 V4ZL48_TOXGO Dolichyl-diphosphooligosaccharide--protein glycosyltransferase subunit 1 955 356-361
565. VAAGVA V5B047 V5B047_TOXGO Putative transmembrane protein 345 311-316
566. VAAGVA V5BGJ9 V5BGJ9_TOXGO Uncharacterized protein 818 734-739
567. AAGVAV Q1JTA6 Q1JTA6_TOXGO Putative uncharacterized protein 304 271-276
568. AAGVAV V5B047 V5B047_TOXGO Putative transmembrane protein 345 312-317
569. AAGVAV V5B6P1 V5B6P1_TOXGO Putative galactosyltransferase 441 330-335
570. AGVAVV B6KRG4 B6KRG4_TOXGO Uncharacterized protein 1174 332-337
571. AGVAVV V5B6P1 V5B6P1_TOXGO Putative galactosyltransferase 441 331-336
572. AVVARG V4ZR42 V4ZR42_TOXGO Uncharacterized protein 1471 1057-1062
573. AVVARG V4ZTF9 V4ZTF9_TOXGO HEAT repeat-containing protein 766 380-385
574. AVVARG V5B518 V5B518_TOXGO Putative COPI protein 1256 362-367
575. VVARGA B9QD38 B9QD38_TOXGO NudC family protein 384 131-136
576. RGAQAL B6KKZ6 B6KKZ6_TOXGO DEAD/DEAH box helicase domain-containing protein 682 85-90
577. AQALLR Q9XY88 Q9XY88_TOXGO Glucose-6-phosphate isomerase 563 347-352
578. AQALLR V4Z3X6 V4Z3X6_TOXGO DNA gyrase/topoisomerase IV, A subunit domain-containing protein 1273 721-726
579. AQALLR V4Z938 V4Z938_TOXGO Glucose-6-phosphate isomerase 661 445-450
580. AQALLR V4ZBH1 V4ZBH1_TOXGO Uncharacterized protein 3736 2725-2730
581. AQALLR V4ZNQ0 V4ZNQ0_TOXGO Uncharacterized protein 5025 1428-1433
582. AQALLR V4ZRJ7 V4ZRJ7_TOXGO Asparagine synthase 1101 279-284
583. AQALLR V5B185 V5B185_TOXGO Chromosome condensation regulator repeat protein 2834 1386-1391
584. QALLRD B9Q8D6 B9Q8D6_TOXGO Uncharacterized protein 1313 373-378
585. GESLHR B9PY04 B9PY04_TOXGO Uncharacterized protein 3263 2215-2220
586. GESLHR V4ZGA2 V4ZGA2_TOXGO Uncharacterized protein 708 558-563
587. SLHRYF V4Z214 V4Z214_TOXGO WD domain, G-beta repeat-containing protein 3345 713-718
588. EDGFLV B6KHQ9 B6KHQ9_TOXGO Tetratricopeptide repeat-containing protein 2068 430-435
589. FLVNPS B9QRA2 B9QRA2_TOXGO GYF domain-containing protein 2331 78-83
590. LVNPSL V4Z402 V4Z402_TOXGO Uncharacterized protein 1883 1654-1659
591. SLTRDR Q1JSD2 Q1JSD2_TOXGO Uncharacterized protein 1409 922-927
592. SLTRDR V4Z6L1 V4Z6L1_TOXGO Protein kinase domain protein 1375 888-893
593. SLTRDR V4ZL97 V4ZL97_TOXGO Uncharacterized protein 7954 519-524
594. QQTLRL V5AYL8 V5AYL8_TOXGO Uncharacterized protein 1236 380-385
595. PLWSRY V5BMD9 V5BMD9_TOXGO UBA/TS-N domain-containing protein 7817 2022-2027
596. RFLQPV V4Z851 V4Z851_TOXGO NAC domain-containing protein 359 108-113
597. QPVDDT V4ZGS5 V4ZGS5_TOXGO Putative cullin 3 930 576-581
598. EPADPI V4ZJW7 V4ZJW7_TOXGO Sma protein 2746 1031-1036
599. SGTCIR B6KAZ3 B6KAZ3_TOXGO Putative proteophosphoglycan 5, related protein 2250 742-747
600. SPPPDA V4ZQY3 V4ZQY3_TOXGO TBC domain-containing kinase (Incomplete catalytic triad) 1887 1085-1090
601. PPPDAP V4ZA17 V4ZA17_TOXGO Uncharacterized protein 2390 2101-2106
602. IDILKR B9PN35 B9PN35_TOXGO Acid phosphatase 1632 1030-1035
603. DILKRL V4ZNK4 V4ZNK4_TOXGO Radical SAM domain-containing protein 1011 410-415
604. ILKRLA B9PYC3 B9PYC3_TOXGO Eukaryotic translation initiation factor 3 subunit C 1104 339-344
605. ILKRLA V4Z1I1 V4Z1I1_TOXGO Eukaryotic translation initiation factor 3 subunit C 971 339-344
606. GKKIDG B9Q072 B9Q072_TOXGO RNA recognition motif-containing protein 274 160-165
607. TVVAVT A3FKJ8 A3FKJ8_TOXGO PMCA-type calcium ATPase A2 1200 895-900
608. TVVAVT B9QIY1 B9QIY1_TOXGO CMGC kinase, CDK family 1310 31-36
609. TVVAVT V5BLZ2 V5BLZ2_TOXGO Calcium-translocating P-type ATPase, PMCA-type protein 1448 1028-1033
610. VAVTVF V4ZBS3 V4ZBS3_TOXGO Patched family protein 1705 1463-1468
611. SLATGK V4YJD5 V4YJD5_TOXGO Uncharacterized protein 2264 2177-2182
612. TGKRPG B9QJL9 B9QJL9_TOXGO Uncharacterized protein 1492 1281-1286
613. GKRPGG B9QMB6 B9QMB6_TOXGO AP2 domain transcription factor AP2IX-8 1753 663-668
614. KSIWLL Q9MTD7 Q9MTD7_TOXGO Clp 765 382-387
615. VDTVSG V4Z4M8 V4Z4M8_TOXGO Amine-terminal region of chorein, A TM vesicle-mediated sorter 13455 4494-4499
616. SGLSDR V4ZR91 V4ZR91_TOXGO Uncharacterized protein 3903 151-156
617. GLSDRK V4ZJD7 V4ZJD7_TOXGO Uncharacterized protein 561 85-90
618. SDRKFQ V5AXQ7 V5AXQ7_TOXGO Putative proton ATPase 2261 734-739
619. GSTEKN B6KHD0 B6KHD0_TOXGO AP2 domain transcription factor AP2XII-6 279 116-121
620. EKNIRS B9Q7F5 B9Q7F5_TOXGO AP2 domain transcription factor AP2X-4 896 671-676
621. PRVEEA V4Z782 V4Z782_TOXGO Sad1/UNC family protein 3923 3911-3916
622. RVEEAL V4Z6T1 V4Z6T1_TOXGO Dopey, N-terminal domain-containing protein 3103 685-690
623. RVEEAL V4ZBX8 V4ZBX8_TOXGO Phosphatidylinositol 3-and 4-kinase 6746 3703-3708
624. RVEEAL V5BMI9 V5BMI9_TOXGO Uncharacterized protein 1612 1204-1209
625. ALTQLK V4ZFK2 V4ZFK2_TOXGO SWI2/SNF2-containing protein RAD16 1700 225-230
626. LTQLKA V4ZFK2 V4ZFK2_TOXGO SWI2/SNF2-containing protein RAD16 1700 226-231
627. LKAGKL V4ZB16 V4ZB16_TOXGO DnaJ domain-containing protein 442 264-269
628. ARKDEG B9Q4Z8 B9Q4Z8_TOXGO Uncharacterized protein 2714 1820-1825
629. ARKDEG V4ZAK3 V4ZAK3_TOXGO Uncharacterized protein 3378 1382-1387
630. SRWKRP V4ZNH6 V4ZNH6_TOXGO Putative aquarius 2250 132-137
631. EMLERL B9Q022 B9Q022_TOXGO SF-assemblin/beta giardin protein 262 89-94
632. EMLERL V4YKM6 V4YKM6_TOXGO Zinc finger, C3HC4 type (RING finger) protein 1815 394-399
633. GLSLLV B9PSG2 B9PSG2_TOXGO Putative proteasome subunit alpha2, protease of the acylase family and NTN hydrolase fold 236 132-137
634. GLSLLV Q6GYB4 Q6GYB4_TOXGO 69N21 531 281-286
635. GLSLLV V4YTX9 V4YTX9_TOXGO Inner membrane complex protein IMC3 695 280-285
636. GLSLLV V4Z4U3 V4Z4U3_TOXGO ABC transporter, ATP-binding domain-containing protein 1885 829-834
637. GLSLLV V4ZEL7 V4ZEL7_TOXGO CPSF A subunit region protein 2847 1041-1046
638. GLSLLV V4ZNB8 V4ZNB8_TOXGO Putative CLEC16A 1806 178-183
639. GLSLLV V5B4G9 V5B4G9_TOXGO Uncharacterized protein 2072 1652-1657
640. LSLLVF B9PUL3 B9PUL3_TOXGO Multi-pass transmembrane protein 305 132-137
641. LSLLVF V4Z500 V4Z500_TOXGO Uncharacterized protein 604 15-20
642. LSLLVF V4Z5G1 V4Z5G1_TOXGO Putative, related protein 1658 1173-1178
643. LSLLVF V4ZEL7 V4ZEL7_TOXGO CPSF A subunit region protein 2847 1042-1047
644. SLLVFA Q6DMN0 Q6DMN0_TOXGO Cathepsin CPL 422 71-76
645. SLLVFA V4ZGC3 V4ZGC3_TOXGO DNA-directed RNA polymerase 2141 1696-1701
646. CSAEAA V4ZF87 V4ZF87_TOXGO AP2 domain transcription factor AP2X-6 4495 1369-1374
647. CSAEAA V4ZJP8 V4ZJP8_TOXGO Amine-terminal region of chorein, A TM vesicle-mediated sorter 12207 10085-10090
648. EAAPPP B9Q7W6 B9Q7W6_TOXGO Putative F-box protein 1461 1031-1036
649. EAAPPP V4YRN2 V4YRN2_TOXGO Uncharacterized protein 720 517-522
650. EAAPPP V4Z357 V4Z357_TOXGO Toxoplasma gondii family A protein 610 255-260
651. EAAPPP V4ZEC4 V4ZEC4_TOXGO Uncharacterized protein 1155 701-706
652. AAPPPA B6K9E1 B6K9E1_TOXGO Ribosomal L1p/L10e family protein 469 363-368
653. AAPPPA B6KGA5 B6KGA5_TOXGO HXXEE motif protein 385 34-39
654. AAPPPA B9QI97 B9QI97_TOXGO Uncharacterized protein 160 21-26
655. AAPPPA V4ZG55 V4ZG55_TOXGO CBS domain-containing protein 924 169-174
656. AAPPPA V4ZPX5 V4ZPX5_TOXGO Surp module domain-containing protein 658 534-539
657. APPPAK B6K9E1 B6K9E1_TOXGO Ribosomal L1p/L10e family protein 469 364-369
658. APPPAK V4YJ26 V4YJ26_TOXGO LSM domain-containing protein 169 89-94
659. PPPAKP V4YJ26 V4YJ26_TOXGO LSM domain-containing protein 169 90-95
660. PAKPPP B6KDM8 B6KDM8_TOXGO O-linked N-acetylglucosamine transferase 978 771-776
661. PAKPPP B9QLX2 B9QLX2_TOXGO Uncharacterized protein 1198 602-607
662. PAKPPP V4Z9U0 V4Z9U0_TOXGO Uncharacterized protein 5083 2901-2906
663. PAKPPP V4ZBN7 V4ZBN7_TOXGO Uncharacterized protein 2456 222-227
664. AKPPPP V4ZBB6 V4ZBB6_TOXGO Uncharacterized protein 3325 2808-2813
665. AKPPPP V4ZPQ9 V4ZPQ9_TOXGO Uncharacterized protein 1528 1068-1073
666. KPPPPP B6KAY9 B6KAY9_TOXGO Uncharacterized protein 1116 996-1001
667. KPPPPP B9Q655 B9Q655_TOXGO RNA recognition motif-containing protein 1374 244-249
668. KPPPPP V4ZA19 V4ZA19_TOXGO Uncharacterized protein 882 603-608
669. KPPPPP V4ZPQ9 V4ZPQ9_TOXGO Uncharacterized protein 1528 1069-1074
670. PPPPPQ B9PLM3 B9PLM3_TOXGO Putative U1 snRNP-associated protein Usp106 394 283-288
671. PPPPPQ B9PY54 B9PY54_TOXGO Putative uncharacterized protein 583 521-526
672. PPPPPQ B9QGZ1 B9QGZ1_TOXGO RAP domain-containing protein 571 373-378
673. PPPPPQ B9QR64 B9QR64_TOXGO RNA recognition motif (A.K.A RRM, RBD, or RNP domain) protein 2072 486-491
674. PPPPPQ V4YLP1 V4YLP1_TOXGO Arsenite-resistance protein 2 811 749-754
675. PPPPPQ V4ZD34 V4ZD34_TOXGO Putative RNA recognition motif (A.K.A RRM, RBD, or RNP domain) protein 826 532-537
676. PPPPPQ V5B7I9 V5B7I9_TOXGO OST-HTH associated domain protein 383 363-368
677. PPPPQP B6KJS1 B6KJS1_TOXGO snRNA-activating of 50 kDa MW carboxy-terminal protein 1179 390-395
678. PPPPQP B9PLM3 B9PLM3_TOXGO Putative U1 snRNP-associated protein Usp106 394 284-289
679. PPPPQP B9QFU9 B9QFU9_TOXGO ATP-dependent metallopeptidase HflB subfamily protein 1188 404-409
680. PPPPQP B9QPL5 B9QPL5_TOXGO Vacuolar sorting 9 (VPS9 domain ) protein 483 398-403
681. PPPPQP V4ZEN1 V4ZEN1_TOXGO Putative transmembrane protein 329 273-278
682. PPPQPL B6KJS1 B6KJS1_TOXGO snRNA-activating of 50 kDa MW carboxy-terminal protein 1179 391-396
683. PPPQPL B9QPH7 B9QPH7_TOXGO Putative transmembrane protein 394 283-288
684. PPPQPL V5B720 V5B720_TOXGO KH domain protein 1275 208-213
685. PPQPLP B6KJS1 B6KJS1_TOXGO snRNA-activating of 50 kDa MW carboxy-terminal protein 1179 392-397
686. PPQPLP B9Q8I2 B9Q8I2_TOXGO Uncharacterized protein 2497 429-434
687. PPQPLP V4Z889 V4Z889_TOXGO CRAL/TRIO domain-containing protein 994 26-31
688. QPLPSP V4ZJC9 V4ZJC9_TOXGO Uncharacterized protein 939 40-45
689. QPLPSP V5B8H8 V5B8H8_TOXGO Proteasome/cyclosome repeat-containing protein 167 9-14
690. PLPSPA B9Q792 B9Q792_TOXGO Uncharacterized protein 4118 2088-2093
691. PLPSPA Q1JT10 Q1JT10_TOXGO Phosphodiesterase, putative 2092 634-639
692. PLPSPA V4YYX8 V4YYX8_TOXGO EF-hand protein 1299 594-599
693. PLPSPA V4Z1J2 V4Z1J2_TOXGO 3'5'-cyclic nucleotide phosphodiesterase domain-containing protein 2238 634-639
694. PLPSPA V4Z1T8 V4Z1T8_TOXGO Uncharacterized protein 4885 3562-3567
695. PLPSPA V4Z6X7 V4Z6X7_TOXGO Exportin 1-like protein 1511 812-817
696. PLPSPA V4ZQG9 V4ZQG9_TOXGO Uncharacterized protein 3806 1039-1044
697. PLPSPA V5BJM2 V5BJM2_TOXGO Putative DNA ligase (NAD+) 1975 422-427
698. LPSPAY B9QF02 B9QF02_TOXGO Uncharacterized protein 1847 1565-1570
699. LPSPAY V5BKW8 V5BKW8_TOXGO Uncharacterized protein 713 6-11
700. PSPAYP B9QF02 B9QF02_TOXGO Uncharacterized protein 1847 1566-1571
701. PSPAYP V5BKW8 V5BKW8_TOXGO Uncharacterized protein 713 7-12
702. SPAYPA V4ZS52 V4ZS52_TOXGO Sulfite exporter TauE/SafE protein 1632 1029-1034
703. SPAYPA V5BKW8 V5BKW8_TOXGO Uncharacterized protein 713 8-13
704. PAPRPA V4Z772 V4Z772_TOXGO AP2 domain transcription factor AP2X-11 1372 457-462
705. PAPRPA V4ZCG9 V4ZCG9_TOXGO Diacylglycerol kinase 679 147-152
706. PAPRPA V4ZDA4 V4ZDA4_TOXGO Uncharacterized protein 1681 703-708
707. PAPRPA V4ZHM6 V4ZHM6_TOXGO Uncharacterized protein 2986 1250-1255
708. APRPAP V4ZJB1 V4ZJB1_TOXGO Uncharacterized protein 337 19-24
709. APRPAP V4ZRI4 V4ZRI4_TOXGO Uncharacterized protein 141 29-34
710. RPAPGP B6KAE3 B6KAE3_TOXGO Emp24/gp25L/p24 family protein 601 160-165
711. RPAPGP V4YK13 V4YK13_TOXGO Uncharacterized protein 1986 1403-1408
712. RPAPGP V4Z4T1 V4Z4T1_TOXGO Uncharacterized protein 1799 677-682
713. PAPGPA B9QAT4 B9QAT4_TOXGO Kelch repeat and K+ channel tetramerisation domain containing protein 818 170-175
714. PAPGPA B9QN68 B9QN68_TOXGO AP2 domain transcription factor AP2X-8 3817 3534-3539
715. PAPGPA V4YYU7 V4YYU7_TOXGO Putative glyoxalase 4813 428-433
716. PAPGPA V5B421 V5B421_TOXGO Uncharacterized protein 1878 1065-1070
717. APGPAP B9Q560 B9Q560_TOXGO Transport protein Trs120 2958 73-78
718. APGPAP V4Z4M8 V4Z4M8_TOXGO Amine-terminal region of chorein, A TM vesicle-mediated sorter 13455 5085-5090
719. APGPAP V4ZHE1 V4ZHE1_TOXGO ATG C terminal domain-containing protein 8079 4797-4802
720. FVPRER B6K8Y9 B6K8Y9_TOXGO DEAD (Asp-Glu-Ala-Asp) box polypeptide DDX6 475 271-276
721. RERASV B9QJE8 B9QJE8_TOXGO Uncharacterized protein 3150 2617-2622
722. RERASV V4YTX2 V4YTX2_TOXGO Uncharacterized protein 2251 188-193
723. RERASV V4YYG6 V4YYG6_TOXGO Putative zinc finger protein 2469 2388-2393
724. RERASV V4ZV44 V4ZV44_TOXGO Putative transmembrane protein 1476 1250-1255
725. RASVDR B9QH07 B9QH07_TOXGO Uncharacterized protein 412 6-11
726. RASVDR V4ZMY4 V4ZMY4_TOXGO Uncharacterized protein 1350 409-414
727. RASVDR V5BKN7 V5BKN7_TOXGO Putative GCN1 3416 1264-1269
728. ASVDRW Q1JSU4 Q1JSU4_TOXGO Uncharacterized protein 766 203-208
729. ASVDRW V4ZAA6 V4ZAA6_TOXGO Putative transmembrane protein 726 203-208
730. RRTKGA B9QE30 B9QE30_TOXGO AP2 domain transcription factor AP2IX-5 2282 1704-1709
731. KGAGPP V5BEI1 V5BEI1_TOXGO Putative 50S ribosomal protein L21 662 405-410
732. GAGPPG B6KGY9 B6KGY9_TOXGO Uncharacterized protein 2303 1605-1610
733. GAGPPG V4YS99 V4YS99_TOXGO Putative Myb-like DNA-binding domain protein 1147 783-788
734. GAGPPG V4ZMJ1 V4ZMJ1_TOXGO Uncharacterized protein 4969 3350-3355
735. AGPPGG V4ZBR9 V4ZBR9_TOXGO Protein kinase 1763 818-823
736. AGPPGG V4ZJI3 V4ZJI3_TOXGO SPOC domain-containing protein 704 404-409
737. AGPPGG V4ZMJ1 V4ZMJ1_TOXGO Uncharacterized protein 4969 3351-3356
738. GPPGGA B9PYE9 B9PYE9_TOXGO Uncharacterized protein 2457 2442-2447
739. GPPGGA Q1JTE4 Q1JTE4_TOXGO Putative uncharacterized protein 2465 2450-2455
740. GPPGGA V4Z6Q7 V4Z6Q7_TOXGO AP2 domain transcription factor AP2VIII-2 2503 2343-2348
741. GPPGGA V5BAB1 V5BAB1_TOXGO RNA recognition motif-containing protein 539 297-302
742. PPGGAG B6KHG4 B6KHG4_TOXGO Uncharacterized protein 1652 524-529
743. PPGGAG B9PYE9 B9PYE9_TOXGO Uncharacterized protein 2457 2443-2448
744. PPGGAG B9QMH6 B9QMH6_TOXGO RNA pseudouridine synthase superfamily protein 6535 8-13
745. PPGGAG B9QRA2 B9QRA2_TOXGO GYF domain-containing protein 2331 521-526
746. PPGGAG Q1JTE4 Q1JTE4_TOXGO Putative uncharacterized protein 2465 2451-2456
747. PPGGAG V4ZGI8 V4ZGI8_TOXGO Uncharacterized protein 474 221-226
748. PPGGAG V5BAD4 V5BAD4_TOXGO STE kinase 3951 3501-3506
749. PGGAGL B9QMH6 B9QMH6_TOXGO RNA pseudouridine synthase superfamily protein 6535 9-14
750. PGGAGL B9QN68 B9QN68_TOXGO AP2 domain transcription factor AP2X-8 3817 1570-1575
751. PGGAGL V4Z414 V4Z414_TOXGO Uncharacterized protein 662 70-75
752. PGGAGL V4Z839 V4Z839_TOXGO Uncharacterized protein 1953 664-669
753. GGAGLA B6KAM8 B6KAM8_TOXGO Tetratricopeptide repeat-containing protein 1697 657-662
754. GGAGLA B9QN68 B9QN68_TOXGO AP2 domain transcription factor AP2X-8 3817 1571-1576
755. GGAGLA V4ZPQ9 V4ZPQ9_TOXGO Uncharacterized protein 1528 1078-1083
756. PQGLGL B9QI26 B9QI26_TOXGO Pumilio-family RNA binding repeat-containing protein 1913 471-476
757. QGLGLG B9QI26 B9QI26_TOXGO Pumilio-family RNA binding repeat-containing protein 1913 472-477
758. GLGLGL V4ZP14 V4ZP14_TOXGO RIC1 protein 3789 1118-1123
759. GLGLGE B9QI26 B9QI26_TOXGO Pumilio-family RNA binding repeat-containing protein 1913 473-478
760. GLGLGE V4YZ64 V4YZ64_TOXGO Putative transmembrane protein 1698 1430-1435
761. GLGLGE V5B872 V5B872_TOXGO Myb family DNA-binding domain-containing protein 1755 1204-1209
762. LGLGEA V4YSA9 V4YSA9_TOXGO Putative alpha-glucan water dikinase 1 163 30-35
763. GLGEAR V4ZDE5 V4ZDE5_TOXGO Histidine acid phosphatase superfamily protein 2930 2150-2155
764. LGEARA V4ZDU9 V4ZDU9_TOXGO Putative transmembrane protein 3661 69-74
765. LGEARA V4ZSU4 V4ZSU4_TOXGO Putative transmembrane protein 1274 1144-1149
766. GEARAA V4Z893 V4Z893_TOXGO Uncharacterized protein 740 734-739
767. GEARAA V4ZC41 V4ZC41_TOXGO Uncharacterized protein 496 309-314
768. GEARAA V4ZEF3 V4ZEF3_TOXGO Putative transmembrane protein 1605 823-828
769. GEARAA V4ZML4 V4ZML4_TOXGO R3H domain-containing protein 1307 1081-1086
770. GEARAA V4ZPH3 V4ZPH3_TOXGO Protein kinase domain protein 2329 1201-1206
771. GEARAA V4ZX05 V4ZX05_TOXGO RNA recognition motif-containing protein 2089 88-93
772. GEARAA V5B1A6 V5B1A6_TOXGO Uncharacterized protein 1824 411-416
773. EARAAP B9PHY3 B9PHY3_TOXGO WW domain binding protein 11 561 438-443
774. EARAAP V4Z893 V4Z893_TOXGO Uncharacterized protein 740 735-740
775. EARAAP V5B1R8 V5B1R8_TOXGO Putative adenylate kinase 2334 1039-1044
776. ARAAPR V4YNA1 V4YNA1_TOXGO Uncharacterized protein 352 31-36
777. ARAAPR V4Z5G1 V4Z5G1_TOXGO Putative, related protein 1658 1309-1314
778. ARAAPR V4ZRP3 V4ZRP3_TOXGO RecF/RecN/SMC N terminal domain-containing protein 2097 1484-1489
779. RAAPRG V4YNA1 V4YNA1_TOXGO Uncharacterized protein 352 32-37
780. RAAPRG V4Z2A1 V4Z2A1_TOXGO Parkinson disease 7 domain containing 1 family protein 765 349-354
781. RAAPRG V4ZBT9 V4ZBT9_TOXGO Uncharacterized protein 260 183-188
782. AAPRGA B6KP41 B6KP41_TOXGO Putative pinA 589 293-298
783. AAPRGA Q1JSS3 Q1JSS3_TOXGO Uncharacterized protein 589 293-298
784. AAPRGA V4ZE60 V4ZE60_TOXGO Internal kinesin motor domain protein 1360 1008-1013
785. AAPRGA V5BG16 V5BG16_TOXGO Uncharacterized protein 1767 670-675
786. APRGAA Q1JTL1 Q1JTL1_TOXGO Putative uncharacterized protein 1525 973-978
787. APRGAA V4YNM0 V4YNM0_TOXGO Uncharacterized protein 2518 1999-2004
788. APRGAA V4ZP24 V4ZP24_TOXGO Putative DNA mismatch repair protein MSH6-1 1567 111-116
789. APRGAA V4ZQG9 V4ZQG9_TOXGO Uncharacterized protein 3806 3330-3335
790. APRGAA V5AZJ8 V5AZJ8_TOXGO Vps54 family protein 1983 1431-1436
791. PRGAAG V4YN85 V4YN85_TOXGO Uncharacterized protein 1434 791-796
792. PRGAAG V4ZKG4 V4ZKG4_TOXGO Zinc finger (CCCH type) motif-containing protein 816 801-806
793. PRGAAG V4ZP24 V4ZP24_TOXGO Putative DNA mismatch repair protein MSH6-1 1567 112-117
794. RGAAGR V4YV31 V4YV31_TOXGO Putative Bardet-Biedl syndrome 5 716 378-383
795. RGAAGR V4Z718 V4Z718_TOXGO Concanavalin A-like lectin/glucanase family protein 1494 918-923
796. RGAAGR V4ZJT3 V4ZJT3_TOXGO Uncharacterized protein 1521 559-564
797. GAAGRP B9Q6B6 B9Q6B6_TOXGO Uncharacterized protein 1988 1575-1580
798. GAAGRP V4Z9H4 V4Z9H4_TOXGO Uncharacterized protein 2348 1373-1378
799. AAGRPL V5B1Q8 V5B1Q8_TOXGO Putative transmembrane protein 913 352-357
800. AGRPLS B9Q6C0 B9Q6C0_TOXGO Filamin/ABP280 repeat-containing protein 2728 3-8
801. AGRPLS V4ZHU3 V4ZHU3_TOXGO Transporter, cation channel family protein 1356 8-13
802. AGRPLS V5B154 V5B154_TOXGO Putative SAC3/GANP family protein 2520 13-18
803. AGRPLS V5B7V9 V5B7V9_TOXGO L1P family of ribosomal protein 928 249-254
804. GRPLSP B9QL14 B9QL14_TOXGO Inositol polyphosphate kinase 2851 1370-1375
805. GRPLSP V4Z4W9 V4Z4W9_TOXGO Uncharacterized protein 2995 2194-2199
806. RPLSPP B6KGF5 B6KGF5_TOXGO Uncharacterized protein 1182 426-431
807. RPLSPP B9QGI1 B9QGI1_TOXGO YGGT family protein 466 205-210
808. RPLSPP Q1JT37 Q1JT37_TOXGO Putative uncharacterized protein 719 12-17
809. RPLSPP V5AZW2 V5AZW2_TOXGO Histone lysine acetyltransferase HAT1 741 12-17
810. RPLSPP V5B5Z2 V5B5Z2_TOXGO Hydrolase, NUDIX family protein 220 23-28
811. RPLSPP V5BDD5 V5BDD5_TOXGO CPSF A subunit region protein 2077 1238-1243
812. PLSPPA V4Z8R9 V4Z8R9_TOXGO Uncharacterized protein 627 394-399
813. PLSPPA V4Z8V0 V4Z8V0_TOXGO AP2 domain transcription factor AP2VI-3 1246 400-405
814. PLSPPA V4ZD39 V4ZD39_TOXGO Uncharacterized protein 3436 2993-2998
815. LSPPAA V4Z4F4 V4Z4F4_TOXGO Uncharacterized protein 3102 2465-2470
816. LSPPAA V4ZG82 V4ZG82_TOXGO AP2 domain transcription factor AP2XII-3 659 348-353
817. LSPPAA V4ZNI0 V4ZNI0_TOXGO Putative transmembrane protein 6079 344-349
818. PPAAQP B9PUT7 B9PUT7_TOXGO WD domain, G-beta repeat-containing protein 654 603-608
819. PPAAQP B9QEX1 B9QEX1_TOXGO SAG-related sequence SRS44 2347 2012-2017
820. PPAAQP V4ZA19 V4ZA19_TOXGO Uncharacterized protein 882 811-816
821. PPAAQP V5B1K2 V5B1K2_TOXGO Uncharacterized protein 1673 1620-1625
822. PAAQPP V5B9F1 V5B9F1_TOXGO Chromodomain helicase DNA binding protein CHD1/SWI2/SNF2 2492 180-185
823. IVRDKE B9QFF4 B9QFF4_TOXGO Putative transmembrane protein 744 163-168
824. VRDKEP B9QFF4 B9QFF4_TOXGO Putative transmembrane protein 744 164-169
825. VRDKEP B9QH70 B9QH70_TOXGO Putative proteophosphoglycan ppg4, related protein 1992 327-332
826. PAEPPA B6KP28 B6KP28_TOXGO PIG-P protein 407 144-149
827. PAEPPA Q1JST5 Q1JST5_TOXGO Uncharacterized protein 554 266-271
828. PAEPPA V4ZFB6 V4ZFB6_TOXGO Patched family protein 1178 103-108
829. PAEPPA V4ZL65 V4ZL65_TOXGO Uncharacterized protein 1082 728-733
830. AEPPAG V4ZM84 V4ZM84_TOXGO Uncharacterized protein 2165 115-120
831. EPPAGA V5BE64 V5BE64_TOXGO Uncharacterized protein 658 399-404
832. PPAGAF V4ZAP5 V4ZAP5_TOXGO Zinc finger, C3HC4 type (RING finger) domain-containing protein 3893 1511-1516
833. PPAGAF V4ZD45 V4ZD45_TOXGO Tryptophanyl-tRNA synthetase (TrpRS1) 835 205-210
834. PPAGAF V4ZJE9 V4ZJE9_TOXGO Enhancer of polycomb-like protein 1474 1401-1406
835. AGAFPG V4Z0J5 V4Z0J5_TOXGO Putative nuclear NF-kB activator 1053 503-508
836. AGAFPG V4Z4B4 V4Z4B4_TOXGO Sec7 domain-containing protein 3015 809-814
837. AGAFPG V4Z4R2 V4Z4R2_TOXGO AP2 domain transcription factor AP2IX-9 1338 1033-1038
838. AGAFPG V5BF78 V5BF78_TOXGO SWI2/SNF2-containing protein 3110 2584-2589
839. GAFPGF V4ZLR9 V4ZLR9_TOXGO GYF domain-containing protein 1821 1573-1578
840. AFPGFP B6KFT5 B6KFT5_TOXGO Toxoplasma gondii family A protein 431 108-113
841. PGFPSP B6KU24 B6KU24_TOXGO Uncharacterized protein 1177 82-87
842. PGFPSP B9Q341 B9Q341_TOXGO Dynamin-related protein DRPB 860 445-450
843. PGFPSP Q1JSC9 Q1JSC9_TOXGO DyNamin-like protein, putative 835 420-425
844. GFPSPP V4ZGU8 V4ZGU8_TOXGO Zinc finger in N-recognin protein 4383 173-178
845. FPSPPA B9Q6D3 B9Q6D3_TOXGO DnaJ domain-containing protein 520 469-474
846. FPSPPA Q1JST6 Q1JST6_TOXGO Uncharacterized protein 1030 833-838
847. FPSPPA V4Z1S4 V4Z1S4_TOXGO HEAT repeat-containing protein 1450 1244-1249
848. FPSPPA V5BF78 V5BF78_TOXGO SWI2/SNF2-containing protein 3110 331-336
849. PSPPAP B6KRY9 B6KRY9_TOXGO Putative transmembrane protein 1246 538-543
850. PSPPAP B9Q7Y5 B9Q7Y5_TOXGO PolyA polymerase 778 30-35
851. PSPPAP K7X7G8 K7X7G8_TOXGO Palmitoyltransferase 510 109-114
852. PSPPAP V4YRC2 V4YRC2_TOXGO Putative transmembrane protein 4703 13-18
853. PSPPAP V4Z0N1 V4Z0N1_TOXGO Cyclin dependent kinase binding protein 912 89-94
854. PSPPAP V4Z3J3 V4Z3J3_TOXGO Uncharacterized protein 1357 13-18
855. PSPPAP V4Z843 V4Z843_TOXGO Uncharacterized protein 1132 263-268, 268-273, 273-278, 278-283
856. PSPPAP V4ZGV1 V4ZGV1_TOXGO Palmitoyltransferase 466 64-69
857. SPPAPP K7X7G8 K7X7G8_TOXGO Palmitoyltransferase 510 110-115
858. SPPAPP Q1JSP6 Q1JSP6_TOXGO Vacuolar ATP synthase subunit h, putative 425 14-19
859. SPPAPP Q1JTI3 Q1JTI3_TOXGO Ubiquitin-protein ligase 1, putative 8112 3727-3732
860. SPPAPP V4Z4W9 V4Z4W9_TOXGO Uncharacterized protein 2995 308-313
861. SPPAPP V4Z553 V4Z553_TOXGO HECT-domain (Ubiquitin-transferase) domain-containing protein 8007 3727-3732
862. SPPAPP V4ZGB0 V4ZGB0_TOXGO Zinc finger (CCCH type) motif-containing protein 1513 249-254
863. SPPAPP V4ZGV1 V4ZGV1_TOXGO Palmitoyltransferase 466 65-70
864. SPPAPP V4ZIA7 V4ZIA7_TOXGO Uncharacterized protein 814 672-677
865. PPAPPA B6KBE4 B6KBE4_TOXGO 2OG-Fe(II) oxygenase family protein 927 776-781
866. PPAPPA B9PJA8 B9PJA8_TOXGO Reactive oxygen species modulator 1 263 68-73
867. PPAPPA B9QQX0 B9QQX0_TOXGO Putative transmembrane protein 342 194-199
868. PPAPPA Q1JSL2 Q1JSL2_TOXGO Uncharacterized protein 107 101-106
869. PPAPPA V4Z6A8 V4Z6A8_TOXGO Cwfj family protein 821 433-438
870. PPAPPA V4Z843 V4Z843_TOXGO Uncharacterized protein 1132 321-326
871. PPAPPA V4ZAJ7 V4ZAJ7_TOXGO Family UPF0139 protein 113 107-112
872. PPAPPA V4ZAL3 V4ZAL3_TOXGO Cation-transporting atpase family protein 1260 389-394
873. PPAPPA V4ZJP8 V4ZJP8_TOXGO Amine-terminal region of chorein, A TM vesicle-mediated sorter 12207 11205-11210
874. PAPPAA B6KBE4 B6KBE4_TOXGO 2OG-Fe(II) oxygenase family protein 927 777-782
875. PAPPAA B9QAT0 B9QAT0_TOXGO TLD protein 1392 854-859
876. PAPPAA B9QP51 B9QP51_TOXGO Uncharacterized protein 1652 1336-1341
877. PAPPAA B9QPW9 B9QPW9_TOXGO Uncharacterized protein 680 306-311
878. PAPPAA Q1JSL2 Q1JSL2_TOXGO Uncharacterized protein 107 102-107
879. PAPPAA V4Z214 V4Z214_TOXGO WD domain, G-beta repeat-containing protein 3345 722-727
880. PAPPAA V4Z7K0 V4Z7K0_TOXGO Uncharacterized protein 1512 215-220
881. PAPPAA V4Z8M7 V4Z8M7_TOXGO Putative transmembrane protein 1037 608-613
882. PAPPAA V4ZAJ7 V4ZAJ7_TOXGO Family UPF0139 protein 113 108-113
883. PAPPAA V5B5U5 V5B5U5_TOXGO Putative alpha-glucan water dikinase 1 1552 287-292
884. APPAAA B6KGE9 B6KGE9_TOXGO Uncharacterized protein 2533 1019-1024
885. APPAAA B9QAT0 B9QAT0_TOXGO TLD protein 1392 855-860
886. APPAAA B9QPT3 B9QPT3_TOXGO Uncharacterized protein 1541 190-195
887. APPAAA B9QPV6 B9QPV6_TOXGO Organic solute transporter ostalpha protein 1734 236-241
888. APPAAA V4YJS1 V4YJS1_TOXGO Phosphatidylinositol-4-phosphate 5-Kinase 4165 3792-3797
889. APPAAA V4Z0Z2 V4Z0Z2_TOXGO Uncharacterized protein 1456 1263-1268
890. APPAAA V4Z214 V4Z214_TOXGO WD domain, G-beta repeat-containing protein 3345 723-728
891. APPAAA V4Z772 V4Z772_TOXGO AP2 domain transcription factor AP2X-11 1372 427-432
892. APPAAA V4ZAK3 V4ZAK3_TOXGO Uncharacterized protein 3378 1089-1094
893. PPAAAA B6E3J1 B6E3J1_TOXGO ATP-binding cassette sub-family C member 1 1883 588-593
894. PPAAAA B6E3J4 B6E3J4_TOXGO ATP-binding cassette sub-family C member 1 1883 588-593
895. PPAAAA B9Q7H1 B9Q7H1_TOXGO AP2 domain transcription factor AP2X-3 1919 1235-1240
896. PPAAAA D0EP42 D0EP42_TOXGO ATP-binding cassette protein subfamily C member 1 1883 588-593
897. PPAAAA Q1JT45 Q1JT45_TOXGO DNA ligase IV, putative 1023 95-100
898. PPAAAA Q45W20 Q45W20_TOXGO ABC transporter transmembrane region domain-containing protein 1883 588-593
899. PPAAAA V4YQA3 V4YQA3_TOXGO Serine esterase (DUF676) protein 1565 1056-1061
900. PPAAAA V4YXT8 V4YXT8_TOXGO Leucine rich repeat protein 1466 239-244
901. PPAAAA V4Z188 V4Z188_TOXGO ATP-dependent DNA ligase domain-containing protein 1385 95-100
902. PPAAAA V4Z1L7 V4Z1L7_TOXGO Putative transmembrane protein 1241 136-141
903. PPAAAA V4Z2A2 V4Z2A2_TOXGO IgA-specific serine endopeptidase 348 91-96
904. PPAAAA V4Z6M5 V4Z6M5_TOXGO IgA-specific serine endopeptidase 336 238-243
905. PPAAAA V4Z9X6 V4Z9X6_TOXGO Pumilio-family RNA binding repeat-containing protein 1676 924-929
906. PPAAAA V4ZAK3 V4ZAK3_TOXGO Uncharacterized protein 3378 1090-1095
907. PPAAAA V4ZJP8 V4ZJP8_TOXGO Amine-terminal region of chorein, A TM vesicle-mediated sorter 12207 11769-11774
908. PPAAAA V4ZN38 V4ZN38_TOXGO Putative arabinogalactan protein 328 180-185
909. PPAAAA V4ZNW9 V4ZNW9_TOXGO SAG-related sequence SRS53B 395 173-178
910. PPAAAA V4ZTH9 V4ZTH9_TOXGO Putative transmembrane protein 1278 1012-1017
911. PAAAAT B9QAR2 B9QAR2_TOXGO Uncharacterized protein 283 139-144
912. PAAAAT B9QDZ9 B9QDZ9_TOXGO Methionyl-tRNA synthetase 976 236-241
913. PAAAAT B9QJE8 B9QJE8_TOXGO Uncharacterized protein 3150 612-617
914. PAAAAT D0V3Y0 D0V3Y0_TOXGO Formin 1 5051 1086-1091
915. PAAAAT V4YN76 V4YN76_TOXGO Cpw-wpc domain-containing protein 592 143-148
916. PAAAAT V4YY56 V4YY56_TOXGO Putative transmembrane protein 225 121-126
917. PAAAAT V4YZ27 V4YZ27_TOXGO Formin FRM1 5048 1044-1049
918. PAAAAT V4Z782 V4Z782_TOXGO Sad1/UNC family protein 3923 2896-2901
919. PAAAAT V4ZPK3 V4ZPK3_TOXGO Uncharacterized protein 1917 89-94
920. PAAAAT V5B196 V5B196_TOXGO Uncharacterized protein 2539 511-516
921. PAAAAT V5B252 V5B252_TOXGO Uncharacterized protein 1191 719-724
922. AAAATA A8CBF6 A8CBF6_TOXGO Delta-aminolevulinic acid synthetase 584 427-432
923. AAAATA B6KA30 B6KA30_TOXGO AP2 domain transcription factor AP2XI-1 1060 622-627
924. AAAATA B6KH17 B6KH17_TOXGO AP2 domain transcription factor AP2XII-5 1502 362-367
925. AAAATA B6KNZ3 B6KNZ3_TOXGO Pyridine nucleotide-disulfide oxidoreductase domain-containing protein 664 243-248
926. AAAATA B6KR29 B6KR29_TOXGO Uncharacterized protein 288 187-192
927. AAAATA B6KVC0 B6KVC0_TOXGO Uncharacterized protein 1208 520-525
928. AAAATA B9PUI6 B9PUI6_TOXGO RNA recognition motif-containing protein 532 197-202
929. AAAATA B9PY79 B9PY79_TOXGO Sporozoite protein with an altered thrombospondin repeat SPATR 534 149-154
930. AAAATA B9QAR2 B9QAR2_TOXGO Uncharacterized protein 283 140-145
931. AAAATA B9QB70 B9QB70_TOXGO Helicase associated domain (Ha2) protein 1277 88-93
932. AAAATA B9QFM0 B9QFM0_TOXGO Uncharacterized protein 2080 660-665
933. AAAATA B9QFY8 B9QFY8_TOXGO Histone deacetylase HDAC5 1452 769-774
934. AAAATA B9QMH6 B9QMH6_TOXGO RNA pseudouridine synthase superfamily protein 6535 2349-2354
935. AAAATA Q1JSY0 Q1JSY0_TOXGO Ferrodoxin reductase-like protein precursor 612 191-196
936. AAAATA Q1JT84 Q1JT84_TOXGO Putative uncharacterized protein precursor 573 149-154
937. AAAATA V4YQ06 V4YQ06_TOXGO Glucosidase II beta subunit-like protein 964 102-107
938. AAAATA V4YXF7 V4YXF7_TOXGO Calcium dependent protein kinase CDPK7 2133 1114-1119
939. AAAATA V4YYG2 V4YYG2_TOXGO Putative transmembrane protein 2635 436-441
940. AAAATA V4YZX5 V4YZX5_TOXGO Acetyl-coA carboxylase ACC2 3400 1423-1428, 1483-1488
941. AAAATA V4Z0D1 V4Z0D1_TOXGO Putative FIKK kinase 2887 1795-1800, 2029-2034
942. AAAATA V4Z6I1 V4Z6I1_TOXGO GAF domain protein 1026 472-477
943. AAAATA V4Z6T1 V4Z6T1_TOXGO Dopey, N-terminal domain-containing protein 3103 2668-2673
944. AAAATA V4Z782 V4Z782_TOXGO Sad1/UNC family protein 3923 2897-2902
945. AAAATA V4Z7A9 V4Z7A9_TOXGO Putative transmembrane protein 777 546-551
946. AAAATA V4Z7C7 V4Z7C7_TOXGO Polynucleotide adenylyltransferase 835 209-214
947. AAAATA V4Z819 V4Z819_TOXGO DEAD/DEAH box helicase domain-containing protein 1850 1651-1656
948. AAAATA V4Z9Z4 V4Z9Z4_TOXGO Uncharacterized protein 1105 583-588
949. AAAATA V4ZAF6 V4ZAF6_TOXGO Uncharacterized protein 1660 635-640
950. AAAATA V4ZBJ0 V4ZBJ0_TOXGO Uncharacterized protein 3206 1127-1132
951. AAAATA V4ZBM6 V4ZBM6_TOXGO Uncharacterized protein 1503 872-877
952. AAAATA V4ZIT3 V4ZIT3_TOXGO 5-aminolevulinic acid synthase domain-containing protein 752 490-495
953. AAAATA V4ZJI9 V4ZJI9_TOXGO DnaJ domain-containing protein 3043 2877-2882
954. AAAATA V4ZMG2 V4ZMG2_TOXGO AP2 domain transcription factor AP2XI-3 1399 646-651
955. AAAATA V4ZNP4 V4ZNP4_TOXGO Putative GRIP domain protein 921 63-68
956. AAAATA V4ZPK3 V4ZPK3_TOXGO Uncharacterized protein 1917 527-532
957. AAAATA V4ZQG6 V4ZQG6_TOXGO Putative transmembrane protein 988 459-464
958. AAAATA V4ZRN6 V4ZRN6_TOXGO Uncharacterized protein 481 58-63
959. AAAATA V4ZV14 V4ZV14_TOXGO Uncharacterized protein 982 783-788
960. AAAATA V5B196 V5B196_TOXGO Uncharacterized protein 2539 512-517
961. AAAATA V5B672 V5B672_TOXGO Uncharacterized protein 810 537-542
962. AAAATA V5B8E3 V5B8E3_TOXGO Tetratricopeptide repeat-containing protein 1548 1453-1458
963. AAAATA V5BBS6 V5BBS6_TOXGO Ribosome biogenesis GTPase Der protein 1125 696-701
964. AAAATA V5BEQ5 V5BEQ5_TOXGO Putative sortilin 1033 793-798
965. AAAATA V5BHA5 V5BHA5_TOXGO Putative transmembrane protein 963 658-663
966. AAATAV B9QMH6 B9QMH6_TOXGO RNA pseudouridine synthase superfamily protein 6535 2350-2355
967. AAATAV Q1JSB3 Q1JSB3_TOXGO Uncharacterized protein 4189 1368-1373
968. AAATAV V4Z015 V4Z015_TOXGO Cyclin-dependent kinases regulatory subunit 588 475-480
969. AAATAV V4Z6J3 V4Z6J3_TOXGO Myb family DNA-binding domain-containing protein 4221 1368-1373
970. AAATAV V4Z7W4 V4Z7W4_TOXGO Uncharacterized protein 556 243-248
971. AAATAV V4Z819 V4Z819_TOXGO DEAD/DEAH box helicase domain-containing protein 1850 416-421
972. AAATAV V4ZBM6 V4ZBM6_TOXGO Uncharacterized protein 1503 873-878
973. AAATAV V4ZCD5 V4ZCD5_TOXGO FHA domain-containing protein 1042 609-614
974. AAATAV V4ZMA9 V4ZMA9_TOXGO Putative transmembrane protein 244 194-199
975. AAATAV V4ZNB4 V4ZNB4_TOXGO Putative transmembrane protein 696 207-212
976. AAATAV V5B8E3 V5B8E3_TOXGO Tetratricopeptide repeat-containing protein 1548 1454-1459
977. AATAVG B9QER9 B9QER9_TOXGO RNA recognition motif-containing protein 1564 887-892
978. AATAVG V4YNU1 V4YNU1_TOXGO Putative transmembrane protein 3158 1505-1510
979. AATAVG V4ZIR0 V4ZIR0_TOXGO Ubiquitin carboxyl-terminal hydrolase 2294 1798-1803
980. AATAVG V4ZSE4 V4ZSE4_TOXGO WD domain, G-beta repeat-containing protein 4664 925-930
981. AATAVG V5B7K5 V5B7K5_TOXGO Putative 2-oxoglutarate dehydrogenase e1 component, mitochondrial 1116 1074-1079
982. AVGPPL B9QGA4 B9QGA4_TOXGO Uncharacterized protein 1572 995-1000
983. AVGPPL V5BKJ8 V5BKJ8_TOXGO Suppressor of forked protein SUF 1295 1197-1202
984. PPLCRL V4ZEF2 V4ZEF2_TOXGO AP2 domain transcription factor AP2IX-3 3096 1778-1783
985. FEDESP V4Z9L0 V4Z9L0_TOXGO Non-specific serine/threonine protein kinase 1299 985-990
986. EDESPP V4Z3A3 V4Z3A3_TOXGO Putative transmembrane protein 388 54-59
987. EDESPP V4Z9L0 V4Z9L0_TOXGO Non-specific serine/threonine protein kinase 1299 986-991
988. ESPPAP K7WFS9 K7WFS9_TOXGO DHHC11 944 669-674
989. ESPPAP V4YY91 V4YY91_TOXGO Uncharacterized protein 2624 2461-2466
990. ESPPAP V4Z994 V4Z994_TOXGO DHHC zinc finger domain-containing protein 951 676-681
991. ESPPAP V4ZL97 V4ZL97_TOXGO Uncharacterized protein 7954 5879-5884
992. SPPAPA B6KFI1 B6KFI1_TOXGO MoaC family protein 384 287-292
993. SPPAPA B6KRY9 B6KRY9_TOXGO Putative transmembrane protein 1246 539-544
994. SPPAPA V4Z3J3 V4Z3J3_TOXGO Uncharacterized protein 1357 14-19
995. SPPAPA V4Z4G0 V4Z4G0_TOXGO GCC2 and GCC3 domain-containing protein 3768 967-972
996. SPPAPA V4Z8G8 V4Z8G8_TOXGO Met-10+ like-protein 641 43-48
997. SPPAPA V4ZAT6 V4ZAT6_TOXGO DEAD/DEAH box helicase domain-containing protein 1773 1430-1435
998. SPPAPA V4ZF62 V4ZF62_TOXGO Uncharacterized protein 1578 548-553
999. SPPAPA V4ZGQ7 V4ZGQ7_TOXGO Putative activating signal cointegrator 1 complex subunit 3 2304 912-917
1000. SPPAPA V4ZL97 V4ZL97_TOXGO Uncharacterized protein 7954 5880-5885
1001. SPPAPA V5AYI0 V5AYI0_TOXGO NEK kinase 2906 607-612
1002. PPAPAR B9QLS7 B9QLS7_TOXGO WD domain, G-beta repeat-containing protein 2649 2093-2098
1003. PPAPAR V4YID7 V4YID7_TOXGO Uncharacterized protein 383 255-260
1004. PPAPAR V5BFT9 V5BFT9_TOXGO Putative transmembrane protein 829 122-127
1005. PAPARW B6KV99 B6KV99_TOXGO Phosphatidylinositol n-acetylglucosaminyltransferase 348 62-67
1006. PAPARW Q53HZ0 Q53HZ0_TOXGO Phosphatidylinositol N-acetylglucosaminyltransferase subunit C 367 62-67
1007. PARWPR V4ZLX5 V4ZLX5_TOXGO Uncharacterized protein 825 161-166
1008. PESQPL V4ZK24 V4ZK24_TOXGO Uncharacterized protein 709 460-465
1009. ESQPLL B9PHS4 B9PHS4_TOXGO Putative transmembrane protein 297 244-249
1010. ESQPLL V4ZD13 V4ZD13_TOXGO ELMO/CED-12 family protein 527 407-412
1011. SQPLLG V4ZDF4 V4ZDF4_TOXGO Putative PX domain protein 2371 1527-1532
1012. SQPLLG V5AXQ7 V5AXQ7_TOXGO Putative proton ATPase 2261 986-991
1013. QPLLGP V4YVW6 V4YVW6_TOXGO Putative kinesin-related protein 3A 702 447-452
1014. LLGPGA B6KH14 B6KH14_TOXGO AP2 domain transcription factor AP2XII-4 3837 530-535
1015. GPGAGG B6KTZ8 B6KTZ8_TOXGO RNA recognition motif-containing protein 351 265-270
1016. GPGAGG B9PYD2 B9PYD2_TOXGO RNA recognition motif-containing protein 1151 247-252
1017. GPGAGG B9QMH6 B9QMH6_TOXGO RNA pseudouridine synthase superfamily protein 6535 6331-6336
1018. GPGAGG O61001 O61001_TOXGO Heat shock protein 70 642 621-626
1019. GPGAGG Q1JTD1 Q1JTD1_TOXGO Possible RNA-binding protein 982 247-252
1020. GPGAGG Q9U540 Q9U540_TOXGO Chaperonin protein BiP precursor 668 647-652
1021. GPGAGG V4Z7M7 V4Z7M7_TOXGO Transcription factor/nuclear export subunit 2 3000 2889-2894
1022. GPGAGG V4ZKT9 V4ZKT9_TOXGO ELMO/CED-12 family protein 2252 1363-1368
1023. GPGAGG V4ZT48 V4ZT48_TOXGO Uncharacterized protein 830 11-16
1024. GPGAGG V5BAB1 V5BAB1_TOXGO RNA recognition motif-containing protein 539 421-426
1025. PGAGGA B9QPL6 B9QPL6_TOXGO Putative PP2C 673 166-171
1026. PGAGGA O61001 O61001_TOXGO Heat shock protein 70 642 622-627
1027. PGAGGA Q9U540 Q9U540_TOXGO Chaperonin protein BiP precursor 668 648-653
1028. PGAGGA V4YND4 V4YND4_TOXGO eIF2 kinase IF2K-D (Incomplete catalytic triad) 2866 2758-2763
1029. PGAGGA V4ZF62 V4ZF62_TOXGO Uncharacterized protein 1578 835-840
1030. PGAGGA V5BMJ1 V5BMJ1_TOXGO 'chromo' (CHRromatin Organization MOdifier) domain-containing protein 1808 1145-1150
1031. GAGGAG B6KR65 B6KR65_TOXGO Vps51/Vps67 protein 1196 1015-1020
1032. GAGGAG B9PYH2 B9PYH2_TOXGO ATP-dependent RNA helicase, putative 1603 907-912
1033. GAGGAG B9PZU5 B9PZU5_TOXGO Flavoprotein subunit of succinate dehydrogenase 669 80-85
1034. GAGGAG B9Q0T7 B9Q0T7_TOXGO Bromodomain-containing protein 714 278-283
1035. GAGGAG B9QD34 B9QD34_TOXGO Kinesin motor domain-containing protein 1436 996-1001
1036. GAGGAG B9QMH6 B9QMH6_TOXGO RNA pseudouridine synthase superfamily protein 6535 3670-3675, 4431-4436
1037. GAGGAG Q1JTI3 Q1JTI3_TOXGO Ubiquitin-protein ligase 1, putative 8112 6177-6182
1038. GAGGAG Q309Z9 Q309Z9_TOXGO Mitochondrial succinate dehydrogenase flavoprotein subunit precursor 669 80-85
1039. GAGGAG V4YLG5 V4YLG5_TOXGO Helicase associated domain (Ha2) protein 1261 565-570
1040. GAGGAG V4YND4 V4YND4_TOXGO eIF2 kinase IF2K-D (Incomplete catalytic triad) 2866 477-482
1041. GAGGAG V4YRC2 V4YRC2_TOXGO Putative transmembrane protein 4703 1874-1879
1042. GAGGAG V4YXF7 V4YXF7_TOXGO Calcium dependent protein kinase CDPK7 2133 14-19
1043. GAGGAG V4Z214 V4Z214_TOXGO WD domain, G-beta repeat-containing protein 3345 1536-1541
1044. GAGGAG V4Z553 V4Z553_TOXGO HECT-domain (Ubiquitin-transferase) domain-containing protein 8007 6179-6184
1045. GAGGAG V4Z6C0 V4Z6C0_TOXGO LsmAD domain-containing protein 1524 856-861
1046. GAGGAG V4ZBE8 V4ZBE8_TOXGO AP2 domain transcription factor AP2IV-4 2404 802-807
1047. GAGGAG V4ZGU8 V4ZGU8_TOXGO Zinc finger in N-recognin protein 4383 2178-2183
1048. GAGGAG V4ZLW7 V4ZLW7_TOXGO Uncharacterized protein 3800 2334-2339
1049. GAGGAG V4ZUZ7 V4ZUZ7_TOXGO Uncharacterized protein 1822 870-875
1050. GAGGAG V5BBP7 V5BBP7_TOXGO Putative transmembrane protein 1377 1098-1103
1051. AGGAGG B6KH14 B6KH14_TOXGO AP2 domain transcription factor AP2XII-4 3837 2146-2151
1052. AGGAGG B6KN23 B6KN23_TOXGO Putative translation initiation factor SUI1 114 21-26
1053. AGGAGG B9PLQ7 B9PLQ7_TOXGO RNA recognition motif-containing protein 293 203-208
1054. AGGAGG B9PYG4 B9PYG4_TOXGO Sec1 family protein 1053 583-588
1055. AGGAGG B9Q1A8 B9Q1A8_TOXGO 40S ribosomal protein SA 287 256-261
1056. AGGAGG B9Q280 B9Q280_TOXGO Uncharacterized protein 735 383-388
1057. AGGAGG B9QB13 B9QB13_TOXGO MutS domain V domain-containing protein 936 96-101
1058. AGGAGG B9QD34 B9QD34_TOXGO Kinesin motor domain-containing protein 1436 997-1002
1059. AGGAGG B9QMH6 B9QMH6_TOXGO RNA pseudouridine synthase superfamily protein 6535 5869-5874
1060. AGGAGG Q1JT52 Q1JT52_TOXGO Putative uncharacterized protein 694 537-542
1061. AGGAGG Q1JTI3 Q1JTI3_TOXGO Ubiquitin-protein ligase 1, putative 8112 6178-6183
1062. AGGAGG Q38LF1 Q38LF1_TOXGO Eukaryotic translation initiation factor 1 163 66-71
1063. AGGAGG Q8MPF7 RSSA_TOXGO 40S ribosomal protein SA 287 256-261
1064. AGGAGG V4Z4M8 V4Z4M8_TOXGO Amine-terminal region of chorein, A TM vesicle-mediated sorter 13455 10689-10694
1065. AGGAGG V4Z553 V4Z553_TOXGO HECT-domain (Ubiquitin-transferase) domain-containing protein 8007 6180-6185
1066. AGGAGG V4Z5J2 V4Z5J2_TOXGO Uncharacterized protein 882 725-730
1067. AGGAGG V4ZCZ3 V4ZCZ3_TOXGO Uncharacterized protein 1116 354-359
1068. GGAGGT B9Q280 B9Q280_TOXGO Uncharacterized protein 735 384-389
1069. GGAGGT B9QB13 B9QB13_TOXGO MutS domain V domain-containing protein 936 97-102
1070. GGAGGT B9QPA4 B9QPA4_TOXGO Coatomer subunit beta 1103 798-803
1071. GGAGGT Q9U4N3 COPB_TOXGO Coatomer subunit beta 1103 798-803
1072. GGAGGT V4Z5J8 V4Z5J8_TOXGO Condensin complex subunit 1 2466 2309-2314
1073. GGAGGT V4ZJ18 V4ZJ18_TOXGO GIY-YIG catalytic domain-containing protein 1236 847-852
1074. GGAGGT V4ZKZ5 V4ZKZ5_TOXGO AP2 domain transcription factor AP2VIIa-6 2438 1610-1615
1075. GGAGGT V5BCF9 V5BCF9_TOXGO Putative proteophosphoglycan 5, related protein 1474 645-650
1076. GGAGGT V5BDM3 V5BDM3_TOXGO Sec7 domain-containing protein 3987 2532-2537
1077. GGAGGT V5BJ38 V5BJ38_TOXGO RPAP1 family, C-terminal protein 3059 2580-2585
1078. GAGGTG B9PIG8 B9PIG8_TOXGO DEAD/DEAH box ATP-dependent RNA helicase 569 422-427
1079. GAGGTG B9QB13 B9QB13_TOXGO MutS domain V domain-containing protein 936 98-103
1080. GAGGTG Q1JSK4 Q1JSK4_TOXGO Uncharacterized protein 753 648-653
1081. GAGGTG V4Z4I1 V4Z4I1_TOXGO PLU-1 family protein 8088 2392-2397
1082. GAGGTG V4ZAI7 V4ZAI7_TOXGO Uncharacterized protein 753 648-653
1083. AGGTGG B6KJT4 B6KJT4_TOXGO CW-type Zinc Finger protein 1156 826-831
1084. AGGTGG B9QPR3 B9QPR3_TOXGO SWI2/SNF2 SRCAP/Ino80 2924 2279-2284
1085. AGGTGG Q1JSK4 Q1JSK4_TOXGO Uncharacterized protein 753 649-654
1086. AGGTGG Q7Z2C2 Q7Z2C2_TOXGO Snf2-related chromatin remodeling factor SRCAP 2924 2279-2284
1087. AGGTGG V4YP73 V4YP73_TOXGO TBC domain-containing protein 1717 496-501
1088. AGGTGG V4Z4I1 V4Z4I1_TOXGO PLU-1 family protein 8088 2393-2398
1089. AGGTGG V4ZAI7 V4ZAI7_TOXGO Uncharacterized protein 753 649-654
1090. GGTGGA B9PYA4 B9PYA4_TOXGO DEAD/DEAH box helicase domain-containing protein 2434 617-622
1091. GGTGGA B9QLN5 B9QLN5_TOXGO Uncharacterized protein 1856 1527-1532
1092. GGTGGA B9QRA2 B9QRA2_TOXGO GYF domain-containing protein 2331 232-237
1093. GGTGGA Q1JTA7 Q1JTA7_TOXGO Dead/deah box helicase, putative 2471 617-622
1094. GGTGGA V4Z880 V4Z880_TOXGO Uncharacterized protein 4668 1606-1611
1095. GGTGGA V4ZBJ2 V4ZBJ2_TOXGO Tyrosine kinase-like (TKL) protein 1673 447-452
1096. GGTGGA V4ZI71 V4ZI71_TOXGO Uncharacterized protein 2569 1604-1609
1097. GGTGGA V4ZLV5 V4ZLV5_TOXGO Zinc finger, C3HC4 type (RING finger) domain-containing protein 610 522-527
1098. GTGGAG B6K9V8 B6K9V8_TOXGO RNA pseudouridine synthase superfamily protein 2780 1848-1853
1099. GTGGAG B6KGI1 B6KGI1_TOXGO RNA recognition motif protein 2507 431-436
1100. GTGGAG B9PYA4 B9PYA4_TOXGO DEAD/DEAH box helicase domain-containing protein 2434 618-623
1101. GTGGAG B9QIB9 B9QIB9_TOXGO Uncharacterized protein 964 938-943
1102. GTGGAG B9QIW3 B9QIW3_TOXGO AP2 domain transcription factor AP2III-2 1670 1493-1498
1103. GTGGAG Q1JTA7 Q1JTA7_TOXGO Dead/deah box helicase, putative 2471 618-623
1104. GTGGAG V4Z7M7 V4Z7M7_TOXGO Transcription factor/nuclear export subunit 2 3000 2316-2321
1105. GTGGAG V4ZIZ2 V4ZIZ2_TOXGO Putative collagen alpha-1(III) chain, related protein 2799 3-8
1106. GTGGAG V4ZJH2 V4ZJH2_TOXGO RNA recognition motif-containing protein 2070 1213-1218
1107. TGGAGG B9PYA4 B9PYA4_TOXGO DEAD/DEAH box helicase domain-containing protein 2434 619-624
1108. TGGAGG B9QIW3 B9QIW3_TOXGO AP2 domain transcription factor AP2III-2 1670 1494-1499
1109. TGGAGG B9QK32 B9QK32_TOXGO Putative coatomer protein complex, subunit alpha 1300 174-179
1110. TGGAGG Q1JTA7 Q1JTA7_TOXGO Dead/deah box helicase, putative 2471 619-624
1111. TGGAGG V4Z0D1 V4Z0D1_TOXGO Putative FIKK kinase 2887 1092-1097
1112. TGGAGG V4ZJ18 V4ZJ18_TOXGO GIY-YIG catalytic domain-containing protein 1236 846-851
1113. TGGAGG V4ZUI3 V4ZUI3_TOXGO Putative vacuolar protein sorting 11 carboxy-terminal protein 1241 1200-1205
1114. GGAGGG B9PYG4 B9PYG4_TOXGO Sec1 family protein 1053 584-589
1115. GGAGGG B9Q4U1 B9Q4U1_TOXGO Amine-terminal region of chorein, A TM vesicle-mediated sorter 10329 5511-5516
1116. GGAGGG B9Q734 B9Q734_TOXGO Putative signal recognition particle domain protein 1049 133-138
1117. GGAGGG B9QBV0 B9QBV0_TOXGO NOT2 / NOT3 / NOT5 family protein 499 489-494
1118. GGAGGG B9QEC7 B9QEC7_TOXGO CCR4-Not complex component, Not1 protein 2562 1857-1862
1119. GGAGGG B9QFU9 B9QFU9_TOXGO ATP-dependent metallopeptidase HflB subfamily protein 1188 612-617
1120. GGAGGG V4YT75 V4YT75_TOXGO Toxoplasma gondii family E protein 1345 587-592
1121. GGAGGG V4YTC4 V4YTC4_TOXGO Toxoplasma gondii family E protein 1432 648-653
1122. GGAGGG V4YXW0 V4YXW0_TOXGO Toxoplasma gondii family E protein 596 208-213
1123. GGAGGG V4ZBZ6 V4ZBZ6_TOXGO Toxoplasma gondii family E protein 429 189-194
1124. GGAGGG V4ZGB3 V4ZGB3_TOXGO Toxoplasma gondii family E protein 1015 227-232
1125. GGAGGG V4ZHM6 V4ZHM6_TOXGO Uncharacterized protein 2986 2065-2070
1126. GGAGGG V4ZIQ1 V4ZIQ1_TOXGO Uncharacterized protein 1557 1342-1347
1127. GGAGGG V4ZSU4 V4ZSU4_TOXGO Putative transmembrane protein 1274 390-395
1128. GGAGGG V5B6W4 V5B6W4_TOXGO Toxoplasma gondii family E protein 926 660-665
1129. GGAGGG V5B6X0 V5B6X0_TOXGO Toxoplasma gondii family E protein 851 679-684
1130. GGAGGG V5BMI9 V5BMI9_TOXGO Uncharacterized protein 1612 843-848
1131. GAGGGA B9Q0M8 B9Q0M8_TOXGO Leucine zipper-like transcriptional regulator 855 484-489
1132. GAGGGA B9Q8D8 B9Q8D8_TOXGO Chloroquine resistance marker 3946 3806-3811
1133. GAGGGA B9QBV0 B9QBV0_TOXGO NOT2 / NOT3 / NOT5 family protein 499 490-495
1134. GAGGGA Q3S2X2 Q3S2X2_TOXGO Leucine zipper-like transcriptional regulator 855 484-489
1135. GAGGGA V4YUJ7 V4YUJ7_TOXGO Rhoptry kinase family protein ROP21 750 733-738
1136. GAGGGA V4YXF7 V4YXF7_TOXGO Calcium dependent protein kinase CDPK7 2133 10-15
1137. GAGGGA V4YYT0 V4YYT0_TOXGO Sulfite exporter TauE/SafE protein 697 206-211
1138. GAGGGA V4ZCD1 V4ZCD1_TOXGO Zinc finger (CCCH type) motif-containing protein 1298 925-930
1139. GAGGGA V4ZHC6 V4ZHC6_TOXGO Zinc finger, C3HC4 type (RING finger) domain-containing protein 806 591-596
1140. GAGGGA V4ZIQ1 V4ZIQ1_TOXGO Uncharacterized protein 1557 1343-1348
1141. GAGGGA V4ZP44 V4ZP44_TOXGO Uncharacterized protein 544 175-180
1142. GAGGGA V5BAH3 V5BAH3_TOXGO Metal cation transporter, ZIP family protein 717 396-401
1143. GAGGGA V5BMI9 V5BMI9_TOXGO Uncharacterized protein 1612 844-849
1144. AGGGAP V4Z3M3 V4Z3M3_TOXGO Actin-like protein ALP 5 612 400-405
1145. AGGGAP V4ZPY4 V4ZPY4_TOXGO RAVE 1 carboxy-terminal protein 6665 200-205
1146. GGGAPA B6KFT9 B6KFT9_TOXGO Uncharacterized protein 1258 834-839
1147. GGGAPA B9QPU3 B9QPU3_TOXGO Putative RNase protein H 1344 811-816
1148. GGGAPA V4YQ01 V4YQ01_TOXGO Putative cell-cycle-associated protein kinase SRPK 1762 18-23
1149. GGGAPA V4ZL32 V4ZL32_TOXGO Uncharacterized protein 1689 730-735
1150. GGAPAA B6KFT9 B6KFT9_TOXGO Uncharacterized protein 1258 835-840
1151. GGAPAA B9Q560 B9Q560_TOXGO Transport protein Trs120 2958 440-445
1152. GGAPAA V4Z843 V4Z843_TOXGO Uncharacterized protein 1132 957-962
1153. GGAPAA V4ZL32 V4ZL32_TOXGO Uncharacterized protein 1689 731-736
1154. GAPAAP A8CBF6 A8CBF6_TOXGO Delta-aminolevulinic acid synthetase 584 546-551
1155. GAPAAP B9PP05 B9PP05_TOXGO Nucleosome assembly protein (Nap) protein 433 77-82
1156. GAPAAP B9PT26 B9PT26_TOXGO PRP38 family protein 614 44-49
1157. GAPAAP B9PY04 B9PY04_TOXGO Uncharacterized protein 3263 2255-2260
1158. GAPAAP B9Q5R6 B9Q5R6_TOXGO WD domain, G-beta repeat-containing protein 1127 744-749
1159. GAPAAP V4YSI5 V4YSI5_TOXGO Putative Gas41 517 392-397
1160. GAPAAP V4ZCJ4 V4ZCJ4_TOXGO Uncharacterized protein 636 269-274
1161. GAPAAP V4ZFL5 V4ZFL5_TOXGO RNA helicase (UPF2 interacting domain) protein 1539 1141-1146
1162. APAAPP B9PT26 B9PT26_TOXGO PRP38 family protein 614 45-50
1163. APAAPP B9QE64 B9QE64_TOXGO Serine/threonine-protein phosphatase 934 398-403
1164. APAAPP B9QFF6 B9QFF6_TOXGO Putative cell-cycle-associated protein kinase DYRK 1180 161-166
1165. APAAPP V4YQD6 V4YQD6_TOXGO Putative transmembrane protein 391 257-262
1166. APAAPP V4ZEN8 V4ZEN8_TOXGO Uncharacterized protein 4618 3324-3329
1167. APAAPP V4ZFL5 V4ZFL5_TOXGO RNA helicase (UPF2 interacting domain) protein 1539 1142-1147
1168. APAAPP V5B5Z7 V5B5Z7_TOXGO Putative transmembrane protein 1546 1278-1283
1169. PAAPPP B6K9E1 B6K9E1_TOXGO Ribosomal L1p/L10e family protein 469 362-367
1170. PAAPPP B6KP56 B6KP56_TOXGO GTP-binding family protein 1064 771-776
1171. PAAPPP Q1JSQ7 Q1JSQ7_TOXGO GTP binding protein, putative 1060 767-772
1172. PAAPPP V4ZCA3 V4ZCA3_TOXGO DEAD/DEAH box helicase domain-containing protein 1144 27-32
1173. PAAPPP V4ZFL5 V4ZFL5_TOXGO RNA helicase (UPF2 interacting domain) protein 1539 1143-1148
1174. PAAPPP V4ZI75 V4ZI75_TOXGO Polycystin cation channel protein 1684 1214-1219
1175. PAAPPP V4ZPX5 V4ZPX5_TOXGO Surp module domain-containing protein 658 533-538
1176. AAPPPC V4Z394 V4Z394_TOXGO SNF7 family protein 265 180-185
1177. CRAAPP B9QDK9 B9QDK9_TOXGO 3'-5' exonuclease domain-containing protein 1353 350-355
1178. RAAPPP B9QDK9 B9QDK9_TOXGO 3'-5' exonuclease domain-containing protein 1353 351-356
1179. RAAPPP C0L7I2 C0L7I2_TOXGO Plastid replication-repair enzyme 2579 221-226
1180. RAAPPP V5B8Q0 V5B8Q0_TOXGO Putative helicase 2573 221-226
1181. LEPSPS B9QQF5 B9QQF5_TOXGO Uncharacterized protein 976 350-355
1182. LEPSPS V4YNZ5 V4YNZ5_TOXGO Putative glycogen synthase 3004 589-594
1183. LEPSPS V4ZFJ6 V4ZFJ6_TOXGO HECT-domain (Ubiquitin-transferase) domain-containing protein 4155 2948-2953
1184. EPSPSD V4ZWR7 V4ZWR7_TOXGO Man1-Src1p-carboxy-terminal domain protein 595 3-8
1185. PSPSDS B9QMX0 B9QMX0_TOXGO WD domain, G-beta repeat-containing protein 697 425-430
1186. PSPSDS V5BB89 V5BB89_TOXGO Putative transmembrane protein 3658 822-827
1187. SPSDSE V4Z2E4 V4Z2E4_TOXGO Pre-RNA processing PIH1/Nop17 protein 692 464-469
1188. SPSDSE V4Z5Q0 V4Z5Q0_TOXGO GDP-L-fucose synthetase 445 119-124
1189. SPSDSE V4ZC58 V4ZC58_TOXGO Uncharacterized protein 1223 1169-1174
1190. SPSDSE V4ZDU9 V4ZDU9_TOXGO Putative transmembrane protein 3661 1613-1618
1191. SPSDSE V4ZJZ9 V4ZJZ9_TOXGO ATPase, AAA family protein 3910 1147-1152
1192. PSDSED V4Z9Z0 V4Z9Z0_TOXGO Uncharacterized protein 1080 1067-1072
1193. PSDSED V4ZJZ9 V4ZJZ9_TOXGO ATPase, AAA family protein 3910 1148-1153
1194. SDSEDS B6KAZ7 B6KAZ7_TOXGO Myosin J 2532 703-708
1195. SDSEDS B6KSW5 B6KSW5_TOXGO Zinc finger, C3HC4 type (RING finger) domain-containing protein 1027 170-175
1196. SDSEDS B9QAM8 B9QAM8_TOXGO Uncharacterized protein 2194 2107-2112
1197. SDSEDS V4ZJD0 V4ZJD0_TOXGO Uncharacterized protein 956 350-355
1198. SDSEDS V4ZSF0 V4ZSF0_TOXGO Transporter, major facilitator family protein 719 287-292
1199. SDSEDS V4ZW70 V4ZW70_TOXGO Uncharacterized protein 5655 2359-2364
1200. DSEDSE B6KFV0 B6KFV0_TOXGO Uncharacterized protein 1266 683-688
1201. DSEDSE B6KSW5 B6KSW5_TOXGO Zinc finger, C3HC4 type (RING finger) domain-containing protein 1027 171-176
1202. DSEDSE B9PYC3 B9PYC3_TOXGO Eukaryotic translation initiation factor 3 subunit C 1104 226-231
1203. DSEDSE V4Z1I1 V4Z1I1_TOXGO Eukaryotic translation initiation factor 3 subunit C 971 226-231
1204. DSEDSE V5B4G9 V5B4G9_TOXGO Uncharacterized protein 2072 1315-1320
1205. SEDSES B9Q591 B9Q591_TOXGO Putative transmembrane protein 7354 3655-3660
1206. SEDSES B9QEQ3 B9QEQ3_TOXGO G-protein beta WD-40 repeat containing protein 607 249-254
1207. SEDSES Q1JSF3 Q1JSF3_TOXGO Uncharacterized protein 4600 2331-2336
1208. SEDSES V4YSU1 V4YSU1_TOXGO Protein disulfide-isomerase domain-containing protein 1133 553-558
1209. SEDSES V4Z6H5 V4Z6H5_TOXGO Putative glutamic acid-rcih protein 4436 1856-1861
1210. SEDSES V5B724 V5B724_TOXGO DEAD/DEAH box helicase domain-containing protein 1454 116-121
1211. EDSESL K7WT78 K7WT78_TOXGO DHHC15 1327 238-243
1212. EDSESL Q1JT20 Q1JT20_TOXGO Zinc finger domain containing protein, putative precursor 1100 112-117
1213. EDSESL V4Z536 V4Z536_TOXGO Macro domain-containing protein 692 305-310
1214. EDSESL V5B086 V5B086_TOXGO DHHC zinc finger domain-containing protein 1115 238-243
1215. DSESLG V4ZI71 V4ZI71_TOXGO Uncharacterized protein 2569 2483-2488
1216. SESLGG V4ZBI5 V4ZBI5_TOXGO Uncharacterized protein 457 64-69
1217. ESLGGA B6KBF1 B6KBF1_TOXGO JmjC domain-containing protein C2orf60 903 185-190
1218. ESLGGA V5AWR3 V5AWR3_TOXGO RAP domain-containing protein 1385 992-997
1219. SLGGAS B9QEC7 B9QEC7_TOXGO CCR4-Not complex component, Not1 protein 2562 2420-2425
1220. SLGGAS V4YNM0 V4YNM0_TOXGO Uncharacterized protein 2518 169-174
1221. SLGGAS V5BJC6 V5BJC6_TOXGO EF hand domain-containing protein 6368 6036-6041
1222. LGGASL B9Q8D8 B9Q8D8_TOXGO Chloroquine resistance marker 3946 3006-3011
1223. LGGASL V4ZEJ1 V4ZEJ1_TOXGO WD domain, G-beta repeat-containing protein 672 174-179
1224. GGASLG B9QEV1 B9QEV1_TOXGO Uncharacterized protein 1937 1386-1391
1225. GGASLG V4ZJ24 V4ZJ24_TOXGO Phospholipase, patatin family protein 994 628-633
1226. GGASLG V5BGS1 V5BGS1_TOXGO Zinc finger (CCCH type) motif-containing protein 3460 1749-1754
1227. GASLGG B6KB25 B6KB25_TOXGO Homocysteine s-methyltransferase domain-containing protein 434 163-168
1228. GASLGG B9QEC7 B9QEC7_TOXGO CCR4-Not complex component, Not1 protein 2562 905-910
1229. GASLGG V4YN30 V4YN30_TOXGO Putative vacuolar protein sorting-associated protein 8650 8001-8006
1230. GASLGG V4YXF7 V4YXF7_TOXGO Calcium dependent protein kinase CDPK7 2133 1973-1978
1231. GASLGG V4Z2F7 V4Z2F7_TOXGO Putative transmembrane protein 2862 2815-2820
1232. ASLGGL B9QPR3 B9QPR3_TOXGO SWI2/SNF2 SRCAP/Ino80 2924 428-433
1233. ASLGGL Q7Z2C2 Q7Z2C2_TOXGO Snf2-related chromatin remodeling factor SRCAP 2924 428-433
1234. ASLGGL V5BBB2 V5BBB2_TOXGO Putative transmembrane protein 1241 879-884
1235. SLGGLE Q1JTC2 Q1JTC2_TOXGO Putative uncharacterized protein 1737 167-172
1236. SLGGLE V4ZA61 V4ZA61_TOXGO Putative histone lysine methyltransferase, SET 1737 167-172
1237. AERLGP B9QEC7 B9QEC7_TOXGO CCR4-Not complex component, Not1 protein 2562 2121-2126
1238. AERLGP V5BDM3 V5BDM3_TOXGO Sec7 domain-containing protein 3987 2988-2993
1239. ERLGPP V4ZF87 V4ZF87_TOXGO AP2 domain transcription factor AP2X-6 4495 2231-2236
1240. RLGPPP V4ZD88 V4ZD88_TOXGO Transporter, major facilitator family protein 1302 728-733
1241. LGPPPG B9Q6C0 B9Q6C0_TOXGO Filamin/ABP280 repeat-containing protein 2728 295-300
1242. LGPPPG V5BAB1 V5BAB1_TOXGO RNA recognition motif-containing protein 539 494-499
1243. GPPPGR B9PSE3 B9PSE3_TOXGO Zinc finger, c2h2 type domain-containing protein 287 241-246
1244. GPPPGR V5B3Z0 V5B3Z0_TOXGO FATC domain-containing protein 6012 4132-4137
1245. GGWRAG V4ZJ96 V4ZJ96_TOXGO Uncharacterized protein 1123 711-716
1246. YLPPRS V4Z9W6 V4Z9W6_TOXGO Leucine rich repeat-containing protein 1333 650-655
1247. PPRSGP B9Q591 B9Q591_TOXGO Putative transmembrane protein 7354 6105-6110
1248. PPRSGP B9Q851 B9Q851_TOXGO SSXT (Amine-terminal region) protein 826 559-564
1249. PPRSGP B9QIS7 B9QIS7_TOXGO Uncharacterized protein 295 154-159
1250. RSGPAA B9Q792 B9Q792_TOXGO Uncharacterized protein 4118 2816-2821
1251. RSGPAA V5BFH2 V5BFH2_TOXGO Uncharacterized protein 785 225-230
1252. ASLELL B9QI15 B9QI15_TOXGO Phosphoglycerate kinase 418 399-404
1253. ASLELL Q1KSE7 Q1KSE7_TOXGO Phosphoglycerate kinase 551 528-533
1254. ASLELL Q1KSE8 Q1KSE8_TOXGO Phosphoglycerate kinase 416 398-403
1255. ASLELL V4Z1X6 V4Z1X6_TOXGO Uncharacterized protein 1516 1138-1143
1256. ASLELL V4ZCI3 V4ZCI3_TOXGO Phosphoglycerate kinase 593 570-575
1257. ASLELL V4ZEX6 V4ZEX6_TOXGO Uncharacterized protein 697 366-371
1258. SLELLP B9Q479 B9Q479_TOXGO WD domain, G-beta repeat-containing protein 3633 3457-3462
1259. LELLPP B9PTW7 B9PTW7_TOXGO Ubiquinol-cytochrome c reductase 234 91-96
1260. LELLPP V4Z6D3 V4Z6D3_TOXGO Putative ribonuclease z 1062 1048-1053
1261. LLPPPR B6KRX2 B6KRX2_TOXGO Putative transmembrane protein 412 88-93
1262. LLPPPR B9QF02 B9QF02_TOXGO Uncharacterized protein 1847 71-76
1263. LLPPPR V4YRE6 V4YRE6_TOXGO PHD-finger domain-containing protein 4647 2389-2394
1264. LLPPPR V4ZKY1 V4ZKY1_TOXGO Putative transmembrane protein 831 522-527
1265. LLPPPR V4ZSE4 V4ZSE4_TOXGO WD domain, G-beta repeat-containing protein 4664 852-857
1266. LLPPPR V5BDZ6 V5BDZ6_TOXGO Uncharacterized protein 4591 120-125
1267. PPRHLS V4Z0M3 V4Z0M3_TOXGO Putative WDSUB1 family SAM domain protein 1912 717-722
1268. PRHLSC V4Z0M3 V4Z0M3_TOXGO Putative WDSUB1 family SAM domain protein 1912 718-723
1269. HDGLDG B9PGW0 B9PGW0_TOXGO WD domain, G-beta repeat-containing protein 565 484-489
1270. DGGWWA V4ZSS2 V4ZSS2_TOXGO Inositol phospholipid synthesis protein Scs3p 1642 1312-1317
1271. GWWAPP V4ZMK6 V4ZMK6_TOXGO TRAPP trafficking subunit Trs65 801 472-477
1272. APPPPP B6K9F2 B6K9F2_TOXGO Zinc knuckle domain-containing protein 723 325-330
1273. APPPPP B6KFV0 B6KFV0_TOXGO Uncharacterized protein 1266 888-893
1274. APPPPP B6KGY9 B6KGY9_TOXGO Uncharacterized protein 2303 2080-2085
1275. APPPPP B6KP07 B6KP07_TOXGO DnaJ C terminal region domain-containing protein 1519 1404-1409
1276. APPPPP B6KP56 B6KP56_TOXGO GTP-binding family protein 1064 773-778
1277. APPPPP B6KQW9 B6KQW9_TOXGO Sec16 Sec23-binding domain protein 2142 1814-1819
1278. APPPPP B6KTX9 B6KTX9_TOXGO Putative methyltransferase MTA70 819 373-378, 410-415
1279. APPPPP B9PJA8 B9PJA8_TOXGO Reactive oxygen species modulator 1 263 42-47
1280. APPPPP B9PYC0 B9PYC0_TOXGO Toxoplasma gondii family D protein precursor 565 556-561
1281. APPPPP B9Q700 B9Q700_TOXGO Nuclear fragile X mental retardation-interacting protein 1 830 6-11
1282. APPPPP B9QCY2 B9QCY2_TOXGO Putative nuclear cap-binding protein 1201 709-714
1283. APPPPP B9QEJ0 B9QEJ0_TOXGO Putative histone lysine methyltransferase, SET 1632 1489-1494, 1500-1505, 1511-1516, 1544-1549, 1566-1571, 1588-1593
1284. APPPPP B9QEK9 B9QEK9_TOXGO HEAT repeat-containing protein 910 8-13
1285. APPPPP B9QFB5 B9QFB5_TOXGO Uncharacterized protein 1207 8-13
1286. APPPPP B9QH19 B9QH19_TOXGO Putative transmembrane protein 1423 209-214
1287. APPPPP B9QHV0 B9QHV0_TOXGO Oocyst wall protein 782 749-754
1288. APPPPP B9QIC7 B9QIC7_TOXGO RNA recognition motif-containing protein 1216 1060-1065
1289. APPPPP B9QIG0 B9QIG0_TOXGO Putative elongation factor Tu 586 21-26
1290. APPPPP B9QIY1 B9QIY1_TOXGO CMGC kinase, CDK family 1310 750-755
1291. APPPPP Q1JSQ7 Q1JSQ7_TOXGO GTP binding protein, putative 1060 769-774
1292. APPPPP Q1JSW5 Q1JSW5_TOXGO DnaJ protein, putative 539 424-429
1293. APPPPP Q1JT54 Q1JT54_TOXGO Putative uncharacterized protein 1990 708-713
1294. APPPPP Q1JTD3 Q1JTD3_TOXGO Putative uncharacterized protein 1998 1814-1819
1295. APPPPP V4YN30 V4YN30_TOXGO Putative vacuolar protein sorting-associated protein 8650 3042-3047
1296. APPPPP V4Z632 V4Z632_TOXGO Uncharacterized protein 1391 620-625
1297. APPPPP V4Z6Y4 V4Z6Y4_TOXGO SWI2/SNF2-containing protein RAD5 1748 893-898
1298. APPPPP V4Z9Y8 V4Z9Y8_TOXGO Uncharacterized protein 2154 708-713
1299. APPPPP V4ZAK3 V4ZAK3_TOXGO Uncharacterized protein 3378 864-869
1300. APPPPP V4ZEC4 V4ZEC4_TOXGO Uncharacterized protein 1155 703-708
1301. APPPPP V4ZNH6 V4ZNH6_TOXGO Putative aquarius 2250 40-45
1302. APPPPP V5B1K2 V5B1K2_TOXGO Uncharacterized protein 1673 516-521
1303. APPPPP V5BLD5 V5BLD5_TOXGO Adaptin c-terminal domain-containing protein 1672 22-27
1304. AAGPLP V4ZJY9 V4ZJY9_TOXGO Pre-rRNA processing protein 2863 1888-1893
1305. AAGPLP V4ZRV3 V4ZRV3_TOXGO MCM2/3/5 family protein 1385 1106-1111
1306. AAGPLP V5AY70 V5AY70_TOXGO Putative transmembrane protein 2314 2042-2047
1307. AGPLPR B9Q6I9 B9Q6I9_TOXGO Uncharacterized protein 5651 1728-1733
1308. AGPLPR V4ZFL6 V4ZFL6_TOXGO DnaJ domain-containing protein 755 359-364
1309. GPLPRR B9QGJ2 B9QGJ2_TOXGO Plasma-membrane choline transporter 1060 383-388
1310. PLPRRR B9QGJ2 B9QGJ2_TOXGO Plasma-membrane choline transporter 1060 384-389
1311. PLPRRR V4YY35 V4YY35_TOXGO Uncharacterized protein 410 240-245
1312. PLPRRR V4ZIB1 V4ZIB1_TOXGO Putative transmembrane protein 1818 592-597
1313. PLPRRR V5B7E4 V5B7E4_TOXGO Putative transmembrane protein 2871 2591-2596
1314. PLPRRR V5BHA1 V5BHA1_TOXGO Uncharacterized protein 5632 2764-2769
1315. PLPRRR V5BJ63 V5BJ63_TOXGO Uncharacterized protein 1998 1083-1088
1316. LPRRRA V4ZLE5 V4ZLE5_TOXGO Uncharacterized protein 2186 917-922
1317. LPRRRA V5BBV7 V5BBV7_TOXGO RNB family domain-containing protein 2425 143-148
1318. PRRRAR Q1JTI3 Q1JTI3_TOXGO Ubiquitin-protein ligase 1, putative 8112 1702-1707
1319. PRRRAR V4Z553 V4Z553_TOXGO HECT-domain (Ubiquitin-transferase) domain-containing protein 8007 1702-1707
1320. RRRARC B9QFW3 B9QFW3_TOXGO AP2 domain transcription factor AP2VIIa-7 3112 2514-2519
1321. RRRARC V4ZWR7 V4ZWR7_TOXGO Man1-Src1p-carboxy-terminal domain protein 595 175-180
1322. RRARCG B9QFW3 B9QFW3_TOXGO AP2 domain transcription factor AP2VIIa-7 3112 2515-2520
1323. ARCGCP B9QHC5 B9QHC5_TOXGO Cation channel family transporter 1162 792-797
1324. GCPRSH B9QFP4 B9QFP4_TOXGO Uncharacterized protein 330 58-63
1325. HPHRPR V4ZGU1 V4ZGU1_TOXGO Uncharacterized protein 2180 107-112
1326. RPRASH B6KAZ3 B6KAZ3_TOXGO Putative proteophosphoglycan 5, related protein 2250 1333-1338
1327. HRTPAA B6KAP5 B6KAP5_TOXGO Ankyrin repeat-containing protein 1158 777-782
1328. HRTPAA V4Z3Y8 V4Z3Y8_TOXGO Uncharacterized protein 666 148-153
1329. RTPAAA B6KAP5 B6KAP5_TOXGO Ankyrin repeat-containing protein 1158 778-783
1330. RTPAAA B9QCR4 B9QCR4_TOXGO Pentatricopeptide repeat domain-containing protein 2141 17-22
1331. RTPAAA B9QFR6 B9QFR6_TOXGO NUC173 domain protein 1915 433-438
1332. RTPAAA V4Z523 V4Z523_TOXGO Smg-4/UPF3 family protein 594 507-512
1333. RTPAAA V4ZDW7 V4ZDW7_TOXGO Mcm10 replication factor 872 669-674
1334. TPAAAA B6KHU4 B6KHU4_TOXGO Heat shock protein 728 41-46
1335. TPAAAA B6KP82 B6KP82_TOXGO SAG-related sequence SRS11 190 137-142
1336. TPAAAA B9Q843 B9Q843_TOXGO Histone lysine methyltransferase SET1 7555 4662-4667
1337. TPAAAA B9QCR4 B9QCR4_TOXGO Pentatricopeptide repeat domain-containing protein 2141 18-23
1338. TPAAAA B9QM95 B9QM95_TOXGO Putative transmembrane protein 407 301-306
1339. TPAAAA B9QPV3 B9QPV3_TOXGO Putative 200 kDa antigen p200 1207 953-958
1340. TPAAAA Q4JGN8 Q4JGN8_TOXGO Cytoplasmic protein Cyt16 205 7-12
1341. TPAAAA Q9XYH0 Q9XYH0_TOXGO SAG2 related antigen SAG2B 190 137-142
1342. TPAAAA R4JAM5 R4JAM5_TOXGO Surface antigen 2B 130 77-82
1343. TPAAAA V4YJL4 V4YJL4_TOXGO Histone lysine methyltransferase SET2 2286 294-299
1344. TPAAAA V4YZR2 V4YZR2_TOXGO RNA pseudouridine synthase superfamily protein 777 146-151
1345. TPAAAA V4ZBJ0 V4ZBJ0_TOXGO Uncharacterized protein 3206 1246-1251
1346. TPAAAA V4ZBQ6 V4ZBQ6_TOXGO Putative transmembrane domain protein 878 518-523
1347. TPAAAA V4ZHR6 V4ZHR6_TOXGO WD domain, G-beta repeat-containing protein 1581 864-869
1348. TPAAAA V4ZLT7 V4ZLT7_TOXGO Uncharacterized protein 1920 978-983
1349. PAAAAP B6KAE3 B6KAE3_TOXGO Emp24/gp25L/p24 family protein 601 220-225
1350. PAAAAP B6KAR1 B6KAR1_TOXGO WD domain, G-beta repeat-containing protein 845 138-143
1351. PAAAAP B9PKQ4 B9PKQ4_TOXGO Ribosomal protein RPP1 179 142-147
1352. PAAAAP B9PSH4 B9PSH4_TOXGO CHCH domain-containing protein 149 100-105
1353. PAAAAP B9Q6B6 B9Q6B6_TOXGO Uncharacterized protein 1988 104-109
1354. PAAAAP B9Q7P3 B9Q7P3_TOXGO Uncharacterized protein 2473 545-550
1355. PAAAAP B9QIQ8 B9QIQ8_TOXGO DNA repair protein endonuclease SAE2/CtIP carboxy-terminal protein 1801 1387-1392
1356. PAAAAP V4YJL4 V4YJL4_TOXGO Histone lysine methyltransferase SET2 2286 295-300
1357. PAAAAP V4Z445 V4Z445_TOXGO Histone acetyltransferase TAF1/250 2775 305-310
1358. PAAAAP V4Z6B3 V4Z6B3_TOXGO Putative transmembrane protein 462 334-339
1359. PAAAAP V4ZAK3 V4ZAK3_TOXGO Uncharacterized protein 3378 1091-1096
1360. PAAAAP V4ZSV5 V4ZSV5_TOXGO Uncharacterized protein 864 217-222
1361. PAAAAP V4ZTH9 V4ZTH9_TOXGO Putative transmembrane protein 1278 1013-1018
1362. PAAAAP V4ZV93 V4ZV93_TOXGO Uncharacterized protein 4618 357-362
1363. PAAAAP V5BAX7 V5BAX7_TOXGO Uncharacterized protein 1633 156-161
1364. PAAAAP V5BKN7 V5BKN7_TOXGO Putative GCN1 3416 886-891
1365. PAAAAP V5BL04 V5BL04_TOXGO SWI2/SNF2-containing protein RAD54 866 803-808
1366. AAAPHH B9QEM9 B9QEM9_TOXGO Putative ribonuclease ZC3H12D 1968 367-372
1367. HHHRHR B9QIX9 B9QIX9_TOXGO Uncharacterized protein 1904 1088-1093
1368. RHRRAA V4Z1L7 V4Z1L7_TOXGO Putative transmembrane protein 1241 984-989
1369. HRRAAG V4YZ80 V4YZ80_TOXGO HECT-domain (Ubiquitin-transferase) domain-containing protein 15897 3857-3862
1370. HRRAAG V4ZNI2 V4ZNI2_TOXGO WW domain protein 2115 285-290
1371. RRAAGG B6KRJ8 B6KRJ8_TOXGO Uncharacterized protein 2419 1361-1366
1372. RRAAGG V4YYZ6 V4YYZ6_TOXGO Ribosome biogenesis protein BOP1 homolog 1049 867-872
1373. RRAAGG V4YZ80 V4YZ80_TOXGO HECT-domain (Ubiquitin-transferase) domain-containing protein 15897 3858-3863
1374. RRAAGG V4Z3G5 V4Z3G5_TOXGO Uncharacterized protein 893 753-758
1375. RRAAGG V4ZAX6 V4ZAX6_TOXGO Uncharacterized protein 8643 6573-6578
1376. RRAAGG V5BHA1 V5BHA1_TOXGO Uncharacterized protein 5632 5555-5560
1377. RAAGGW V5BJD1 V5BJD1_TOXGO tRNA nucleotidyltransferase/poly(A) polymerase family protein 1114 423-428
1378. DLPPPA B9QIC6 B9QIC6_TOXGO Uncharacterized protein 585 317-322
1379. LPPPAP Q1JSM9 Q1JSM9_TOXGO Uncharacterized protein 3444 1481-1486
1380. LPPPAP V4YRE6 V4YRE6_TOXGO PHD-finger domain-containing protein 4647 3202-3207
1381. LPPPAP V4YU57 V4YU57_TOXGO Adenosine-deaminase domain protein 1494 71-76
1382. LPPPAP V4Z6U1 V4Z6U1_TOXGO Uncharacterized protein 494 355-360
1383. LPPPAP V4Z7L4 V4Z7L4_TOXGO FAD binding domain-containing protein 1390 962-967
1384. LPPPAP V4Z923 V4Z923_TOXGO RNase P subunit p30 632 52-57
1385. LPPPAP V4ZA44 V4ZA44_TOXGO Site-specific recombinase, phage integrase family protein 1022 126-131
1386. LPPPAP V4ZG44 V4ZG44_TOXGO Putative transmembrane protein 2478 739-744
1387. LPPPAP V4ZGX1 V4ZGX1_TOXGO Uncharacterized protein 3917 3284-3289
1388. LPPPAP V4ZIY3 V4ZIY3_TOXGO Rhoptry kinase family protein ROP27 975 157-162
1389. LPPPAP V5AWP5 V5AWP5_TOXGO HEAT repeat-containing protein 1766 68-73
1390. PPPAPT B9QG52 B9QG52_TOXGO Uncharacterized protein 1051 400-405
1391. PPPAPT V4Z4A2 V4Z4A2_TOXGO Putative oxidoreductase 1318 113-118
1392. PPPAPT V4ZEN1 V4ZEN1_TOXGO Putative transmembrane protein 329 14-19
1393. PPPAPT V5BL04 V5BL04_TOXGO SWI2/SNF2-containing protein RAD54 866 115-120
1394. PPAPTS B9QH54 B9QH54_TOXGO Phospho-2-dehydro-3-deoxyheptonate aldolase,related protein 1052 42-47
1395. PPAPTS V5BL04 V5BL04_TOXGO SWI2/SNF2-containing protein RAD54 866 116-121
1396. PAPTSR B9QH54 B9QH54_TOXGO Phospho-2-dehydro-3-deoxyheptonate aldolase,related protein 1052 43-48
1397. APTSRS B6KR48 B6KR48_TOXGO Putative transmembrane protein 517 317-322
1398. APTSRS B9QFC2 B9QFC2_TOXGO Uncharacterized protein 897 77-82
1399. PTSRSL A4UQJ5 A4UQJ5_TOXGO Apicoplast glyceraldehyde-3-phosphate dehydrogenase 2 995 43-48
1400. PTSRSL V5BBU1 V5BBU1_TOXGO Carrier superfamily protein 682 78-83
1401. PTSRSL V5BHW3 V5BHW3_TOXGO PGAP1 family protein 2784 80-85
1402. PTSRSL V5BMS8 V5BMS8_TOXGO Glyceraldehyde-3-phosphate dehydrogenase GAPDH2 995 43-48
1403. SRSLED V4Z3G1 V4Z3G1_TOXGO Dynein heavy chain family protein 4610 392-397
1404. RSLEDL V4Z3X9 V4Z3X9_TOXGO Uncharacterized protein 2041 1458-1463
1405. SLEDLS V5BJM2 V5BJM2_TOXGO Putative DNA ligase (NAD+) 1975 698-703
1406. LEDLSS B9QIC6 B9QIC6_TOXGO Uncharacterized protein 585 204-209
1407. LSSCPR B6KU03 B6KU03_TOXGO Putative 50S ribosomal protein L17 552 106-111
1408. LSSCPR V4YZ80 V4YZ80_TOXGO HECT-domain (Ubiquitin-transferase) domain-containing protein 15897 3281-3286
1409. SSCPRA B9Q6Q4 B9Q6Q4_TOXGO Uncharacterized protein 1285 915-920
1410. SSCPRA V4Z1T8 V4Z1T8_TOXGO Uncharacterized protein 4885 2705-2710
1411. SCPRAA V4Z1T8 V4Z1T8_TOXGO Uncharacterized protein 4885 2706-2711
1412. SCPRAA V4ZV93 V4ZV93_TOXGO Uncharacterized protein 4618 1837-1842
1413. PRAAPA B9PUT9 B9PUT9_TOXGO Putative eukaryotic initiation factor-3 subunit 10 1033 912-917
1414. PRAAPA B9QND4 B9QND4_TOXGO Uncharacterized protein 1638 188-193
1415. PRAAPA V4Z251 V4Z251_TOXGO Uncharacterized protein 3633 778-783
1416. PRAAPA V4Z4Q0 V4Z4Q0_TOXGO Uncharacterized protein 864 194-199
1417. RAAPAR Q1JSJ1 Q1JSJ1_TOXGO Uncharacterized protein 3577 779-784
1418. RAAPAR V4Z251 V4Z251_TOXGO Uncharacterized protein 3633 779-784
1419. AAPARR V4ZLW7 V4ZLW7_TOXGO Uncharacterized protein 3800 1133-1138
1420. AAPARR V4ZSL1 V4ZSL1_TOXGO Putative transmembrane protein 1892 1781-1786
1421. AAPARR V5BK29 V5BK29_TOXGO Transporter, major facilitator family protein 847 11-16
1422. APARRL B9QMX3 B9QMX3_TOXGO Toxoplasma gondii family A protein 668 618-623
1423. APARRL V4Z2M2 V4Z2M2_TOXGO Putative transmembrane protein 668 143-148
1424. APARRL V4Z4V2 V4Z4V2_TOXGO Putative flagellar associated protein 187 19-24
1425. APARRL V4ZR91 V4ZR91_TOXGO Uncharacterized protein 3903 3732-3737
1426. PARRLT B9QMX3 B9QMX3_TOXGO Toxoplasma gondii family A protein 668 619-624
1427. ARRLTG B6KNZ3 B6KNZ3_TOXGO Pyridine nucleotide-disulfide oxidoreductase domain-containing protein 664 342-347
1428. ARRLTG Q1JSY0 Q1JSY0_TOXGO Ferrodoxin reductase-like protein precursor 612 290-295
1429. RRLTGP V4YPB3 V4YPB3_TOXGO Uncharacterized protein 1552 1340-1345
1430. RRLTGP V4ZPK3 V4ZPK3_TOXGO Uncharacterized protein 1917 454-459
1431. GPSRHA K7X7G3 K7X7G3_TOXGO Palmitoyltransferase 537 185-190
1432. SRHARR V4Z7H9 V4Z7H9_TOXGO Ion channel protein 2540 2339-2344
1433. PPLPTA A8CBG8 A8CBG8_TOXGO Uroporphyrinogen III synthase 1275 261-266
1434. PPLPTA V4Z8Y1 V4Z8Y1_TOXGO Putative transmembrane protein 1276 261-266
1435. PLPTAS B9QE64 B9QE64_TOXGO Serine/threonine-protein phosphatase 934 538-543
1436. PLPTAS V4Z4I1 V4Z4I1_TOXGO PLU-1 family protein 8088 2032-2037
1437. PLPTAS V4ZEK9 V4ZEK9_TOXGO Putative transmembrane protein 579 415-420
1438. LPTASH B9Q536 B9Q536_TOXGO Myosin I 1821 1461-1466
1439. LPTASH B9QBS0 B9QBS0_TOXGO WD domain, G-beta repeat-containing protein 1017 698-703
1440. LPTASH V4YNN3 V4YNN3_TOXGO Putative Ubiquinol-cytochrome c chaperone 697 125-130
1441. LPTASH V4ZEK9 V4ZEK9_TOXGO Putative transmembrane protein 579 416-421
1442. LPTASH V4ZIF3 V4ZIF3_TOXGO Putative proteophosphoglycan 5, related protein 1564 1130-1135
1443. PTASHR B9QBS0 B9QBS0_TOXGO WD domain, G-beta repeat-containing protein 1017 699-704
1444. PTASHR V4ZDQ1 V4ZDQ1_TOXGO Putative myosin heavy chain 244 207-212
1445. TASHRR V5BEH6 V5BEH6_TOXGO Uncharacterized protein 1275 694-699
1446. ASHRRH V4ZKB2 V4ZKB2_TOXGO Uncharacterized protein 605 27-32
1447. RHRGGD V4Z4W1 V4Z4W1_TOXGO Putative target of rapamycin (TOR) 4922 4099-4104
1448. RHRGGD V4ZMJ1 V4ZMJ1_TOXGO Uncharacterized protein 4969 543-548
1449. RGGDLG B9Q591 B9Q591_TOXGO Putative transmembrane protein 7354 6366-6371
1450. RGGDLG V4ZI16 V4ZI16_TOXGO Uncharacterized protein 1216 468-473
1451. RGGDLG V4ZJ50 V4ZJ50_TOXGO Oligomeric complex protein COG6 1298 731-736
1452. LGTRRG V5B323 V5B323_TOXGO Uncharacterized protein 4188 917-922
1453. GTRRGS B9Q843 B9Q843_TOXGO Histone lysine methyltransferase SET1 7555 371-376
1454. TRRGSA V4Z0D1 V4Z0D1_TOXGO Putative FIKK kinase 2887 2148-2153
1455. TRRGSA V4ZJI9 V4ZJI9_TOXGO DnaJ domain-containing protein 3043 2499-2504
1456. TRRGSA V4ZLZ2 V4ZLZ2_TOXGO Hydrolase 3142 55-60
1457. RRGSAH B9Q504 B9Q504_TOXGO Uncharacterized protein 4030 75-80
1458. RRGSAH B9QPG6 B9QPG6_TOXGO SET domain containing lysine methyltransferase KMTox 880 291-296
1459. RRGSAH V4Z7A2 V4Z7A2_TOXGO Uncharacterized protein 1109 601-606
1460. RRGSAH V4ZGG2 V4ZGG2_TOXGO Uncharacterized protein 3620 399-404
1461. AHFSSL V4Z7U1 V4Z7U1_TOXGO HECT-domain (Ubiquitin-transferase) domain-containing protein 1978 818-823
1462. HFSSLE V4Z0V9 V4Z0V9_TOXGO Uncharacterized protein 2663 583-588
1463. HFSSLE V4ZQC7 V4ZQC7_TOXGO Putative transmembrane protein 1718 1038-1043

**NMDA 3A**

1. MRRLSL V4ZBH1 V4ZBH1_TOXGO Uncharacterized protein 3736 957-962
2. MRRLSL V4ZGP4 V4ZGP4_TOXGO Mannosyl-oligosaccharide glucosidase 1384 1-6
3. LSLWWL B9Q5Y8 B9Q5Y8_TOXGO Putative transmembrane protein 662 368-373
4. LLSRVC B6K9V5 B6K9V5_TOXGO Uncharacterized protein 1315 731-736
5. LLSRVC B9PRN1 B9PRN1_TOXGO Putative phosphate carrier 479 61-66
6. LLSRVC V4ZTD6 V4ZTD6_TOXGO Kinesin motor domain-containing protein 594 208-213
7. LLSRVC V5B9R9 V5B9R9_TOXGO XPG N-terminal domain-containing protein 421 227-232
8. LSRVCL V4ZGN9 V4ZGN9_TOXGO Poly(ADP-ribose) polymerase catalytic domain protein 1907 1779-1784
9. SRVCLL B9PY21 B9PY21_TOXGO ACR-like protein 1530 204-209
10. SRVCLL V5AYS9 V5AYS9_TOXGO Uncharacterized protein 171 10-15
11. RVCLLL B9PYG5 B9PYG5_TOXGO DEAD/DEAH box helicase domain-containing protein 698 355-360
12. RVCLLL Q1JTF7 Q1JTF7_TOXGO ATP-dependent RNA helicase, putative 574 231-236
13. RVCLLL V4ZBN2 V4ZBN2_TOXGO Patched family protein 2498 1166-1171
14. RVCLLL V4ZMT3 V4ZMT3_TOXGO tRNA dimethylallyltransferase 933 19-24
15. RVCLLL V4ZSA4 V4ZSA4_TOXGO Putative crooked neck family 1 protein isoform 2 686 156-161
16. VCLLLP V4Z8Q6 V4Z8Q6_TOXGO Uncharacterized protein 1568 586-591
17. VCLLLP V4ZNB4 V4ZNB4_TOXGO Putative transmembrane protein 696 140-145
18. VCLLLP V4ZSA4 V4ZSA4_TOXGO Putative crooked neck family 1 protein isoform 2 686 157-162
19. CLLLPP B6KFK5 B6KFK5_TOXGO N-acetylglucosaminyl-phosphatidylinositol biosynthetic protein PigA, family GT4 protein 616 491-496
20. CLLLPP B9PY21 B9PY21_TOXGO ACR-like protein 1530 602-607
21. CLLLPP Q867V4 Q867V4_TOXGO Phosphatidylinositolglycan class A protein 616 491-496
22. CLLLPP V4ZEE5 V4ZEE5_TOXGO Uncharacterized protein 3039 2870-2875
23. CLLLPP V4ZHR6 V4ZHR6_TOXGO WD domain, G-beta repeat-containing protein 1581 1353-1358
24. LLLPPP B9Q6R3 B9Q6R3_TOXGO Uncharacterized protein 587 97-102
25. LLLPPP Q1JTJ7 Q1JTJ7_TOXGO Putative uncharacterized protein precursor 1839 1286-1291
26. LLLPPP Q6JD66 Q6JD66_TOXGO Eukaryotic initiation factor-2 alpha kinase-A 5072 2971-2976
27. LLLPPP V4YLB1 V4YLB1_TOXGO C2 domain-containing protein 1570 112-117
28. LLLPPP V4YZ84 V4YZ84_TOXGO Putative divalent metal transporter 1232 659-664
29. LLLPPP V4Z4M8 V4Z4M8_TOXGO Amine-terminal region of chorein, A TM vesicle-mediated sorter 13455 4229-4234
30. LLLPPP V4Z5T8 V4Z5T8_TOXGO Uncharacterized protein 3255 2919-2924
31. LLLPPP V4Z9X5 V4Z9X5_TOXGO Uncharacterized protein 4983 4237-4242
32. LLLPPP V4ZER7 V4ZER7_TOXGO Uncharacterized protein 3307 3177-3182
33. LLLPPP V4ZU83 V4ZU83_TOXGO eIF2 kinase IF2K-A (Incomplete catalytic triad) 4638 2537-2542
34. LLLPPP V5BHW3 V5BHW3_TOXGO PGAP1 family protein 2784 1945-1950
35. LLPPPC B9Q6R3 B9Q6R3_TOXGO Uncharacterized protein 587 98-103
36. LLPPPC V4Z4M8 V4Z4M8_TOXGO Amine-terminal region of chorein, A TM vesicle-mediated sorter 13455 4230-4235
37. LLPPPC V4Z933 V4Z933_TOXGO Type I inorganic pyrophosphatase PPase 491 52-57
38. LLPPPC V4ZJP8 V4ZJP8_TOXGO Amine-terminal region of chorein, A TM vesicle-mediated sorter 12207 1755-1760
39. PPPCAL V4YR59 V4YR59_TOXGO Uncharacterized protein 1342 183-188
40. PPPCAL V5BI59 V5BI59_TOXGO WD domain, G-beta repeat-containing protein 3142 1624-1629
41. ALVLAG B9PLM7 B9PLM7_TOXGO Dolichol-phosphate mannosyltransferase subunit 3 114 11-16
42. ALVLAG B9QPN0 B9QPN0_TOXGO Inorganic anion transporter, sulfate permease (SulP) family protein 1279 811-816
43. LVLAGV B9QNZ2 B9QNZ2_TOXGO Glycerophosphodiester phosphodiesterase family protein 866 471-476
44. LVLAGV Q1JSG3 Q1JSG3_TOXGO Uncharacterized protein precursor 1314 7-12
45. LVLAGV V4Z212 V4Z212_TOXGO Putative tryptophan-rich antigen, related protein 1362 7-12
46. LVLAGV V4Z7T4 V4Z7T4_TOXGO Elongation factor Tu GTP binding domain-containing protein 1331 930-935
47. AGVPSS A3FKJ8 A3FKJ8_TOXGO PMCA-type calcium ATPase A2 1200 640-645
48. AGVPSS B9PUT1 B9PUT1_TOXGO Uncharacterized protein 336 187-192
49. AGVPSS V4YND4 V4YND4_TOXGO eIF2 kinase IF2K-D (Incomplete catalytic triad) 2866 1330-1335
50. AGVPSS V4ZE20 V4ZE20_TOXGO AP2 domain transcription factor AP2V-2 3456 1954-1959
51. AGVPSS V4ZL97 V4ZL97_TOXGO Uncharacterized protein 7954 6641-6646
52. AGVPSS V4ZS19 V4ZS19_TOXGO Uncharacterized protein 1139 79-84
53. AGVPSS V5BLZ2 V5BLZ2_TOXGO Calcium-translocating P-type ATPase, PMCA-type protein 1448 773-778
54. GVPSSS B6KB45 B6KB45_TOXGO Actin like protein ALP2a 568 36-41
55. GVPSSS B6KRI3 B6KRI3_TOXGO Carrier superfamily protein 885 386-391
56. GVPSSS B9PUT1 B9PUT1_TOXGO Uncharacterized protein 336 188-193
57. GVPSSS B9QML2 B9QML2_TOXGO Myosin F 1953 154-159
58. GVPSSS Q286V9 Q286V9_TOXGO Myosin F 1953 154-159
59. GVPSSS V4Z6C0 V4Z6C0_TOXGO LsmAD domain-containing protein 1524 300-305
60. GVPSSS V4ZE20 V4ZE20_TOXGO AP2 domain transcription factor AP2V-2 3456 1955-1960
61. GVPSSS V4ZMZ5 V4ZMZ5_TOXGO Uncharacterized protein 643 175-180
62. GVPSSS V4ZPH3 V4ZPH3_TOXGO Protein kinase domain protein 2329 1138-1143
63. VPSSSS B9QAM8 B9QAM8_TOXGO Uncharacterized protein 2194 446-451
64. VPSSSS B9QDV9 B9QDV9_TOXGO Spc97/Spc98 family protein 1981 1319-1324
65. VPSSSS B9QFP6 B9QFP6_TOXGO Rad17 cell cycle checkpoint protein 1867 1071-1076
66. VPSSSS B9QH19 B9QH19_TOXGO Putative transmembrane protein 1423 894-899
67. VPSSSS B9QL14 B9QL14_TOXGO Inositol polyphosphate kinase 2851 300-305
68. VPSSSS B9QLX5 B9QLX5_TOXGO Carrier superfamily protein 716 41-46
69. VPSSSS B9QML2 B9QML2_TOXGO Myosin F 1953 155-160
70. VPSSSS B9QR35 B9QR35_TOXGO Zinc finger, C3HC4 type (RING finger) domain-containing protein 710 599-604
71. VPSSSS H3K408 H3K408_TOXGO CaMK-related kinase 3196 789-794
72. VPSSSS Q1JT54 Q1JT54_TOXGO Putative uncharacterized protein 1990 1733-1738
73. VPSSSS Q286V9 Q286V9_TOXGO Myosin F 1953 155-160
74. VPSSSS V4YMZ6 V4YMZ6_TOXGO Putative origin recognition complex subunit 878 98-103
75. VPSSSS V4YNY2 V4YNY2_TOXGO Uncharacterized protein 760 97-102
76. VPSSSS V4YU10 V4YU10_TOXGO Breast carcinoma amplified sequence protein 2 359 316-321
77. VPSSSS V4Z3A5 V4Z3A5_TOXGO Uncharacterized protein 1525 790-795, 848-853
78. VPSSSS V4Z3M6 V4Z3M6_TOXGO Uncharacterized protein 2856 1920-1925
79. VPSSSS V4Z474 V4Z474_TOXGO Uncharacterized protein 1469 115-120
80. VPSSSS V4Z4F4 V4Z4F4_TOXGO Uncharacterized protein 3102 1934-1939, 2803-2808
81. VPSSSS V4Z5P5 V4Z5P5_TOXGO Uncharacterized protein 5771 3508-3513
82. VPSSSS V4Z8D8 V4Z8D8_TOXGO Putative zinc carboxypeptidase 2203 445-450
83. VPSSSS V4Z9Y8 V4Z9Y8_TOXGO Uncharacterized protein 2154 1733-1738
84. VPSSSS V4ZDV4 V4ZDV4_TOXGO HEAT repeat-containing protein 2373 2174-2179
85. VPSSSS V4ZDY2 V4ZDY2_TOXGO Ribonuclease type III Dicer 4343 2572-2577
86. VPSSSS V4ZH09 V4ZH09_TOXGO Uncharacterized protein 325 132-137
87. VPSSSS V4ZKD8 V4ZKD8_TOXGO Uncharacterized protein 11926 1186-1191
88. VPSSSS V4ZMZ5 V4ZMZ5_TOXGO Uncharacterized protein 643 176-181
89. VPSSSS V4ZQY3 V4ZQY3_TOXGO TBC domain-containing kinase (Incomplete catalytic triad) 1887 1033-1038
90. VPSSSS V4ZVZ1 V4ZVZ1_TOXGO Uncharacterized protein 2363 784-789
91. VPSSSS V5AZE6 V5AZE6_TOXGO Uncharacterized protein 2045 1003-1008
92. VPSSSS V5B884 V5B884_TOXGO SWI2/SNF2-containing protein RAD26 1590 1095-1100
93. VPSSSS V5BF72 V5BF72_TOXGO WD domain, G-beta repeat-containing protein 1605 801-806
94. VPSSSS V5BJK8 V5BJK8_TOXGO CAM kinase, SNF1 family 3071 789-794
95. PSSSSH B9QFM9 B9QFM9_TOXGO Anaphase-promoting complex subunit APC10 601 160-165
96. PSSSSH B9QG15 B9QG15_TOXGO Aspartyl protease ASP1 619 59-64
97. PSSSSH B9QR35 B9QR35_TOXGO Zinc finger, C3HC4 type (RING finger) domain-containing protein 710 600-605
98. PSSSSH Q6PTV2 Q6PTV2_TOXGO Toxomepsin 1 620 59-64
99. PSSSSH V4YTH9 V4YTH9_TOXGO DEAD/DEAH box helicase domain-containing protein 4426 834-839
100. PSSSSH V4Z3Y3 V4Z3Y3_TOXGO Uncharacterized protein 1915 968-973
101. PSSSSH V4Z5U2 V4Z5U2_TOXGO TBC domain-containing protein 2711 1970-1975
102. PSSSSH V4Z866 V4Z866_TOXGO Protein kinase domain-containing protein 1901 546-551
103. PSSSSH V4Z8B0 V4Z8B0_TOXGO Uncharacterized protein 1838 1332-1337
104. PSSSSH V4ZS91 V4ZS91_TOXGO Inositol polyphosphate kinase 1573 226-231
105. PSSSSH V5B2E0 V5B2E0_TOXGO MORN repeat-containing protein 3443 273-278
106. PSSSSH V5BBA8 V5BBA8_TOXGO Hydrolase CocE/NonD family protein 1260 805-810
107. PSSSSH V5BBQ8 V5BBQ8_TOXGO Leucine rich repeat-containing protein 4458 4109-4114
108. PSSSSH V5BDM3 V5BDM3_TOXGO Sec7 domain-containing protein 3987 1611-1616
109. SSSSHP B9Q7Y3 B9Q7Y3_TOXGO Transmembrane amino acid transporter protein 726 50-55
110. SSSSHP B9QEV3 B9QEV3_TOXGO Zinc finger, C3HC4 type (RING finger) domain-containing protein 3872 241-246
111. SSSSHP B9QH18 B9QH18_TOXGO tRNA ligases class I (M) protein 1768 361-366
112. SSSSHP Q1JT79 Q1JT79_TOXGO Putative uncharacterized protein precursor 1342 721-726
113. SSSSHP Q1JTB6 Q1JTB6_TOXGO Methyl transferase, putative 842 537-542
114. SSSSHP V4YI12 V4YI12_TOXGO Thrombospondin type 1 domain-containing protein 1218 356-361
115. SSSSHP V4Z0B6 V4Z0B6_TOXGO Tetratricopeptide repeat-containing protein 1383 514-519
116. SSSSHP V4Z1C2 V4Z1C2_TOXGO Uncharacterized protein 2290 562-567
117. SSSSHP V4Z2U4 V4Z2U4_TOXGO Uncharacterized protein 1386 1025-1030
118. SSSSHP V4Z445 V4Z445_TOXGO Histone acetyltransferase TAF1/250 2775 279-284
119. SSSSHP V4Z7V2 V4Z7V2_TOXGO Putative transmembrane protein 371 47-52
120. SSSSHP V4ZBN7 V4ZBN7_TOXGO Uncharacterized protein 2456 2043-2048
121. SSSSHP V4ZE07 V4ZE07_TOXGO Ubiquitin fusion degradation protein UFD1AP 749 150-155
122. SSSSHP V4ZIG9 V4ZIG9_TOXGO Putative transmembrane protein 1334 540-545
123. SSSSHP V4ZK39 V4ZK39_TOXGO Putative transmembrane protein 764 491-496
124. SSSSHP V4ZNK7 V4ZNK7_TOXGO Uncharacterized protein 188 47-52
125. SSSSHP V5B055 V5B055_TOXGO NOL1/NOP2/sun family protein 970 567-572
126. SSSHPQ V4Z7V2 V4Z7V2_TOXGO Putative transmembrane protein 371 48-53
127. SSHPQP B6K9M4 B6K9M4_TOXGO GYF domain protein 825 31-36
128. QILKRI B9PYC5 B9PYC5_TOXGO Ribonucleoside-diphosphate reductase 877 76-81
129. QILKRI Q1JTC5 Q1JTC5_TOXGO Ribonucleoside-diphosphate reductase 855 54-59
130. LKRIGH B9PHP5 B9PHP5_TOXGO Synaptobrevin family protein 225 40-45
131. VRVGAV V4ZD79 V4ZD79_TOXGO Utp14 1028 986-991
132. RVGAVH V4ZPK7 V4ZPK7_TOXGO Sec20 protein 808 257-262
133. TAPRAA V4ZPL2 V4ZPL2_TOXGO Uncharacterized protein 1352 210-215
134. APRAAS B6KU21 B6KU21_TOXGO Uncharacterized protein 1566 847-852
135. APRAAS V4ZAJ4 V4ZAJ4_TOXGO Histone lysine demethylase JMJD5 1087 137-142
136. APRAAS V5AY44 V5AY44_TOXGO Putative transmembrane protein 7450 2720-2725
137. APRAAS V5BDD5 V5BDD5_TOXGO CPSF A subunit region protein 2077 433-438
138. PRAASR B9Q5H6 B9Q5H6_TOXGO MIF4G domain-containing protein 2668 1732-1737
139. PRAASR B9QNI0 B9QNI0_TOXGO Riboflavin kinase 1146 385-390
140. PRAASR C7EUB5 C7EUB5_TOXGO MAR domain containing protein 4 1016 550-555
141. PRAASR Q1JSP2 Q1JSP2_TOXGO Uncharacterized protein precursor 926 544-549
142. PRAASR V5B0P5 V5B0P5_TOXGO Putative microneme protein 999 533-538
143. PRAASR V5BGS1 V5BGS1_TOXGO Zinc finger (CCCH type) motif-containing protein 3460 3233-3238
144. PRAASR V5BIM4 V5BIM4_TOXGO Uncharacterized protein 1813 1617-1622
145. RAASRA B6KHD0 B6KHD0_TOXGO AP2 domain transcription factor AP2XII-6 279 101-106
146. RAASRA B6KJN2 B6KJN2_TOXGO Putative tola protein 946 91-96
147. RAASRA B6KR18 B6KR18_TOXGO Putative cell-cycle-associated protein kinase CDK 1372 431-436
148. RAASRA Q68J50 Q68J50_TOXGO Nucleolar G-protein NOG1 719 636-641
149. RAASRA V4ZE30 V4ZE30_TOXGO Putative nucleolar GTP-binding protein 1 719 636-641
150. RAASRA V4ZL10 V4ZL10_TOXGO Putative transmembrane protein 2026 1637-1642
151. RAASRA V4ZQC7 V4ZQC7_TOXGO Putative transmembrane protein 1718 1332-1337
152. AASRAP B9QB74 B9QB74_TOXGO NOL1/NOP2/Sun family protein 919 410-415
153. AASRAP B9QMB6 B9QMB6_TOXGO AP2 domain transcription factor AP2IX-8 1753 185-190
154. AASRAP Q1JSL3 Q1JSL3_TOXGO mRNA decapping enzyme, putative precursor 512 160-165
155. AASRAP V4YMB2 V4YMB2_TOXGO Putative mRNA decapping enzyme 400 48-53
156. AASRAP V5BAM1 V5BAM1_TOXGO Uncharacterized protein 957 273-278
157. ASRAPD V4YYU7 V4YYU7_TOXGO Putative glyoxalase 4813 2376-2381
158. ASRAPD V4ZLJ6 V4ZLJ6_TOXGO MIZ/SP-RING zinc finger domain-containing protein 1779 765-770
159. APDDSR B9QFY1 B9QFY1_TOXGO Inosine triphosphate pyrophosphatase 222 13-18
160. PDDSRA V4Z7R5 V4Z7R5_TOXGO Uncharacterized protein 372 326-331
161. DSRAGA V4YNU1 V4YNU1_TOXGO Putative transmembrane protein 3158 1488-1493
162. DSRAGA V4ZVU8 V4ZVU8_TOXGO Uncharacterized protein 4014 876-881
163. SRAGAQ V4Z2X0 V4Z2X0_TOXGO WD domain, G-beta repeat-containing protein 552 270-275
164. RAGAQR V4ZEA3 V4ZEA3_TOXGO Putative transmembrane protein 1292 1159-1164
165. AQRDEP V5B4Q9 V5B4Q9_TOXGO WD domain, G-beta repeat-containing protein 3086 1519-1524
166. QRDEPE V5B4Q9 V5B4Q9_TOXGO WD domain, G-beta repeat-containing protein 3086 1520-1525
167. PGTRRS B6KFU8 B6KFU8_TOXGO Putative transmembrane protein 633 85-90
168. GTRRSP B9QPU3 B9QPU3_TOXGO Putative RNase protein H 1344 1229-1234
169. GTRRSP V4YVM3 V4YVM3_TOXGO Uncharacterized protein 2336 476-481
170. TRRSPA B6KH80 B6KH80_TOXGO Uncharacterized protein 236 28-33
171. TRRSPA V4Z9J2 V4Z9J2_TOXGO Uncharacterized protein 1923 1784-1789
172. RRSPAP B6K8P4 B6K8P4_TOXGO Uncharacterized protein 3213 2975-2980
173. RRSPAP Q6JD66 Q6JD66_TOXGO Eukaryotic initiation factor-2 alpha kinase-A 5072 3376-3381
174. RRSPAP V4ZU83 V4ZU83_TOXGO eIF2 kinase IF2K-A (Incomplete catalytic triad) 4638 2942-2947
175. RSPAPS B6KVJ2 B6KVJ2_TOXGO Uncharacterized protein 475 462-467
176. RSPAPS B9QQ39 B9QQ39_TOXGO KRUF family protein 1023 841-846
177. RSPAPS Q1JTH2 Q1JTH2_TOXGO Putative uncharacterized protein precursor 1321 713-718
178. RSPAPS V4YLD5 V4YLD5_TOXGO KRUF family protein 367 188-193
179. RSPAPS V4Z405 V4Z405_TOXGO Uncharacterized protein 140 70-75
180. RSPAPS V4Z4B4 V4Z4B4_TOXGO Sec7 domain-containing protein 3015 753-758
181. RSPAPS V4ZMY1 V4ZMY1_TOXGO Flagellar/basal body protein 2758 2302-2307
182. SPAPSP A3FKJ8 A3FKJ8_TOXGO PMCA-type calcium ATPase A2 1200 173-178
183. SPAPSP B6KJY3 B6KJY3_TOXGO Uncharacterized protein 1537 83-88
184. SPAPSP B9PMH5 B9PMH5_TOXGO Putative mitochondrial inner membrane translocase subunit TIM17 229 19-24
185. SPAPSP B9Q700 B9Q700_TOXGO Nuclear fragile X mental retardation-interacting protein 1 830 182-187
186. SPAPSP B9Q8I2 B9Q8I2_TOXGO Uncharacterized protein 2497 1766-1771
187. SPAPSP B9QNV2 B9QNV2_TOXGO ABC transporter transmembrane region domain-containing protein 1005 318-323
188. SPAPSP B9QR17 B9QR17_TOXGO Uncharacterized protein 6038 4342-4347
189. SPAPSP Q1JSV3 Q1JSV3_TOXGO Uncharacterized protein 116 84-89
190. SPAPSP V4Z2Q0 V4Z2Q0_TOXGO Uncharacterized protein 1684 142-147
191. SPAPSP V4Z8N2 V4Z8N2_TOXGO Uncharacterized protein 264 183-188
192. SPAPSP V4ZAB1 V4ZAB1_TOXGO GCC2 and GCC3 domain-containing protein 2914 1989-1994
193. SPAPSP V4ZEE0 V4ZEE0_TOXGO Putative transmembrane protein 933 365-370
194. SPAPSP V4ZEN8 V4ZEN8_TOXGO Uncharacterized protein 4618 4348-4353
195. SPAPSP V4ZJ96 V4ZJ96_TOXGO Uncharacterized protein 1123 271-276
196. SPAPSP V4ZV93 V4ZV93_TOXGO Uncharacterized protein 4618 325-330
197. SPAPSP V5AXK1 V5AXK1_TOXGO FG-GAP repeat-containing protein 1678 1195-1200
198. SPAPSP V5BDT5 V5BDT5_TOXGO Phosphatidylinositol 3-and 4-kinase 8859 4909-4914
199. SPAPSP V5BLZ2 V5BLZ2_TOXGO Calcium-translocating P-type ATPase, PMCA-type protein 1448 306-311
200. PAPSPG B6KHN4 B6KHN4_TOXGO tRNA (Uracil-5-)-methyltransferase 1116 639-644
201. PAPSPG B6KU19 B6KU19_TOXGO Uncharacterized protein 156 109-114
202. PAPSPG V4ZDL1 V4ZDL1_TOXGO Uncharacterized protein 306 287-292
203. PAPSPG V4ZF76 V4ZF76_TOXGO Uncharacterized protein 1816 68-73
204. PAPSPG V5BLE2 V5BLE2_TOXGO 3'5'-cyclic nucleotide phosphodiesterase domain-containing protein 1294 209-214
205. APSPGA B9Q792 B9Q792_TOXGO Uncharacterized protein 4118 2674-2679
206. APSPGA V4ZPY4 V4ZPY4_TOXGO RAVE 1 carboxy-terminal protein 6665 5699-5704
207. LGSTLH V4Z040 V4Z040_TOXGO Putative cation-transporting ATPase 3425 1801-1806
208. LGSTLH V4ZU57 V4ZU57_TOXGO Mediator complex subunit MED14 4355 3791-3796
209. STLHGR V4ZMJ1 V4ZMJ1_TOXGO Uncharacterized protein 4969 2706-2711
210. RGPPGS B9QAV2 B9QAV2_TOXGO Hydrolase, alpha/beta fold family protein 844 169-174
211. RGPPGS V4YQ73 V4YQ73_TOXGO MIF4G domain-containing protein 3756 536-541
212. RGPPGS V5BJC6 V5BJC6_TOXGO EF hand domain-containing protein 6368 5009-5014
213. GPPGSR Q1JSJ1 Q1JSJ1_TOXGO Uncharacterized protein 3577 918-923
214. GPPGSR V4Z251 V4Z251_TOXGO Uncharacterized protein 3633 918-923
215. GPPGSR V4ZIA7 V4ZIA7_TOXGO Uncharacterized protein 814 492-497
216. GPPGSR V5BJC6 V5BJC6_TOXGO EF hand domain-containing protein 6368 5010-5015
217. PPGSRK V5BIT9 V5BIT9_TOXGO Uncharacterized protein 2397 2329-2334
218. KPGEGA V5BLF5 V5BLF5_TOXGO Uncharacterized protein 703 43-48
219. PGEGAR V4Z4M8 V4Z4M8_TOXGO Amine-terminal region of chorein, A TM vesicle-mediated sorter 13455 3371-3376
220. GEGARA V4YSC6 V4YSC6_TOXGO Uncharacterized protein 1572 749-754
221. GEGARA V4ZCD6 V4ZCD6_TOXGO Putative transmembrane protein 628 532-537
222. GEGARA V4ZMD1 V4ZMD1_TOXGO Putative transmembrane protein 431 412-417
223. EGARAE B6K8I5 B6K8I5_TOXGO SURF1 family protein 685 272-277
224. EGARAE B6K973 B6K973_TOXGO Zinc finger, C3HC4 type (RING finger) domain-containing protein 2190 569-574
225. EGARAE V4ZCD6 V4ZCD6_TOXGO Putative transmembrane protein 628 533-538
226. GARAEA B6K8I5 B6K8I5_TOXGO SURF1 family protein 685 273-278
227. GARAEA V4YUP1 V4YUP1_TOXGO Uncharacterized protein 1171 1120-1125
228. GARAEA V4Z3J3 V4Z3J3_TOXGO Uncharacterized protein 1357 276-281
229. GARAEA V4ZBJ2 V4ZBJ2_TOXGO Tyrosine kinase-like (TKL) protein 1673 189-194
230. GARAEA V4ZGI1 V4ZGI1_TOXGO Calcium-dependent protein kinase 2228 2056-2061
231. ARAEAL B6KRB8 B6KRB8_TOXGO DNA-directed RNA polymerase III RPC1 1746 588-593
232. ARAEAL B9PQJ8 B9PQJ8_TOXGO Uncharacterized protein 1162 936-941
233. ARAEAL B9QKB8 B9QKB8_TOXGO Uncharacterized protein 2225 1762-1767
234. WPRDAL V4YXU2 V4YXU2_TOXGO Dynein heavy chain family protein 4551 3027-3032
235. PRDALL Q1JSJ8 Q1JSJ8_TOXGO Uncharacterized protein 2342 592-597
236. PRDALL V4Z766 V4Z766_TOXGO Uncharacterized protein 1905 1687-1692
237. PRDALL V5B0W4 V5B0W4_TOXGO Uncharacterized protein 1806 592-597
238. DALLFA V4YPB3 V4YPB3_TOXGO Uncharacterized protein 1552 1022-1027
239. LLFAVD V4ZVZ1 V4ZVZ1_TOXGO Uncharacterized protein 2363 1293-1298
240. FAVDNL V4Z7B8 V4Z7B8_TOXGO Alpha/beta hydrolase family protein 3457 3449-3454
241. NRVEGL V4ZPH7 V4ZPH7_TOXGO Putative transmembrane protein 2525 1660-1665
242. RVEGLL V5BG52 V5BG52_TOXGO Peroxisomal membrane anchor 1019 425-430
243. RVEGLL V5BKN7 V5BKN7_TOXGO Putative GCN1 3416 3091-3096
244. VEGLLP B6K9F0 B6K9F0_TOXGO Ubiquitin carboxyl-terminal hydrolase 1697 797-802
245. VEGLLP Q1JT54 Q1JT54_TOXGO Putative uncharacterized protein 1990 1422-1427
246. VEGLLP V4Z2E9 V4Z2E9_TOXGO AP2 domain transcription factor AP2IV-5 1966 1612-1617
247. VEGLLP V4Z9Y8 V4Z9Y8_TOXGO Uncharacterized protein 2154 1422-1427
248. EGLLPY V4Z3T0 V4Z3T0_TOXGO Aldehyde dehydrogenase 497 470-475
249. LSLEVV V4ZQG6 V4ZQG6_TOXGO Putative transmembrane protein 988 834-839
250. AIEAGL B6KJX9 B6KJX9_TOXGO V-type proton ATPase subunit a 909 750-755
251. AGLGDL B9QAQ9 B9QAQ9_TOXGO Putative transmembrane protein 637 322-327
252. AGLGDL V4ZCD0 V4ZCD0_TOXGO Putative trichohyalin 497 127-132
253. AGLGDL V4ZJH2 V4ZJH2_TOXGO RNA recognition motif-containing protein 2070 315-320
254. AGLGDL V5BH73 V5BH73_TOXGO Elongation factor Tu GTP binding domain-containing protein 2560 2167-2172
255. GLGDLP V4ZI01 V4ZI01_TOXGO Uncharacterized protein 532 467-472
256. GDLPLL B9QLY4 B9QLY4_TOXGO RNA methyltransferase, TrmH family protein 2898 1676-1681
257. GDLPLL V4YNM0 V4YNM0_TOXGO Uncharacterized protein 2518 2108-2113
258. GDLPLL V4Z6U5 V4Z6U5_TOXGO Uncharacterized protein 1269 860-865
259. LPLLPF B6KJ47 B6KJ47_TOXGO Uncharacterized protein 3460 2103-2108
260. LPLLPF B6KRA7 B6KRA7_TOXGO ATP-dependent DNA helicase, RecQ family protein 1626 192-197
261. LPLLPF V4YNU1 V4YNU1_TOXGO Putative transmembrane protein 3158 1433-1438
262. LPLLPF V4YQT3 V4YQT3_TOXGO Putative transmembrane protein 3329 1684-1689
263. LPLLPF V4Z7R9 V4Z7R9_TOXGO Uncharacterized protein 1083 374-379
264. LPLLPF V4ZBS8 V4ZBS8_TOXGO ABC transporter transmembrane region domain-containing protein 1951 55-60
265. LPLLPF V4ZFK2 V4ZFK2_TOXGO SWI2/SNF2-containing protein RAD16 1700 295-300
266. LPLLPF V4ZNF8 V4ZNF8_TOXGO Uncharacterized protein 1765 516-521
267. LPLLPF V4ZNQ0 V4ZNQ0_TOXGO Uncharacterized protein 5025 178-183
268. PLLPFS B6KF87 B6KF87_TOXGO Pescadillo homolog 733 476-481
269. PLLPFS B6KJ47 B6KJ47_TOXGO Uncharacterized protein 3460 2104-2109
270. PLLPFS B6KRA7 B6KRA7_TOXGO ATP-dependent DNA helicase, RecQ family protein 1626 193-198
271. PLLPFS Q1JTG1 Q1JTG1_TOXGO tRNA ligase class II core domain (G, H, P, S and T) domain-containing protein precursor 1480 61-66
272. PLLPFS V4YU57 V4YU57_TOXGO Adenosine-deaminase domain protein 1494 1017-1022
273. PLLPFS V4ZJD6 V4ZJD6_TOXGO Putative cell-cycle-associated protein kinase CLK 1699 150-155
274. LLPFSS B6K8J1 B6K8J1_TOXGO Putative transmembrane protein 4690 3602-3607
275. LLPFSS V4ZB68 V4ZB68_TOXGO Putative transmembrane protein 208 13-18
276. LLPFSS V4ZJD6 V4ZJD6_TOXGO Putative cell-cycle-associated protein kinase CLK 1699 151-156
277. LLPFSS V5BA46 V5BA46_TOXGO Uncharacterized protein 2092 1269-1274
278. LPFSSP B6K9V2 B6K9V2_TOXGO WD domain, G-beta repeat-containing protein 1218 550-555
279. LPFSSP B9QQF1 B9QQF1_TOXGO Proteasome/cyclosome repeat-containing protein 3187 665-670
280. LPFSSP V4YWT0 V4YWT0_TOXGO Uncharacterized protein 1747 941-946
281. LPFSSP V4YZ55 V4YZ55_TOXGO DNA repair protein Rad4 domain-containing protein 1935 591-596
282. LPFSSP V4Z8P2 V4Z8P2_TOXGO Endonuclease/exonuclease/phosphatase family protein 1111 143-148
283. LPFSSP V4ZFZ6 V4ZFZ6_TOXGO Ribosomal protein RPS6 993 755-760
284. LPFSSP V4ZVI6 V4ZVI6_TOXGO SufB/sufD domain-containing protein 1860 1545-1550
285. LPFSSP V5BBQ8 V5BBQ8_TOXGO Leucine rich repeat-containing protein 4458 3136-3141
286. PFSSPS B6KRJ1 B6KRJ1_TOXGO Uncharacterized protein 2272 1627-1632
287. PFSSPS B9Q0M8 B9Q0M8_TOXGO Leucine zipper-like transcriptional regulator 855 327-332
288. PFSSPS B9Q6W0 B9Q6W0_TOXGO ThiF family protein 1073 57-62
289. PFSSPS B9QCL9 B9QCL9_TOXGO CAAX amino terminal protease family protein 1263 665-670
290. PFSSPS B9QFM9 B9QFM9_TOXGO Anaphase-promoting complex subunit APC10 601 173-178
291. PFSSPS B9QFZ7 B9QFZ7_TOXGO Enoyl-CoA hydratase/isomerase family protein 602 6-11
292. PFSSPS B9QPW3 B9QPW3_TOXGO Uncharacterized protein 596 512-517
293. PFSSPS B9QQF1 B9QQF1_TOXGO Proteasome/cyclosome repeat-containing protein 3187 666-671
294. PFSSPS Q1JT79 Q1JT79_TOXGO Putative uncharacterized protein precursor 1342 764-769
295. PFSSPS Q3S2X2 Q3S2X2_TOXGO Leucine zipper-like transcriptional regulator 855 327-332
296. PFSSPS V4YNU1 V4YNU1_TOXGO Putative transmembrane protein 3158 2666-2671
297. PFSSPS V4YTU2 V4YTU2_TOXGO Protamine P1 protein 2719 662-667
298. PFSSPS V4YZ55 V4YZ55_TOXGO DNA repair protein Rad4 domain-containing protein 1935 592-597
299. PFSSPS V4YZ80 V4YZ80_TOXGO HECT-domain (Ubiquitin-transferase) domain-containing protein 15897 2613-2618
300. PFSSPS V4Z1C2 V4Z1C2_TOXGO Uncharacterized protein 2290 605-610
301. PFSSPS V4Z2T5 V4Z2T5_TOXGO Putative SNARE domain protein 397 170-175
302. PFSSPS V4Z2Y1 V4Z2Y1_TOXGO Uncharacterized protein 3246 2238-2243
303. PFSSPS V4Z5Y9 V4Z5Y9_TOXGO Putative transmembrane protein 635 320-325
304. PFSSPS V4Z8A5 V4Z8A5_TOXGO Putative trigger factor protein 1031 46-51
305. PFSSPS V4Z8P2 V4Z8P2_TOXGO Endonuclease/exonuclease/phosphatase family protein 1111 144-149
306. PFSSPS V4Z8T5 V4Z8T5_TOXGO Uncharacterized protein 3210 1895-1900
307. PFSSPS V4ZE20 V4ZE20_TOXGO AP2 domain transcription factor AP2V-2 3456 2199-2204
308. PFSSPS V4ZGB0 V4ZGB0_TOXGO Zinc finger (CCCH type) motif-containing protein 1513 237-242
309. PFSSPS V4ZGQ4 V4ZGQ4_TOXGO Uncharacterized protein 2272 2043-2048
310. PFSSPS V4ZI48 V4ZI48_TOXGO Uncharacterized protein 924 191-196
311. FSSPSS B6KDU6 B6KDU6_TOXGO Uncharacterized protein 1366 518-523
312. FSSPSS B6KGN5 B6KGN5_TOXGO Large subunit ribosomal protein IMG2 310 51-56
313. FSSPSS B6KGN7 B6KGN7_TOXGO SprT domain-containing protein 839 186-191
314. FSSPSS B6KMV3 B6KMV3_TOXGO S1/P1 nuclease 632 225-230
315. FSSPSS B6KRJ1 B6KRJ1_TOXGO Uncharacterized protein 2272 1628-1633
316. FSSPSS B9Q0M8 B9Q0M8_TOXGO Leucine zipper-like transcriptional regulator 855 328-333
317. FSSPSS B9Q504 B9Q504_TOXGO Uncharacterized protein 4030 1268-1273
318. FSSPSS B9Q6G0 B9Q6G0_TOXGO Pyruvate dehydrogenase complex subunit PD-HE1Beta 470 77-82
319. FSSPSS B9Q8B0 B9Q8B0_TOXGO Uncharacterized protein 2014 163-168
320. FSSPSS B9Q8F9 B9Q8F9_TOXGO Putative elongation factor Tu GTP-binding domain protein 1766 1647-1652
321. FSSPSS B9QCL9 B9QCL9_TOXGO CAAX amino terminal protease family protein 1263 666-671
322. FSSPSS B9QEM9 B9QEM9_TOXGO Putative ribonuclease ZC3H12D 1968 1165-1170
323. FSSPSS B9QHR3 B9QHR3_TOXGO Putative armadillo/beta-catenin-like repeat protein 1297 68-73
324. FSSPSS B9QJT5 B9QJT5_TOXGO WLM domain protein 1333 1065-1070
325. FSSPSS B9QQF5 B9QQF5_TOXGO Uncharacterized protein 976 323-328
326. FSSPSS Q1JSM3 Q1JSM3_TOXGO Uncharacterized protein 701 248-253
327. FSSPSS Q1JT79 Q1JT79_TOXGO Putative uncharacterized protein precursor 1342 765-770
328. FSSPSS Q1JTE7 Q1JTE7_TOXGO Putative uncharacterized protein precursor 651 310-315
329. FSSPSS Q1KSE7 Q1KSE7_TOXGO Phosphoglycerate kinase 551 31-36
330. FSSPSS Q1KSF0 Q1KSF0_TOXGO Apicoplast pyruvate dehydrogenase E1 beta subunit 470 77-82
331. FSSPSS Q3S2X2 Q3S2X2_TOXGO Leucine zipper-like transcriptional regulator 855 328-333
332. FSSPSS V4YI17 V4YI17_TOXGO Lipase 1130 470-475
333. FSSPSS V4YLH6 V4YLH6_TOXGO Uncharacterized protein 3421 3049-3054, 3056-3061
334. FSSPSS V4YNU1 V4YNU1_TOXGO Putative transmembrane protein 3158 2667-2672
335. FSSPSS V4YS05 V4YS05_TOXGO Putative transmembrane protein 2088 1026-1031
336. FSSPSS V4YV42 V4YV42_TOXGO Elongation factor G, mitochondrial 878 42-47
337. FSSPSS V4Z1C2 V4Z1C2_TOXGO Uncharacterized protein 2290 606-611
338. FSSPSS V4Z2F7 V4Z2F7_TOXGO Putative transmembrane protein 2862 117-122
339. FSSPSS V4Z3I2 V4Z3I2_TOXGO SCY kinase-related protein (Incomplete catalytic triad) 1065 928-933
340. FSSPSS V4Z3K3 V4Z3K3_TOXGO PIK3R4 kinase-related protein (Incomplete catalytic triad) 3028 2419-2424
341. FSSPSS V4Z3N7 V4Z3N7_TOXGO Alpha/beta hydrolase family protein 1235 260-265
342. FSSPSS V4Z3T6 V4Z3T6_TOXGO Uncharacterized protein 1806 156-161
343. FSSPSS V4Z550 V4Z550_TOXGO Uncharacterized protein 1008 113-118, 454-459
344. FSSPSS V4Z5U2 V4Z5U2_TOXGO TBC domain-containing protein 2711 1978-1983
345. FSSPSS V4Z6R4 V4Z6R4_TOXGO Uncharacterized protein 1724 1003-1008
346. FSSPSS V4Z6T1 V4Z6T1_TOXGO Dopey, N-terminal domain-containing protein 3103 1716-1721
347. FSSPSS V4Z754 V4Z754_TOXGO AAA domain protein 2605 634-639
348. FSSPSS V4Z7F3 V4Z7F3_TOXGO TBC domain-containing protein 2116 573-578
349. FSSPSS V4Z7N4 V4Z7N4_TOXGO Uncharacterized protein 2114 861-866
350. FSSPSS V4Z7P3 V4Z7P3_TOXGO Uncharacterized protein 1860 191-196
351. FSSPSS V4Z818 V4Z818_TOXGO Uncharacterized protein 639 204-209
352. FSSPSS V4Z842 V4Z842_TOXGO DUSP domain protein 1154 451-456
353. FSSPSS V4Z8G0 V4Z8G0_TOXGO Guanylyl cyclase 4367 553-558
354. FSSPSS V4Z8T5 V4Z8T5_TOXGO Uncharacterized protein 3210 1896-1901
355. FSSPSS V4Z967 V4Z967_TOXGO Uncharacterized protein 2849 527-532
356. FSSPSS V4Z9U0 V4Z9U0_TOXGO Uncharacterized protein 5083 2648-2653
357. FSSPSS V4Z9U8 V4Z9U8_TOXGO Uncharacterized protein 630 310-315
358. FSSPSS V4ZAA2 V4ZAA2_TOXGO Uncharacterized protein 2719 1236-1241
359. FSSPSS V4ZB80 V4ZB80_TOXGO Phosphatidylinositol 3-and 4-kinase 2983 702-707
360. FSSPSS V4ZBM9 V4ZBM9_TOXGO Uncharacterized protein 2643 46-51
361. FSSPSS V4ZCB0 V4ZCB0_TOXGO Histone lysine-specific demethylase 3802 1245-1250
362. FSSPSS V4ZCI3 V4ZCI3_TOXGO Phosphoglycerate kinase 593 73-78
363. FSSPSS V4ZDU9 V4ZDU9_TOXGO Putative transmembrane protein 3661 58-63
364. FSSPSS V4ZF86 V4ZF86_TOXGO Putative transmembrane protein 476 35-40
365. FSSPSS V4ZFD5 V4ZFD5_TOXGO AP2 domain transcription factor AP2IX-7 2672 1713-1718
366. FSSPSS V4ZFS7 V4ZFS7_TOXGO Zinc finger, C3HC4 type (RING finger) domain-containing protein 1500 1010-1015
367. FSSPSS V4ZI48 V4ZI48_TOXGO Uncharacterized protein 924 123-128
368. FSSPSS V4ZKZ1 V4ZKZ1_TOXGO Uncharacterized protein 1477 1032-1037
369. FSSPSS V4ZL18 V4ZL18_TOXGO ATP-binding domain-containing protein 1917 766-771
370. FSSPSS V4ZSL1 V4ZSL1_TOXGO Putative transmembrane protein 1892 247-252
371. FSSPSS V5AYL1 V5AYL1_TOXGO Putative phosphoserine aminotransferase 481 198-203
372. FSSPSS V5AZE6 V5AZE6_TOXGO Uncharacterized protein 2045 1623-1628
373. FSSPSS V5B0M0 V5B0M0_TOXGO Uncharacterized protein 934 464-469
374. FSSPSS V5B218 V5B218_TOXGO ATPase/histidine kinase/DNA gyrase B/HSP90 domain-containing protein 1440 812-817
375. FSSPSS V5B3Z0 V5B3Z0_TOXGO FATC domain-containing protein 6012 2243-2248
376. FSSPSS V5B555 V5B555_TOXGO Putative AAA family domain ATPase 2965 1466-1471
377. FSSPSS V5BBQ8 V5BBQ8_TOXGO Leucine rich repeat-containing protein 4458 3634-3639
378. FSSPSS V5BKC0 V5BKC0_TOXGO Uncharacterized protein 2239 448-453
379. FSSPSS V5BMI1 V5BMI1_TOXGO Uncharacterized protein 1082 244-249
380. SSPSSP A8CBH4 A8CBH4_TOXGO Oxygen-dependent protoporphyrinogen IX oxidase 937 189-194
381. SSPSSP B6K9C3 B6K9C3_TOXGO Uncharacterized protein 362 90-95
382. SSPSSP B6K9U0 B6K9U0_TOXGO XRN 5'-3' exonuclease N-terminus protein 2089 1665-1670
383. SSPSSP B6K9V2 B6K9V2_TOXGO WD domain, G-beta repeat-containing protein 1218 101-106
384. SSPSSP B6KA99 B6KA99_TOXGO WD domain, G-beta repeat-containing protein 744 242-247
385. SSPSSP B6KAC5 B6KAC5_TOXGO Uncharacterized protein 421 225-230
386. SSPSSP B6KAS7 B6KAS7_TOXGO PA14 domain-containing protein 998 388-393
387. SSPSSP B6KB41 B6KB41_TOXGO Putative calcium signaling protein kinase RAD53 1436 1299-1304
388. SSPSSP B6KFI3 B6KFI3_TOXGO Putative ADP-ribosylation factor 483 90-95
389. SSPSSP B6KFV2 B6KFV2_TOXGO Atypical MEK-related kinase (Incomplete catalytic triad) 821 58-63, 89-94, 92-97
390. SSPSSP B6KGA9 B6KGA9_TOXGO Uncharacterized protein 1548 1329-1334
391. SSPSSP B6KGF8 B6KGF8_TOXGO Carrier superfamily protein 850 115-120
392. SSPSSP B6KGN7 B6KGN7_TOXGO SprT domain-containing protein 839 548-553
393. SSPSSP B6V6I8 B6V6I8_TOXGO Pseudouridine synthase 1 2117 825-830
394. SSPSSP B9PIE2 B9PIE2_TOXGO Protoporphyrinogen oxidase 1028 189-194
395. SSPSSP B9PT44 B9PT44_TOXGO Uncharacterized protein 166 96-101
396. SSPSSP B9Q2S2 B9Q2S2_TOXGO MtN3/saliva family protein 666 150-155
397. SSPSSP B9Q3G3 B9Q3G3_TOXGO Gpi16 subunit, GPI transamidase component protein 769 133-138, 136-141, 139-144, 142-147
398. SSPSSP B9Q504 B9Q504_TOXGO Uncharacterized protein 4030 1269-1274
399. SSPSSP B9Q562 B9Q562_TOXGO Non-specific serine/threonine protein kinase 8428 5360-5365
400. SSPSSP B9Q6W0 B9Q6W0_TOXGO ThiF family protein 1073 44-49, 47-52
401. SSPSSP B9Q780 B9Q780_TOXGO PHD-finger domain-containing protein 546 217-222
402. SSPSSP B9Q7S4 B9Q7S4_TOXGO Uncharacterized protein 911 402-407
403. SSPSSP B9Q7W5 B9Q7W5_TOXGO Uncharacterized protein 2115 251-256
404. SSPSSP B9Q7W6 B9Q7W6_TOXGO Putative F-box protein 1461 868-873, 921-926, 924-929
405. SSPSSP B9Q8F9 B9Q8F9_TOXGO Putative elongation factor Tu GTP-binding domain protein 1766 1648-1653
406. SSPSSP B9Q8I2 B9Q8I2_TOXGO Uncharacterized protein 2497 334-339
407. SSPSSP B9QE40 B9QE40_TOXGO TBC domain-containing protein 1047 44-49
408. SSPSSP B9QES7 B9QES7_TOXGO Uncharacterized protein 452 157-162
409. SSPSSP B9QFQ5 B9QFQ5_TOXGO Uncharacterized protein 1798 358-363
410. SSPSSP B9QFX0 B9QFX0_TOXGO CRAL/TRIO domain protein 1948 15-20
411. SSPSSP B9QHA2 B9QHA2_TOXGO Subtilisin SUB12 1959 1862-1867, 1865-1870, 1868-1873, 1871-1876, 1878-1883
412. SSPSSP B9QI26 B9QI26_TOXGO Pumilio-family RNA binding repeat-containing protein 1913 638-643
413. SSPSSP B9QLX5 B9QLX5_TOXGO Carrier superfamily protein 716 143-148
414. SSPSSP B9QM71 B9QM71_TOXGO Putative transmembrane protein 2545 538-543, 541-546, 549-554
415. SSPSSP B9QP13 B9QP13_TOXGO Putative histone deacetylase SIR2 1703 284-289, 287-292, 308-313, 328-333
416. SSPSSP B9QQF1 B9QQF1_TOXGO Proteasome/cyclosome repeat-containing protein 3187 651-656
417. SSPSSP B9QR55 B9QR55_TOXGO Putative transmembrane protein 957 265-270
418. SSPSSP D2XQ61 D2XQ61_TOXGO Sodium/hydrogen exchanger 853 154-159
419. SSPSSP K7WT78 K7WT78_TOXGO DHHC15 1327 683-688
420. SSPSSP Q1JSB2 Q1JSB2_TOXGO SWI/SNF family transcriptional activator protein, putative 1383 78-83
421. SSPSSP Q1JSS9 Q1JSS9_TOXGO Uncharacterized protein 799 438-443
422. SSPSSP Q1JT86 Q1JT86_TOXGO Zinc finger, putative 768 167-172, 185-190
423. SSPSSP Q1JTB6 Q1JTB6_TOXGO Methyl transferase, putative 842 526-531
424. SSPSSP Q1JTE7 Q1JTE7_TOXGO Putative uncharacterized protein precursor 651 338-343, 347-352, 356-361
425. SSPSSP Q1JTH9 Q1JTH9_TOXGO Hyothetical protein 1821 1253-1258
426. SSPSSP Q3HLX0 Q3HLX0_TOXGO Mitochondrial alternative NADH dehydrogenase 1 618 30-35
427. SSPSSP Q45W19 Q45W19_TOXGO ATP-binding cassette, sub-family C, member 2 1328 891-896
428. SSPSSP Q6JD66 Q6JD66_TOXGO Eukaryotic initiation factor-2 alpha kinase-A 5072 1370-1375 126226
429. SSPSSP V4YI12 V4YI12_TOXGO Thrombospondin type 1 domain-containing protein 1218 76-81
430. SSPSSP V4YJB7 V4YJB7_TOXGO Protein kinase domain-containing protein 1290 840-845
431. SSPSSP V4YKE7 V4YKE7_TOXGO Calcium-dependent protein kinase CDPK6 1477 517-522
432. SSPSSP V4YLJ7 V4YLJ7_TOXGO Uncharacterized protein 894 292-297
433. SSPSSP V4YLR5 V4YLR5_TOXGO YrdC domain-containing protein 828 161-166
434. SSPSSP V4YLV1 V4YLV1_TOXGO PP-loop domain-containing protein 1701 242-247
435. SSPSSP V4YNU1 V4YNU1_TOXGO Putative transmembrane protein 3158 2161-2166
436. SSPSSP V4YPY6 V4YPY6_TOXGO Uncharacterized protein 2436 76-81
437. SSPSSP V4YQT3 V4YQT3_TOXGO Putative transmembrane protein 3329 1813-1818, 1816-1821, 3035-3040
438. SSPSSP V4YRV5 V4YRV5_TOXGO Putative transmembrane protein 918 900-905
439. SSPSSP V4YTH9 V4YTH9_TOXGO DEAD/DEAH box helicase domain-containing protein 4426 2562-2567
440. SSPSSP V4YTX2 V4YTX2_TOXGO Uncharacterized protein 2251 2094-2099
441. SSPSSP V4YTY4 V4YTY4_TOXGO Dullard family phosphatase domain-containing protein 479 338-343, 341-346, 344-349
442. SSPSSP V4YV20 V4YV20_TOXGO Uncharacterized protein 2126 22-27
443. SSPSSP V4YV99 V4YV99_TOXGO Uncharacterized protein 1717 441-446
444. SSPSSP V4YXQ4 V4YXQ4_TOXGO Ubiquitin carboxyl-terminal hydrolase 3144 1540-1545
445. SSPSSP V4YZ55 V4YZ55_TOXGO DNA repair protein Rad4 domain-containing protein 1935 1132-1137, 1135-1140
446. SSPSSP V4YZC1 V4YZC1_TOXGO Uncharacterized protein 3026 1290-1295, 2661-2666
447. SSPSSP V4Z060 V4Z060_TOXGO Putative GTP binding protein 7 isoform 2 family protein 453 36-41
448. SSPSSP V4Z0M3 V4Z0M3_TOXGO Putative WDSUB1 family SAM domain protein 1912 712-717, 728-733
449. SSPSSP V4Z106 V4Z106_TOXGO Uncharacterized protein 2597 490-495, 493-498
450. SSPSSP V4Z1V2 V4Z1V2_TOXGO Uncharacterized protein 898 704-709
451. SSPSSP V4Z222 V4Z222_TOXGO RNA-directed DNA polymerase 4625 1522-1527
452. SSPSSP V4Z2F7 V4Z2F7_TOXGO Putative transmembrane protein 2862 416-421
453. SSPSSP V4Z2M0 V4Z2M0_TOXGO Uncharacterized protein 2289 1977-1982
454. SSPSSP V4Z2T9 V4Z2T9_TOXGO Uncharacterized protein 2435 656-661
455. SSPSSP V4Z2X5 V4Z2X5_TOXGO Uncharacterized protein 1443 78-83
456. SSPSSP V4Z303 V4Z303_TOXGO DnaJ domain-containing protein 2010 719-724
457. SSPSSP V4Z344 V4Z344_TOXGO Ankyrin repeat-containing protein 882 524-529
458. SSPSSP V4Z377 V4Z377_TOXGO Ribosomal protein L15 protein 1395 204-209, 231-236, 246-251, 752-757
459. SSPSSP V4Z3B7 V4Z3B7_TOXGO Uncharacterized protein 2613 1074-1079, 1089-1094, 1114-1119
460. SSPSSP V4Z3K3 V4Z3K3_TOXGO PIK3R4 kinase-related protein (Incomplete catalytic triad) 3028 1664-1669
461. SSPSSP V4Z3N0 V4Z3N0_TOXGO Uncharacterized protein 1403 645-650
462. SSPSSP V4Z3S9 V4Z3S9_TOXGO BTB/POZ domain protein 1849 1058-1063
463. SSPSSP V4Z4C0 V4Z4C0_TOXGO Uncharacterized protein 2996 994-999, 2430-2435
464. SSPSSP V4Z4E2 V4Z4E2_TOXGO Elongation factor G C-terminus domain-containing protein 874 112-117
465. SSPSSP V4Z4J5 V4Z4J5_TOXGO tRNA ligase class II core domain (G, H, P, S and T) domain-containing protein 1209 707-712, 714-719
466. SSPSSP V4Z4Z1 V4Z4Z1_TOXGO Histone lysine demethylase JMJD6b 740 646-651
467. SSPSSP V4Z559 V4Z559_TOXGO Putative histone lysine methyltransferase, SET 5175 1904-1909
468. SSPSSP V4Z585 V4Z585_TOXGO ThiF family protein 2933 1472-1477
469. SSPSSP V4Z693 V4Z693_TOXGO Uncharacterized protein 651 239-244
470. SSPSSP V4Z6A3 V4Z6A3_TOXGO Putative DNA double-strand break repair rad50 ATPase 619 34-39, 37-42
471. SSPSSP V4Z6I1 V4Z6I1_TOXGO GAF domain protein 1026 812-817
472. SSPSSP V4Z6U5 V4Z6U5_TOXGO Uncharacterized protein 1269 49-54, 57-62
473. SSPSSP V4Z7P3 V4Z7P3_TOXGO Uncharacterized protein 1860 1477-1482
474. SSPSSP V4Z7R9 V4Z7R9_TOXGO Uncharacterized protein 1083 837-842
475. SSPSSP V4Z7T4 V4Z7T4_TOXGO Elongation factor Tu GTP binding domain-containing protein 1331 745-750
476. SSPSSP V4Z7V3 V4Z7V3_TOXGO Putative transmembrane protein 1948 362-367
477. SSPSSP V4Z801 V4Z801_TOXGO Uncharacterized protein 2495 1352-1357
478. SSPSSP V4Z827 V4Z827_TOXGO Glucose inhibited division protein A subfamily protein 1512 1208-1213, 1336-1341, 1339-1344, 1342-1347
479. SSPSSP V4Z842 V4Z842_TOXGO DUSP domain protein 1154 848-853
480. SSPSSP V4Z883 V4Z883_TOXGO SRP72 RNA-binding domain-containing protein 731 423-428
481. SSPSSP V4Z8D8 V4Z8D8_TOXGO Putative zinc carboxypeptidase 2203 752-757, 755-760, 762-767, 765-770
482. SSPSSP V4Z8E9 V4Z8E9_TOXGO Hydrolase, TatD family protein 557 274-279
483. SSPSSP V4Z8G3 V4Z8G3_TOXGO RNA-dependent RNA polymerase RDP 2894 1957-1962
484. SSPSSP V4Z923 V4Z923_TOXGO RNase P subunit p30 632 540-545
485. SSPSSP V4Z9F1 V4Z9F1_TOXGO Zinc finger, C3HC4 type (RING finger) domain-containing protein 1284 704-709, 735-740
486. SSPSSP V4Z9U8 V4Z9U8_TOXGO Uncharacterized protein 630 338-343, 347-352, 356-361
487. SSPSSP V4Z9W0 V4Z9W0_TOXGO Uncharacterized protein 1434 202-207
488. SSPSSP V4Z9X0 V4Z9X0_TOXGO ATP-dependent Clp endopeptidase, proteolytic subunit ClpP 594 142-147
489. SSPSSP V4Z9Y5 V4Z9Y5_TOXGO Uncharacterized protein 1461 1206-1211, 1209-1214, 1221-1226
490. SSPSSP V4ZAE5 V4ZAE5_TOXGO Aldo-keto reductase 1425 423-428
491. SSPSSP V4ZAT6 V4ZAT6_TOXGO DEAD/DEAH box helicase domain-containing protein 1773 759-764
492. SSPSSP V4ZAY6 V4ZAY6_TOXGO Spc97 / Spc98 family protein 2023 362-367
493. SSPSSP V4ZBL5 V4ZBL5_TOXGO Uncharacterized protein 1033 714-719, 717-722, 720-725, 723-728
494. SSPSSP V4ZBN7 V4ZBN7_TOXGO Uncharacterized protein 2456 349-354
495. SSPSSP V4ZBS8 V4ZBS8_TOXGO ABC transporter transmembrane region domain-containing protein 1951 1435-1440
496. SSPSSP V4ZBX8 V4ZBX8_TOXGO Phosphatidylinositol 3-and 4-kinase 6746 1090-1095
497. SSPSSP V4ZCI0 V4ZCI0_TOXGO Methyltransferase domain protein 440 212-217
498. SSPSSP V4ZCT4 V4ZCT4_TOXGO Uncharacterized protein 768 328-333
499. SSPSSP V4ZCY2 V4ZCY2_TOXGO DEAD/DEAH box helicase domain-containing protein 1867 397-402, 400-405, 403-408
500. SSPSSP V4ZCY7 V4ZCY7_TOXGO Sodium/hydrogen exchanger 853 154-159
501. SSPSSP V4ZDQ0 V4ZDQ0_TOXGO Glycine cleavage T-protein (Aminomethyl transferase) domain-containing protein 1807 1301-1306
502. SSPSSP V4ZDR5 V4ZDR5_TOXGO NLI interacting factor family phosphatase 500 140-145
503. SSPSSP V4ZE20 V4ZE20_TOXGO AP2 domain transcription factor AP2V-2 3456 1550-1555, 1649-1654
504. SSPSSP V4ZEC8 V4ZEC8_TOXGO Uncharacterized protein 1400 309-314
505. SSPSSP V4ZEF3 V4ZEF3_TOXGO Putative transmembrane protein 1605 792-797
506. SSPSSP V4ZEG5 V4ZEG5_TOXGO Uncharacterized protein 1277 96-101, 99-104
507. SSPSSP V4ZEK9 V4ZEK9_TOXGO Putative transmembrane protein 579 68-73
508. SSPSSP V4ZET0 V4ZET0_TOXGO Putative DNA topoisomerase I 890 68-73
509. SSPSSP V4ZEV2 V4ZEV2_TOXGO Uncharacterized protein 1205 508-513
510. SSPSSP V4ZEW9 V4ZEW9_TOXGO CorA family Mg2+ transporter protein 1190 383-388
511. SSPSSP V4ZEX6 V4ZEX6_TOXGO Uncharacterized protein 697 427-432
512. SSPSSP V4ZF86 V4ZF86_TOXGO Putative transmembrane protein 476 36-41
513. SSPSSP V4ZFD5 V4ZFD5_TOXGO AP2 domain transcription factor AP2IX-7 2672 1714-1719
514. SSPSSP V4ZFP2 V4ZFP2_TOXGO IgA-specific metalloendopeptidase 1814 1680-1685
515. SSPSSP V4ZFS7 V4ZFS7_TOXGO Zinc finger, C3HC4 type (RING finger) domain-containing protein 1500 93-98, 96-101, 99-104
516. SSPSSP V4ZFY2 V4ZFY2_TOXGO Peptidyl-prolyl cis-trans isomerase 283 27-32
517. SSPSSP V4ZFZ2 V4ZFZ2_TOXGO Sec7 domain-containing protein 3546 615-620
518. SSPSSP V4ZG98 V4ZG98_TOXGO RNB family domain-containing protein 1165 944-949
519. SSPSSP V4ZGA9 V4ZGA9_TOXGO GTP1/Obg protein 1917 1495-1500, 1498-1503
520. SSPSSP V4ZGM6 V4ZGM6_TOXGO Methyltransferase domain-containing protein 883 253-258
521. SSPSSP V4ZGP4 V4ZGP4_TOXGO Mannosyl-oligosaccharide glucosidase 1384 78-83, 86-91, 886-891
522. SSPSSP V4ZGQ4 V4ZGQ4_TOXGO Uncharacterized protein 2272 527-532
523. SSPSSP V4ZGQ7 V4ZGQ7_TOXGO Putative activating signal cointegrator 1 complex subunit 3 2304 10-15, 13-18
524. SSPSSP V4ZH67 V4ZH67_TOXGO Uncharacterized protein 3076 2082-2087, 2085-2090
525. SSPSSP V4ZHC8 V4ZHC8_TOXGO Uncharacterized protein 1667 667-672
526. SSPSSP V4ZHK2 V4ZHK2_TOXGO Phosphatidate cytidylyltransferase 1047 379-384, 382-387
527. SSPSSP V4ZHS6 V4ZHS6_TOXGO Putative transmembrane protein 889 117-122
528. SSPSSP V4ZIG9 V4ZIG9_TOXGO Putative transmembrane protein 1334 995-1000
529. SSPSSP V4ZJE9 V4ZJE9_TOXGO Enhancer of polycomb-like protein 1474 45-50
530. SSPSSP V4ZJT7 V4ZJT7_TOXGO RAP domain-containing protein 1011 450-455
531. SSPSSP V4ZJZ9 V4ZJZ9_TOXGO ATPase, AAA family protein 3910 87-92
532. SSPSSP V4ZKD9 V4ZKD9_TOXGO Putative ATP-dependent Clp protease proteolytic subunit,related protein 900 663-668
533. SSPSSP V4ZKH7 V4ZKH7_TOXGO SWI2/SNF2-containing protein 1537 942-947
534. SSPSSP V4ZL93 V4ZL93_TOXGO Uncharacterized protein 1323 144-149
535. SSPSSP V4ZL97 V4ZL97_TOXGO Uncharacterized protein 7954 3942-3947
536. SSPSSP V4ZLE5 V4ZLE5_TOXGO Uncharacterized protein 2186 856-861
537. SSPSSP V4ZLY5 V4ZLY5_TOXGO Uncharacterized protein 1281 123-128
538. SSPSSP V4ZM09 V4ZM09_TOXGO PET112 family, C terminal region domain-containing protein 1070 112-117
539. SSPSSP V4ZM26 V4ZM26_TOXGO Regulator of chromosome condensation (RCC1) repeat-containing protein 762 324-329
540. SSPSSP V4ZNF6 V4ZNF6_TOXGO Putative glutamic acid-rich protein 2698 2104-2109
541. SSPSSP V4ZNI0 V4ZNI0_TOXGO Putative transmembrane protein 6079 4434-4439
542. SSPSSP V4ZP18 V4ZP18_TOXGO Putative transmembrane protein 2590 801-806
543. SSPSSP V4ZP54 V4ZP54_TOXGO Uncharacterized protein 2445 1602-1607, 1808-1813, 1811-1816, 1814-1819, 1970-1975, 2035-2040
544. SSPSSP V4ZPH3 V4ZPH3_TOXGO Protein kinase domain protein 2329 963-968
545. SSPSSP V4ZPQ8 V4ZPQ8_TOXGO Symplekin tight junction carboxy-terminal protein 1891 1142-1147
546. SSPSSP V4ZSB4 V4ZSB4_TOXGO Putative zinc finger protein 969 614-619
547. SSPSSP V4ZSL1 V4ZSL1_TOXGO Putative transmembrane protein 1892 275-280
548. SSPSSP V4ZSR1 V4ZSR1_TOXGO DNA-directed RNA polymerase, alpha subunit 1113 323-328, 326-331, 329-334, 332-337
549. SSPSSP V4ZTQ1 V4ZTQ1_TOXGO DEAD/DEAH box helicase domain-containing protein 1991 75-80, 111-116
550. SSPSSP V4ZU83 V4ZU83_TOXGO eIF2 kinase IF2K-A (Incomplete catalytic triad) 4638 936-941
551. SSPSSP V4ZVI6 V4ZVI6_TOXGO SufB/sufD domain-containing protein 1860 623-628
552. SSPSSP V4ZW70 V4ZW70_TOXGO Uncharacterized protein 5655 625-630
553. SSPSSP V5AXL6 V5AXL6_TOXGO Tyrosine kinase-like (TKL) protein 3571 3245-3250
554. SSPSSP V5AY25 V5AY25_TOXGO Dolichyl-phosphate beta-glucosyltransferase 704 176-181, 179-184
555. SSPSSP V5AZA6 V5AZA6_TOXGO Sodium/hydrogen exchanger 1572 426-431
556. SSPSSP V5AZN9 V5AZN9_TOXGO Putative transmembrane protein 486 96-101, 99-104
557. SSPSSP V5B018 V5B018_TOXGO Zinc finger, C3HC4 type (RING finger) domain-containing protein 768 167-172, 185-190
558. SSPSSP V5B055 V5B055_TOXGO NOL1/NOP2/sun family protein 970 556-561
559. SSPSSP V5B086 V5B086_TOXGO DHHC zinc finger domain-containing protein 1115 683-688
560. SSPSSP V5B0G2 V5B0G2_TOXGO DNA ligase 1331 803-808
561. SSPSSP V5B124 V5B124_TOXGO SWI2/SNF2 ISWI-like SANT 1249 78-83
562. SSPSSP V5B2I5 V5B2I5_TOXGO HECT-domain (Ubiquitin-transferase) domain-containing protein 1674 88-93
563. SSPSSP V5B2X1 V5B2X1_TOXGO Uncharacterized protein 4035 2233-2238, 2788-2793
564. SSPSSP V5B399 V5B399_TOXGO PRELI family protein 482 297-302, 300-305, 303-308, 306-311
565. SSPSSP V5B4F8 V5B4F8_TOXGO Non-specific serine/threonine protein kinase 960 674-679
566. SSPSSP V5B857 V5B857_TOXGO Pentatricopeptide repeat domain-containing protein 2538 452-457
567. SSPSSP V5B8F2 V5B8F2_TOXGO Peptidase family c50 protein 6890 195-200, 2334-2339
568. SSPSSP V5B8F8 V5B8F8_TOXGO Putative transmembrane protein 2667 588-593, 591-596, 594-599
569. SSPSSP V5B9C4 V5B9C4_TOXGO Uncharacterized protein 1271 363-368
570. SSPSSP V5B9N0 V5B9N0_TOXGO ABC1 family protein 1640 1119-1124
571. SSPSSP V5BAT5 V5BAT5_TOXGO Eukaryotic glutathione synthase, atp binding domain-containing protein 1474 1371-1376
572. SSPSSP V5BBW1 V5BBW1_TOXGO RNA pseudouridine synthase superfamily protein 2252 957-962
573. SSPSSP V5BC25 V5BC25_TOXGO ABC1 family protein 942 670-675
574. SSPSSP V5BDC0 V5BDC0_TOXGO Uncharacterized protein 1391 606-611
575. SSPSSP V5BDW3 V5BDW3_TOXGO Las1 family protein 1717 700-705
576. SSPSSP V5BDZ6 V5BDZ6_TOXGO Uncharacterized protein 4591 3449-3454
577. SSPSSP V5BE57 V5BE57_TOXGO Chloride transporter, chloride channel (ClC) family protein 2052 746-751
578. SSPSSP V5BEH6 V5BEH6_TOXGO Uncharacterized protein 1275 870-875
579. SSPSSP V5BGB2 V5BGB2_TOXGO Carrier superfamily protein 592 143-148
580. SSPSSP V5BH35 V5BH35_TOXGO Uncharacterized protein 599 160-165, 163-168
581. SSPSSP V5BHR4 V5BHR4_TOXGO Uncharacterized protein 878 162-167
582. SSPSSP V5BHW3 V5BHW3_TOXGO PGAP1 family protein 2784 711-716
583. SSPSSP V5BJX1 V5BJX1_TOXGO Uncharacterized protein 1332 642-647
584. SSPSSP V5BKV1 V5BKV1_TOXGO Protein phosphatase 2C domain-containing protein 4071 1505-1510
585. SSPSSP V5BL43 V5BL43_TOXGO Uncharacterized protein 2687 941-946
586. SSPSSP V5BM22 V5BM22_TOXGO WD domain, G-beta repeat-containing protein 2869 1070-1075
587. SSPSSP V5BMI1 V5BMI1_TOXGO Uncharacterized protein 1082 645-650
588. SPSSPW V4ZP14 V4ZP14_TOXGO RIC1 protein 3789 3646-3651
589. SPSSPW V5BBQ8 V5BBQ8_TOXGO Leucine rich repeat-containing protein 4458 921-926
590. PSSPWS V5BBQ8 V5BBQ8_TOXGO Leucine rich repeat-containing protein 4458 922-927
591. SSPWSS V4ZG44 V4ZG44_TOXGO Putative transmembrane protein 2478 412-417
592. SSPWSS V5BBQ8 V5BBQ8_TOXGO Leucine rich repeat-containing protein 4458 923-928
593. SSDPFS B6KH95 B6KH95_TOXGO Uncharacterized protein 3071 370-375
594. SSDPFS Q45KY9 Q45KY9_TOXGO Structural maintenance of chromosomes protein 1479 1312-1317
595. SSDPFS V4YZE7 V4YZE7_TOXGO Uncharacterized protein 1048 715-720
596. SSDPFS V4ZPP4 V4ZPP4_TOXGO Structural maintenance of chromosomes protein 1528 1361-1366
597. FSFLQS B9Q589 B9Q589_TOXGO Putative transmembrane protein 289 84-89
598. VQGVSA B9Q751 B9Q751_TOXGO Subtilisin SUB8 1366 54-59
599. QGVSAL B9Q751 B9Q751_TOXGO Subtilisin SUB8 1366 55-60
600. QGVSAL V4ZDT2 V4ZDT2_TOXGO Putative transmembrane protein 930 86-91
601. GVSALL B6KAG9 B6KAG9_TOXGO U3 small nucleolar RNA-associated protein 10 3738 1488-1493
602. GVSALL B6KJA3 B6KJA3_TOXGO Putative 50S ribosomal protein L3 514 169-174
603. GVSALL V4YU44 V4YU44_TOXGO Uncharacterized protein 2391 1540-1545
604. GVSALL V4ZPZ2 V4ZPZ2_TOXGO ARID/BRIGHT DNA binding domain-containing protein 2713 2587-2592
605. GVSALL V5BBQ8 V5BBQ8_TOXGO Leucine rich repeat-containing protein 4458 953-958
606. VSALLA B9QFP6 B9QFP6_TOXGO Rad17 cell cycle checkpoint protein 1867 647-652
607. VSALLA Q3HLX0 Q3HLX0_TOXGO Mitochondrial alternative NADH dehydrogenase 1 618 39-44
608. VSALLA V4Z8B4 V4Z8B4_TOXGO Putative thioredoxin 711 466-471
609. VSALLA V4Z9X0 V4Z9X0_TOXGO ATP-dependent Clp endopeptidase, proteolytic subunit ClpP 594 194-199
610. VSALLA V4ZM49 V4ZM49_TOXGO Putative lysosomal cobalamin transporter,related protein 774 384-389
611. VSALLA V4ZRC9 V4ZRC9_TOXGO Uncharacterized protein 737 200-205
612. VSALLA V5B9G5 V5B9G5_TOXGO Ribosomal RNA-processing protein 7 376 143-148
613. SALLAF V4ZFD5 V4ZFD5_TOXGO AP2 domain transcription factor AP2IX-7 2672 2025-2030
614. SALLAF V4ZM49 V4ZM49_TOXGO Putative lysosomal cobalamin transporter,related protein 774 385-390
615. ALLAFP V4YN15 V4YN15_TOXGO Putative transmembrane protein 1062 531-536
616. ALLAFP V4Z7Q3 V4Z7Q3_TOXGO Uncharacterized protein 2521 1042-1047
617. ALLAFP V5AYI0 V5AYI0_TOXGO NEK kinase 2906 575-580
618. ALLAFP V5AYL8 V5AYL8_TOXGO Uncharacterized protein 1236 820-825
619. LLAFPQ V5B7V4 V5B7V4_TOXGO tRNA pseudouridine synthase D 1489 1386-1391
620. LDLVSL Q1JTI7 Q1JTI7_TOXGO Putative uncharacterized protein 3352 256-261
621. LDLVSL V4Z556 V4Z556_TOXGO Zinc finger in N-recognin protein 4439 1125-1130
622. LDLVSL V5BB89 V5BB89_TOXGO Putative transmembrane protein 3658 3229-3234
623. DLVSLV B9PI32 B9PI32_TOXGO Sec1 family protein 648 7-12
624. DLVSLV B9PL31 B9PL31_TOXGO SAG-related sequence SRS28 291 210-215
625. DLVSLV Q6RUA7 Q6RUA7_TOXGO Sporozoite-specific SAG protein 291 210-215
626. DLVSLV V4YNL5 V4YNL5_TOXGO Uncharacterized protein 2072 1777-1782
627. DLVSLV V5B3G4 V5B3G4_TOXGO Uncharacterized protein 924 833-838
628. LVSLVL B6KJ39 B6KJ39_TOXGO HEAT repeat-containing protein 1238 39-44
629. LVSLVL V5B4Q4 V5B4Q4_TOXGO Uncharacterized protein 1046 29-34
630. VSLVLH V4Z788 V4Z788_TOXGO Uncharacterized protein 675 144-149
631. VLHIPV V4Z2Q9 V4Z2Q9_TOXGO Uncharacterized protein 665 477-482
632. LHIPVI V5BI48 V5BI48_TOXGO Uncharacterized protein 307 224-229
633. HEFPRE V4Z3L3 V4Z3L3_TOXGO Ribosomal death-associated protein 3 545 516-521
634. PLHLQL V4Z7B6 V4Z7B6_TOXGO Uncharacterized protein 607 307-312
635. LHLQLS B9PRV7 B9PRV7_TOXGO Zn-finger in Ran binding protein and others domain-containing protein 1258 60-65
636. LHLQLS B9PY04 B9PY04_TOXGO Uncharacterized protein 3263 1947-1952
637. HLQLSL B9PSR7 B9PSR7_TOXGO Uncharacterized protein 417 51-56
638. HLQLSL B9Q072 B9Q072_TOXGO RNA recognition motif-containing protein 274 209-214
639. HLQLSL V4YR36 V4YR36_TOXGO Uncharacterized protein 562 196-201
640. QLSLEN Q1JSM9 Q1JSM9_TOXGO Uncharacterized protein 3444 631-636
641. LSLENS Q1JTE7 Q1JTE7_TOXGO Putative uncharacterized protein precursor 651 97-102
642. LSLENS V4Z9U8 V4Z9U8_TOXGO Uncharacterized protein 630 97-102
643. SLENSL Q1JTE7 Q1JTE7_TOXGO Putative uncharacterized protein precursor 651 98-103
644. SLENSL V4Z9U8 V4Z9U8_TOXGO Uncharacterized protein 630 98-103
645. LENSLS B9QMH6 B9QMH6_TOXGO RNA pseudouridine synthase superfamily protein 6535 503-508
646. LENSLS Q1JSC2 Q1JSC2_TOXGO Uncharacterized protein 335 87-92
647. LENSLS V4Z6K3 V4Z6K3_TOXGO Uncharacterized protein 326 87-92
648. ENSLSS V4YZ95 V4YZ95_TOXGO Kinesin motor domain-containing protein 2394 300-305
649. ENSLSS V4Z8G0 V4Z8G0_TOXGO Guanylyl cyclase 4367 2663-2668
650. ENSLSS V4ZIQ1 V4ZIQ1_TOXGO Uncharacterized protein 1557 1463-1468
651. NSLSSD V4ZB19 V4ZB19_TOXGO Uncharacterized protein 386 267-272
652. SLSSDA B6KHL5 B6KHL5_TOXGO DNA-directed RNA polymerase I RPA12 348 10-15
653. SLSSDA B9Q0L1 B9Q0L1_TOXGO Peptidase family c78 protein 853 166-171
654. SLSSDA V4YT41 V4YT41_TOXGO Protein kinase domain protein 6052 2762-2767
655. SLSSDA V4Z4I3 V4Z4I3_TOXGO Peptidyl-tRNA hydrolase PTH2 domain-containing protein 266 97-102
656. SLSSDA V4ZE20 V4ZE20_TOXGO AP2 domain transcription factor AP2V-2 3456 1510-1515
657. SLSSDA V4ZP08 V4ZP08_TOXGO Uncharacterized protein 3263 3082-3087
658. SLSSDA V5B3Z0 V5B3Z0_TOXGO FATC domain-containing protein 6012 2435-2440
659. SLSSDA V5BJ10 V5BJ10_TOXGO Ribosomal l25 family protein 739 450-455
660. LSSDAD V4ZAX6 V4ZAX6_TOXGO Uncharacterized protein 8643 5975-5980
661. SDADVT B9QQK8 B9QQK8_TOXGO SAG-related sequence SRS12A 386 118-123
662. SDADVT Q7YWC3 Q7YWC3_TOXGO Putative GPI-anchored surface BSR4-related antigen 386 118-123
663. ADVTVS B9QAZ9 B9QAZ9_TOXGO Ankyrin repeat-containing protein 1382 1178-1183
664. NFSLLL V4ZDY2 V4ZDY2_TOXGO Ribonuclease type III Dicer 4343 956-961
665. FSLLLC B9Q510 B9Q510_TOXGO Putative transmembrane protein 737 606-611
666. FSLLLC V5BCB9 V5BCB9_TOXGO Putative autoantigen, coiled-coil vesicle tethering subfamily A protein 1 431 2-7
667. SLLLCQ B9Q510 B9Q510_TOXGO Putative transmembrane protein 737 607-612
668. LLLCQE B9Q510 B9Q510_TOXGO Putative transmembrane protein 737 608-613
669. TQNNSK V4ZHM8 V4ZHM8_TOXGO Uncharacterized protein 249 191-196
670. LGSIIN B6KGR6 B6KGR6_TOXGO Protein phosphatase 2a regulatory b subunit (B56 family) protein 578 349-354
671. PSTQDL V4ZDS0 V4ZDS0_TOXGO AP2 domain transcription factor AP2IX-1 1009 436-441
672. STQDLL B6KT44 B6KT44_TOXGO FUSE-binding protein 2 / KH-type splicing regulatory protein 941 44-49
673. DLLSFL V4Z9W0 V4Z9W0_TOXGO Uncharacterized protein 1434 636-641
674. DLLSFL V5BL43 V5BL43_TOXGO Uncharacterized protein 2687 1232-1237
675. LLSFLQ B9QI99 B9QI99_TOXGO Kinesin motor domain-containing protein 1317 1160-1165
676. LLSFLQ V4ZAK3 V4ZAK3_TOXGO Uncharacterized protein 3378 2385-2390
677. LLSFLQ V4ZDH3 V4ZDH3_TOXGO Sigma-70, region 3 protein 1233 113-118
678. LLSFLQ V4ZH21 V4ZH21_TOXGO GTPase 1352 1163-1168
679. LSFLQI V4ZDH3 V4ZDH3_TOXGO Sigma-70, region 3 protein 1233 114-119
680. IQLESI V4Z7H9 V4Z7H9_TOXGO Ion channel protein 2540 2444-2449
681. SIRRIF A5YVK6 A5YVK6_TOXGO Regulator of chromosome condensation 1 1155 589-594
682. SIRRIF B9QGB2 B9QGB2_TOXGO Regulator of chromosome condensation RCC1 1156 589-594
683. IRRIFE V5B415 V5B415_TOXGO DEAD/DEAH box helicase domain-containing protein 379 58-63
684. ELRWVL B6KHQ9 B6KHQ9_TOXGO Tetratricopeptide repeat-containing protein 2068 108-113
685. NVEELR B6KHT0 B6KHT0_TOXGO Flap endonuclease 1 552 121-126
686. VEELRT B6K973 B6K973_TOXGO Zinc finger, C3HC4 type (RING finger) domain-containing protein 2190 1617-1622
687. VEELRT V4Z4K1 V4Z4K1_TOXGO Peptidase, S9A/B/C family, catalytic domain protein 2343 1557-1562
688. VEELRT V5AZE6 V5AZE6_TOXGO Uncharacterized protein 2045 1411-1416
689. EELRTE B9QE40 B9QE40_TOXGO TBC domain-containing protein 1047 986-991
690. LRTEGL B9QH54 B9QH54_TOXGO Phospho-2-dehydro-3-deoxyheptonate aldolase,related protein 1052 964-969
691. RTEGLP V4Z4Z4 V4Z4Z4_TOXGO Uncharacterized protein 148 50-55
692. RTEGLP V4ZG50 V4ZG50_TOXGO Uncharacterized protein 1834 588-593
693. TEGLPL V4Z4Z4 V4Z4Z4_TOXGO Uncharacterized protein 148 51-56
694. GLPLGL B6K9F0 B6K9F0_TOXGO Ubiquitin carboxyl-terminal hydrolase 1697 620-625
695. GLPLGL V4Z2G8 V4Z2G8_TOXGO Uncharacterized protein 394 128-133
696. LPLGLI B9QNZ6 B9QNZ6_TOXGO MerC mercury resistance protein 181 70-75
697. LPLGLI V4ZDD1 V4ZDD1_TOXGO PHD-finger domain-containing protein 1073 128-133
698. AMELVA V4ZMS0 V4ZMS0_TOXGO Putative anonymous antigen-1 2639 425-430
699. ELVARA V4Z8N9 V4Z8N9_TOXGO TAP42 family protein 512 305-310
700. VARAVA B9Q0M5 B9Q0M5_TOXGO Putative trichohyalin 1427 814-819
701. ARAVAT A7Y418 A7Y418_TOXGO Membrane protein FtsH1 1250 401-406
702. ARAVAT V4ZE58 V4ZE58_TOXGO Membrane protein FtsH1 1250 401-406
703. RAVATA B9PJB3 B9PJB3_TOXGO Uncharacterized protein 139 77-82
704. ELALIP V4Z8K6 V4Z8K6_TOXGO Uncharacterized protein 946 366-371
705. LALIPS V4Z8K6 V4Z8K6_TOXGO Uncharacterized protein 946 367-372
706. ALIPST V4Z831 V4Z831_TOXGO Putative transmembrane protein 766 497-502
707. TTNLTS B9QJE8 B9QJE8_TOXGO Uncharacterized protein 3150 516-521
708. TTNLTS V4YMQ7 V4YMQ7_TOXGO Uncharacterized protein 203 187-192
709. LSRFLA B6KB54 B6KB54_TOXGO Uncharacterized protein 182 64-69
710. LSRFLA V4ZA17 V4ZA17_TOXGO Uncharacterized protein 2390 925-930
711. LSRFLA V4ZEE5 V4ZEE5_TOXGO Uncharacterized protein 3039 2113-2118
712. ANTTFR V4ZF78 V4ZF78_TOXGO Haloacid dehalogenase family hydrolase domain-containing protein 2473 687-692
713. TFRGLS V4ZE02 V4ZE02_TOXGO Dynein heavy chain family protein 3987 3336-3341
714. RGLSGS V4Z2Y1 V4Z2Y1_TOXGO Uncharacterized protein 3246 1730-1735
715. IRVKGS B9QEY7 B9QEY7_TOXGO Uncharacterized protein 2300 627-632
716. PEQAQR B6KFU0 B6KFU0_TOXGO DUF862 domain-containing protein 1092 738-743
717. PEQAQR B6KR52 B6KR52_TOXGO mRNA capping enzyme 509 86-91
718. QAQRHK V4ZR91 V4ZR91_TOXGO Uncharacterized protein 3903 1816-1821
719. PSKLHL V5BMA5 V5BMA5_TOXGO Uncharacterized protein 2399 2061-2066
720. LHLRVV V4Z402 V4Z402_TOXGO Uncharacterized protein 1883 375-380
721. LRVVTL V4Z2E9 V4Z2E9_TOXGO AP2 domain transcription factor AP2IV-5 1966 1832-1837
722. RVVTLI V5BHN2 V5BHN2_TOXGO Transmembrane amino acid transporter 2130 17-22
723. EHPFVF B9QFF3 B9QFF3_TOXGO Putative transmembrane protein 2209 371-376
724. REVDDE V4YYU7 V4YYU7_TOXGO Putative glyoxalase 4813 638-643
725. REVDDE V4ZLZ2 V4ZLZ2_TOXGO Hydrolase 3142 939-944
726. EVDDEG V4Z3T6 V4Z3T6_TOXGO Uncharacterized protein 1806 74-79
727. EVDDEG V4ZLZ2 V4ZLZ2_TOXGO Hydrolase 3142 940-945
728. EVDDEG V4ZWM5 V4ZWM5_TOXGO Putative blood stage antigen 41-3 441 292-297
729. GLCPAG B6KRJ1 B6KRJ1_TOXGO Uncharacterized protein 2272 1110-1115
730. GLCPAG Q1JSY4 Q1JSY4_TOXGO Uncharacterized protein 3344 733-738, 1008-1013
731. GLCPAG V4Z1R2 V4Z1R2_TOXGO GCC2 and GCC3 domain-containing protein 5081 2502-2507, 2777-2782
732. CPAGQL B9Q5K9 B9Q5K9_TOXGO Uncharacterized protein 1656 1632-1637
733. SSTLDS B6KAY9 B6KAY9_TOXGO Uncharacterized protein 1116 758-763
734. SSTLDS V5B595 V5B595_TOXGO Pantothenate kinase 1672 355-360
735. TLDSLF V4ZED0 V4ZED0_TOXGO Thioredoxin domain protein 1014 831-836
736. LDSLFS B9Q791 B9Q791_TOXGO LSM domain protein 166 127-132
737. LDSLFS V4Z0S5 V4Z0S5_TOXGO HEAT repeat-containing protein 2505 1335-1340
738. DSLFSS B9QP30 B9QP30_TOXGO Leucine-rich repeat protein LRR1 369 224-229
739. DSLFSS Q1KTE8 Q1KTE8_TOXGO Leucine-rich repeat protein 1 369 224-229
740. DSLFSS V4YKX4 V4YKX4_TOXGO Serine/threonine specific protein phosphatase 2883 715-720
741. SLFSSL B9QJX1 B9QJX1_TOXGO tRNA ligase class I (E and Q), catalytic domain-containing protein 1420 87-92
742. SLFSSL B9QLV4 B9QLV4_TOXGO Putative edge expressed protein 313 298-303
743. SLFSSL V4YZA1 V4YZA1_TOXGO RNA 2'-phosphotransferase, Tpt1/KptA family protein 1305 1072-1077
744. SLFSSL V4Z1R3 V4Z1R3_TOXGO Synaptobrevin 557 36-41
745. SLFSSL V4Z6D9 V4Z6D9_TOXGO Subtilisin SUB11 1213 440-445
746. SLFSSL V4Z7G5 V4Z7G5_TOXGO Uncharacterized protein 449 239-244
747. SLFSSL V4ZBX8 V4ZBX8_TOXGO Phosphatidylinositol 3-and 4-kinase 6746 3136-3141
748. SLFSSL V4ZF86 V4ZF86_TOXGO Putative transmembrane protein 476 18-23
749. SLFSSL V4ZGQ4 V4ZGQ4_TOXGO Uncharacterized protein 2272 897-902
750. SLFSSL V4ZJN0 V4ZJN0_TOXGO MCM2/3/5 family protein 1238 55-60, 95-100, 99-104, 103-108, 107-112, 143-148
751. SLFSSL V4ZPR1 V4ZPR1_TOXGO Putative transmembrane protein 2050 368-373
752. SLFSSL V4ZQG3 V4ZQG3_TOXGO Uncharacterized protein 1179 858-863
753. LFSSLH A0A023NL40 A0A023NL40_TOXGO RNA polymerase B 420 56-61
754. LFSSLH A0A023NLF5 A0A023NLF5_TOXGO RNA polymerase B 420 56-61
755. LFSSLH A0A023NM35 A0A023NM35_TOXGO RNA polymerase B 420 56-61
756. LFSSLH Q9MTD3 RPOB_TOXGO DNA-directed RNA polymerase subunit beta 1051 652-657
757. LFSSLH V4Z7Z2 V4Z7Z2_TOXGO DEAD/DEAH box helicase domain-containing protein 911 493-498
758. LFSSLH V5B6U5 V5B6U5_TOXGO Putative transmembrane protein 226 146-151
759. FSSLHS B9QPQ1 B9QPQ1_TOXGO DNA polymerase epsilon subunit B protein 864 107-112
760. FSSLHS B9QPU3 B9QPU3_TOXGO Putative RNase protein H 1344 210-215
761. FSSLHS V4ZGZ6 V4ZGZ6_TOXGO AP2 domain transcription factor AP2VIIa-5 2597 324-329
762. FSSLHS V4ZJU9 V4ZJU9_TOXGO Uncharacterized protein 299 46-51
763. SSLHSS B6KPB1 B6KPB1_TOXGO Putative transmembrane protein 1018 391-396
764. SSLHSS B6KRA7 B6KRA7_TOXGO ATP-dependent DNA helicase, RecQ family protein 1626 1411-1416
765. SSLHSS B9QPU3 B9QPU3_TOXGO Putative RNase protein H 1344 211-216
766. SSLHSS Q1JSK0 Q1JSK0_TOXGO Uncharacterized protein 1085 458-463
767. SSLHSS V4YR59 V4YR59_TOXGO Uncharacterized protein 1342 889-894
768. SSLHSS V4YSI1 V4YSI1_TOXGO Thiamin pyrophosphokinase, catalytic domain-containing protein 813 413-418
769. SSLHSS V4YT41 V4YT41_TOXGO Protein kinase domain protein 6052 5423-5428
770. SSLHSS V4Z3V3 V4Z3V3_TOXGO Uncharacterized protein 3415 1369-1374
771. SSLHSS V4Z7A4 V4Z7A4_TOXGO Histone lysine-specific demethylase LSD1/BHC110/KDMA1A 2947 1466-1471, 1581-1586
772. SSLHSS V4ZCD6 V4ZCD6_TOXGO Putative transmembrane protein 628 361-366
773. SSLHSS V4ZGZ6 V4ZGZ6_TOXGO AP2 domain transcription factor AP2VIIa-5 2597 325-330
774. SSLHSS V4ZHM6 V4ZHM6_TOXGO Uncharacterized protein 2986 714-719
775. SSLHSS V4ZJS7 V4ZJS7_TOXGO Uncharacterized protein 1111 308-313
776. SLHSSN V4Z7N1 V4Z7N1_TOXGO Uncharacterized protein 673 549-554
777. IDLLEK B9QL21 B9QL21_TOXGO Putative rRNA methyltransferase 981 482-487
778. IDLLEK Q1JST4 Q1JST4_TOXGO Putative rRNA methyltransferase 981 482-487
779. LLEKIA V5BA46 V5BA46_TOXGO Uncharacterized protein 2092 51-56
780. WTGLVG V4ZD56 V4ZD56_TOXGO Uncharacterized protein 152 147-152
781. LVGDLL B9PTE8 B9PTE8_TOXGO Clathrin heavy chain 1731 1587-1592
782. LVGDLL B9QJN6 B9QJN6_TOXGO Transcription initiation factor TFIID complex subunit TAF12 569 70-75
783. LVGDLL Q1JSF3 Q1JSF3_TOXGO Uncharacterized protein 4600 756-761
784. LVGDLL V4YZC8 V4YZC8_TOXGO WD repeat domain 35 family protein 1334 967-972
785. LVGDLL V4Z6H5 V4Z6H5_TOXGO Putative glutamic acid-rcih protein 4436 294-299
786. LVGDLL V4ZUD7 V4ZUD7_TOXGO ATPase family associated with various cellular activities (AAA) domain-containing protein 4901 4619-4624
787. VGDLLR B9PTE8 B9PTE8_TOXGO Clathrin heavy chain 1731 1588-1593
788. GDLLRG V4ZJW7 V4ZJW7_TOXGO Sma protein 2746 1783-1788
789. GDLLRG V4ZSE5 V4ZSE5_TOXGO Uncharacterized protein 2061 980-985
790. LLRGTA Q1JT06 Q1JT06_TOXGO Uncharacterized protein 1979 372-377
791. LLRGTA V4YW09 V4YW09_TOXGO PHD-finger domain-containing protein 5658 5460-5465
792. SFSINT Q1JSY5 Q1JSY5_TOXGO Uncharacterized protein 1811 729-734
793. SFSINT V4Z1R2 V4Z1R2_TOXGO GCC2 and GCC3 domain-containing protein 5081 715-720
794. SQVIDF V5BC14 V5BC14_TOXGO Putative transmembrane protein 1041 985-990
795. FTSPFF V4Z3T6 V4Z3T6_TOXGO Uncharacterized protein 1806 151-156
796. TSPFFS V4Z3T6 V4Z3T6_TOXGO Uncharacterized protein 1806 152-157
797. TSPFFS V4ZEN8 V4ZEN8_TOXGO Uncharacterized protein 4618 501-506
798. TSPFFS V4ZHV0 V4ZHV0_TOXGO Uncharacterized protein 815 360-365
799. TSPFFS V4ZL97 V4ZL97_TOXGO Uncharacterized protein 7954 1452-1457
800. SPFFST V4YZ55 V4YZ55_TOXGO DNA repair protein Rad4 domain-containing protein 1935 1069-1074
801. PFFSTS B9QQJ8 B9QQJ8_TOXGO Uncharacterized protein 1182 1115-1120
802. PFFSTS Q1JSC7 Q1JSC7_TOXGO Uncharacterized protein 1182 1115-1120
803. PFFSTS V4Z7J7 V4Z7J7_TOXGO Uncharacterized protein 1162 102-107
804. FFSTSL V4Z255 V4Z255_TOXGO Thioredoxin domain-containing protein 490 188-193
805. FSTSLG V5B6P1 V5B6P1_TOXGO Putative galactosyltransferase 441 306-311
806. SLGILV B9QDJ4 B9QDJ4_TOXGO Nudix-type motif 9 isoform a family protein 546 49-54
807. SLGILV V4ZMY1 V4ZMY1_TOXGO Flagellar/basal body protein 2758 232-237
808. GILVRT B9Q0H3 B9Q0H3_TOXGO RNA methyltransferase, TrmH family protein 840 670-675
809. ILVRTR V4Z445 V4Z445_TOXGO Histone acetyltransferase TAF1/250 2775 1785-1790
810. RDTAAP V4ZLJ6 V4ZLJ6_TOXGO MIZ/SP-RING zinc finger domain-containing protein 1779 889-894
811. DTAAPI B9QIV8 B9QIV8_TOXGO Rhoptry kinase family protein 377 247-252
812. TAAPIG B9QIV8 B9QIV8_TOXGO Rhoptry kinase family protein 377 248-253
813. ALHITA V4Z263 V4Z263_TOXGO Glutaredoxin domain-containing protein 312 43-48
814. ITAVFL B9Q4E9 B9Q4E9_TOXGO Putative transmembrane protein 305 67-72
815. TAVFLT B9Q4E9 B9Q4E9_TOXGO Putative transmembrane protein 305 68-73
816. SPFGLT V4ZGU1 V4ZGU1_TOXGO Uncharacterized protein 2180 623-628
817. RNRSKV V4YU76 V4YU76_TOXGO Tubulin-tyrosine ligase family protein 2376 1425-1430
818. KVFSFS A4UQJ4 A4UQJ4_TOXGO Isocitrate dehydrogenase 2 621 339-344
819. KVFSFS V5BIR7 V5BIR7_TOXGO Isocitrate dehydrogenase 621 339-344
820. VFSFSS B9PGL7 B9PGL7_TOXGO Putative transmembrane protein 745 20-25
821. VFSFSS B9QE58 B9QE58_TOXGO Putative proteasome subunit beta type 1 361 98-103
822. VFSFSS V4ZGQ4 V4ZGQ4_TOXGO Uncharacterized protein 2272 996-1001
823. FSFSSA V4YRN9 V4YRN9_TOXGO Carrier superfamily protein 555 351-356
824. FSFSSA V4Z2M0 V4Z2M0_TOXGO Uncharacterized protein 2289 1752-1757
825. FSFSSA V4ZP08 V4ZP08_TOXGO Uncharacterized protein 3263 26-31
826. FSFSSA V5B5Z7 V5B5Z7_TOXGO Putative transmembrane protein 1546 578-583
827. FSFSSA V5BCB0 V5BCB0_TOXGO 4'-phosphopantetheinyl transferase domain-containing protein 1135 518-523
828. FSFSSA V5BM94 V5BM94_TOXGO Uncharacterized protein 3520 206-211
829. SFSSAL B9Q3Z1 B9Q3Z1_TOXGO Uncharacterized protein 559 187-192
830. SFSSAL B9Q8I6 B9Q8I6_TOXGO Putative POP4 domain protein 501 377-382
831. SFSSAL V4YZD3 V4YZD3_TOXGO DnaJ domain-containing protein 651 256-261
832. SFSSAL V4Z3C9 V4Z3C9_TOXGO Uncharacterized protein 1899 380-385
833. SFSSAL V4ZI73 V4ZI73_TOXGO Uncharacterized protein 4519 448-453
834. SFSSAL V4ZLH0 V4ZLH0_TOXGO Putative transmembrane protein 1257 462-467
835. SSALNI B9QGN4 B9QGN4_TOXGO Uncharacterized protein 2182 848-853
836. SALNIC B9QGN4 B9QGN4_TOXGO Uncharacterized protein 2182 849-854
837. YALLFG B9Q7G7 B9Q7G7_TOXGO tRNA pseudouridine synthase 904 177-182
838. ALLFGR V4ZJW0 V4ZJW0_TOXGO Exostosin family protein 1327 1005-1010
839. LLFGRT Q1PCQ8 Q1PCQ8_TOXGO Mitochondrial type I phosphatidylserine decarboxylase 427 131-136
840. LLFGRT V4ZB70 V4ZB70_TOXGO Phosphatidylserine decarboxylase 427 131-136
841. STYTAN V4ZE87 V4ZE87_TOXGO Uncharacterized protein 461 361-366
842. ANLAAV V4YK78 V4YK78_TOXGO Uncharacterized protein 4948 4557-4562
843. ANLAAV V4Z0B6 V4Z0B6_TOXGO Tetratricopeptide repeat-containing protein 1383 1060-1065
844. ANLAAV V4Z819 V4Z819_TOXGO DEAD/DEAH box helicase domain-containing protein 1850 735-740
845. EELSGI V4YYW3 V4YYW3_TOXGO Uncharacterized protein 642 440-445
846. EELSGI V4ZBL9 V4ZBL9_TOXGO Uncharacterized protein 2020 1069-1074
847. KLHHPS V4Z8L2 V4Z8L2_TOXGO Uncharacterized protein 4801 1777-1782
848. PSQGFR V4ZEF2 V4ZEF2_TOXGO AP2 domain transcription factor AP2IX-3 3096 552-557
849. GTVRES V4ZE39 V4ZE39_TOXGO Putative U5 small nuclear ribonuclear protein 257 48-53
850. TVRESS B9Q851 B9Q851_TOXGO SSXT (Amine-terminal region) protein 826 51-56
851. TVRESS V4ZAE2 V4ZAE2_TOXGO Bromodomain-containing protein 1827 1689-1694
852. TVRESS V4ZGN7 V4ZGN7_TOXGO Zinc finger, C3HC4 type (RING finger) domain-containing protein 1105 960-965
853. VRESSA B9QF24 B9QF24_TOXGO Dihydrolipoyl dehydrogenase 519 292-297
854. VRESSA Q1KSF4 Q1KSF4_TOXGO Dihydrolipoyl dehydrogenase 519 292-297
855. ESSAED B6K915 B6K915_TOXGO WD domain, G-beta repeat-containing protein 793 259-264
856. ESSAED B9QL14 B9QL14_TOXGO Inositol polyphosphate kinase 2851 239-244
857. ESSAED V4Z4P9 V4Z4P9_TOXGO Uncharacterized protein 2174 652-657
858. FPEMHE B6DST1 B6DST1_TOXGO MutS-like protein 2163 1848-1853
859. FPEMHE V4Z4E7 V4Z4E7_TOXGO MutS domain protein 2163 1848-1853
860. EYMRRY B6KJJ3 B6KJJ3_TOXGO Beta-lactamase superfamily domain protein 662 548-553
861. PATPDG B9QR14 B9QR14_TOXGO Zinc finger, C3HC4 type (RING finger) domain-containing protein 971 299-304
862. ATPDGV B9Q7B5 B9Q7B5_TOXGO TLD protein 2195 437-442
863. LLTVGK V4ZW65 V4ZW65_TOXGO Uncharacterized protein 1272 1244-1249
864. PFAIEG B9QH65 B9QH65_TOXGO Autophagy protein Apg6 644 592-597
865. GIGLPP B9QR24 B9QR24_TOXGO Prostaglandin-E synthase 540 160-165
866. GLPPNS B9QRA2 B9QRA2_TOXGO GYF domain-containing protein 2331 1208-1213
867. GLPPNS V4ZIB0 V4ZIB0_TOXGO Uncharacterized protein 209 203-208
868. LPPNSP B6KAG9 B6KAG9_TOXGO U3 small nucleolar RNA-associated protein 10 3738 167-172
869. LPPNSP V4ZM28 V4ZM28_TOXGO Uncharacterized protein 1190 41-46
870. LPPNSP V5BGF9 V5BGF9_TOXGO Rhoptry kinase family protein 510 282-287
871. SPLTAN Q1JT54 Q1JT54_TOXGO Putative uncharacterized protein 1990 1697-1702
872. SPLTAN V4Z9Y8 V4Z9Y8_TOXGO Uncharacterized protein 2154 1697-1702
873. LISQYK B9PIK4 B9PIK4_TOXGO Uncharacterized protein 274 217-222
874. GKRSFA V4ZDZ3 V4ZDZ3_TOXGO Uncharacterized protein 2127 1719-1724
875. GKRSFA V4ZFI3 V4ZFI3_TOXGO Uncharacterized protein 3747 964-969
876. KRSFAV V4ZFI3 V4ZFI3_TOXGO Uncharacterized protein 3747 965-970
877. RSFAVT V4Z7F3 V4Z7F3_TOXGO TBC domain-containing protein 2116 2076-2081
878. SFAVTE B2D1U3 B2D1U3_TOXGO MIC3 protein 359 55-60
879. SFAVTE G0ZSE8 G0ZSE8_TOXGO Microneme protein MIC3 344 46-51
880. SFAVTE Q9GRG4 Q9GRG4_TOXGO MIC3 microneme protein precursor 359 55-60
881. SFAVTE T2FG71 T2FG71_TOXGO Microneme protein 3 333 30-35
882. SFAVTE V4YND8 V4YND8_TOXGO Microneme protein MIC3 383 55-60
883. FAVTET B2D1U3 B2D1U3_TOXGO MIC3 protein 359 56-61
884. FAVTET G0ZSE8 G0ZSE8_TOXGO Microneme protein MIC3 344 47-52
885. FAVTET Q9GRG4 Q9GRG4_TOXGO MIC3 microneme protein precursor 359 56-61
886. FAVTET T2FG71 T2FG71_TOXGO Microneme protein 3 333 31-36
887. FAVTET V4YND8 V4YND8_TOXGO Microneme protein MIC3 383 56-61
888. AVTETL V4ZK62 V4ZK62_TOXGO Putative transmembrane protein 905 829-834
889. HFSGLF V4YU85 V4YU85_TOXGO DNA-directed RNA polymerase 2768 838-843
890. GLFVLL V4ZGI1 V4ZGI1_TOXGO Calcium-dependent protein kinase 2228 1872-1877
891. GLFVLL V4ZHU0 V4ZHU0_TOXGO ATPase, AAA family protein 1031 59-64
892. GLFVLL V5BGB9 V5BGB9_TOXGO SNARE associated Golgi protein 394 167-172
893. LFVLLC V4ZDJ4 V4ZDJ4_TOXGO POPLD (NUC188) domain-containing protein 1757 1001-1006
894. VLLCIG V4ZPQ2 V4ZPQ2_TOXGO Putative transmembrane protein 378 13-18
895. VYRLLL B9QJX3 B9QJX3_TOXGO Uncharacterized protein 4533 4463-4468
896. VYRLLL B9QMH6 B9QMH6_TOXGO RNA pseudouridine synthase superfamily protein 6535 1190-1195
897. VYRLLL V4ZBY1 V4ZBY1_TOXGO Putative transmembrane protein 3864 1586-1591
898. VYRLLL V5BJC6 V5BJC6_TOXGO EF hand domain-containing protein 6368 445-450
899. RLLLPR Q1JSM5 Q1JSM5_TOXGO Ubiquitin-transferase, putative precursor 12269 12226-12231
900. RLLLPR V4YRP3 V4YRP3_TOXGO Uncharacterized protein 374 173-178
901. RLLLPR V4Z1Z4 V4Z1Z4_TOXGO HECT-domain (Ubiquitin-transferase) domain-containing protein 12299 12256-12261
902. RLLLPR V4ZA53 V4ZA53_TOXGO Uncharacterized protein 2160 987-992
903. IKNKSK Q9MTD8 Q9MTD8_TOXGO Ribosomal protein S7 136 95-100
904. RLHRAI B6KLP1 RON22_TOXGO Rhoptry neck protein 2-like protein 2 precursor 1167 668-673
905. RLHRAI V4ZEK4 V4ZEK4_TOXGO Putative rhoptry neck protein 1167 668-673
906. EEKQQH V4ZJ15 V4ZJ15_TOXGO Uncharacterized protein 118 32-37
907. KRVEKR V4ZQN2 V4ZQN2_TOXGO Putative cell-cycle-associated protein kinase CDK 1903 855-860
908. VEKRSN Q1JT11 Q1JT11_TOXGO Putative uncharacterized protein 488 237-242
909. IFSDEE B9QNA3 B9QNA3_TOXGO Peptidase M16 inactive domain-containing protein 1353 1172-1177
910. IFSDEE V4YXS2 V4YXS2_TOXGO Uncharacterized protein 201 147-152
911. FSDEEG B9Q850 B9Q850_TOXGO Uncharacterized protein 2068 1402-1407
912. SDEEGQ V4Z1B1 V4Z1B1_TOXGO Phospholipid-translocating P-type ATPase, flippase subfamily protein 2427 1018-1023
913. LGIRIH V4ZV07 V4ZV07_TOXGO Uncharacterized protein 432 218-223
914. GIRIHQ V4ZV07 V4ZV07_TOXGO Uncharacterized protein 432 219-224
915. IRIHQD V4ZV07 V4ZV07_TOXGO Uncharacterized protein 432 220-225
916. DIPLPP B6KGT6 B6KGT6_TOXGO Uncharacterized protein 502 238-243
917. IPLPPR V4ZRY6 V4ZRY6_TOXGO Calcium binding egf domain-containing protein 3105 3002-3007
918. IPLPPR V5BE95 V5BE95_TOXGO Serine/threonine specific protein phosphatase 1265 807-812
919. PLPPRR B6KFR4 B6KFR4_TOXGO Putative trichohyalin 3900 155-160
920. PLPPRR B6KGK0 B6KGK0_TOXGO Uncharacterized protein 421 147-152
921. PLPPRR B9QCB5 B9QCB5_TOXGO Uncharacterized protein 413 140-145
922. PLPPRR Q1JT25 Q1JT25_TOXGO Putative uncharacterized protein 1257 707-712
923. PLPPRR V4Z9J2 V4Z9J2_TOXGO Uncharacterized protein 1923 1520-1525
924. PLPPRR V4Z9V6 V4Z9V6_TOXGO Uncharacterized protein 1571 1021-1026
925. PLPPRR V4ZRS2 V4ZRS2_TOXGO Uncharacterized protein 1304 796-801
926. PLPPRR V4ZRY6 V4ZRY6_TOXGO Calcium binding egf domain-containing protein 3105 3003-3008
927. LPPRRR Q1JT10 Q1JT10_TOXGO Phosphodiesterase, putative 2092 4-9 125705
928. LPPRRR V4Z1J2 V4Z1J2_TOXGO 3'5'-cyclic nucleotide phosphodiesterase domain-containing protein 2238 4-9
929. PPRRRE B9QMG4 B9QMG4_TOXGO Putative transmembrane protein 1215 406-411
930. PPRRRE V4Z3Z6 V4Z3Z6_TOXGO Amine-terminal region of chorein, A TM vesicle-mediated sorter 2204 437-442
931. RRRELP B9PYE4 B9PYE4_TOXGO Putative type I fatty acid synthase 10021 5614-5619
932. RRRELP B9Q591 B9Q591_TOXGO Putative transmembrane protein 7354 3699-3704
933. RRRELP Q1JTE1 Q1JTE1_TOXGO Type I fatty acid synthase, putative 9940 5533-5538
934. RRRELP V4Z3T2 V4Z3T2_TOXGO Uncharacterized protein 2075 1986-1991
935. RRRELP V4Z4C0 V4Z4C0_TOXGO Uncharacterized protein 2996 2750-2755
936. RRRELP V4ZGQ4 V4ZGQ4_TOXGO Uncharacterized protein 2272 1812-1817
937. RRELPA B6KHU0 B6KHU0_TOXGO AP2 domain transcription factor AP2XII-9 1893 1251-1256
938. RRELPA B9PYE4 B9PYE4_TOXGO Putative type I fatty acid synthase 10021 5615-5620
939. RRELPA Q1JTE1 Q1JTE1_TOXGO Type I fatty acid synthase, putative 9940 5534-5539
940. RRELPA V4Z4A1 V4Z4A1_TOXGO WD domain, G-beta repeat-containing protein 1314 795-800
941. RELPAL B9QH93 B9QH93_TOXGO Putative ppg3 1271 331-336
942. RELPAL V4ZBB6 V4ZBB6_TOXGO Uncharacterized protein 3325 3031-3036
943. ELPALR V4ZDK9 V4ZDK9_TOXGO Uncharacterized protein 2679 2249-2254
944. ELPALR V4ZIR0 V4ZIR0_TOXGO Ubiquitin carboxyl-terminal hydrolase 2294 294-299
945. LPALRT V4Z8G3 V4Z8G3_TOXGO RNA-dependent RNA polymerase RDP 2894 517-522
946. PALRTT V4YQT3 V4YQT3_TOXGO Putative transmembrane protein 3329 1653-1658
947. GKADSL B6K8L1 B6K8L1_TOXGO Fibrillarin 304 84-89
948. DSLNVS B9QP13 B9QP13_TOXGO Putative histone deacetylase SIR2 1703 1132-1137
949. QELSEL B9PTP8 B9PTP8_TOXGO WD domain, G-beta repeat-containing protein 530 32-37
950. ELSELE V4Z952 V4Z952_TOXGO Uncharacterized protein 4210 2569-2574
951. LSELEK B9QFW3 B9QFW3_TOXGO AP2 domain transcription factor AP2VIIa-7 3112 2049-2054
952. LSELEK V4YPY6 V4YPY6_TOXGO Uncharacterized protein 2436 423-428
953. LSELEK V4ZR17 V4ZR17_TOXGO Uncharacterized protein 558 386-391
954. SELEKQ B6KHM9 B6KHM9_TOXGO Uncharacterized protein 1509 192-197
955. SELEKQ V4YUS4 V4YUS4_TOXGO Putative enterophilin-2L 1024 483-488
956. ELEKQI B6KAF0 B6KAF0_TOXGO Putative SLU7 splicing factor 544 179-184
957. ELEKQI B6KVW0 B6KVW0_TOXGO Cwf18 pre-mRNA splicing factor protein 152 43-48
958. ELEKQI V4YUS4 V4YUS4_TOXGO Putative enterophilin-2L 1024 484-489
959. ELEKQI V4ZKH8 V4ZKH8_TOXGO Methyltransferase TYW3 793 768-773
960. LEKQIQ B9Q1S5 B9Q1S5_TOXGO Putative 26S protease regulatory subunit 4 441 191-196
961. LEKQIQ V4YR63 V4YR63_TOXGO RAP domain-containing protein 713 455-460
962. LEKQIQ V4ZKH8 V4ZKH8_TOXGO Methyltransferase TYW3 793 769-774
963. QELQLA V4YN30 V4YN30_TOXGO Putative vacuolar protein sorting-associated protein 8650 1412-1417
964. QELQLA V4YVA9 V4YVA9_TOXGO Uncharacterized protein 1694 1231-1236
965. LQLAVS V4Z4S8 V4Z4S8_TOXGO Putative transmembrane protein 351 174-179
966. LQLAVS V4Z9X5 V4Z9X5_TOXGO Uncharacterized protein 4983 2934-2939
967. LQLAVS V4ZT82 V4ZT82_TOXGO Uncharacterized protein 3407 3186-3191
968. QLAVSR V4Z4P9 V4Z4P9_TOXGO Uncharacterized protein 2174 118-123
969. QLAVSR V4Z718 V4Z718_TOXGO Concanavalin A-like lectin/glucanase family protein 1494 1406-1411
970. QLAVSR V4ZFI3 V4ZFI3_TOXGO Uncharacterized protein 3747 684-689
971. QLAVSR V5B2E0 V5B2E0_TOXGO MORN repeat-containing protein 3443 326-331
972. LAVSRK V4Z9Y9 V4Z9Y9_TOXGO Uncharacterized protein 1486 1276-1281
973. LAVSRK V4ZF42 V4ZF42_TOXGO Uncharacterized protein 635 63-68
974. AVSRKT V4ZSE4 V4ZSE4_TOXGO WD domain, G-beta repeat-containing protein 4664 1628-1633
975. VSRKTE V4Z8R9 V4Z8R9_TOXGO Uncharacterized protein 627 176-181
976. LEEYQR B6KGC0 B6KGC0_TOXGO Uncharacterized protein 880 547-552
977. RTSRTC V4ZCK2 V4ZCK2_TOXGO Uncharacterized protein 1430 175-180

**NMDA 3B**

1. VRALWL B6KHB8 B6KHB8_TOXGO Leucine rich repeat-containing protein 354 60-65
2. LWLGLA V4ZBH1 V4ZBH1_TOXGO Uncharacterized protein 3736 2885-2890
3. LGLALA V4ZR91 V4ZR91_TOXGO Uncharacterized protein 3903 798-803
4. LGLALA V4ZUU4 V4ZUU4_TOXGO UBA/TS-N domain-containing protein 1868 1798-1803
5. GLALAL B6K9K0 B6K9K0_TOXGO Putative saccharopine dehydrogenase 537 492-497
6. GLALAL V4ZUU4 V4ZUU4_TOXGO UBA/TS-N domain-containing protein 1868 1799-1804
7. GLALAL V5BEU8 V5BEU8_TOXGO Myosin head (Motor domain) domain-containing protein 2484 1665-1670
8. LALALG V5BAH3 V5BAH3_TOXGO Metal cation transporter, ZIP family protein 717 549-554
9. ALGPGS V5AY44 V5AY44_TOXGO Putative transmembrane protein 7450 4663-4668
10. LGPGSA V4YZY7 V4YZY7_TOXGO ATP-dependent DNA helicase, RecQ family protein 1759 1558-1563
11. LGPGSA V5AY44 V5AY44_TOXGO Putative transmembrane protein 7450 4664-4669
12. LGPGSA V5BDZ6 V5BDZ6_TOXGO Uncharacterized protein 4591 2768-2773
13. GPGSAG V4YT75 V4YT75_TOXGO Toxoplasma gondii family E protein 1345 733-738
14. GPGSAG V4YTC4 V4YTC4_TOXGO Toxoplasma gondii family E protein 1432 794-799
15. GPGSAG V4YXW0 V4YXW0_TOXGO Toxoplasma gondii family E protein 596 354-359
16. GPGSAG V4Z7M7 V4Z7M7_TOXGO Transcription factor/nuclear export subunit 2 3000 777-782
17. GPGSAG V4Z7S6 V4Z7S6_TOXGO Toxoplasma gondii family E protein 683 39-44
18. GPGSAG V4ZBV6 V4ZBV6_TOXGO Toxoplasma gondii family E protein 642 5-10
19. GPGSAG V4ZBZ6 V4ZBZ6_TOXGO Toxoplasma gondii family E protein 429 335-340
20. GPGSAG V4ZGB3 V4ZGB3_TOXGO Toxoplasma gondii family E protein 1015 373-378
21. GPGSAG V5AZC7 V5AZC7_TOXGO Corepressor complex CRC230 1916 477-482
22. GPGSAG V5B6W4 V5B6W4_TOXGO Toxoplasma gondii family E protein 926 806-811
23. GPGSAG V5B6X0 V5B6X0_TOXGO Toxoplasma gondii family E protein 851 825-830
24. GPGSAG V5BDZ6 V5BDZ6_TOXGO Uncharacterized protein 4591 2769-2774
25. PGSAGG B6KB04 B6KB04_TOXGO Uncharacterized protein 621 314-319
26. PGSAGG B9Q792 B9Q792_TOXGO Uncharacterized protein 4118 2867-2872
27. PGSAGG H3K408 H3K408_TOXGO CaMK-related kinase 3196 812-817
28. PGSAGG V4Z6H7 V4Z6H7_TOXGO UvrD/REP helicase domain-containing protein 3190 729-734
29. PGSAGG V4Z7M7 V4Z7M7_TOXGO Transcription factor/nuclear export subunit 2 3000 778-783
30. PGSAGG V5BC38 V5BC38_TOXGO Uncharacterized protein 1048 666-671
31. GSAGGH B6KA89 B6KA89_TOXGO Putative transmembrane protein 788 311-316
32. GSAGGH V4Z7U1 V4Z7U1_TOXGO HECT-domain (Ubiquitin-transferase) domain-containing protein 1978 983-988
33. SAGGHP B6KA89 B6KA89_TOXGO Putative transmembrane protein 788 312-317
34. GVLARL V4ZMG2 V4ZMG2_TOXGO AP2 domain transcription factor AP2XI-3 1399 267-272
35. GVLARL V5B7S3 V5B7S3_TOXGO Putative PX domain protein 1765 494-499
36. VLARLG B6KH76 B6KH76_TOXGO Uncharacterized protein 283 24-29
37. VLARLG B9QB74 B9QB74_TOXGO NOL1/NOP2/Sun family protein 919 649-654
38. VLARLG V4ZB28 V4ZB28_TOXGO Uncharacterized protein 1836 749-754
39. VLARLG V5B4Q9 V5B4Q9_TOXGO WD domain, G-beta repeat-containing protein 3086 2477-2482
40. VLARLG V5BKB4 V5BKB4_TOXGO START domain-containing protein 857 256-261
41. LARLGG B6KH76 B6KH76_TOXGO Uncharacterized protein 283 25-30
42. LARLGG B6KHT7 B6KHT7_TOXGO Putative seryl-tRNA synthetase, cytoplasmic 482 156-161
43. LARLGG B9QB74 B9QB74_TOXGO NOL1/NOP2/Sun family protein 919 650-655
44. LARLGG V4Z4Z7 V4Z4Z7_TOXGO Putative raffinose synthase 1 1483 350-355
45. ARLGGS Q1JT96 Q1JT96_TOXGO Putative uncharacterized protein precursor 1412 88-93
46. ARLGGS V4YLT3 V4YLT3_TOXGO Putative transmembrane protein 1521 88-93
47. ARLGGS V4ZC14 V4ZC14_TOXGO Uncharacterized protein 1420 498-503
48. GGSVRL B6KGB1 B6KGB1_TOXGO DNA-directed RNA polymerase II RPB3 484 476-481
49. GGSVRL V4YZ80 V4YZ80_TOXGO HECT-domain (Ubiquitin-transferase) domain-containing protein 15897 2127-2132
50. GGSVRL V4Z9J2 V4Z9J2_TOXGO Uncharacterized protein 1923 1889-1894
51. SVRLGA B9QI34 B9QI34_TOXGO Helicase associated domain (Ha2) protein 2234 1732-1737
52. SVRLGA Q1JT36 Q1JT36_TOXGO Putative uncharacterized protein 396 390-395
53. SVRLGA V4YPA0 V4YPA0_TOXGO RNA pseudouridine synthase 1269 91-96
54. SVRLGA V4Z3R9 V4Z3R9_TOXGO Uncharacterized protein 150 84-89
55. SVRLGA V4Z5L5 V4Z5L5_TOXGO Uncharacterized protein 622 307-312
56. VRLGAL V4Z8B4 V4Z8B4_TOXGO Putative thioredoxin 711 87-92
57. VRLGAL V4ZRA3 V4ZRA3_TOXGO Putative transmembrane protein 1026 301-306
58. RLGALL B9QNS1 B9QNS1_TOXGO tRNA pseudouridine synthase 1818 961-966
59. RLGALL V4YY31 V4YY31_TOXGO Uncharacterized protein 371 355-360
60. RLGALL V4Z8B4 V4Z8B4_TOXGO Putative thioredoxin 711 88-93
61. LGALLP B9Q0M5 B9Q0M5_TOXGO Putative trichohyalin 1427 712-717
62. LGALLP V4YY31 V4YY31_TOXGO Uncharacterized protein 371 356-361
63. LGALLP V4ZAX6 V4ZAX6_TOXGO Uncharacterized protein 8643 1062-1067
64. LGALLP V4ZBC0 V4ZBC0_TOXGO SCP family extracellular subfamily protein 820 516-521
65. LGALLP V4ZLY5 V4ZLY5_TOXGO Uncharacterized protein 1281 606-611
66. LGALLP V5B535 V5B535_TOXGO ImpB/MucB/SamB family protein 1253 326-331
67. LGALLP V5B7D1 V5B7D1_TOXGO Uncharacterized protein 3118 418-423
68. LGALLP V5B9N6 V5B9N6_TOXGO Nucleolar GTP-binding protein 1 922 252-257
69. GALLPR V4YRP9 V4YRP9_TOXGO Peptidase M16 inactive domain-containing protein 1692 760-765
70. GALLPR V4ZVI6 V4ZVI6_TOXGO SufB/sufD domain-containing protein 1860 674-679
71. GALLPR V5B9N6 V5B9N6_TOXGO Nucleolar GTP-binding protein 1 922 253-258
72. ALLPRA B6KRX4 B6KRX4_TOXGO HECT-domain (Ubiquitin-transferase) domain-containing protein 1288 1245-1250
73. ALLPRA V4ZFS3 V4ZFS3_TOXGO Putative transmembrane protein 1541 1472-1477
74. ALLPRA V5B8L5 V5B8L5_TOXGO IQ calmodulin-binding motif domain-containing protein 1179 351-356
75. LLPRAP V4YTJ8 V4YTJ8_TOXGO Uncharacterized protein 1357 144-149
76. LLPRAP V4ZES6 V4ZES6_TOXGO Transporter, small conductance mechanosensitive ion channel protein 3400 2622-2627
77. LPRAPL B6K9R7 B6K9R7_TOXGO Dynactin subunit 4 689 25-30
78. LPRAPL V4Z4I3 V4Z4I3_TOXGO Peptidyl-tRNA hydrolase PTH2 domain-containing protein 266 30-35
79. PRAPLA Q6JD66 Q6JD66_TOXGO Eukaryotic initiation factor-2 alpha kinase-A 5072 2022-2027, 2979-2984
80. PRAPLA V4ZU83 V4ZU83_TOXGO eIF2 kinase IF2K-A (Incomplete catalytic triad) 4638 1588-1593, 2545-2550
81. RAPLAR Q6JD66 Q6JD66_TOXGO Eukaryotic initiation factor-2 alpha kinase-A 5072 2023-2028
82. RAPLAR V4ZU83 V4ZU83_TOXGO eIF2 kinase IF2K-A (Incomplete catalytic triad) 4638 1589-1594
83. APLARA Q1JTI3 Q1JTI3_TOXGO Ubiquitin-protein ligase 1, putative 8112 5785-5790
84. APLARA V4Z553 V4Z553_TOXGO HECT-domain (Ubiquitin-transferase) domain-containing protein 8007 5787-5792
85. APLARA V4ZI56 V4ZI56_TOXGO Uncharacterized protein 2083 906-911
86. PLARAR B6K9V5 B6K9V5_TOXGO Uncharacterized protein 1315 1157-1162
87. PLARAR V4Z303 V4Z303_TOXGO DnaJ domain-containing protein 2010 1168-1173
88. PLARAR V4ZJF5 V4ZJF5_TOXGO Calcium-dependent protein kinase CDPK5 682 668-673
89. LARARA B6K9V5 B6K9V5_TOXGO Uncharacterized protein 1315 1158-1163
90. LARARA V4YZY7 V4YZY7_TOXGO ATP-dependent DNA helicase, RecQ family protein 1759 439-444
91. LARARA V4ZFB3 V4ZFB3_TOXGO Uncharacterized protein 447 246-251
92. LARARA V4ZJF5 V4ZJF5_TOXGO Calcium-dependent protein kinase CDPK5 682 669-674
93. LARARA V4ZLU9 V4ZLU9_TOXGO Uncharacterized protein 1026 676-681
94. ARARAR Q1JTJ3 Q1JTJ3_TOXGO SET-domain protein, putative 4382 3740-3745
95. ARARAR V4Z559 V4Z559_TOXGO Putative histone lysine methyltransferase, SET 5175 4397-4402
96. ARARAR V5BMD9 V5BMD9_TOXGO UBA/TS-N domain-containing protein 7817 5306-5311
97. RARARA B9Q4E9 B9Q4E9_TOXGO Putative transmembrane protein 305 265-270
98. RARARA V4Z9U0 V4Z9U0_TOXGO Uncharacterized protein 5083 898-903
99. RARARA V5BMD9 V5BMD9_TOXGO UBA/TS-N domain-containing protein 7817 5305-5310
100. ARARAA B9Q560 B9Q560_TOXGO Transport protein Trs120 2958 181-186
101. ARARAA B9QBS0 B9QBS0_TOXGO WD domain, G-beta repeat-containing protein 1017 29-34
102. ARARAA B9QEH5 B9QEH5_TOXGO GCC2 and GCC3 domain-containing protein 7428 7069-7074
103. ARARAA V4Z399 V4Z399_TOXGO Uncharacterized protein 612 317-322
104. ARARAA V4Z3P9 V4Z3P9_TOXGO Putative Tbc domain,related protein 3378 1423-1428
105. ARARAA V4Z6Z5 V4Z6Z5_TOXGO Uncharacterized protein 1551 727-732
106. ARARAA V4ZAY6 V4ZAY6_TOXGO Spc97 / Spc98 family protein 2023 1995-2000
107. ARARAA V4ZEW1 V4ZEW1_TOXGO Uncharacterized protein 131 32-37
108. RARAAL B9QAM8 B9QAM8_TOXGO Uncharacterized protein 2194 521-526
109. RARAAL V4Z3N7 V4Z3N7_TOXGO Alpha/beta hydrolase family protein 1235 1102-1107
110. RARAAL V4Z9U4 V4Z9U4_TOXGO Surface antigen repeat-containing protein 6661 4908-4913
111. RARAAL V4ZAY6 V4ZAY6_TOXGO Spc97 / Spc98 family protein 2023 1996-2001
112. ARAALA B6KB56 B6KB56_TOXGO ATP-dependent (S)-NAD(P)H-hydrate dehydratase 508 369-374
113. ARAALA B6KDN0 B6KDN0_TOXGO Eukaryotic translation initiation factor 3 subunit E 601 234-239
114. ARAALA B6KT39 B6KT39_TOXGO EF hand domain-containing protein 3700 205-210
115. ARAALA B9QQ97 B9QQ97_TOXGO Uncharacterized protein 1284 646-651
116. ARAALA V4YS99 V4YS99_TOXGO Putative Myb-like DNA-binding domain protein 1147 727-732
117. ARAALA V4YUW1 V4YUW1_TOXGO Uncharacterized protein 936 470-475
118. ARAALA V4ZJ24 V4ZJ24_TOXGO Phospholipase, patatin family protein 994 280-285
119. ARAALA V5B196 V5B196_TOXGO Uncharacterized protein 2539 1941-1946
120. ARAALA V5B694 V5B694_TOXGO TPR repeat-containing protein 586 320-325
121. ARAALA V5BGJ9 V5BGJ9_TOXGO Uncharacterized protein 818 448-453
122. RAALAR B6KGN6 B6KGN6_TOXGO Alpha/beta hydrolase family protein 1012 567-572
123. RAALAR B9QH52 B9QH52_TOXGO Putative transmembrane protein 574 262-267
124. RAALAR B9QNX5 B9QNX5_TOXGO Nop53 (60S ribosomal biogenesis) protein 599 468-473
125. RAALAR V5BGJ9 V5BGJ9_TOXGO Uncharacterized protein 818 449-454
126. AALARA B6KTZ2 B6KTZ2_TOXGO Uncharacterized protein 1589 251-256
127. AALARA B9QFB6 B9QFB6_TOXGO PGAP1 family protein 2110 487-492
128. AALARA V4Z8R0 V4Z8R0_TOXGO PIK3R4 kinase-related protein 1269 543-548
129. AALARA V4ZED0 V4ZED0_TOXGO Thioredoxin domain protein 1014 253-258
130. AALARA V4ZFB3 V4ZFB3_TOXGO Uncharacterized protein 447 244-249
131. ALARAA B6KA05 B6KA05_TOXGO Transducin beta-like protein TBL1 794 262-267
132. ALARAA B6KRY9 B6KRY9_TOXGO Putative transmembrane protein 1246 329-334
133. ALARAA Q1JSM4 Q1JSM4_TOXGO Elongation factor Ts, mitochondrial precursor 466 151-156
134. ALARAA Q1JT96 Q1JT96_TOXGO Putative uncharacterized protein precursor 1412 8-13
135. ALARAA Q2Y2Q9 Q2Y2Q9_TOXGO Transducin beta-like protein 1 794 262-267
136. ALARAA V4YLT3 V4YLT3_TOXGO Putative transmembrane protein 1521 8-13
137. ALARAA V4YZ78 V4YZ78_TOXGO Pre-RNA processing PIH1/Nop17 protein 830 395-400
138. ALARAA V4Z682 V4Z682_TOXGO Elongation factor Ts, mitochondrial 518 203-208
139. ALARAA V4ZFD6 V4ZFD6_TOXGO Uncharacterized protein 1385 933-938
140. ALARAA V4ZUU4 V4ZUU4_TOXGO UBA/TS-N domain-containing protein 1868 1803-1808
141. ALARAA V5B7V9 V5B7V9_TOXGO L1P family of ribosomal protein 928 457-462
142. LARAAL B6KT39 B6KT39_TOXGO EF hand domain-containing protein 3700 204-209
143. LARAAL V4Z6D0 V4Z6D0_TOXGO Glucosamine-fructose-6-phosphate aminotransferase 898 238-243
144. RAALAP V4ZGI1 V4ZGI1_TOXGO Calcium-dependent protein kinase 2228 1000-1005
145. RAALAP V4ZQT3 V4ZQT3_TOXGO Uncharacterized protein 224 147-152
146. RAALAP V5B9M0 V5B9M0_TOXGO Putative vacuolar ATP synthase subunit d 396 287-292
147. ALAPRL B6KAF6 B6KAF6_TOXGO AP2 domain transcription factor AP2XI-2 2243 1607-1612
148. ALAPRL B9QFH6 B9QFH6_TOXGO Myb family DNA-binding domain-containing protein 2519 1095-1100
149. ALAPRL V4Z4C0 V4Z4C0_TOXGO Uncharacterized protein 2996 1072-1077
150. ALAPRL V4Z7H9 V4Z7H9_TOXGO Ion channel protein 2540 1394-1399
151. LAPRLP V4ZKH5 V4ZKH5_TOXGO Putative ATP-dependent DNA helicase II, 70 kDa subunit 1102 133-138
152. APRLPH B6KAP5 B6KAP5_TOXGO Ankyrin repeat-containing protein 1158 772-777
153. APRLPH V4YWZ4 V4YWZ4_TOXGO Putative transmembrane protein 181 163-168
154. HNLSLE V4ZHG0 V4ZHG0_TOXGO GDA1/CD39 (Nucleoside phosphatase) family protein 696 691-696
155. ELVVAA B6KJF8 B6KJF8_TOXGO Uncharacterized protein 1963 1559-1564
156. ELVVAA V4Z5P5 V4Z5P5_TOXGO Uncharacterized protein 5771 1006-1011
157. VVAAPP Q9U754 Q9U754_TOXGO Acetyl-CoA carboxylase 2 1102 497-502
158. VVAAPP V4YZX5 V4YZX5_TOXGO Acetyl-coA carboxylase ACC2 3400 473-478
159. VVAAPP V4Z4T1 V4Z4T1_TOXGO Uncharacterized protein 1799 216-221
160. VAAPPA B6KFV0 B6KFV0_TOXGO Uncharacterized protein 1266 824-829
161. VAAPPA B6KJS1 B6KJS1_TOXGO snRNA-activating of 50 kDa MW carboxy-terminal protein 1179 915-920
162. VAAPPA V4YJS1 V4YJS1_TOXGO Phosphatidylinositol-4-phosphate 5-Kinase 4165 1258-1263
163. AAPPAR B9QCV4 B9QCV4_TOXGO Uncharacterized protein 1702 1552-1557
164. AAPPAR V4Z4I8 V4Z4I8_TOXGO Uncharacterized protein 991 800-805
165. AAPPAR V4Z670 V4Z670_TOXGO Uncharacterized protein 166 137-142
166. AAPPAR V4Z6Z0 V4Z6Z0_TOXGO Uncharacterized protein 4064 761-766
167. AAPPAR V4Z8K1 V4Z8K1_TOXGO RNA polymerase Rpb1 C-terminal repeat-containing protein 2419 234-239
168. AAPPAR V4ZJ50 V4ZJ50_TOXGO Oligomeric complex protein COG6 1298 525-530
169. APPARD V4Z819 V4Z819_TOXGO DEAD/DEAH box helicase domain-containing protein 1850 772-777
170. PPARDP V4Z4I4 V4Z4I4_TOXGO Molybdopterin guanine dinucleotide synthesis protein B 2440 1321-1326
171. ARDPAS A7Y418 A7Y418_TOXGO Membrane protein FtsH1 1250 130-135
172. ARDPAS V4ZE58 V4ZE58_TOXGO Membrane protein FtsH1 1250 130-135
173. RDPASL B6KHN8 B6KHN8_TOXGO Uncharacterized protein 1264 377-382
174. RDPASL B9Q4U1 B9Q4U1_TOXGO Amine-terminal region of chorein, A TM vesicle-mediated sorter 10329 2426-2431
175. RDPASL V4ZCY8 V4ZCY8_TOXGO Pentatricopeptide repeat domain-containing protein 1904 1707-1712
176. RDPASL V4ZL39 V4ZL39_TOXGO Zn-finger in ubiquitin-hydrolases domain-containing protein 2582 1188-1193
177. ASLTRG V5BBK1 V5BBK1_TOXGO Aldo/keto reductase family oxidoreductase 525 53-58
178. SLTRGL K7WFS9 K7WFS9_TOXGO DHHC11 944 705-710
179. SLTRGL V4Z994 V4Z994_TOXGO DHHC zinc finger domain-containing protein 951 712-717
180. SLTRGL V5BBK1 V5BBK1_TOXGO Aldo/keto reductase family oxidoreductase 525 54-59
181. GLCQAL B6KBA0 B6KBA0_TOXGO Transporter, major facilitator family protein 775 401-406
182. GLCQAL B6KRH9 B6KRH9_TOXGO Uncharacterized protein 1898 1857-1862
183. LCQALV B6KRH9 B6KRH9_TOXGO Uncharacterized protein 1898 1858-1863
184. QALVPP B9QN82 B9QN82_TOXGO Uncharacterized protein 759 550-555
185. ALVPPG B9QN82 B9QN82_TOXGO Uncharacterized protein 759 551-556
186. ALVPPG V4Z5P5 V4Z5P5_TOXGO Uncharacterized protein 5771 546-551
187. ALVPPG V4Z9I2 V4Z9I2_TOXGO Metallo-beta-lactamase domain-containing protein 1196 832-837
188. ALVPPG V4ZPB5 V4ZPB5_TOXGO Putative tRNA (Cytosine(34)-C(5))-methyltransferase 1066 258-263
189. PPGVAA B9Q655 B9Q655_TOXGO RNA recognition motif-containing protein 1374 227-232
190. PPGVAA V4ZCD5 V4ZCD5_TOXGO FHA domain-containing protein 1042 598-603
191. PPGVAA V4ZDF1 V4ZDF1_TOXGO Uncharacterized protein 1485 957-962
192. PGVAAL B9QI15 B9QI15_TOXGO Phosphoglycerate kinase 418 410-415
193. PGVAAL Q1KSE7 Q1KSE7_TOXGO Phosphoglycerate kinase 551 539-544
194. PGVAAL Q1KSE8 Q1KSE8_TOXGO Phosphoglycerate kinase 416 409-414
195. PGVAAL V4ZCI3 V4ZCI3_TOXGO Phosphoglycerate kinase 593 581-586
196. PGVAAL V4ZEL7 V4ZEL7_TOXGO CPSF A subunit region protein 2847 1601-1606
197. PGVAAL V5B7V9 V5B7V9_TOXGO L1P family of ribosomal protein 928 473-478
198. GVAALL V4YQD6 V4YQD6_TOXGO Putative transmembrane protein 391 54-59
199. GVAALL V4Z6U0 V4Z6U0_TOXGO ATP-dependent Clp protease proteolytic subunit 486 358-363
200. GVAALL V4ZAY0 V4ZAY0_TOXGO Protein phosphatase 2C domain-containing protein 909 811-816
201. GVAALL V4ZBH2 V4ZBH2_TOXGO DnaJ domain-containing protein 645 192-197
202. GVAALL V4ZFR1 V4ZFR1_TOXGO HEAT repeat-containing protein 1697 647-652
203. GVAALL V4ZQ33 V4ZQ33_TOXGO Putative folate/biopterin transporter 469 296-301
204. GVAALL V5B1Q8 V5B1Q8_TOXGO Putative transmembrane protein 913 496-501
205. GVAALL V5B7E4 V5B7E4_TOXGO Putative transmembrane protein 2871 678-683
206. GVAALL V5BL61 V5BL61_TOXGO Putative transmembrane protein 564 388-393
207. VAALLA B6K9H9 B6K9H9_TOXGO Uncharacterized protein 417 12-17
208. VAALLA B6KUG9 B6KUG9_TOXGO Putative transmembrane protein 373 17-22
209. VAALLA B9QF97 B9QF97_TOXGO Uncharacterized protein 187 14-19
210. VAALLA B9QNV6 B9QNV6_TOXGO DIE2/alg10 family protein 927 91-96
211. VAALLA Q1JTK1 Q1JTK1_TOXGO Putative uncharacterized protein precursor 373 17-22
212. VAALLA V4Z0N9 V4Z0N9_TOXGO Putative phosphoenolpyruvate carboxykinase 614 596-601
213. VAALLA V4Z409 V4Z409_TOXGO HECT-domain (Ubiquitin-transferase) domain-containing protein 1709 93-98
214. VAALLA V4Z8P7 V4Z8P7_TOXGO Zinc finger protein ZFP1 1070 683-688
215. VAALLA V4ZCZ6 V4ZCZ6_TOXGO AP2 domain transcription factor AP2VIIa-4 3431 482-487
216. VAALLA V4ZDV1 V4ZDV1_TOXGO Uncharacterized protein 815 556-561
217. VAALLA V4ZGX1 V4ZGX1_TOXGO Uncharacterized protein 3917 2953-2958
218. VAALLA V4ZN78 V4ZN78_TOXGO Uncharacterized protein 1185 287-292
219. AALLAF B9QJF2 B9QJF2_TOXGO Toxoplasma gondii family A protein 416 8-13
220. AALLAF V4YTT3 V4YTT3_TOXGO Toxoplasma gondii family A protein 427 2-7
221. AALLAF V4Z0T5 V4Z0T5_TOXGO Uncharacterized protein 964 329-334
222. AALLAF V4Z8E0 V4Z8E0_TOXGO Toxoplasma gondii family A protein 151 8-13
223. AALLAF V4Z967 V4Z967_TOXGO Uncharacterized protein 2849 1583-1588
224. AALLAF V4ZDU0 V4ZDU0_TOXGO Putative transmembrane protein 1921 1190-1195
225. AALLAF V4ZV93 V4ZV93_TOXGO Uncharacterized protein 4618 4379-4384
226. LLAFPE B9Q792 B9Q792_TOXGO Uncharacterized protein 4118 1143-1148
227. LLAFPE B9QGB0 B9QGB0_TOXGO Uncharacterized protein 1165 1047-1052
228. LLAFPE V4ZU93 V4ZU93_TOXGO 3'5'-cyclic nucleotide phosphodiesterase domain-containing protein 1037 816-821
229. RPELLQ V4Z5V7 V4Z5V7_TOXGO Uncharacterized protein 1087 335-340
230. RPELLQ V4Z9E6 V4Z9E6_TOXGO Uncharacterized protein 2263 846-851
231. ELLQLH V4YQQ2 V4YQQ2_TOXGO Uncharacterized protein 1830 990-995
232. HFLAAA V4ZTS0 V4ZTS0_TOXGO Uncharacterized protein 2211 538-543
233. LAAATE B6KAG9 B6KAG9_TOXGO U3 small nucleolar RNA-associated protein 10 3738 391-396
234. LAAATE B6KL02 B6KL02_TOXGO Putative DNA double-strand break repair rad50 ATPase 600 526-531
235. LAAATE B9QI90 B9QI90_TOXGO Bestrophin 878 773-778
236. LAAATE B9QJ37 B9QJ37_TOXGO Uncharacterized protein 719 386-391
237. LAAATE V4Z3V3 V4Z3V3_TOXGO Uncharacterized protein 3415 2955-2960
238. LAAATE V4Z6S2 V4Z6S2_TOXGO AP2 domain transcription factor AP2X-5 1972 1118-1123
239. LAAATE V4Z7M0 V4Z7M0_TOXGO T-complex protein 1 eta subunit 546 508-513
240. AAATET V4Z3V3 V4Z3V3_TOXGO Uncharacterized protein 3415 2956-2961
241. AATETP B9Q8F9 B9Q8F9_TOXGO Putative elongation factor Tu GTP-binding domain protein 1766 1673-1678
242. AATETP Q1JT85 Q1JT85_TOXGO Putative uncharacterized protein 663 533-538
243. AATETP V4YLR5 V4YLR5_TOXGO YrdC domain-containing protein 828 672-677
244. AATETP V4Z3V3 V4Z3V3_TOXGO Uncharacterized protein 3415 2957-2962
245. AATETP V4Z5T8 V4Z5T8_TOXGO Uncharacterized protein 3255 1509-1514
246. AATETP V4ZA66 V4ZA66_TOXGO Uncharacterized protein 1241 873-878
247. ATETPV Q1JT85 Q1JT85_TOXGO Putative uncharacterized protein 663 534-539
248. ATETPV V4YLR5 V4YLR5_TOXGO YrdC domain-containing protein 828 673-678
249. TETPVL V4ZBT0 V4ZBT0_TOXGO Uncharacterized protein 100 82-87
250. ETPVLS V4ZUZ7 V4ZUZ7_TOXGO Uncharacterized protein 1822 1465-1470
251. TPVLSL Q1JT90 Q1JT90_TOXGO tRNA (guanine-N(7)-)-methyltransferase 309 222-227
252. TPVLSL V4Z5N5 V4Z5N5_TOXGO tRNA (guanine-N(7)-)-methyltransferase 325 238-243
253. TPVLSL V4ZEL7 V4ZEL7_TOXGO CPSF A subunit region protein 2847 98-103
254. TPVLSL V4ZUZ7 V4ZUZ7_TOXGO Uncharacterized protein 1822 1466-1471
255. PVLSLL B9QDX7 B9QDX7_TOXGO Uncharacterized protein 530 35-40
256. PVLSLL V4Z500 V4Z500_TOXGO Uncharacterized protein 604 13-18
257. PVLSLL V4ZAZ4 V4ZAZ4_TOXGO AP2 domain transcription factor AP2X-7 1869 1142-1147
258. VLSLLR B6KTX8 B6KTX8_TOXGO Putative transmembrane protein 785 242-247
259. VLSLLR B9QH60 B9QH60_TOXGO Acetyl-CoA carboxylase ACC1 2612 1191-1196
260. VLSLLR Q9U755 Q9U755_TOXGO Acetyl-CoA carboxylase 1 2564 1191-1196
261. VLSLLR V4YZN6 V4YZN6_TOXGO Putative nucleolar protein 9 960 589-594
262. VLSLLR V4Z9U0 V4Z9U0_TOXGO Uncharacterized protein 5083 4821-4826
263. VLSLLR V4ZDY2 V4ZDY2_TOXGO Ribonuclease type III Dicer 4343 422-427
264. VLSLLR V4ZMI2 V4ZMI2_TOXGO Uncharacterized protein 186 107-112
265. LSLLRR B6KEU8 GRA3_TOXGO Dense granule protein 3 222 119-124
266. LSLLRR B6KRH9 B6KRH9_TOXGO Uncharacterized protein 1898 587-592
267. LSLLRR B9Q792 B9Q792_TOXGO Uncharacterized protein 4118 227-232
268. LSLLRR B9QED8 B9QED8_TOXGO Uncharacterized protein 1404 379-384
269. LSLLRR I7BEL3 I7BEL3_TOXGO Dense granule protein 3 222 119-124
270. LSLLRR I7BEL9 I7BEL9_TOXGO Dense granule protein 3 222 119-124
271. LSLLRR I7BEN0 I7BEN0_TOXGO Dense granule protein 3 222 119-124
272. LSLLRR I7BPD1 I7BPD1_TOXGO Dense granule protein 3 222 119-124
273. LSLLRR I7CB41 I7CB41_TOXGO Dense granule protein 3 222 119-124
274. LSLLRR I7CKX6 I7CKX6_TOXGO Dense granule protein 3 222 119-124
275. LSLLRR I7CKY8 I7CKY8_TOXGO Dense granule protein 3 222 119-124
276. LSLLRR I7CKZ3 I7CKZ3_TOXGO Dense granule protein 3 220 119-124
277. LSLLRR I7CPR3 I7CPR3_TOXGO Dense granule protein 3 222 119-124
278. LSLLRR I7CPS1 I7CPS1_TOXGO Dense granule protein 3 220 119-124
279. LSLLRR V4ZF62 V4ZF62_TOXGO Uncharacterized protein 1578 321-326
280. LSLLRR V4ZJ61 V4ZJ61_TOXGO Uncharacterized protein 627 327-332
281. LSLLRR V4ZMI2 V4ZMI2_TOXGO Uncharacterized protein 186 108-113
282. LSLLRR V4ZRU8 V4ZRU8_TOXGO BTB/POZ domain-containing protein 1289 595-600
283. LSLLRR V4ZVU8 V4ZVU8_TOXGO Uncharacterized protein 4014 1118-1123
284. SLLRRE B6KEU8 GRA3_TOXGO Dense granule protein 3 222 120-125
285. SLLRRE B6KJS8 B6KJS8_TOXGO Endonuclease/exonuclease/phosphatase family protein 978 228-233
286. SLLRRE I7BEL3 I7BEL3_TOXGO Dense granule protein 3 222 120-125
287. SLLRRE I7BEL9 I7BEL9_TOXGO Dense granule protein 3 222 120-125
288. SLLRRE I7BEN0 I7BEN0_TOXGO Dense granule protein 3 222 120-125
289. SLLRRE I7BPD1 I7BPD1_TOXGO Dense granule protein 3 222 120-125
290. SLLRRE I7CB41 I7CB41_TOXGO Dense granule protein 3 222 120-125
291. SLLRRE I7CKX6 I7CKX6_TOXGO Dense granule protein 3 222 120-125
292. SLLRRE I7CKY8 I7CKY8_TOXGO Dense granule protein 3 222 120-125
293. SLLRRE I7CKZ3 I7CKZ3_TOXGO Dense granule protein 3 220 120-125
294. SLLRRE I7CPR3 I7CPR3_TOXGO Dense granule protein 3 222 120-125
295. SLLRRE I7CPS1 I7CPS1_TOXGO Dense granule protein 3 220 120-125
296. SLLRRE Q1JT23 Q1JT23_TOXGO Putative uncharacterized protein 1439 707-712
297. SLLRRE V4Z0B8 V4Z0B8_TOXGO Uncharacterized protein 144 48-53
298. SLLRRE V4Z164 V4Z164_TOXGO Uncharacterized protein 1439 707-712
299. SLLRRE V4ZMI2 V4ZMI2_TOXGO Uncharacterized protein 186 109-114
300. SLLRRE V5B823 V5B823_TOXGO Spc97 / Spc98 family protein 1877 493-498
301. LLRREA B6KP07 B6KP07_TOXGO DnaJ C terminal region domain-containing protein 1519 936-941
302. LLRREA Q1JSW6 Q1JSW6_TOXGO Uncharacterized protein 1064 936-941
303. LLRREA V4YT41 V4YT41_TOXGO Protein kinase domain protein 6052 4793-4798
304. LLRREA V4Z2F7 V4Z2F7_TOXGO Putative transmembrane protein 2862 1688-1693
305. LLRREA V4ZIE0 V4ZIE0_TOXGO DNA polymerase 4247 2293-2298
306. LRREAR B6KHB5 B6KHB5_TOXGO Zinc knuckle protein 502 406-411
307. LRREAR V4YU44 V4YU44_TOXGO Uncharacterized protein 2391 694-699
308. LRREAR V4YY09 V4YY09_TOXGO KRUF family protein 652 514-519
309. LRREAR V4Z6B8 V4Z6B8_TOXGO Uncharacterized protein 351 152-157
310. LRREAR V4ZIE0 V4ZIE0_TOXGO DNA polymerase 4247 2294-2299
311. RREARA V5BE00 V5BE00_TOXGO Uncharacterized protein 144 43-48
312. ARAPLG V4YSP2 V4YSP2_TOXGO Ribosomal protein RPL29 877 227-232
313. RAPLGA Q1JSL3 Q1JSL3_TOXGO mRNA decapping enzyme, putative precursor 512 163-168
314. RAPLGA V4YMB2 V4YMB2_TOXGO Putative mRNA decapping enzyme 400 51-56
315. RAPLGA V4YU76 V4YU76_TOXGO Tubulin-tyrosine ligase family protein 2376 1278-1283
316. APLGAP Q1JSL3 Q1JSL3_TOXGO mRNA decapping enzyme, putative precursor 512 164-169
317. APLGAP V4YMB2 V4YMB2_TOXGO Putative mRNA decapping enzyme 400 52-57
318. APLGAP V4ZBH1 V4ZBH1_TOXGO Uncharacterized protein 3736 3574-3579
319. PLGAPN B6KA08 B6KA08_TOXGO TAF7-like RNA polymerase II TAF7L 728 20-25
320. LHWASP V4Z4U3 V4Z4U3_TOXGO ABC transporter, ATP-binding domain-containing protein 1885 948-953
321. ASPLET V4ZAC1 V4ZAC1_TOXGO Rhoptry kinase family protein ROP31 531 87-92
322. ASPLET V4ZKG4 V4ZKG4_TOXGO Zinc finger (CCCH type) motif-containing protein 816 698-703
323. ASPLET V5B7E4 V5B7E4_TOXGO Putative transmembrane protein 2871 2179-2184
324. SPLETL B6KHJ3 B6KHJ3_TOXGO Putative dynein heavy chain 2 4140 813-818
325. SPLETL V4ZC76 V4ZC76_TOXGO Phosphofructokinase domain-containing protein 3001 1506-1511
326. SPLETL V4ZKG4 V4ZKG4_TOXGO Zinc finger (CCCH type) motif-containing protein 816 699-704
327. PLETLL V4ZC76 V4ZC76_TOXGO Phosphofructokinase domain-containing protein 3001 1507-1512
328. LETLLD B9Q0Q3 B9Q0Q3_TOXGO Pb-fam-5 protein 160 56-61
329. LETLLD B9QNV2 B9QNV2_TOXGO ABC transporter transmembrane region domain-containing protein 1005 866-871
330. LETLLD Q45W13 Q45W13_TOXGO Putative ATP-binding cassette protein 342 195-200
331. ETLLDV B6KTZ2 B6KTZ2_TOXGO Uncharacterized protein 1589 172-177
[truncated: 54,106 more chars]
