# Supplementary material for: From Toxoplasmosis to Schizophrenia via NMDA Dysfunction: Peptide Overlap between Toxoplasma gondii and N-Methyl-d-Aspartate Receptors As a Potential Mechanistic Link
Source: Front Psychiatry. 2017 Mar 15;8:37. doi: 10.3389/fpsyt.2017.00037 (PMC5350139; doi:10.3389/fpsyt.2017.00037)
Supplement: Supplementary file 2 [file Table_2.DOC]

**Supplementary Table S2. Distribution of NMDAR hexapeptides (1394 including multiple occurrences) throughout the *E. histolytica* proteome**

N° NMDAR *E. histolytica* *E. histolytica* *E. histolytica* Protein Name Protein Matched Range(s)

Peptide Protein AC Protein ID length (aa)

**NMDA 1**

1. STMRLL C4LZ30 C4LZ30_ENTHI [tr] Uncharacterized protein 730 156-161
2. STMRLL C4LZD1 C4LZD1_ENTHI [tr] Protein with RhoGEF and ArfGAP domains 1098 423-428
3. TLALLF C4LSF8 C4LSF8_ENTHI [tr] Uncharacterized protein 4492 2303-2308
4. TLALLF C4M5C8 C4M5C8_ENTHI [tr] Uncharacterized protein 2378 2334-2339
5. LALLFS C4M5C8 C4M5C8_ENTHI [tr] Uncharacterized protein 2378 2335-2340
6. KIVNIG A0A060N6K9 A0A060N6K9_ENTHI [tr] LIM zinc finger domain containing protein 301 15-20
7. IVNIGA C4M369 C4M369_ENTHI [tr] Ras guanine nucleotide exchange factor putative 1246 167-172
8. AIQMAL C4M3B4 C4M3B4_ENTHI [tr] Uncharacterized protein 830 557-562
9. EDLISS B1N5I5 B1N5I5_ENTHI [tr] Uncharacterized protein 152 67-72
10. EDLISS C4LW50 C4LW50_ENTHI [tr] Uncharacterized protein 396 311-316
11. TPTPVS A0A175JWR2 A0A175JWR2_ENTHI [tr] Protein phosphatase domain-containing protein 769 451-456
12. TPTPVS B1N4E9 B1N4E9_ENTHI [tr] Protein phosphatase domain-containing protein 709 391-396
13. RMSIYS C4LX13 C4LX13_ENTHI [tr] Cell division control protein CDC24, putative 749 557-562
14. IYSDKS A0A175JIP0 A0A175JIP0_ENTHI [tr] Uncharacterized protein 3302 313-318
15. IYSDKS C4LX72 C4LX72_ENTHI [tr] Uncharacterized protein 3289 300-305
16. KSIHLS A0A175JUE0 A0A175JUE0_ENTHI [tr] Spindle pole body component 571 371-376
17. KSIHLS C4LUJ9 C4LUJ9_ENTHI [tr] RhoGAP domain containing protein 593 64-69
18. LSFLRT B1N521 B1N521_ENTHI [tr] P-glycoprotein 5, putative 290 104-109
19. LSFLRT C4M3B2 C4M3B2_ENTHI [tr] P-glycoprotein-5 1296 122-127
20. LSFLRT C4M9Z7 C4M9Z7_ENTHI [tr] P-glycoprotein 5, putative 609 96-101
21. LSFLRT Q24850 Q24850_ENTHI [tr] P-glycoprotein 5 1301 123-128
22. IILLVS A0A175JCP4 A0A175JCP4_ENTHI [tr] Uncharacterized protein 556 201-206
23. IILLVS C4LSN0 C4LSN0_ENTHI [tr] Uncharacterized protein 530 175-180
24. IILLVS C4LTT9 C4LTT9_ENTHI [tr] Uncharacterized protein 321 278-283
25. IILLVS C4M8P6 C4M8P6_ENTHI [tr] Helicase putative 1192 377-382
26. IILLVS Q1EQ04 Q1EQ04_ENTHI [tr] EhSyntaxin A 214 189-194
27. ILLVSD C4LSX1 C4LSX1_ENTHI [tr] Uncharacterized protein 296 83-88
28. ILLVSD C4LTT9 C4LTT9_ENTHI [tr] Uncharacterized protein 321 279-284
29. AQKRLE C4M013 C4M013_ENTHI [tr] Acetyltransferase putative 202 45-50
30. AQKRLE C4MAJ4 C4MAJ4_ENTHI [tr] Acetyltransferase putative 202 45-50
31. RLETLL C4M9I9 C4M9I9_ENTHI [tr] Ubiquitin-protein ligase putative 661 77-82
32. LLEERE A0A175JY54 A0A175JY54_ENTHI [tr] Repeat organellar protein putative 824 410-415
33. LLEERE A0A175JYA0 A0A175JYA0_ENTHI [tr] Uncharacterized protein 683 269-274
34. LLEERE A0A175JYE8 A0A175JYE8_ENTHI [tr] Uncharacterized protein 598 184-189
35. LLEERE A0A175JYZ7 A0A175JYZ7_ENTHI [tr] Uncharacterized protein 824 410-415
36. LLEERE A0A175JZ11 A0A175JZ11_ENTHI [tr] Interaptin putative 683 269-274
37. LLEERE C4LWD6 C4LWD6_ENTHI [tr] Helicase putative 675 179-184
38. LLEERE C4MA92 C4MA92_ENTHI [tr] Interaptin, putative 1073 659-664
39. LLEERE C4MA98 C4MA98_ENTHI [tr] Uncharacterized protein 1105 691-696
40. LLEERE C4MAB8 C4MAB8_ENTHI [tr] Repeat organellar protein, putative 1105 691-696
41. LLEERE C4MAE6 C4MAE6_ENTHI [tr] Uncharacterized protein 1105 691-696
42. LLEERE C4MAQ0 C4MAQ0_ENTHI [tr] Uncharacterized protein 1073 659-664
43. KNVTAL C4LU61 C4LU61_ENTHI [tr] Uncharacterized protein 260 40-45
44. NVTALL B1N5W6 B1N5W6_ENTHI [tr] Uncharacterized protein 294 240-245
45. NVTALL C4LY68 C4LY68_ENTHI [tr] Transporter major facilitator family 524 320-325
46. NVTALL C4M4T5 C4M4T5_ENTHI [tr] Transporter major facilitator family 519 301-306
47. EAKELE C4M052 C4M052_ENTHI [tr] Uncharacterized protein 307 188-193
48. AKELEA A0A175JZ71 A0A175JZ71_ENTHI [tr] Uncharacterized protein 190 88-93
49. AKELEA C4M2E5 C4M2E5_ENTHI [tr] Uncharacterized protein 243 82-87
50. AKELEA C4MB17 C4MB17_ENTHI [tr] Uncharacterized protein 249 88-93
51. LSASED C4M762 C4M762_ENTHI [tr] Tyrosine kinase putative 1228 110-115
52. LGLQLI C4LZQ7 C4LZQ7_ENTHI [tr] Rab GTPase activating protein putative 874 22-27
53. QLINGK A0A175JUS6 A0A175JUS6_ENTHI [tr] Uncharacterized protein 2426 453-458
54. QLINGK B1N4E0 B1N4E0_ENTHI [tr] Uncharacterized protein 123 94-99
55. QLINGK C4M6M0 C4M6M0_ENTHI [tr] Uncharacterized protein 2415 442-447
56. INGKNE C4M7Q5 C4M7Q5_ENTHI [tr] AMP deaminase, putative 1327 364-369
57. HELLEK C4LXM2 C4LXM2_ENTHI [tr] Uncharacterized protein 713 298-303
58. ELLEKE A0A175JRE8 A0A175JRE8_ENTHI [tr] Protein mnn4 putative 340 188-193
59. ELLEKE B1N606 B1N606_ENTHI [tr] Protein MNN4, putative 214 62-67
60. ELLEKE C4M4I2 C4M4I2_ENTHI [tr] Uncharacterized protein 647 365-370
61. ELLEKE C4M654 C4M654_ENTHI [tr] Uncharacterized protein 540 163-168
62. ELLEKE C4M879 C4M879_ENTHI [tr] Uncharacterized protein 105 40-45
63. LEKENI C4M741 C4M741_ENTHI [tr] Cortexillin putative 753 569-574
64. EKENIT A0A175JGK9 A0A175JGK9_ENTHI [tr] Uncharacterized protein 538 298-303
65. EKENIT C4LVW3 C4LVW3_ENTHI [tr] Uncharacterized protein 534 294-299
66. ENITDP C4LXX0 C4LXX0_ENTHI [tr] Phosphatidylinositol-glycan biosynthesis class O protein, putative 870 94-99
67. ENITDP C4M173 C4M173_ENTHI [tr] Phosphatidylinositol-glycan biosynthesis class O protein, putative 866 94-99
68. DPPRGC C4LY36 C4LY36_ENTHI [tr] 26S protease regulatory subunit putative 389 168-173
69. SKYADG C4LXG6 C4LXG6_ENTHI [tr] AAA family ATPase putative 482 214-219
70. FNEDGD A0A175JFU2 A0A175JFU2_ENTHI [tr] Uncharacterized protein 1081 247-252
71. FNEDGD C4LUP4 C4LUP4_ENTHI [tr] Uncharacterized protein 1060 226-231
72. MNLQNR C4LVS1 C4LVS1_ENTHI [tr] Phosphofructokinase putative 439 108-113
73. MNLQNR C4M3U3 C4M3U3_ENTHI [tr] Phosphofructokinase putative 439 108-113
74. VGIYNG C4M770 C4M770_ENTHI [tr] 70 kDa heat shock protein putative 661 41-46
75. VGIYNG O77164 O77164_ENTHI [tr] 70 kDa heat shock protein Hsp70-Bip 661 41-46
76. GTHVIP C4LTU9 C4LTU9_ENTHI [tr] Structural maintenance of chromosomes protein 1197 17-22
77. GGETEK C4LTM7 C4LTM7_ENTHI [tr] Uncharacterized protein 883 803-808
78. GGETEK E5RSJ5 E5RSJ5_ENTHI [tr] Cysteine protease binding protein family 4 893 813-818
79. YQMSTR C4M2X9 C4M2X9_ENTHI [tr] Leucine rich repeat protein bspa family 722 41-46
80. QMSTRL A0A175JWZ4 A0A175JWZ4_ENTHI [tr] Protein kinase putative 1933 1657-1662
81. QMSTRL C4M8M5 C4M8M5_ENTHI [tr] Tyrosine kinase, putative 1056 780-785
82. QEPFVY A0A175JWU4 A0A175JWU4_ENTHI [tr] Uncharacterized protein 710 691-696
83. QEPFVY C4M9B5 C4M9B5_ENTHI [tr] Uncharacterized protein 701 682-687
84. KPTLSD C4M6U2 C4M6U2_ENTHI [tr] Uncharacterized protein 555 270-275
85. TLSDGT C4LVF1 C4LVF1_ENTHI [tr] Protein tyrosine kinase domain-containing protein 1055 488-493
86. LSDGTC A0A175JG05 A0A175JG05_ENTHI [tr] Uncharacterized protein 349 305-310
87. LSDGTC C4LUU4 C4LUU4_ENTHI [tr] Uncharacterized protein 334 290-295
88. DGTCKE A0A175JWZ4 A0A175JWZ4_ENTHI [tr] Protein kinase putative 1933 1156-1161
89. DGTCKE C4M7I1 C4M7I1_ENTHI [tr] CXXC-rich protein 1179 1019-1024
90. DGTCKE C4M8M5 C4M8M5_ENTHI [tr] Tyrosine kinase, putative 1056 279-284
91. GTCKEE C4M481 C4M481_ENTHI [tr] Protein kinase putative 756 668-673
92. KKVICT C4LX44 C4LX44_ENTHI [tr] Iron hydrogenase putative 398 98-103
93. IDLLIK C4M0K7 C4M0K7_ENTHI [tr] Diaphanous protein, homolog 3, putative 843 641-646
94. DLLIKL C4M0K7 C4M0K7_ENTHI [tr] Diaphanous protein, homolog 3, putative 843 642-647
95. TYEVHL C4M446 C4M446_ENTHI [tr] Tyrosine kinase putative 1348 1003-1008
96. VNNSNK C4M643 C4M643_ENTHI [tr] Uncharacterized protein 1656 830-835
97. SNKKEW A0A175JQ50 A0A175JQ50_ENTHI [tr] tRNA nucleotidyltransferase putative 468 342-347
98. SNKKEW C4M2J8 C4M2J8_ENTHI [tr] tRNA nucleotidyltransferase, putative 461 335-340
99. MGELLS C4M142 C4M142_ENTHI [tr] N-system amino acid transporter 1 putative 699 221-226
100. MGELLS C4M9T9 C4M9T9_ENTHI [tr] Amino acid transporter putative 700 215-220
101. GELLSG C4LUB5 C4LUB5_ENTHI [tr] Tryptophanyl-tRNA synthetase putative 381 338-343
102. LTINNE C4M7L7 C4M7L7_ENTHI [tr] Uncharacterized protein 267 126-131
103. TINNER C4M7L7 C4M7L7_ENTHI [tr] Uncharacterized protein 267 127-132
104. ILVKKE C4LYI1 C4LYI1_ENTHI [tr] DEAD/DEAH box helicase, putative 391 219-224
105. KKEIPR C4LUF9 C4LUF9_ENTHI [tr] Uncharacterized protein 512 97-102
106. LVGLSV A0A175JM31 A0A175JM31_ENTHI [tr] Uncharacterized protein 135 115-120
107. LVGLSV C4M089 C4M089_ENTHI [tr] Uncharacterized protein 126 106-111
108. HVVAVM A0A175JDF8 A0A175JDF8_ENTHI [tr] Uncharacterized protein 180 145-150
109. HVVAVM C4LSF4 C4LSF4_ENTHI [tr] Uncharacterized protein 137 102-107
110. VMLYLL C4M462 C4M462_ENTHI [tr] Phospholipid-transporting ATPase 1335 1137-1142
111. VNSEEE C4M2M6 C4M2M6_ENTHI [tr] Rho guanine nucleotide exchange factor putative 921 441-446
112. SEEEEE A0A175JJL6 A0A175JJL6_ENTHI [tr] Uncharacterized protein 289 193-198
113. SEEEEE B1N300 B1N300_ENTHI [tr] Uncharacterized protein 242 146-151
114. SEEEEE C4LTF9 C4LTF9_ENTHI [tr] High mobility group (HMG) box domain containing protein 111 100-105
115. SEEEEE C4LTR5 C4LTR5_ENTHI [tr] Uncharacterized protein 385 190-195
116. SEEEEE C4LUU3 C4LUU3_ENTHI [tr] Uncharacterized protein 256 27-32
117. SEEEEE C4LYH1 C4LYH1_ENTHI [tr] High mobility group (HMG) box domain containing protein 114 103-108
118. SEEEEE C4LZ23 C4LZ23_ENTHI [tr] Phospholipid-transporting ATPase 1099 410-415
119. SEEEEE C4M012 C4M012_ENTHI [tr] DNAj domain containing protein 719 709-714
120. SEEEEE C4M0T7 C4M0T7_ENTHI [tr] Probable ribosome biogenesis protein CL6EHI_100490 234 221-226, 228-233
121. SEEEEE C4M0X9 C4M0X9_ENTHI [tr] Uncharacterized protein 257 176-181
122. SEEEEE C4M3V7 C4M3V7_ENTHI [tr] Uncharacterized protein 329 174-179
123. SEEEEE C4M442 C4M442_ENTHI [tr] Uncharacterized protein 391 75-80
124. SEEEEE C4M496 C4M496_ENTHI [tr] 158792 GRFA protein putative 858 36-41
125. SEEEEE C4M4W6 C4M4W6_ENTHI [tr] Uncharacterized protein 245 198-203
126. SEEEEE C4M5V6 C4M5V6_ENTHI [tr] Uncharacterized protein 496 164-169
127. SEEEEE C4M6A1 C4M6A1_ENTHI [tr] Structure specific recognition protein putative 376 368-373
128. SEEEEE C4M761 C4M761_ENTHI [tr] Ras-likeGTP-binding protein YPT1, putative 239 212-217
129. SEEEEE C4M7E0 C4M7E0_ENTHI [tr] Uncharacterized protein 565 46-51
130. EEEEED A0A060N0B5 A0A060N0B5_ENTHI [tr] 60S acidic ribosomal protein P0 316 303-308
131. EEEEED A0A060N0H1 A0A060N0H1_ENTHI [tr] 60S acidic ribosomal protein P0 316 303-308
132. EEEEED A0A060N1F4 A0A060N1F4_ENTHI [tr] 60S acidic ribosomal protein P0 316 303-308
133. EEEEED A0A060N4V3 A0A060N4V3_ENTHI [tr] 60S acidic ribosomal protein P0 316 303-308
134. EEEEED A0A060N6L5 A0A060N6L5_ENTHI [tr] 60S acidic ribosomal protein P0 316 303-308
135. EEEEED A0A175JIV9 A0A175JIV9_ENTHI [tr] Uncharacterized protein 284 74-79, 217-222
136. EEEEED A0A175JJL6 A0A175JJL6_ENTHI [tr] Uncharacterized protein 289 194-199
137. EEEEED A0A175K1V8 A0A175K1V8_ENTHI [tr] Uncharacterized protein 287 74-79, 220-225
138. EEEEED A0A175K2E1 A0A175K2E1_ENTHI [tr] Uncharacterized protein 259 228-233
139. EEEEED B1N2Y9 B1N2Y9_ENTHI [tr] Uncharacterized protein 284 74-79, 217-222
140. EEEEED B1N2Z3 B1N2Z3_ENTHI [tr] 60S acidic ribosomal protein P0 316 303-308
141. EEEEED B1N300 B1N300_ENTHI [tr] Uncharacterized protein 242 147-152
142. EEEEED B1N3L3 B1N3L3_ENTHI [tr] 60S acidic ribosomal protein p2 putative 61 48-53
143. EEEEED C4LTR5 C4LTR5_ENTHI [tr] Uncharacterized protein 385 191-196
144. EEEEED C4LUQ8 C4LUQ8_ENTHI [tr] Uncharacterized protein 581 422-427
145. EEEEED C4LVH5 C4LVH5_ENTHI [tr] Translation initiation factor eIF-1A, putative 171 150-155
146. EEEEED C4LVQ0 C4LVQ0_ENTHI [tr] 60S acidic ribosomal protein P2, putative 106 93-98
147. EEEEED C4LXI2 C4LXI2_ENTHI [tr] Uncharacterized protein 850 773-778
148. EEEEED C4LYG8 C4LYG8_ENTHI [tr] Bromodomain protein, putative 1163 846-851
149. EEEEED C4LZN1 C4LZN1_ENTHI [tr] Uncharacterized protein 256 186-191
150. EEEEED C4M0T7 C4M0T7_ENTHI [tr] Probable ribosome biogenesis protein CL6EHI_100490 234 229-234
151. EEEEED C4M2L6 C4M2L6_ENTHI [tr] Uncharacterized protein 271 260-265
152. EEEEED C4M3N3 C4M3N3_ENTHI [tr] Uncharacterized protein 614 32-37, 57-62
153. EEEEED C4M3N7 C4M3N7_ENTHI [tr] Putative transcription enhancer protein 1175 333-338
154. EEEEED C4M4I3 C4M4I3_ENTHI [tr] 60S acidic ribosomal protein P2, putative 105 92-97
155. EEEEED C4M4W6 C4M4W6_ENTHI [tr] Uncharacterized protein 245 204-209
156. EEEEED C4M5Q8 C4M5Q8_ENTHI [tr] Eukaryotic translation initiation factor eIF-5 349 342-347
157. EEEEED C4M649 C4M649_ENTHI [tr] 60S acidic ribosomal protein P2, putative 105 92-97
158. EEEEED C4M660 C4M660_ENTHI [tr] 60S acidic riboomal protein P1, putative 106 93-98
159. EEEEED C4M661 C4M661_ENTHI [tr] 60S acidic ribosomal protein P1, putative 111 98-103
160. EEEEED C4M9R6 C4M9R6_ENTHI [tr] Uncharacterized protein 246 16-21
161. EEEEED C4MAH9 C4MAH9_ENTHI [tr] Uncharacterized protein 283 175-180
162. EEEEED C4MB14 C4MB14_ENTHI [tr] Serine threonine-protein kinase 3 putative 354 331-336
163. EEEEED C4MB35 C4MB35_ENTHI [tr] Ubiquitin carboxyl-terminal hydrolase domain containing protein 1825 1086-1091
164. EEEEED C4MBT9 C4MBT9_ENTHI [tr] Uncharacterized protein 287 74-79, 220-225
165. EEEEED Q24850 Q24850_ENTHI [tr] P-glycoprotein 5 1301 670-675
166. EEEEED S0AUW3 S0AUW3_ENTHI [tr] 60S acidic ribosomal protein P0 316 303-308
167. EEEEED S0AVG0 S0AVG0_ENTHI [tr] 60S acidic ribosomal protein P0 316 303-308
168. EEEEED S0AVH2 S0AVH2_ENTHI [tr] 60S acidic ribosomal protein P0 316 303-308
169. EEEEED S0AW70 S0AW70_ENTHI [tr] 60S acidic ribosomal protein P0 316 303-308
170. EEEEED S0AWH1 S0AWH1_ENTHI [tr] 60S acidic ribosomal protein P0 319 306-311
171. EEEEED S0AWP8 S0AWP8_ENTHI [tr] 60S acidic ribosomal protein P0 316 303-308
172. EEEEED S0AWV6 S0AWV6_ENTHI [tr] 60S acidic ribosomal protein P0 316 303-308
173. EEEEED S0AX84 S0AX84_ENTHI [tr] 60S acidic ribosomal protein P0 316 303-308
174. EEEEED S0AXG4 S0AXG4_ENTHI [tr] 60S acidic ribosomal protein P0 316 303-308
175. EEEEED S0AXR9 S0AXR9_ENTHI [tr] Serine/threonine-protein kinase 3, putative 353 330-335
176. EEEEED S0AYC4 S0AYC4_ENTHI [tr] 60S acidic ribosomal protein P0 316 303-308
177. EEEEED S0AZ89 S0AZ89_ENTHI [tr] Uncharacterized protein 236 128-133
178. EEEEED S0AZB8 S0AZB8_ENTHI [tr] Serine/threonine-protein kinase 3, putative 353 330-335
179. EEEEED S0AZC9 S0AZC9_ENTHI [tr] 60S acidic ribosomal protein P0 316 303-308
180. EEEEED S0B010 S0B010_ENTHI [tr] 60S acidic ribosomal protein P0 316 303-308
181. EEEEED S0B0F9 S0B0F9_ENTHI [tr] 60S acidic ribosomal protein P0 316 303-308
182. EEEEED S0B1C1 S0B1C1_ENTHI [tr] Uncharacterized protein 250 150-155
183. EEEEED S0B2A3 S0B2A3_ENTHI [tr] 60S acidic ribosomal protein P0 316 303-308
184. EEEEED S0B2T7 S0B2T7_ENTHI [tr] 60S acidic ribosomal protein P0 316 303-308
185. EEEEDA A0A175JJL6 A0A175JJL6_ENTHI [tr] Uncharacterized protein 289 195-200
186. EEEEDA B1N300 B1N300_ENTHI [tr] Uncharacterized protein 242 148-153
187. EEEEDA C4M4W6 C4M4W6_ENTHI [tr] Uncharacterized protein 245 205-210
188. EEEEDA C4M816 C4M816_ENTHI [tr] Uncharacterized protein 607 273-278
189. EEEDAL A0A175JI58 A0A175JI58_ENTHI [tr] Rhogap domain containing protein 247 76-81
190. EEEDAL B1N2U9 B1N2U9_ENTHI [tr] RhoGAP domain containing protein 282 76-81
191. EEEDAL C4M4N5 C4M4N5_ENTHI [tr] RhoGAP domain containing protein 529 76-81
192. SWGVLL C4LVL0 C4LVL0_ENTHI [tr] Helicase putative 837 520-525
193. VLLNSG C4M1N4 C4M1N4_ENTHI [tr] Uncharacterized protein 345 324-329
194. LLNSGI B1N3S9 B1N3S9_ENTHI [tr] Uncharacterized protein 197 118-123
195. LNSGIG C4LYT4 C4LYT4_ENTHI [tr] Ran binding protein putative 1044 208-213
196. GIGEGA C4M6K0 C4M6K0_ENTHI [tr] Splicing factor Prp8, putative 2270 2151-2156
197. FSARIL A0A175JK81 A0A175JK81_ENTHI [tr] Uncharacterized protein 2654 1783-1788
198. FSARIL C4LYX0 C4LYX0_ENTHI [tr] Uncharacterized protein 2640 1783-1788
199. ILGMVW A0A175K209 A0A175K209_ENTHI [tr] Calponin-homology domain containing protein 353 101-106
200. ILGMVW C4M1S5 C4M1S5_ENTHI [tr] Actinin-like protein putative 458 23-28
201. ILGMVW C4M1S6 C4M1S6_ENTHI [tr] Actinin-like protein putative 522 23-28
202. ILGMVW Q9U3Z8 Q9U3Z8_ENTHI [tr] Actinin-like protein (Fragment) 537 102-107
203. NLAAFL C4M1J7 C4M1J7_ENTHI [tr] Uncharacterized protein 1034 963-968
204. NPSDKF C4M241 C4M241_ENTHI [tr] Uncharacterized protein 252 160-165
205. NPSDKF C4M6F8 C4M6F8_ENTHI [tr] Uncharacterized protein 886 799-804
206. QSSVDI C4M6L0 C4M6L0_ENTHI [tr] Gal galnac lectin subunit igl2 1105 755-760
207. QSSVDI Q964D1 Q964D1_ENTHI [tr] Gal/GalNAc lectin Igl2 (Fragment) 1074 735-740
208. QVELST C4M2Z3 C4M2Z3_ENTHI [tr] Uncharacterized protein 342 208-213
209. QVELST S0B0X0 S0B0X0_ENTHI [tr] Uncharacterized protein 343 209-214
210. YESAAE C4M2Y3 C4M2Y3_ENTHI [tr] Ubiquitin-conjugating enzyme family protein 165 103-108
211. EAIQAV C4LYB5 C4LYB5_ENTHI [tr] Replication factor C subunit 4, putative 329 186-191
212. FEASQK C4M1T1 C4M1T1_ENTHI [tr] Sec61 alpha subunit putative 344 111-116
213. FEASQK Q5XWC1 Q5XWC1_ENTHI [tr] Sec61 alpha subunit 473 111-116
214. QKCDLV C4LSS9 C4LSS9_ENTHI [tr] Tyrosine kinase putative 1754 1293-1298
215. DLVTTG C4MAS1 C4MAS1_ENTHI [tr] Uncharacterized protein 298 168-173
216. VSLSIL C4LUG5 C4LUG5_ENTHI [tr] Phospholipid-transporting ATPase 1068 904-909
217. LSILKS C4LXM3 C4LXM3_ENTHI [tr] Serine-threonine-isoleucine rich protein putative 5069 632-637
218. LKSHEN C4M2V0 C4M2V0_ENTHI [tr] DNAj domain containing protein 340 316-321
219. LKSHEN C4MB12 C4MB12_ENTHI [tr] Uncharacterized protein 828 300-305
220. KSHENG C4MB12 C4MB12_ENTHI [tr] Uncharacterized protein 828 301-306
221. TLTFEN C4M670 C4M670_ENTHI [tr] Protein kinase domain containing protein 482 94-99
222. VAGGIV Q1EQ03 Q1EQ03_ENTHI [tr] EhSyntaxin B 232 206-211
223. VAGGIV S0AWU0 S0AWU0_ENTHI [tr] Uncharacterized protein 232 206-211
224. IVAGIF C4LUY4 C4LUY4_ENTHI [tr] 3-ketoacyl-CoA synthase 487 84-89
225. IFLIFI C4M0B8 C4M0B8_ENTHI [tr] Tyrosine kinase putative 1970 1544-1549
226. LIFIEI C4LYL1 C4LYL1_ENTHI [tr] Uncharacterized protein 962 281-286
227. IFIEIA Q5NT44 Q5NT44_ENTHI [tr] Small GTP binding protein Rab7 putative (Fragment) 204 164-169
228. LAFAAV C4M3A6 C4M3A6_ENTHI [tr] Uncharacterized protein 428 5-10
229. EPDPKK C4LT76 C4LT76_ENTHI [tr] Uncharacterized protein 582 48-53
230. DPKKKA C4M822 C4M822_ENTHI [tr] Uncharacterized protein 484 475-480
231. PKKKAT C4M7M0 C4M7M0_ENTHI [tr] Uncharacterized protein 493 483-488
232. AITSTL A0A175JH31 A0A175JH31_ENTHI [tr] Rhogap domain containing protein 470 356-361
233. AITSTL B1N2T0 B1N2T0_ENTHI [tr] RhoGAP domain containing protein 479 356-361
234. ASSFKR C4LSG4 C4LSG4_ENTHI [tr] Uncharacterized protein 440 364-369
235. SSFKRR C4M216 C4M216_ENTHI [tr] Uncharacterized protein 1322 1244-1249
236. GALQNQ A0A175JG84 A0A175JG84_ENTHI [tr] ATP-dependent 6-phosphofructokinase 436 308-313
237. GALQNQ C4LYN5 C4LYN5_ENTHI [tr] DNA helicase 682 80-85
238. GALQNQ Q27651 PFKA_ENTHI [sp] ATP-dependent 6-phosphofructokinase 436 308-313
239. ALQNQK A0A175JG84 A0A175JG84_ENTHI [tr] ATP-dependent 6-phosphofructokinase 436 309-314
240. ALQNQK Q27651 PFKA_ENTHI [sp] ATP-dependent 6-phosphofructokinase 436 309-314

**NMDA 2A**

1. LVLPAL C4LTE9 C4LTE9_ENTHI [tr] Importin alpha subunit putative 478 271-276
2. AAAEKG A0A060N103 A0A060N103_ENTHI [tr] Enolase, putative 436 120-125
3. AAAEKG A0A060N147 A0A060N147_ENTHI [tr] Enolase, putative 436 120-125
4. AAAEKG A0A060N1F3 A0A060N1F3_ENTHI [tr] Enolase, putative 436 120-125
5. AAAEKG A0A060N4C3 A0A060N4C3_ENTHI [tr] Enolase, putative 436 120-125
6. AAAEKG A0A060N4N6 A0A060N4N6_ENTHI [tr] Enolase, putative 436 120-125
7. AAAEKG A0A060N4P1 A0A060N4P1_ENTHI [tr] Enolase, putative 436 120-125
8. AAAEKG A0A060N4Y0 A0A060N4Y0_ENTHI [tr] Enolase, putative 436 120-125
9. AAAEKG A0A060N511 A0A060N511_ENTHI [tr] Enolase, putative 436 120-125
10. AAAEKG A0A060N5K4 A0A060N5K4_ENTHI [tr] Enolase, putative 436 120-125
11. AAAEKG C4LXE8 C4LXE8_ENTHI [tr] Enolase putative 436 120-125
12. AAAEKG P51555 ENO1_ENTHI [sp] Enolase 1 436 120-125
13. AAAEKG S0AVX3 S0AVX3_ENTHI [tr] Enolase, putative 436 120-125
14. AAAEKG S0AW06 S0AW06_ENTHI [tr] Enolase, putative 436 120-125
15. AAAEKG S0AW15 S0AW15_ENTHI [tr] Enolase, putative 436 120-125
16. AAAEKG S0AWN3 S0AWN3_ENTHI [tr] Enolase, putative 436 120-125
17. AAAEKG S0AX43 S0AX43_ENTHI [tr] Enolase, putative 436 120-125
18. AAAEKG S0AXI3 S0AXI3_ENTHI [tr] Enolase, putative 436 120-125
19. AAAEKG S0AXP8 S0AXP8_ENTHI [tr] Enolase, putative 436 120-125
20. AAAEKG S0AYF3 S0AYF3_ENTHI [tr] Enolase, putative 436 120-125
21. AAAEKG S0AYJ0 S0AYJ0_ENTHI [tr] Enolase, putative 436 120-125
22. AAAEKG S0AYY5 S0AYY5_ENTHI [tr] Enolase, putative 436 120-125
23. AAAEKG S0AZF9 S0AZF9_ENTHI [tr] Enolase, putative 436 120-125
24. AAAEKG S0AZV8 S0AZV8_ENTHI [tr] Enolase, putative 436 120-125
25. AAAEKG S0B0A5 S0B0A5_ENTHI [tr] Enolase, putative 436 120-125
26. ALNIAV A0A175JEI8 A0A175JEI8_ENTHI [tr] AAA family ATPase putative 611 453-458
27. ALNIAV C4LTC0 C4LTC0_ENTHI [tr] AAA family ATPase, putative 611 453-458
28. SHDVTE C4M934 C4M934_ENTHI [tr] Phospholipase b putative 509 352-357
29. VTEREL B1N2P9 B1N2P9_ENTHI [tr] Phospholipid-transporting ATPase 1063 310-315
30. LDVNVV C4M142 C4M142_ENTHI [tr] N-system amino acid transporter 1 putative 699 48-53
31. VNVVAL C4M1F1 C4M1F1_ENTHI [tr] Calcium-transporting ATPase 1026 784-789
32. TDPKSL A0A175JGH8 A0A175JGH8_ENTHI [tr] Centromeric protein e putative 1056 573-578
33. KSLITH C4LZJ1 C4LZJ1_ENTHI [tr] Rap ran GTPase-activating protein putative 778 547-552
34. PTSTFF C4M893 C4M893_ENTHI [tr] Uncharacterized protein 190 10-15
35. LVTTIF C4M127 C4M127_ENTHI [tr] Uncharacterized protein 449 321-326
36. SFVKTT A0A060N262 A0A060N262_ENTHI [tr] Uncharacterized protein 208 113-118
37. SFVKTT C4M269 C4M269_ENTHI [tr] Uncharacterized protein 208 113-118
38. KTTVDN C4M2D1 C4M2D1_ENTHI [tr] Uncharacterized protein 517 44-49
39. TVDNSF C4M2Y2 C4M2Y2_ENTHI [tr] SNF7 family protein 205 8-13
40. VITLDT C4LWQ5 C4LWQ5_ENTHI [tr] Uncharacterized protein 461 130-135
41. ITLDTS C4LYT4 C4LYT4_ENTHI [tr] Ran binding protein putative 1044 497-502
42. VQLKKI C4M1F3 C4M1F3_ENTHI [tr] Uncharacterized protein 292 49-54
43. QLKKIH C4LYT4 C4LYT4_ENTHI [tr] Ran binding protein putative 1044 645-650
44. QLKKIH C4M9M5 C4M9M5_ENTHI [tr] Serine/threonine-protein phosphatase 311 189-194
45. SSVILL C4LT60 C4LT60_ENTHI [tr] Helicase putative 811 539-544
46. SSVILL C4LUD4 C4LUD4_ENTHI [tr] Uncharacterized protein 722 240-245
47. LGLTGY O15609 O15609_ENTHI [tr] MRNA, partial cds, Eh-EST144 (Fragment) 127 106-111
48. FFWIVP A0A175JKT5 A0A175JKT5_ENTHI [tr] Palmitoyltransferase 268 38-43
49. FWIVPS A0A175JKT5 A0A175JKT5_ENTHI [tr] Palmitoyltransferase 268 39-44
50. IVPSLV A0A175JFN4 A0A175JFN4_ENTHI [tr] Uncharacterized protein 597 180-185
51. IVPSLV C4LUU7 C4LUU7_ENTHI [tr] Uncharacterized protein 579 162-167
52. VSGNTE A0A175JXF9 A0A175JXF9_ENTHI [tr] Uncharacterized protein 392 138-143
53. VSGNTE B1N4D5 B1N4D5_ENTHI [tr] Uncharacterized protein 426 172-177
54. VSGNTE B1N5S4 B1N5S4_ENTHI [tr] Uncharacterized protein 418 228-233
55. VSGNTE C4LYE4 C4LYE4_ENTHI [tr] Uncharacterized protein 482 228-233
56. VSGNTE S0AXW7 S0AXW7_ENTHI [tr] Uncharacterized protein 482 228-233
57. TELIPK B1N2J8 B1N2J8_ENTHI [tr] Patatin, putative 538 510-515
58. IPKEFP C4M8E6 C4M8E6_ENTHI [tr] Chromodomain-helicase-DNA-binding protein putative 1247 1208-1213
59. PKEFPS C4M8E6 C4M8E6_ENTHI [tr] Chromodomain-helicase-DNA-binding protein putative 1247 1209-1214
60. GIGILT C4M2U1 C4M2U1_ENTHI [tr] Isoprenylated v-SNARE protein, putative 207 81-86
61. TTAASS C4M9R8 C4M9R8_ENTHI [tr] Protein tyrosine kinase domain-containing protein 1072 754-759
62. SMLEKF B1N304 B1N304_ENTHI [tr] Protein kinase putative 284 60-65
63. EKFSYI C4M1C5 C4M1C5_ENTHI [tr] F-box WD domain containing protein 631 2-7
64. NVTWDG C4M731 C4M731_ENTHI [tr] Uncharacterized protein 886 294-299
65. KDLSFT C4LTK1 C4LTK1_ENTHI [tr] Uncharacterized protein 319 147-152
66. LSFTEE C4LZB1 C4LZB1_ENTHI [tr] Uncharacterized protein 648 140-145
67. TEEGYQ C4M1M7 C4M1M7_ENTHI [tr] Acetyltransferase gnat family 170 22-27
68. LNKDRE C4LSU3 C4LSU3_ENTHI [tr] 60S ribosomal protein L26 putative 213 109-114
69. LNKDRE C4M569 C4M569_ENTHI [tr] 60S ribosomal protein L26 putative 213 109-114
70. HLSIVT A0A175JJ12 A0A175JJ12_ENTHI [tr] Uncharacterized protein 1256 344-349
71. HLSIVT C4LXQ2 C4LXQ2_ENTHI [tr] Uncharacterized protein 1256 344-349
72. VIVEDI C4LTR3 C4LTR3_ENTHI [tr] Uncharacterized protein 510 458-463
73. IVEDID C4LTR3 C4LTR3_ENTHI [tr] Uncharacterized protein 510 459-464
74. ETCVRN C4M5R7 C4M5R7_ENTHI [tr] Uncharacterized protein 237 127-132
75. VKINNS C4M4E3 C4M4E3_ENTHI [tr] Cullin family protein 721 614-619
76. VKINNS C4M8U0 C4M8U0_ENTHI [tr] Uncharacterized protein 1897 1237-1242
77. KINNST C4LSL0 C4LSL0_ENTHI [tr] Uncharacterized protein 285 124-129
78. INNSTN A0A175JMV1 A0A175JMV1_ENTHI [tr] Uncharacterized protein 624 312-317
79. INNSTN C4M0M7 C4M0M7_ENTHI [tr] Uncharacterized protein 604 292-297
80. NNSTNE C4LYC1 C4LYC1_ENTHI [tr] Uncharacterized protein 360 232-237
81. EGMNVK C4M7C7 C4M7C7_ENTHI [tr] Uncharacterized protein 959 857-862
82. FCIDIL C4M2M3 C4M2M3_ENTHI [tr] Uncharacterized protein 547 409-414
83. IDILKK C4LYW2 C4LYW2_ENTHI [tr] Uncharacterized protein 726 497-502
84. IDILKK C4M7N5 C4M7N5_ENTHI [tr] Uncharacterized protein 471 139-144
85. DILKKL C4LXK6 C4LXK6_ENTHI [tr] Dopey domain protein putative 1660 993-998
86. ILKKLS C4LYV4 C4LYV4_ENTHI [tr] Uncharacterized protein 519 92-97
87. LSRTVK A0A175JK68 A0A175JK68_ENTHI [tr] Uncharacterized protein 601 239-244
88. LSRTVK C4LZ19 C4LZ19_ENTHI [tr] Uncharacterized protein 601 239-244
89. VKFTYD C4LUT0 C4LUT0_ENTHI [tr] HAD hydrolase, family IA, variant 3 229 40-45
90. TYDLYL C4M049 C4M049_ENTHI [tr] Aminopeptidase putative 435 195-200
91. LYLVTN C4LXW3 C4LXW3_ENTHI [tr] Cyclin domain containing protein 398 154-159
92. LYLVTN S0AVK0 S0AVK0_ENTHI [tr] Cyclin, domain containing protein 366 122-127
93. YLVTNG C4M1L0 C4M1L0_ENTHI [tr] Uncharacterized protein 273 72-77
94. LVTNGK C4M1L0 C4M1L0_ENTHI [tr] Uncharacterized protein 273 73-78
95. HGKKVN C4M420 C4M420_ENTHI [tr] Protein kinase domain containing protein 1092 175-180
96. LTINEE C4LTX8 C4LTX8_ENTHI [tr] Leucine rich repeat protein bspa family 996 716-721
97. LTINEE C4M670 C4M670_ENTHI [tr] Protein kinase domain containing protein 482 364-369
98. RSEVVD C4LXI3 C4LXI3_ENTHI [tr] DNA-directed RNA polymerase II subunit putative 180 90-95
99. GTVSPS C4M471 C4M471_ENTHI [tr] Kinesin-like protein 629 315-320
100. PSAFLE C4LZI6 C4LZI6_ENTHI [tr] Tyrosine kinase putative 2577 170-175
101. FLEPFS C4M0H6 C4M0H6_ENTHI [tr] Uncharacterized protein 370 77-82
102. FLEPFS S0AWD3 S0AWD3_ENTHI [tr] Uncharacterized protein 370 77-82
103. RNLAKG A0A060N0E3 A0A060N0E3_ENTHI [tr] Fructose-1,6-bisphosphate aldolase, putative 330 73-78
104. RNLAKG A0A060N1Y8 A0A060N1Y8_ENTHI [tr] Fructose-1,6-bisphosphate aldolase, putative 330 73-78
105. RNLAKG A0A175JWA5 A0A175JWA5_ENTHI [tr] Fructose 1 6-bisphosphate aldolase putative 223 73-78
106. RNLAKG C4LXD7 C4LXD7_ENTHI [tr] Fructose 1 6-bisphosphate aldolase putative 330 73-78
107. RNLAKG Q8MTW2 Q8MTW2_ENTHI [tr] Putative fructose-1,6-bisphosphate aldolase (Fragment) 326 69-74
108. RNLAKG S0AUQ3 S0AUQ3_ENTHI [tr] Fructose-1,6-bisphosphate aldolase, putative 330 73-78
109. RNLAKG S0AWU7 S0AWU7_ENTHI [tr] Fructose-1,6-bisphosphate aldolase, putative 330 73-78
110. APHGPS C4LZN4 C4LZN4_ENTHI [tr] Ribosome biogenesis protein putative 291 97-102
111. APHGPS S0AWR7 S0AWR7_ENTHI [tr] Ribosome biogenesis protein, putative 291 97-102
112. TIGKAI C4M229 C4M229_ENTHI [tr] Uncharacterized protein 496 264-269
113. NSVPVQ C4LZR6 C4LZR6_ENTHI [tr] Sec7 domain protein 1660 574-579
114. FFAVIF A0A175JI07 A0A175JI07_ENTHI [tr] Uncharacterized protein 273 202-207
115. FLASYT C4M2H2 C4M2H2_ENTHI [tr] Uncharacterized protein 479 200-205
116. TVPNGS C4MAN9 C4MAN9_ENTHI [tr] Protein kinase putative 779 313-318
117. GVEDAL A0A175JI91 A0A175JI91_ENTHI [tr] Uncharacterized protein 266 49-54
118. GVEDAL C4LXD3 C4LXD3_ENTHI [tr] Uncharacterized protein 261 49-54
119. VEDALV A0A175JI91 A0A175JI91_ENTHI [tr] Uncharacterized protein 266 50-55
120. VEDALV C4LXD3 C4LXD3_ENTHI [tr] Uncharacterized protein 261 50-55
121. DALVSL C4M5P2 C4M5P2_ENTHI [tr] Uncharacterized protein 930 179-184
122. ALVSLK S0AV27 S0AV27_ENTHI [tr] 60S ribosomal protein L3, putative 402 169-174
123. LVTIGS A0A175JT87 A0A175JT87_ENTHI [tr] Rhogap domain containing protein 605 398-403
124. LVTIGS C4M676 C4M676_ENTHI [tr] RhoGAP domain containing protein 509 302-307
125. IFATTG Q1EQ51 Q1EQ51_ENTHI [tr] EhSec31 998 266-271
126. TTGYGI A0A060N124 A0A060N124_ENTHI [tr] Serine/threonine-protein phosphatase 433 33-38
127. TTGYGI C4M672 C4M672_ENTHI [tr] Serine/threonine-protein phosphatase 433 33-38
128. TGYGIA A0A060N124 A0A060N124_ENTHI [tr] Serine/threonine-protein phosphatase 433 34-39
129. TGYGIA C4M672 C4M672_ENTHI [tr] Serine/threonine-protein phosphatase 433 34-39
130. YGIALQ C4LUG0 C4LUG0_ENTHI [tr] Adaptor protein (AP) family protein 735 473-478
131. YGIALQ Q1EQ18 Q1EQ18_ENTHI [tr] Adaptor protein p family protein 767 505-510
132. GIALQK C4M7Z2 C4M7Z2_ENTHI [tr] Uncharacterized protein 919 36-41
133. EMEELE C4LVW8 C4LVW8_ENTHI [tr] PH-protein kinase domain containing protein 441 156-161
134. EELETL C4MAI8 C4MAI8_ENTHI [tr] Uncharacterized protein 382 29-34
135. HNEKNE C4M5M6 C4M5M6_ENTHI [tr] Coronin 1602 981-986
136. HNEKNE Q9XYQ0 Q9XYQ0_ENTHI [tr] Coronin 1602 981-986
137. NEKNEV C4M7A0 C4M7A0_ENTHI [tr] Uncharacterized protein 888 8-13
138. LSLITF C4LVE4 C4LVE4_ENTHI [tr] Uncharacterized protein 293 164-169
139. IEEKKK B1N492 B1N492_ENTHI [tr] Uncharacterized protein 87 57-62
140. IEEKKK C4LVH1 C4LVH1_ENTHI [tr] Diaphanous protein, homolog 2, putative 986 315-320
141. IEEKKK C4LW71 C4LW71_ENTHI [tr] DNA mismatch repair protein PMS1, putative 876 527-532
142. IEEKKK C4M0A7 C4M0A7_ENTHI [tr] Uncharacterized protein 164 26-31
143. IEEKKK C4M5M8 C4M5M8_ENTHI [tr] Sedlin putative 137 47-52
144. IEEKKK C4M8V1 C4M8V1_ENTHI [tr] Cysteinyl-tRNA synthetase putative 701 588-593
145. IEEKKK C4MAI4 C4MAI4_ENTHI [tr] Dual specificity protein phosphatase putative 336 74-79
146. EEKKKS C4LTB3 C4LTB3_ENTHI [tr] Uncharacterized protein 179 69-74
147. EEKKKS C4M0A7 C4M0A7_ENTHI [tr] Uncharacterized protein 164 27-32
148. EEKKKS C4M518 C4M518_ENTHI [tr] Helicase putative 956 637-642
149. EEKKKS C4M7R0 C4M7R0_ENTHI [tr] Uncharacterized protein 695 393-398
150. LKLLRS C4M3E6 C4M3E6_ENTHI [tr] DNA-directed RNA polymerase subunit 1570 102-107
151. KLLRSA C4M3I5 C4M3I5_ENTHI [tr] Uncharacterized protein 628 147-152
152. LLRSAK A0A175JX21 A0A175JX21_ENTHI [tr] Uncharacterized protein 66 21-26
153. KNISSM C4M4C5 C4M4C5_ENTHI [tr] Structure-specific endonuclease subunit SLX1 homolog 308 3-8
154. NRQKDN C4LZ42 C4LZ42_ENTHI [tr] 40S ribosomal protein S18, putative 155 86-91
155. NRQKDN C4M367 C4M367_ENTHI [tr] 40S ribosomal protein S18, putative 143 74-79
156. NRQKDN P48151 RS18_ENTHI [sp] 40S ribosomal protein S18 155 86-91
157. TLNESN A0A175JI84 A0A175JI84_ENTHI [tr] Uncharacterized protein 2034 1051-1056
158. TLNESN C4LWT5 C4LWT5_ENTHI [tr] Uncharacterized protein 2005 1022-1027
159. TLNESN C4M3J8 C4M3J8_ENTHI [tr] START domain containing protein 225 90-95
160. TLNESN S0AZF3 S0AZF3_ENTHI [tr] START domain containing protein 225 90-95
161. SNPNTV C4M6S8 C4M6S8_ENTHI [tr] Uncharacterized protein 1372 35-40
162. ESKANS C4LXX2 C4LXX2_ENTHI [tr] Uncharacterized protein 265 39-44
163. KKSVDS C4M110 C4M110_ENTHI [tr] Serine threonine protein kinase 6 putative 319 31-36
164. KSVDSI B1N2R6 B1N2R6_ENTHI [tr] DNA polymerase epsilon subunit b domain-containing protein 395 90-95
165. IRQDSL C4M7K5 C4M7K5_ENTHI [tr] Uncharacterized protein 980 518-523
166. DEATAE A0A175JR33 A0A175JR33_ENTHI [tr] Uncharacterized protein 2474 2309-2314
167. DEATAE C4M3H1 C4M3H1_ENTHI [tr] Uncharacterized protein 2457 2292-2297
168. SLKSPR A0A175JVH0 A0A175JVH0_ENTHI [tr] Rhogap domain containing protein 523 95-100
169. SLKSPR A0A175JXB2 A0A175JXB2_ENTHI [tr] Rhogap domain containing protein 526 95-100
170. SLKSPR B1N4I0 B1N4I0_ENTHI [tr] RhoGAP domain containing protein 446 15-20
171. SLKSPR C4M768 C4M768_ENTHI [tr] RhoGAP domain containing protein 443 15-20
172. LPEEMA A0A175JJD9 A0A175JJD9_ENTHI [tr] Uncharacterized protein 237 127-132
173. LPEEMA A0A175K0H1 A0A175K0H1_ENTHI [tr] Swirm domain protein 400 127-132
174. LPEEMA B1N2Y2 B1N2Y2_ENTHI [tr] Uncharacterized protein 238 127-132
175. LPEEMA C4MB44 C4MB44_ENTHI [tr] SWIRM domain protein 400 127-132
176. DISETS C4M543 C4M543_ENTHI [tr] Leucine-rich repeat domain-containing protein 1894 883-888
177. NHKTKD A0A175JFT6 A0A175JFT6_ENTHI [tr] Uncharacterized protein 425 174-179
178. NHKTKD C4LV57 C4LV57_ENTHI [tr] Uncharacterized protein 425 174-179
179. HKTKDN A0A175JFT6 A0A175JFT6_ENTHI [tr] Uncharacterized protein 425 175-180
180. HKTKDN C4LV57 C4LV57_ENTHI [tr] Uncharacterized protein 425 175-180
181. KTKSSS C4MBA6 C4MBA6_ENTHI [tr] Uncharacterized protein 312 199-204
182. TKSSSP C4MBH3 C4MBH3_ENTHI [tr] CDP-alcohol phosphatidyltransferase family protein 383 113-118
183. PPQFVE C4M8G1 C4M8G1_ENTHI [tr] Uncharacterized protein 1020 986-991
184. FVENVT Q761X3 Q761X3_ENTHI [tr] Serine threonine protein kinase putative 599 236-241
185. PLHNEE C4M546 C4M546_ENTHI [tr] Protein kinase putative 974 138-143
186. LHNEEG C4M281 C4M281_ENTHI [tr] Uncharacterized protein 656 478-483
187. YKLYSK C4M8P7 C4M8P7_ENTHI [tr] Apyrase putative 352 49-54
188. FTLKDK A0A175JXN7 A0A175JXN7_ENTHI [tr] Protein kinase domain containing protein 787 388-393
189. FTLKDK C4M957 C4M957_ENTHI [tr] Protein kinase domain containing protein 776 377-382
190. NLYDID C4M3P3 C4M3P3_ENTHI [tr] Actin putative 338 182-187
191. NLYDID C4M8Z3 C4M8Z3_ENTHI [tr] Uncharacterized protein 589 368-373
192. DIDEDQ C4LTU5 C4LTU5_ENTHI [tr] Leucine rich repeat / protein phosphatase 2C domain containing protein 837 555-560
193. NPATGE Q5NT22 Q5NT22_ENTHI [tr] Rab family GTPase (Fragment) 233 219-224
194. LQLQKN C4M1F9 C4M1F9_ENTHI [tr] Uncharacterized protein 1177 821-826
195. PRELDL C4LTF7 C4LTF7_ENTHI [tr] Ubiquitin carboxyl-terminal hydrolase domain containing protein 1477 519-524
196. SRSISL A0A175JUS6 A0A175JUS6_ENTHI [tr] Uncharacterized protein 2426 1268-1273
197. SRSISL C4M6M0 C4M6M0_ENTHI [tr] Uncharacterized protein 2415 1257-1262
198. SISLKD C4M020 C4M020_ENTHI [tr] Uncharacterized protein 215 164-169
199. SISLKD C4M6S8 C4M6S8_ENTHI [tr] Uncharacterized protein 1372 83-88
200. DRERLL A0A175JUB5 A0A175JUB5_ENTHI [tr] AP complex subunit beta 855 551-556
201. DRERLL B1N424 B1N424_ENTHI [tr] AP complex subunit beta 855 551-556
202. NFYGSL C4M9B4 C4M9B4_ENTHI [tr] Uncharacterized protein 338 213-218
203. FYGSLF C4M6X4 C4M6X4_ENTHI [tr] Ser Thr protein phosphatase family protein 224 109-114
204. FYGSLF C4M9B4 C4M9B4_ENTHI [tr] Uncharacterized protein 338 214-219
205. LFSVPS C4LTF7 C4LTF7_ENTHI [tr] Ubiquitin carboxyl-terminal hydrolase domain containing protein 1477 230-235
206. PSSKLS A0A175JT28 A0A175JT28_ENTHI [tr] Sec7 domain containing protein 1416 13-18
207. PSSKLS C4M5V4 C4M5V4_ENTHI [tr] Sec7 domain protein 1396 13-18
208. SSKLSG C4M6V3 C4M6V3_ENTHI [tr] Ras guanine nucleotide exchange factor putative 850 109-114
209. SKLSGK C4LV26 C4LV26_ENTHI [tr] Uncharacterized protein 1116 200-205
210. KLSGKK A0A175K209 A0A175K209_ENTHI [tr] Calponin-homology domain containing protein 353 48-53
211. KLSGKK C4LZD1 C4LZD1_ENTHI [tr] Protein with RhoGEF and ArfGAP domains 1098 43-48
212. KLSGKK Q9U3Z8 Q9U3Z8_ENTHI [tr] Actinin-like protein (Fragment) 537 49-54
213. KKSSLF C4M3D0 C4M3D0_ENTHI [tr] Uncharacterized protein 440 227-232
214. KKSSLF C4M5X2 C4M5X2_ENTHI [tr] Lipid phosphate phosphatase putative 259 89-94
215. RSKSLL B1N2W3 B1N2W3_ENTHI [tr] Cleavage stimulation factor putative 550 224-229
216. RSKSLL C4M1C9 C4M1C9_ENTHI [tr] Cleavage stimulation factor putative 550 224-229
217. HRDDQR C4M108 C4M108_ENTHI [tr] Phospholipid-transporting P-type ATPase, putative 982 96-101
218. SDPYKH C4M4L9 C4M4L9_ENTHI [tr] Uncharacterized protein 239 67-72
219. SYLRSS C4M7E6 C4M7E6_ENTHI [tr] Uncharacterized protein 124 90-95
220. RSSLRS A0A175JR82 A0A175JR82_ENTHI [tr] Uncharacterized protein 477 302-307
221. RSSLRS B1N3P1 B1N3P1_ENTHI [tr] Uncharacterized protein 352 177-182
222. RSSLRS C4M2W6 C4M2W6_ENTHI [tr] Uncharacterized protein 1357 1049-1054
223. RSSLRS C4M8X4 C4M8X4_ENTHI [tr] Uncharacterized protein 2524 2272-2277
224. VYISEH Q401L5 Q401L5_ENTHI [tr] Serine O-acetyltransferase 2 311 266-271
225. VYISEH Q9U8X2 Q9U8X2_ENTHI [tr] Serine acetyltransferase 305 266-271
226. SNRRVY C4LZD9 C4LZD9_ENTHI [tr] Leucine rich repeat / protein phosphatase 2C domain containing protein 833 795-800
227. KMPSIE C4M9L5 C4M9L5_ENTHI [tr] Uncharacterized protein 924 594-599

**NMDA 2B**

1. WLVLAV C4M462 C4M462_ENTHI [tr] Phospholipid-transporting ATPase 1335 1128-1133
2. AVLAVS A0A175JPZ2 A0A175JPZ2_ENTHI [tr] Calcium-transporting ATPase 1087 151-156
3. AVLAVS C4M2K1 C4M2K1_ENTHI [tr] Calcium-transporting ATPase 1087 151-156
4. AVLAVS Q27642 Q27642_ENTHI [tr] Calcium-transporting ATPase 1086 151-156
5. SIGIAV C4LSU8 C4LSU8_ENTHI [tr] Uncharacterized protein 533 96-101
6. IAVILV Q1EPY4 Q1EPY4_ENTHI [tr] Uncharacterized protein (Fragment) 252 85-90
7. VILVGT C4LZ62 C4LZ62_ENTHI [tr] Uncharacterized protein 197 21-26
8. ILVGTS C4M4A7 C4M4A7_ENTHI [tr] Cpsf a subunit region protein putative 1108 484-489
9. SDEVAI C4LVP3 C4LVP3_ENTHI [tr] Uncharacterized protein 1082 549-554
10. KDAHEK C4LSG4 C4LSG4_ENTHI [tr] Uncharacterized protein 440 215-220
11. DPKSII C4M924 C4M924_ENTHI [tr] DNA methyltransferase putative 210 169-174
12. PKSIIT A0A175JG52 A0A175JG52_ENTHI [tr] Uncharacterized protein 1004 134-139
13. PKSIIT C4LUZ0 C4LUZ0_ENTHI [tr] Uncharacterized protein 984 114-119
14. SIITRI A0A175JYB2 A0A175JYB2_ENTHI [tr] Uncharacterized protein 132 80-85
15. QEAIAQ A0A175JPW1 A0A175JPW1_ENTHI [tr] Uncharacterized protein 2546 176-181
16. QEAIAQ B1N3J3 B1N3J3_ENTHI [tr] Uncharacterized protein 2532 176-181
17. SAQTLT C4LX15 C4LX15_ENTHI [tr] Tyrosine kinase putative 1637 176-181
18. TLTPIL C4M6Q8 C4M6Q8_ENTHI [tr] HEAT repeat domain containing protein 1157 124-129
19. DKDESS Q56AY2 Q56AY2_ENTHI [tr] CAF1 family ribonuclease, putative 311 149-154
20. PSIEQQ A0A175JE12 A0A175JE12_ENTHI [tr] Poly polymerase putative 522 470-475
21. PSIEQQ C4M573 C4M573_ENTHI [tr] Importin alpha re-exporter putative 889 590-595
22. PSIEQQ Q51D88 PAP_ENTHI [sp] Poly(A) polymerase 522 470-475
23. IEQQAS C4M7I2 C4M7I2_ENTHI [tr] Rho guanine nucleotide exchange factor putative 337 270-275
24. YIFSIV C4LUX9 C4LUX9_ENTHI [tr] Protein YIPF 231 103-108
25. YIFSIV S0AV18 S0AV18_ENTHI [tr] Protein YIPF 231 103-108
26. QDFVNK C4M5A1 C4M5A1_ENTHI [tr] Sec6 protein putative 766 662-667
27. FVNKIR C4M2T6 C4M2T6_ENTHI [tr] S1 RNA binding domain-containing protein 1710 853-858
28. STIENS C4M998 C4M998_ENTHI [tr] CAAX prenyl protease putative 416 390-395
29. VLLLDM C4LXH1 C4LXH1_ENTHI [tr] Uncharacterized protein 1442 1415-1420
30. NQLKKL A0A175JLT0 A0A175JLT0_ENTHI [tr] Uncharacterized protein 321 145-150
31. NQLKKL B1N362 B1N362_ENTHI [tr] Uncharacterized protein 240 64-69
32. NQLKKL C4LZG7 C4LZG7_ENTHI [tr] Uncharacterized protein 106 42-47
33. NQLKKL C4M0Z3 C4M0Z3_ENTHI [tr] RNA recognition motif domain containing protein 379 202-207
34. QLKKLQ C4M8Z3 C4M8Z3_ENTHI [tr] Uncharacterized protein 589 9-14
35. LKKLQS C4LWI1 C4LWI1_ENTHI [tr] Ras GTPase-activating protein putative 449 286-291
36. LKKLQS C4LX13 C4LX13_ENTHI [tr] Cell division control protein CDC24, putative 749 507-512
37. LKKLQS C4M5D7 C4M5D7_ENTHI [tr] Acyl-coA synthetase, putative 1014 993-998
38. SPIILL A0A175JJZ5 A0A175JJZ5_ENTHI [tr] Uncharacterized protein 2653 555-560
39. SPIILL C4LY79 C4LY79_ENTHI [tr] Uncharacterized protein 3652 555-560
40. PIILLY C4M2Q5 C4M2Q5_ENTHI [tr] Uncharacterized protein 724 137-142
41. LLYCTK B1N3J4 B1N3J4_ENTHI [tr] Uncharacterized protein 510 272-277
42. NSVGLT A0A175JVB0 A0A175JVB0_ENTHI [tr] Uncharacterized protein 572 219-224
43. NSVGLT C4M821 C4M821_ENTHI [tr] Uncharacterized protein 562 209-214
44. SVGLTG C4LWH2 C4LWH2_ENTHI [tr] WD domain containing protein 593 60-65
45. GLTGYG O15609 O15609_ENTHI [tr] MRNA, partial cds, Eh-EST144 (Fragment) 127 107-112
46. IVPSLV A0A175JFN4 A0A175JFN4_ENTHI [tr] Uncharacterized protein 597 180-185
47. IVPSLV C4LUU7 C4LUU7_ENTHI [tr] Uncharacterized protein 579 162-167
48. DTDTVP A0A175JQR9 A0A175JQR9_ENTHI [tr] Rab family GTPase 203 183-188
49. DTDTVP Q5NT13 Q5NT13_ENTHI [tr] Rab family GTPase (Fragment) 203 183-188
50. SVSYDE B1N452 B1N452_ENTHI [tr] Uncharacterized protein 444 37-42
51. IAIITT C4M5U8 C4M5U8_ENTHI [tr] CXXC-rich protein 1035 531-536
52. AIITTA C4LTW0 C4LTW0_ENTHI [tr] N-system amino acid transporter 1 putative 674 300-305
53. SDMLSE C4LZI5 C4LZI5_ENTHI [tr] Uncharacterized protein 628 209-214
54. MLSEHS C4LUA8 C4LUA8_ENTHI [tr] Leucine rich repeat protein bspa family 843 318-323
55. LSEHSF C4LUA8 C4LUA8_ENTHI [tr] Leucine rich repeat protein bspa family 843 319-324
56. KSSCYN C4M1X9 C4M1X9_ENTHI [tr] Ras guanine nucleotide exchange factor putative 970 952-957
57. KRIYQS A0A175JG96 A0A175JG96_ENTHI [tr] Uncharacterized protein 615 32-37
58. KRIYQS C4LUW1 C4LUW1_ENTHI [tr] Uncharacterized protein 1070 32-37
59. EGRNLS C4LTU5 C4LTU5_ENTHI [tr] Leucine rich repeat / protein phosphatase 2C domain containing protein 837 579-584
60. LSFSED B1N3U9 B1N3U9_ENTHI [tr] Zinc finger domain containing protein 195 111-116
61. LSFSED C4M1S1 C4M1S1_ENTHI [tr] Zinc finger domain containing protein 219 135-140
62. LVIILL C4M9Q1 C4M9Q1_ENTHI [tr] Uncharacterized protein 222 97-102
63. IILLNK C4LSI3 C4LSI3_ENTHI [tr] Uncharacterized protein 87 47-52
64. ILLNKE C4LU65 C4LU65_ENTHI [tr] Vacuolar ATP synthase subunit H, putative 444 184-189
65. LLNKER A0A175JGS9 A0A175JGS9_ENTHI [tr] Uncharacterized protein 1780 1734-1739
66. LLNKER C4LVB6 C4LVB6_ENTHI [tr] Uncharacterized protein 1755 1709-1714
67. LNKERK C4LTL9 C4LTL9_ENTHI [tr] Uncharacterized protein 134 64-69
68. PRMCPE C4LUZ3 C4LUZ3_ENTHI [tr] Uncharacterized protein 1231 372-377
69. EEQEDD C4LY24 C4LY24_ENTHI [tr] Uncharacterized protein 1151 1119-1124
70. HLSIVT A0A175JJ12 A0A175JJ12_ENTHI [tr] Uncharacterized protein 1256 344-349
71. HLSIVT C4LXQ2 C4LXQ2_ENTHI [tr] Uncharacterized protein 1256 344-349
72. FVIVES C4M107 C4M107_ENTHI [tr] Vacular protein sorting 33A, putative 995 451-456
73. FVIVES Q1EPY0 Q1EPY0_ENTHI [tr] EhVps33 661 117-122
74. IVESVD C4LWT2 C4LWT2_ENTHI [tr] Protein phosphatase domain-containing protein 333 18-23
75. RIVTEN A0A175JZK5 A0A175JZK5_ENTHI [tr] Aig family protein 414 314-319
76. RIVTEN C4M7Z0 C4M7Z0_ENTHI [tr] Ubiquitin carboxyl-terminal hydrolase domain containing protein 1295 959-964
77. IVTENK A0A175JZK5 A0A175JZK5_ENTHI [tr] Aig family protein 414 315-320
78. ENKTDE C4LT04 C4LT04_ENTHI [tr] Long-chain-fatty-acid--CoA ligase, putative 642 564-569
79. NKTDEE C4LT04 C4LT04_ENTHI [tr] Long-chain-fatty-acid--CoA ligase, putative 642 565-570
80. PGYIKK C4M4X7 C4M4X7_ENTHI [tr] Palmitoyltransferase 352 113-118
81. FCIDIL C4M2M3 C4M2M3_ENTHI [tr] Uncharacterized protein 547 409-414
82. IDILKK C4LYW2 C4LYW2_ENTHI [tr] Uncharacterized protein 726 497-502
83. IDILKK C4M7N5 C4M7N5_ENTHI [tr] Uncharacterized protein 471 139-144
84. ILKKIS B1N525 B1N525_ENTHI [tr] Uncharacterized protein 367 360-365
85. ILKKIS C4MB83 C4MB83_ENTHI [tr] Uncharacterized protein 421 414-419
86. LKKISK A0A175JH29 A0A175JH29_ENTHI [tr] F-box domain containing protein putative 283 273-278
87. LKKISK C4M7F8 C4M7F8_ENTHI [tr] Regulator of nonsense transcripts putative 896 561-566
88. ISKSVK A0A175JGZ9 A0A175JGZ9_ENTHI [tr] Uncharacterized protein 504 464-469
89. ISKSVK C4LW24 C4LW24_ENTHI [tr] Uncharacterized protein 470 430-435
90. ISKSVK C4LWD2 C4LWD2_ENTHI [tr] Uncharacterized protein 549 135-140
91. SKSVKF C4LWD2 C4LWD2_ENTHI [tr] Uncharacterized protein 549 136-141
92. VKFTYD C4LUT0 C4LUT0_ENTHI [tr] HAD hydrolase, family IA, variant 3 229 40-45
93. TYDLYL C4M049 C4M049_ENTHI [tr] Aminopeptidase putative 435 195-200
94. LYLVTN C4LXW3 C4LXW3_ENTHI [tr] Cyclin domain containing protein 398 154-159
95. LYLVTN S0AVK0 S0AVK0_ENTHI [tr] Cyclin, domain containing protein 366 122-127
96. YLVTNG C4M1L0 C4M1L0_ENTHI [tr] Uncharacterized protein 273 72-77
97. LVTNGK C4M1L0 C4M1L0_ENTHI [tr] Uncharacterized protein 273 73-78
98. GKKING A0A175K0K4 A0A175K0K4_ENTHI [tr] 1-cys peroxiredoxin putative 144 133-138
99. GKKING B1N4B0 B1N4B0_ENTHI [tr] Uncharacterized protein 374 284-289
100. GKKING C4M7L8 C4M7L8_ENTHI [tr] Uncharacterized protein 375 284-289
101. NGTWNG C4LVY7 C4LVY7_ENTHI [tr] Glucosidase, putative 827 230-235
102. LTINEE C4LTX8 C4LTX8_ENTHI [tr] Leucine rich repeat protein bspa family 996 716-721
103. LTINEE C4M670 C4M670_ENTHI [tr] Protein kinase domain containing protein 482 364-369
104. RSEVVD C4LXI3 C4LXI3_ENTHI [tr] DNA-directed RNA polymerase II subunit putative 180 90-95
105. VPFIET C4M2M3 C4M2M3_ENTHI [tr] Uncharacterized protein 547 79-84
106. VPFIET C4M2S2 C4M2S2_ENTHI [tr] Uncharacterized protein 618 177-182
107. IETGIS C4LXJ5 C4LXJ5_ENTHI [tr] Uncharacterized protein 699 255-260
108. GTVSPS C4M471 C4M471_ENTHI [tr] Kinesin-like protein 629 315-320
109. PSAFLE C4LZI6 C4LZI6_ENTHI [tr] Tyrosine kinase putative 2577 170-175
110. FLEPFS C4M0H6 C4M0H6_ENTHI [tr] Uncharacterized protein 370 77-82
111. FLEPFS S0AWD3 S0AWD3_ENTHI [tr] Uncharacterized protein 370 77-82
112. FSADVW C4M9L7 C4M9L7_ENTHI [tr] Protein kinase domain containing protein 288 210-215
113. TIGKAI C4M229 C4M229_ENTHI [tr] Uncharacterized protein 496 264-269
114. NSVPVQ C4LZR6 C4LZR6_ENTHI [tr] Sec7 domain protein 1660 574-579
115. FFAVIF A0A175JI07 A0A175JI07_ENTHI [tr] Uncharacterized protein 273 202-207
116. FLASYT C4M2H2 C4M2H2_ENTHI [tr] Uncharacterized protein 479 200-205
117. TVPNGS C4MAN9 C4MAN9_ENTHI [tr] Protein kinase putative 779 313-318
118. DALLSL A0A175JLT4 A0A175JLT4_ENTHI [tr] Heat repeat domain containing protein 713 523-528
119. DALLSL C4M0I8 C4M0I8_ENTHI [tr] HEAT repeat domain containing protein 708 518-523
120. LLSLKT C4M3B4 C4M3B4_ENTHI [tr] Uncharacterized protein 830 177-182
121. LVTIGS A0A175JT87 A0A175JT87_ENTHI [tr] Rhogap domain containing protein 605 398-403
122. LVTIGS C4M676 C4M676_ENTHI [tr] RhoGAP domain containing protein 509 302-307
123. TIGSGK C4LYL2 C4LYL2_ENTHI [tr] HEAT repeat domain containing protein 1589 1165-1170
124. STGYGI Q1EQ46 Q1EQ46_ENTHI [tr] EhSec24C 876 690-695
125. TGYGIA A0A060N124 A0A060N124_ENTHI [tr] Serine/threonine-protein phosphatase 433 34-39
126. TGYGIA C4M672 C4M672_ENTHI [tr] Serine/threonine-protein phosphatase 433 34-39
127. DLAILQ A0A175JFN5 A0A175JFN5_ENTHI [tr] Uncharacterized protein 524 206-211
128. DLAILQ A0A175JX61 A0A175JX61_ENTHI [tr] Uncharacterized protein 524 206-211
129. DLAILQ B1N3K1 B1N3K1_ENTHI [tr] Uncharacterized protein 355 37-42
130. DLAILQ B1N5P4 B1N5P4_ENTHI [tr] Uncharacterized protein 355 37-42
131. DLAILQ C4LUH5 C4LUH5_ENTHI [tr] Uncharacterized protein 498 180-185
132. ILQLFG C4M6Z0 C4M6Z0_ENTHI [tr] Uncharacterized protein 459 31-36
133. EMEELE C4LVW8 C4LVW8_ENTHI [tr] PH-protein kinase domain containing protein 441 156-161
134. HNEKNE C4M5M6 C4M5M6_ENTHI [tr] Coronin 1602 981-986
135. HNEKNE Q9XYQ0 Q9XYQ0_ENTHI [tr] Coronin 1602 981-986
136. NEKNEV C4M7A0 C4M7A0_ENTHI [tr] Uncharacterized protein 888 8-13
137. MLGAAM Q24802 Q24802_ENTHI [tr] HM-1-IMSS putative vacuolar proton-transporting ATPase catalytic subunit 607 28-33
138. LSLITF C4LVE4 C4LVE4_ENTHI [tr] Uncharacterized protein 293 164-169
139. GVCSGK C4M6V0 C4M6V0_ENTHI [tr] Protein kinase domain containing protein 292 76-81
140. IEERQS C4M0Z9 C4M0Z9_ENTHI [tr] Uncharacterized protein 911 379-384
141. SNILRL C4M6S4 C4M6S4_ENTHI [tr] Uncharacterized protein 501 246-251
142. NILRLL C4M3K1 C4M3K1_ENTHI [tr] Uncharacterized protein 359 231-236
143. NILRLL C4M6S4 C4M6S4_ENTHI [tr] Uncharacterized protein 501 247-252
144. VNGSPQ C4M5S9 C4M5S9_ENTHI [tr] L-myo-inositol 1 phosphate synthase 508 273-278
145. VNGSPQ P90626 P90626_ENTHI [tr] L-myo-inositol-1-phosphate synthase 508 273-278
146. SALDFI C4M5M8 C4M5M8_ENTHI [tr] Sedlin putative 137 42-47
147. VYDISE C4LXE2 C4LXE2_ENTHI [tr] Uncharacterized protein 418 38-43
148. ISEVER B1N482 B1N482_ENTHI [tr] Uncharacterized protein 686 630-635
149. NVYQDH C4MA24 C4MA24_ENTHI [tr] Ubiquitin-like protein 5 putative 79 55-60
150. PHSIGS C4LWN5 C4LWN5_ENTHI [tr] SP-RING zinc finger domain containing protein 539 470-475
151. SSIDGL A0A175JF45 A0A175JF45_ENTHI [tr] Uncharacterized protein 567 436-441
152. SSIDGL B1N2L5 B1N2L5_ENTHI [tr] Uncharacterized protein 176 45-50
153. SSIDGL C4LUU3 C4LUU3_ENTHI [tr] Uncharacterized protein 256 110-115
154. SSIDGL Q1EQ36 Q1EQ36_ENTHI [tr] Coatomer protein gamma subunit putative 844 357-362
155. SISKKP C4LXU8 C4LXU8_ENTHI [tr] Cysteine protease binding protein family 2 871 296-301
156. SISKKP C4M748 C4M748_ENTHI [tr] Uncharacterized protein 854 166-171
157. SISKKP C4M9I2 C4M9I2_ENTHI [tr] SH3 domain protein 359 149-154
158. SISKKP S0AXC8 S0AXC8_ENTHI [tr] SH3 domain protein 359 149-154
159. IGLPSS C4M9T5 C4M9T5_ENTHI [tr] Uncharacterized protein 493 161-166
160. SKHSQL C4LT15 C4LT15_ENTHI [tr] Uncharacterized protein 298 149-154
161. SKHSQL C4M8K7 C4M8K7_ENTHI [tr] DNA repair helicase putative 770 128-133
162. HSQLSD A0A175JCV5 A0A175JCV5_ENTHI [tr] F-box WD domain containing protein 830 337-342
163. HSQLSD C4LSM0 C4LSM0_ENTHI [tr] F-box/WD domain containing protein 720 227-232
164. QLSDLY C4M963 C4M963_ENTHI [tr] Tyrosine kinase putative 1336 320-325
165. GKFSFK C4LTX8 C4LTX8_ENTHI [tr] Leucine rich repeat protein bspa family 996 926-931
166. KFSFKS C4M935 C4M935_ENTHI [tr] Protein kinase, putative 686 46-51
167. SFKSDR A0A175JFR9 A0A175JFR9_ENTHI [tr] Uncharacterized protein 600 17-22
168. SFKSDR C4LV38 C4LV38_ENTHI [tr] Uncharacterized protein 600 17-22
169. KSDRYS C4LY62 C4LY62_ENTHI [tr] Ras guanine nucleotide exchange factor putative 598 292-297
170. SDVSDI C4MAE3 C4MAE3_ENTHI [tr] F-box domain containing protein 799 380-385
171. DVSDIS C4MAE3 C4MAE3_ENTHI [tr] F-box domain containing protein 799 381-386
172. IEGNAA A0A175JX25 A0A175JX25_ENTHI [tr] Nucleoside transporter putative 407 107-112
173. IEGNAA C4M8V2 C4M8V2_ENTHI [tr] Nucleoside transporter, putative 379 79-84
174. RRKQQY C4M6F4 C4M6F4_ENTHI [tr] Rho guanine nucleotide exchange factor putative 1130 112-117
175. YKDSLK C4LUM7 C4LUM7_ENTHI [tr] Uncharacterized protein 657 607-612
176. YKDSLK C4M4Z7 C4M4Z7_ENTHI [tr] Ubiquitin carboxyl-terminal hydrolase domain containing protein 806 723-728
177. KDSLKK C4LY04 C4LY04_ENTHI [tr] Protein with RhoGEF and ArfGAP domains 1045 826-831
178. KDSLKK C4M4Z7 C4M4Z7_ENTHI [tr] Ubiquitin carboxyl-terminal hydrolase domain containing protein 806 724-729
179. DSLKKR A0A175JZS1 A0A175JZS1_ENTHI [tr] Uncharacterized protein 244 18-23
180. DSLKKR B1N575 B1N575_ENTHI [tr] Uncharacterized protein (Fragment) 218 18-23
181. DSLKKR C4LTW5 C4LTW5_ENTHI [tr] Uncharacterized protein 345 54-59
182. DSLKKR C4M5S1 C4M5S1_ENTHI [tr] Uncharacterized protein 722 56-61
183. FDEIEL C4M5U4 C4M5U4_ENTHI [tr] Protein tyrosine kinase domain-containing protein 1380 823-828
184. FDEIEL C4M762 C4M762_ENTHI [tr] Tyrosine kinase putative 1228 895-900
185. LRDFYL C4MAA1 C4MAA1_ENTHI [tr] Uncharacterized protein 108 22-27
186. RDFYLD C4MAA1 C4MAA1_ENTHI [tr] Uncharacterized protein 108 23-28
187. LDQFRT C4M8A1 C4M8A1_ENTHI [tr] Uncharacterized protein 232 102-107
188. TKENSP C4LXV8 C4LXV8_ENTHI [tr] Myotubularin putative 631 511-516
189. SVSGGG C4MBN7 C4MBN7_ENTHI [tr] Uncharacterized protein 460 190-195
190. PCTNRS C4LVP5 C4LVP5_ENTHI [tr] Uncharacterized protein 167 128-133
191. HGVVSG C4LZJ1 C4LZJ1_ENTHI [tr] Rap ran GTPase-activating protein putative 778 102-107
192. SGVPAP A0A060N1M7 A0A060N1M7_ENTHI [tr] Aldose reductase, putative 305 210-215
193. SGVPAP A0A175JWN7 A0A175JWN7_ENTHI [tr] Aldose reductase putative 305 210-215
194. SGVPAP C4M7W2 C4M7W2_ENTHI [tr] Aldose reductase putative 305 210-215
195. SGVPAP C4M8C2 C4M8C2_ENTHI [tr] Aldose reductase, putative 305 210-215
196. SCPSKL C4M3N7 C4M3N7_ENTHI [tr] Putative transcription enhancer protein 1175 289-294
197. NYSTTV C4LZV2 C4LZV2_ENTHI [tr] Rho family GTPase 211 38-43
198. YSTTVT C4LZV2 C4LZV2_ENTHI [tr] Rho family GTPase 211 39-44
199. TTVTGQ C4LSY5 C4LSY5_ENTHI [tr] Uncharacterized protein 291 110-115
200. DISEDN C4M702 C4M702_ENTHI [tr] Uncharacterized protein 423 87-92
201. EDNSLQ C4M337 C4M337_ENTHI [tr] Uncharacterized protein 749 262-267
202. VTSNAS C4M256 C4M256_ENTHI [tr] Protein kinase putative 1585 1474-1479
203. TSNAST C4LSW5 C4LSW5_ENTHI [tr] Uncharacterized protein 105 7-12
204. TSNAST C4M6H8 C4M6H8_ENTHI [tr] Mucin 2 putative 551 258-263
205. SNASTT C4M6H8 C4M6H8_ENTHI [tr] Mucin 2 putative 551 259-264
206. KYPQSP C4M463 C4M463_ENTHI [tr] GTPase activating protein putative 357 176-181
207. PQSPTN B1N4K3 B1N4K3_ENTHI [tr] Uncharacterized protein 379 278-283
208. PQSPTN B1N5A2 B1N5A2_ENTHI [tr] Uncharacterized protein 388 281-286
209. PQSPTN C4LXU9 C4LXU9_ENTHI [tr] Uncharacterized protein 388 281-286
210. PQSPTN C4LY25 C4LY25_ENTHI [tr] Uncharacterized protein 685 590-595
211. PQSPTN C4M706 C4M706_ENTHI [tr] Uncharacterized protein 379 278-283
212. PQSPTN C4M7N0 C4M7N0_ENTHI [tr] Uncharacterized protein 379 278-283
213. PQSPTN C4MBJ9 C4MBJ9_ENTHI [tr] Uncharacterized protein 388 281-286
214. KKNRNK C4M7H8 C4M7H8_ENTHI [tr] DNA mismatch repair protein mutS, putative 755 81-86
215. RNKLRR C4LYG4 C4LYG4_ENTHI [tr] Uncharacterized protein 425 364-369
216. NKLRRQ C4LYG4 C4LYG4_ENTHI [tr] Uncharacterized protein 425 365-370
217. VDLQKE A0A175K078 A0A175K078_ENTHI [tr] Uncharacterized protein 108 26-31
218. VDLQKE B1N5D9 B1N5D9_ENTHI [tr] Uncharacterized protein (Fragment) 117 35-40
219. VDLQKE C4M7E0 C4M7E0_ENTHI [tr] Uncharacterized protein 565 483-488
220. PRSVSL C4M299 C4M299_ENTHI [tr] Serine/threonine-protein phosphatase 522 491-496
221. SVSLKD C4M7B3 C4M7B3_ENTHI [tr] Protein kinase putative 1134 764-769
222. VTQNPF A0A175JTJ5 A0A175JTJ5_ENTHI [tr] Uncharacterized protein 496 354-359
223. VTQNPF B1N3Z3 B1N3Z3_ENTHI [tr] Uncharacterized protein 704 562-567
224. VTQNPF C4LY45 C4LY45_ENTHI [tr] Uncharacterized protein 459 335-340
225. DDQCLL C4M956 C4M956_ENTHI [tr] Leucine-rich repeat containing protein 666 240-245
226. ASKARP C4LYP7 C4LYP7_ENTHI [tr] Guanine nucleotide regulatory protein putative 488 343-348
227. ASKARP S0AY13 S0AY13_ENTHI [tr] Guanine nucleotide regulatory protein, putative 488 343-348
228. ASKARP S0AZI0 S0AZI0_ENTHI [tr] Guanine nucleotide regulatory protein, putative 488 343-348
229. TNKPVV C4M524 C4M524_ENTHI [tr] Uncharacterized protein 346 242-247
230. KPVVSA C4LUW9 C4LUW9_ENTHI [tr] Sulfate adenylyltransferase putative 477 251-256
231. KPVVSA S0AW95 S0AW95_ENTHI [tr] Sulfate adenylyltransferase, putative 477 251-256
232. PVVSAL C4LZS7 C4LZS7_ENTHI [tr] High mobility group (HMG) box domain containing protein 287 226-231
233. VSALHG C4M3P7 C4M3P7_ENTHI [tr] WD domain containing protein 775 52-57
234. NGSSNG A0A175JMF2 A0A175JMF2_ENTHI [tr] Lysozyme putative 747 356-361
235. NGSSNG C4M0W3 C4M0W3_ENTHI [tr] Lysozyme, putative 722 331-336
236. EKLSSI C4M122 C4M122_ENTHI [tr] Uncharacterized protein 586 371-376
237. EKLSSI C4M339 C4M339_ENTHI [tr] Uncharacterized protein 905 482-487
238. LSSIES A0A175JHG5 A0A175JHG5_ENTHI [tr] Pumilio family RNA-binding protein 471 78-83
239. LSSIES A0A175JWV3 A0A175JWV3_ENTHI [tr] Pumilio family RNA-binding protein 227 42-47
240. LSSIES B1N489 B1N489_ENTHI [tr] Pumilio family RNA-binding protein 262 42-47
241. LSSIES C4LWP2 C4LWP2_ENTHI [tr] Pumilio family RNA-binding protein 435 42-47

**NMDA 2C**

1. PALLLT C4M144 C4M144_ENTHI [tr] Uncharacterized protein 1041 923-928
2. LLLTSL B1N2R1 B1N2R1_ENTHI [tr] Uncharacterized protein 529 8-13
3. LLLTSL C4M703 C4M703_ENTHI [tr] Uncharacterized protein 366 4-9
4. LTSLFG C4M1J9 C4M1J9_ENTHI [tr] 4-alpha-glucanotransferase putative 825 549-554
5. EQGMTV C4M5T0 C4M5T0_ENTHI [tr] Rho family GTPase 194 135-140
6. EQGMTV Q24816 RACC_ENTHI [sp] Rho-related protein racC precursor 194 135-140
7. LDLPLE C4M198 C4M198_ENTHI [tr] Uncharacterized protein 473 463-468
8. VNTTNP C4M467 C4M467_ENTHI [tr] Uncharacterized protein 774 202-207
9. NTTNPS C4M5I0 C4M5I0_ENTHI [tr] Rho guanine nucleotide exchange factor putative 696 624-629
10. TTNPSS C4M5I0 C4M5I0_ENTHI [tr] Rho guanine nucleotide exchange factor putative 696 625-630
11. TNPSSL C4M027 C4M027_ENTHI [tr] Uncharacterized protein 579 104-109
12. TNPSSL C4M6Z8 C4M6Z8_ENTHI [tr] Poly dp-ribose glycohydrolase putative 450 438-443
13. NPSSLL C4M6Z8 C4M6Z8_ENTHI [tr] Poly dp-ribose glycohydrolase putative 450 439-444
14. PSSLLT C4M6Z8 C4M6Z8_ENTHI [tr] Poly dp-ribose glycohydrolase putative 450 440-445
15. SSLLTQ A0A175JUB5 A0A175JUB5_ENTHI [tr] AP complex subunit beta 855 318-323
16. SSLLTQ A0A175K0T5 A0A175K0T5_ENTHI [tr] Uncharacterized protein 807 524-529
17. SSLLTQ B1N424 B1N424_ENTHI [tr] AP complex subunit beta 855 318-323
18. SSLLTQ C4M0Y2 C4M0Y2_ENTHI [tr] Uncharacterized protein 323 306-311
19. SSLLTQ C4MB88 C4MB88_ENTHI [tr] Uncharacterized protein 769 524-529
20. AHVHGI A0A175JUD4 A0A175JUD4_ENTHI [tr] Ubiquitin carboxyl-terminal hydrolase domain containing protein 1163 1117-1122
21. AHVHGI C4M788 C4M788_ENTHI [tr] Ubiquitin carboxyl-terminal hydrolase family protein 1063 972-977
22. DTEAVA C4LYN0 C4LYN0_ENTHI [tr] Phospholipase b putative 552 36-41
23. TEAVAQ C4LV76 C4LV76_ENTHI [tr] Gal galnac lectin igl2 putative 494 203-208
24. TEAVAQ S0AVR6 S0AVR6_ENTHI [tr] Gal/GalNAc lectin Igl2, putative 494 203-208
25. DFISSQ C4M4U2 C4M4U2_ENTHI [tr] Uncharacterized protein 1102 852-857
26. FISSQT C4LSK8 C4LSK8_ENTHI [tr] Protein kinase putative 1893 671-676
27. VPILSI C4LYT2 C4LYT2_ENTHI [tr] Uncharacterized protein 133 27-32
28. VPILSI C4MAI2 C4MAI2_ENTHI [tr] IBR domain containing protein 613 189-194
29. PILSIS C4M1B6 C4M1B6_ENTHI [tr] Uncharacterized protein 1221 1049-1054
30. PILSIS C4MAI2 C4MAI2_ENTHI [tr] IBR domain containing protein 613 190-195
31. SISGGS C4M9D1 C4M9D1_ENTHI [tr] Uncharacterized protein 691 22-27
32. LEQQLQ C4LZH7 C4LZH7_ENTHI [tr] Uncharacterized protein 250 67-72
33. QQLQVL C4M990 C4M990_ENTHI [tr] Uncharacterized protein 755 39-44
34. LQVLFK C4M978 C4M978_ENTHI [tr] ABC transporter putative 1372 1180-1185
35. HALFLE C4LUY1 C4LUY1_ENTHI [tr] Myb family DNA-binding protein shaqkyf family 189 58-63
36. ALFLEG C4LUY1 C4LUY1_ENTHI [tr] Myb family DNA-binding protein shaqkyf family 189 59-64
37. RLLRQL C4LXY7 C4LXY7_ENTHI [tr] Protein kinase putative 1038 558-563
38. LLRQLD A0A175JSH0 A0A175JSH0_ENTHI [tr] Uncharacterized protein 922 273-278
39. LLRQLD C4M4L0 C4M4L0_ENTHI [tr] Uncharacterized protein 908 273-278
40. SREEAE B1N3W3 B1N3W3_ENTHI [tr] Calcium-dependent protein kinase 2, putative 413 359-364
41. SREEAE C4LZF8 C4LZF8_ENTHI [tr] Proteasome subunit beta type 225 171-176
42. SREEAE C4M541 C4M541_ENTHI [tr] HEAT repeat domain containing protein 2075 840-845
43. SREEAE Q5NSY0 Q5NSY0_ENTHI [tr] Rab family GTPase (Fragment) 238 136-141
44. SVVTES C4LX25 C4LX25_ENTHI [tr] Uncharacterized protein 1406 738-743
45. RLSLRQ C4LZN8 C4LZN8_ENTHI [tr] Uncharacterized protein 202 125-130
46. LSLRQK C4LZN8 C4LZN8_ENTHI [tr] Uncharacterized protein 202 126-131
47. KVRDGV C4LSG8 C4LSG8_ENTHI [tr] Uncharacterized protein 2537 717-722
48. VAILAL A0A175K1Y0 A0A175K1Y0_ENTHI [tr] Clathrin heavy chain putative 404 164-169
49. VAILAL A0A175K294 A0A175K294_ENTHI [tr] Clathrin heavy chain putative 1144 68-73
50. VAILAL Q1EQ28 Q1EQ28_ENTHI [tr] Clathrin heavy chain (Fragment) 1622 122-127
51. YSASLQ C4M507 C4M507_ENTHI [tr] Transporter major facilitator family 520 372-377
52. FVIVES C4M107 C4M107_ENTHI [tr] Vacular protein sorting 33A, putative 995 451-456
53. FVIVES Q1EPY0 Q1EPY0_ENTHI [tr] EhVps33 661 117-122
54. TFSSGD A0A175JIP0 A0A175JIP0_ENTHI [tr] Uncharacterized protein 3302 2494-2499
55. TFSSGD C4LX72 C4LX72_ENTHI [tr] Uncharacterized protein 3289 2481-2486
56. FCIDIL C4M2M3 C4M2M3_ENTHI [tr] Uncharacterized protein 547 409-414
57. IDILKK C4LYW2 C4LYW2_ENTHI [tr] Uncharacterized protein 726 497-502
58. IDILKK C4M7N5 C4M7N5_ENTHI [tr] Uncharacterized protein 471 139-144
59. DILKKL C4LXK6 C4LXK6_ENTHI [tr] Dopey domain protein putative 1660 993-998
60. LYLVTN C4LXW3 C4LXW3_ENTHI [tr] Cyclin domain containing protein 398 154-159
61. LYLVTN S0AVK0 S0AVK0_ENTHI [tr] Cyclin, domain containing protein 366 122-127
62. YLVTNG C4M1L0 C4M1L0_ENTHI [tr] Uncharacterized protein 273 72-77
63. LVTNGK C4M1L0 C4M1L0_ENTHI [tr] Uncharacterized protein 273 73-78
64. HGKRVR Q58P26 Q58P26_ENTHI [tr] Ornithine decarboxylase 413 274-279
65. AIGSLT C4M555 C4M555_ENTHI [tr] Leucine rich repeat / protein phosphatase 2C domain containing protein 819 417-422
66. LTINEE C4LTX8 C4LTX8_ENTHI [tr] Leucine rich repeat protein bspa family 996 716-721
67. LTINEE C4M670 C4M670_ENTHI [tr] Protein kinase domain containing protein 482 364-369
68. GTVSPS C4M471 C4M471_ENTHI [tr] Kinesin-like protein 629 315-320
69. PSAFLE C4LZI6 C4LZI6_ENTHI [tr] Tyrosine kinase putative 2577 170-175
70. FLEPYS C4M0N8 C4M0N8_ENTHI [tr] Uncharacterized protein 326 67-72
71. CLTVVA C4M4K7 C4M4K7_ENTHI [tr] Signal peptidase putative 340 150-155
72. CLTVVA S0B255 S0B255_ENTHI [tr] Signal peptidase, putative 340 150-155
73. LTVVAI C4M4K7 C4M4K7_ENTHI [tr] Signal peptidase putative 340 151-156
74. LTVVAI S0B255 S0B255_ENTHI [tr] Signal peptidase, putative 340 151-156
75. AFTIGK C4M401 C4M401_ENTHI [tr] DNA damage-binding protein putative 1088 527-532
76. VPIENP C4LUV4 C4LUV4_ENTHI [tr] Ras family protein 522 296-301
77. FFAVIF A0A175JI07 A0A175JI07_ENTHI [tr] Uncharacterized protein 273 202-207
78. FLASYT C4M2H2 C4M2H2_ENTHI [tr] Uncharacterized protein 479 200-205
79. EQYIDT C4M640 C4M640_ENTHI [tr] Protein kinase domain containing protein 562 221-226
80. TVPNGS C4MAN9 C4MAN9_ENTHI [tr] Protein kinase putative 779 313-318
81. GKDEGC C4M8Y3 C4M8Y3_ENTHI [tr] Glucosamine 6-phosphate N-acetyltransferase. putative 159 121-126
82. LVTIGS A0A175JT87 A0A175JT87_ENTHI [tr] Rhogap domain containing protein 605 398-403
83. LVTIGS C4M676 C4M676_ENTHI [tr] RhoGAP domain containing protein 509 302-307
84. TIGSGK C4LYL2 C4LYL2_ENTHI [tr] HEAT repeat domain containing protein 1589 1165-1170
85. TTGYGI A0A060N124 A0A060N124_ENTHI [tr] Serine/threonine-protein phosphatase 433 33-38
86. TTGYGI C4M672 C4M672_ENTHI [tr] Serine/threonine-protein phosphatase 433 33-38
87. TGYGIA A0A060N124 A0A060N124_ENTHI [tr] Serine/threonine-protein phosphatase 433 34-39
88. TGYGIA C4M672 C4M672_ENTHI [tr] Serine/threonine-protein phosphatase 433 34-39
89. ALLQFL C4LWP9 C4LWP9_ENTHI [tr] Uncharacterized protein 640 82-87
90. ETQKLE A0A175JZ89 A0A175JZ89_ENTHI [tr] Uncharacterized protein 554 158-163
91. ETQKLE C4M596 C4M596_ENTHI [tr] Uncharacterized protein 946 603-608
92. ETQKLE C4MA82 C4MA82_ENTHI [tr] Uncharacterized protein 531 135-140
93. QNEKNE C4LYI4 C4LYI4_ENTHI [tr] Guanylate-binding protein putative 752 579-584
94. NEKNEV C4M7A0 C4M7A0_ENTHI [tr] Uncharacterized protein 888 8-13
95. SSKLDI C4LXG1 C4LXG1_ENTHI [tr] Purine nucleoside phosphorylase putative 235 99-104
96. SSKLDI S0AZI4 S0AZI4_ENTHI [tr] Purine nucleoside phosphorylase, putative 235 99-104
97. LLVFAW C4LYS5 C4LYS5_ENTHI [tr] Uncharacterized protein 434 198-203
98. VPNSSQ C4M401 C4M401_ENTHI [tr] DNA damage-binding protein putative 1088 240-245
99. PNSSQL C4LTH7 C4LTH7_ENTHI [tr] Uncharacterized protein 272 64-69
100. RDMVTT B1N3I6 B1N3I6_ENTHI [tr] Uncharacterized protein 275 107-112
101. RDMVTT C4LV73 C4LV73_ENTHI [tr] Uncharacterized protein 292 107-112
102. AGVSSS B1N4Q0 B1N4Q0_ENTHI [tr] Uncharacterized protein 529 235-240
103. AGVSSS B1N4R7 B1N4R7_ENTHI [tr] Uncharacterized protein 562 235-240
104. AGVSSS C4MA93 C4MA93_ENTHI [tr] Uncharacterized protein 576 234-239
105. AGVSSS C4MAA3 C4MAA3_ENTHI [tr] Uncharacterized protein 577 235-240
106. AGVSSS C4MAP9 C4MAP9_ENTHI [tr] Uncharacterized protein 577 235-240
107. VSSSLD Q24847 Q24847_ENTHI [tr] Surface antigen protein 294 122-127
108. ATRTIE C4M2S0 C4M2S0_ENTHI [tr] Rab family GTPase 212 46-51
109. ATRTIE Q9BLE3 Q9BLE3_ENTHI [tr] Small GTPase Rab11C 212 46-51
110. DVSRVS C4LZK3 C4LZK3_ENTHI [tr] Uncharacterized protein 116 17-22
111. DVSRVS C4M7R8 C4M7R8_ENTHI [tr] Uncharacterized protein 207 160-165
112. RADRSG A0A175JFM0 A0A175JFM0_ENTHI [tr] Uncharacterized protein 2556 2312-2317
113. RADRSG C4LUR0 C4LUR0_ENTHI [tr] Uncharacterized protein 2533 2289-2294
114. LEDLPL C4M870 C4M870_ENTHI [tr] Ribosomal RNA methyltransferase putative 231 208-213
115. EDLPLL A0A175JX29 A0A175JX29_ENTHI [tr] Thioredoxin putative 624 149-154
116. RREALL A0A175JPW1 A0A175JPW1_ENTHI [tr] Uncharacterized protein 2546 2100-2105
117. RREALL B1N3J3 B1N3J3_ENTHI [tr] Uncharacterized protein 2532 2086-2091
118. REALLH A0A060N0B4 A0A060N0B4_ENTHI [tr] Sulfate adenylyltransferase, putative 423 281-286
119. REALLH C4LVD3 C4LVD3_ENTHI [tr] Sulfate adenylyltransferase putative 423 281-286
120. REALLH O76156 SAT_ENTHI [sp] Sulfate adenylyltransferase 423 281-286
121. REALLH S0B1J5 S0B1J5_ENTHI [tr] Sulfate adenylyltransferase, putative 423 281-286
122. EALLHA A0A060N0B4 A0A060N0B4_ENTHI [tr] Sulfate adenylyltransferase, putative 423 282-287
123. EALLHA C4LVD3 C4LVD3_ENTHI [tr] Sulfate adenylyltransferase putative 423 282-287
124. EALLHA O76156 SAT_ENTHI [sp] Sulfate adenylyltransferase 423 282-287
125. EALLHA S0B1J5 S0B1J5_ENTHI [tr] Sulfate adenylyltransferase, putative 423 282-287
126. SLPSSV C4M1F8 C4M1F8_ENTHI [tr] Leucine rich repeat protein bspa family 334 187-192
127. RLAQAQ B1N624 B1N624_ENTHI [tr] Aminoglycoside 3'-phosphotransferase, putative 253 130-135
128. LAQAQS B1N624 B1N624_ENTHI [tr] Aminoglycoside 3'-phosphotransferase, putative 253 131-136
129. LPIYRE C4LSG8 C4LSG8_ENTHI [tr] Uncharacterized protein 2537 469-474
130. GHRGRT B1N461 B1N461_ENTHI [tr] Protein phosphatase domain-containing protein 236 27-32
131. GHRGRT C4LV13 C4LV13_ENTHI [tr] Protein phosphatase putative 263 54-59
132. GRTLGL C4M9X5 C4M9X5_ENTHI [tr] Ribonuclease P protein subunit p30, putative 236 199-204
133. RTLGLG C4MA27 C4MA27_ENTHI [tr] Helicase putative 1214 331-336
134. SGGLDE A0A060N1K6 A0A060N1K6_ENTHI [tr] T-complex protein 1, alpha subunit, putative 544 291-296
135. SGGLDE C4LW75 C4LW75_ENTHI [tr] T-complex protein 1 alpha subunit putative 544 291-296
136. GLDEIS B1N3A9 B1N3A9_ENTHI [tr] Uncharacterized protein 294 236-241
137. LDEISR C4LXP9 C4LXP9_ENTHI [tr] Protein kinase putative 434 179-184
138. LDEISR C4LZ17 C4LZ17_ENTHI [tr] Uncharacterized protein 493 286-291
139. RRISSL C4LY13 C4LY13_ENTHI [tr] Uncharacterized protein 473 254-259
140. ISSLES A0A175JPH6 A0A175JPH6_ENTHI [tr] Uncharacterized protein 557 421-426
141. ISSLES C4M266 C4M266_ENTHI [tr] Uncharacterized protein 886 750-755

**NMDA 2D**

1. KMLLLL C4M9I8 C4M9I8_ENTHI [tr] WD domain containing protein 323 213-218
2. LLLLAL A0A060N4K3 A0A060N4K3_ENTHI [tr] Serine/threonine-protein phosphatase 304 92-97
3. LLLLAL C4LUY7 C4LUY7_ENTHI [tr] Uncharacterized protein 1311 836-841
4. LLLLAL C4M4D1 C4M4D1_ENTHI [tr] Ribonuclease putative 251 6-11
5. LLLLAL C4M907 C4M907_ENTHI [tr] Serine/threonine-protein phosphatase 304 92-97
6. LLLLAL C4MAH9 C4MAH9_ENTHI [tr] Uncharacterized protein 283 4-9
7. LLLLAL S0AZ89 S0AZ89_ENTHI [tr] Uncharacterized protein 236 4-9
8. LLLALA A0A060N1L0 A0A060N1L0_ENTHI [tr] Lysozyme, putative 212 6-11
9. LLLALA C4LWG9 C4LWG9_ENTHI [tr] Lysozyme putative 212 6-11
10. PFPEEA C4MAR1 C4MAR1_ENTHI [tr] Tyrosine kinase, putative 1883 1833-1838
11. GGPGGG C4M3E2 C4M3E2_ENTHI [tr] Uncharacterized protein 240 27-32
12. GGPGGG C4M740 C4M740_ENTHI [tr] Uncharacterized protein 239 26-31
13. LNVALV C4M659 C4M659_ENTHI [tr] Uncharacterized protein 271 212-217
14. AVAAAV C4LSK5 C4LSK5_ENTHI [tr] HEAT repeat domain containing protein 1074 102-107
15. RSLVLQ C4LTP4 C4LTP4_ENTHI [tr] Uncharacterized protein 2909 2537-2542
16. LCDLLS C4LSL6 C4LSL6_ENTHI [tr] Uncharacterized protein 202 110-115
17. DLLSGL C4LZX4 C4LZX4_ENTHI [tr] Protein with RhoGEF and ArfGAP domains 997 26-31
18. VFEDDS C4M3G6 C4M3G6_ENTHI [tr] Uncharacterized protein 2566 2382-2387
19. ILDFLS C4M6R2 C4M6R2_ENTHI [tr] Uncharacterized protein 672 114-119
20. QTSLPI C4M4H5 C4M4H5_ENTHI [tr] Uncharacterized protein 1719 286-291
21. SLPIVA C4LSV9 C4LSV9_ENTHI [tr] Metal cation transporter zinc zn2-iron fe2 permease zip family 289 197-202
22. SLPIVA S0B0W4 S0B0W4_ENTHI [tr] Metal cation transporter, zinc (Zn2 )-iron (Fe2 ) permease (ZIP) family 289 197-202
23. SLPIVA S0B1U0 S0B1U0_ENTHI [tr] Metal cation transporter, zinc (Zn2 )-iron (Fe2 ) permease (ZIP) family 289 197-202
24. LPIVAV C4LSV9 C4LSV9_ENTHI [tr] Metal cation transporter zinc zn2-iron fe2 permease zip family 289 198-203
25. LPIVAV S0B0W4 S0B0W4_ENTHI [tr] Metal cation transporter, zinc (Zn2 )-iron (Fe2 ) permease (ZIP) family 289 198-203
26. LPIVAV S0B1U0 S0B1U0_ENTHI [tr] Metal cation transporter, zinc (Zn2 )-iron (Fe2 ) permease (ZIP) family 289 198-203
27. AALVLT C4M6M7 C4M6M7_ENTHI [tr] Citrate transporter putative 784 608-613
28. ALVLTP C4M1W2 C4M1W2_ENTHI [tr] DEAD/DEAH box helicase, putative 450 101-106
29. TFLQLG C4M3X0 C4M3X0_ENTHI [tr] Calcium-transporting ATPase 1072 43-48
30. LQLGSS C4M3L2 C4M3L2_ENTHI [tr] mRNA capping enzyme putative 595 114-119
31. LQLGSS C4M3Q8 C4M3Q8_ENTHI [tr] Uncharacterized protein 209 182-187
32. TEQQLQ A0A060N5A0 A0A060N5A0_ENTHI [tr] Uncharacterized protein 356 144-149
33. TEQQLQ C4M154 C4M154_ENTHI [tr] Uncharacterized protein 347 135-140
34. EVLEEY C4M1Z4 C4M1Z4_ENTHI [tr] Zinc finger protein putative 478 14-19
35. TSFVAV C4LVP0 C4LVP0_ENTHI [tr] Acetyltransferase gnat family 183 41-46
36. SFVAVT C4LVP0 C4LVP0_ENTHI [tr] Acetyltransferase gnat family 183 42-47
37. YIEVLT C4LVM4 C4LVM4_ENTHI [tr] Sec7 domain containing protein 1163 952-957
38. VLTDGS C4M383 C4M383_ENTHI [tr] Uncharacterized protein 433 414-419
39. RGALTL C4M1Y8 C4M1Y8_ENTHI [tr] RhoGAP domain containing protein 569 278-283
40. ALTLDP C4LWL0 C4LWL0_ENTHI [tr] Uncharacterized protein 1327 1051-1056
41. QIRLLF C4M990 C4M990_ENTHI [tr] Uncharacterized protein 755 211-216
42. AREEAE C4M041 C4M041_ENTHI [tr] Villidin putative 1059 131-136, 139-144
43. AGGGGS A0A175JMK3 A0A175JMK3_ENTHI [tr] Sec1 family protein 349 24-29
44. AGGGGS C4M107 C4M107_ENTHI [tr] Vacular protein sorting 33A, putative 995 24-29
45. EPPLLP C4M5V1 C4M5V1_ENTHI [tr] Rho guanine nucleotide exchange factor putative 492 110-115
46. PLLPGG C4M616 C4M616_ENTHI [tr] Lipase putative 433 356-361
47. PAGLFA C4M295 C4M295_ENTHI [tr] Adenylyl cyclase-associated protein 478 253-258
48. AVVARG P37213 PPDK_ENTHI [sp] Pyruvate, phosphate dikinase 885 456-461
49. AVVARG Q24801 Q24801_ENTHI [tr] Pyruvate phosphate dikinase 885 456-461
50. FNEDGF C4LUB3 C4LUB3_ENTHI [tr] Sand family protein 406 249-254
51. FNEDGF C4M8M7 C4M8M7_ENTHI [tr] Phospholipase b putative 550 545-550
52. RTWEVV A0A175JYI8 A0A175JYI8_ENTHI [tr] Ras guanine nucleotide exchange factor putative 763 633-638
53. RTWEVV C4MAJ3 C4MAJ3_ENTHI [tr] Ras guanine nucleotide exchange factor, putative 763 633-638
54. EQQTLR A0A175K0D6 A0A175K0D6_ENTHI [tr] Uncharacterized protein 396 348-353
55. EQQTLR B1N5H3 B1N5H3_ENTHI [tr] Uncharacterized protein (Fragment) 388 348-353
56. LRLKYP B1N4X1 B1N4X1_ENTHI [tr] Uncharacterized protein 194 2-7
57. LRLKYP C4LZP7 C4LZP7_ENTHI [tr] Uncharacterized protein 450 401-406
58. LRLKYP C4M6T3 C4M6T3_ENTHI [tr] Lipase putative 432 240-245
59. GTCIRD C4M746 C4M746_ENTHI [tr] UDP-glucose 4-epimerase putative 341 232-237
60. GTCIRD S0AYJ6 S0AYJ6_ENTHI [tr] UDP-glucose 4-epimerase, putative 341 232-237
61. EKRCCK B1N5G5 B1N5G5_ENTHI [tr] Uncharacterized protein 866 331-336
62. FCIDIL C4M2M3 C4M2M3_ENTHI [tr] Uncharacterized protein 547 409-414
63. IDILKR A0A175JVD2 A0A175JVD2_ENTHI [tr] Uncharacterized protein 224 159-164
64. IDILKR C4M778 C4M778_ENTHI [tr] Uncharacterized protein 210 145-150
65. GFSYDL C4M9T6 C4M9T6_ENTHI [tr] Uncharacterized protein 1407 416-421
66. LYLVTN C4LXW3 C4LXW3_ENTHI [tr] Cyclin domain containing protein 398 154-159
67. LYLVTN S0AVK0 S0AVK0_ENTHI [tr] Cyclin, domain containing protein 366 122-127
68. YLVTNG C4M1L0 C4M1L0_ENTHI [tr] Uncharacterized protein 273 72-77
69. LVTNGK C4M1L0 C4M1L0_ENTHI [tr] Uncharacterized protein 273 73-78
70. GKKIDG C4M1Z4 C4M1Z4_ENTHI [tr] Zinc finger protein putative 478 368-373
71. KKIDGV C4M3F0 C4M3F0_ENTHI [tr] Uncharacterized protein 335 265-270
72. AIGSLT C4M555 C4M555_ENTHI [tr] Leucine rich repeat / protein phosphatase 2C domain containing protein 819 417-422
73. LTINEE C4LTX8 C4LTX8_ENTHI [tr] Leucine rich repeat protein bspa family 996 716-721
74. LTINEE C4M670 C4M670_ENTHI [tr] Protein kinase domain containing protein 482 364-369
75. GTVSPS C4M471 C4M471_ENTHI [tr] Kinesin-like protein 629 315-320
76. PSAFLE C4LZI6 C4LZI6_ENTHI [tr] Tyrosine kinase putative 2577 170-175
77. FLEPYS C4M0N8 C4M0N8_ENTHI [tr] Uncharacterized protein 326 67-72
78. CLTVVA C4M4K7 C4M4K7_ENTHI [tr] Signal peptidase putative 340 150-155
79. CLTVVA S0B255 S0B255_ENTHI [tr] Signal peptidase, putative 340 150-155
80. LTVVAV C4LU91 C4LU91_ENTHI [tr] Transporter major facilitator family 530 462-467
81. FFAVIF A0A175JI07 A0A175JI07_ENTHI [tr] Uncharacterized protein 273 202-207
82. FLASYT C4M2H2 C4M2H2_ENTHI [tr] Uncharacterized protein 479 200-205
83. EEYVDT A0A175JFL2 A0A175JFL2_ENTHI [tr] Uncharacterized protein 2784 2360-2365
84. EEYVDT C4LUZ9 C4LUZ9_ENTHI [tr] Uncharacterized protein 2772 2348-2353
85. TVPNGS C4MAN9 C4MAN9_ENTHI [tr] Protein kinase putative 779 313-318
86. STEKNI C4MAM3 C4MAM3_ENTHI [tr] Uncharacterized protein 208 97-102
87. PRVEEA C4LXZ6 C4LXZ6_ENTHI [tr] Queuine tRNA-ribosyltransferase catalytic subunit 1 397 156-161
88. VEEALT A0A175JE57 A0A175JE57_ENTHI [tr] Uncharacterized protein 520 446-451
89. VEEALT B1N2H0 B1N2H0_ENTHI [tr] Uncharacterized protein 294 220-225
90. LTQLKA B1N4L2 B1N4L2_ENTHI [tr] Uncharacterized protein 430 115-120
91. LKAGKL C4LX95 C4LX95_ENTHI [tr] Uncharacterized protein 445 211-216
92. KAGKLD C4LX95 C4LX95_ENTHI [tr] Uncharacterized protein 445 212-217
93. LVTIGS A0A175JT87 A0A175JT87_ENTHI [tr] Rhogap domain containing protein 605 398-403
94. LVTIGS C4M676 C4M676_ENTHI [tr] RhoGAP domain containing protein 509 302-307
95. TIGSGK C4LYL2 C4LYL2_ENTHI [tr] HEAT repeat domain containing protein 1589 1165-1170
96. TTGYGI A0A060N124 A0A060N124_ENTHI [tr] Serine/threonine-protein phosphatase 433 33-38
97. TTGYGI C4M672 C4M672_ENTHI [tr] Serine/threonine-protein phosphatase 433 33-38
98. TGYGIA A0A060N124 A0A060N124_ENTHI [tr] Serine/threonine-protein phosphatase 433 34-39
99. TGYGIA C4M672 C4M672_ENTHI [tr] Serine/threonine-protein phosphatase 433 34-39
100. GIALHK A0A175JXP3 A0A175JXP3_ENTHI [tr] 1 4-alpha-glucan branching enzyme putative 595 413-418
101. GIALHK B1N4C6 B1N4C6_ENTHI [tr] 1,4-alpha-glucan branching enzyme, putative 574 392-397
102. GIALHK C4M384 C4M384_ENTHI [tr] 1 4-alpha-glucan branching enzyme putative 680 498-503
103. KRPIDL A0A175JGH4 A0A175JGH4_ENTHI [tr] Ubiquitin-protein ligase putative 663 338-343
104. KRPIDL C4LVT5 C4LVT5_ENTHI [tr] Ubiquitin-protein ligase, putative 652 327-332
105. ALLQFL C4LWP9 C4LWP9_ENTHI [tr] Uncharacterized protein 640 82-87
106. DDEIEM A0A175JHS6 A0A175JHS6_ENTHI [tr] Uncharacterized protein 318 21-26
107. DDEIEM C4LZA6 C4LZA6_ENTHI [tr] Myb-like DNA-binding domain containing protein 238 140-145
108. DEIEML C4LZA6 C4LZA6_ENTHI [tr] Myb-like DNA-binding domain containing protein 238 141-146
109. EIEMLE C4M0B3 C4M0B3_ENTHI [tr] Uncharacterized protein 490 230-235
110. EIEMLE C4M4V5 C4M4V5_ENTHI [tr] RecQ family helicase, putative 1182 282-287
111. IEMLER C4M6N9 C4M6N9_ENTHI [tr] Protein kinase putative 739 314-319
112. LERLWL C4M7W1 C4M7W1_ENTHI [tr] Uncharacterized protein 1656 243-248
113. HNDKIE C4M2I5 C4M2I5_ENTHI [tr] Apyrase putative 362 316-321
114. NDKIEV C4MAK4 C4MAK4_ENTHI [tr] Uncharacterized protein 509 302-307
115. IEVMSS A0A175JT28 A0A175JT28_ENTHI [tr] Sec7 domain containing protein 1416 312-317
116. IEVMSS C4M5V4 C4M5V4_ENTHI [tr] Sec7 domain protein 1396 312-317
117. SSKLDI C4LXG1 C4LXG1_ENTHI [tr] Purine nucleoside phosphorylase putative 235 99-104
118. SSKLDI S0AZI4 S0AZI4_ENTHI [tr] Purine nucleoside phosphorylase, putative 235 99-104
119. GLSLLV A0A175JPW1 A0A175JPW1_ENTHI [tr] Uncharacterized protein 2546 986-991
120. GLSLLV B1N3J3 B1N3J3_ENTHI [tr] Uncharacterized protein 2532 972-977
121. SLLVFA C4LSU8 C4LSU8_ENTHI [tr] Uncharacterized protein 533 238-243
122. SLLVFA C4LYS5 C4LYS5_ENTHI [tr] Uncharacterized protein 434 197-202
123. LLVFAW C4LYS5 C4LYS5_ENTHI [tr] Uncharacterized protein 434 198-203
124. RMDFLL C4M298 C4M298_ENTHI [tr] Proteasome regulatory subunit putative 448 165-170
125. PPPAKP B1N3A9 B1N3A9_ENTHI [tr] Uncharacterized protein 294 279-284
126. PPPAKP C4M203 C4M203_ENTHI [tr] Uncharacterized protein 898 722-727
127. AKPPPP C4M622 C4M622_ENTHI [tr] Diaphanous protein putative 1183 1078-1083
128. AKPPPP C4MAI3 C4MAI3_ENTHI [tr] Rap ran GTPase-activating protein putative 667 77-82
129. AKPPPP Q9NGX1 Q9NGX1_ENTHI [tr] Diaphanous protein (Fragment) 1096 992-997
130. AKPPPP Q9NGX2 Q9NGX2_ENTHI [tr] Diaphanous protein 1209 1104-1109
131. PPPPPQ A0A175JE76 A0A175JE76_ENTHI [tr] Serine threonine-protein kinase putative 601 309-314
132. PPPPPQ C4LT14 C4LT14_ENTHI [tr] Serine/threonine-protein kinase, putative 600 308-313
133. PPPPPQ C4LTV1 C4LTV1_ENTHI [tr] WH2 motif domain contaning protein 415 295-300
134. PPPPPQ C4M6C1 C4M6C1_ENTHI [tr] Uncharacterized protein 168 151-156, 162-167
135. PQGLGL C4M2X6 C4M2X6_ENTHI [tr] Actin putative 713 198-203, 210-215, 222-227, 240-245, 248-253, 256-261, 274-279
136. QGLGLG C4M2X6 C4M2X6_ENTHI [tr] Actin putative 713 199-204, 211-216, 223-228, 241-246, 249-254, 257-262, 275-280
137. GLGLGL C4LXI9 C4LXI9_ENTHI [tr] Actin putative 876 365-370, 394-399, 417-422
138. LGLGLG C4LXI9 C4LXI9_ENTHI [tr] Actin putative 876 366-371, 395-400, 418-423
139. EPAEPP C4M946 C4M946_ENTHI [tr] Uncharacterized protein 1653 1484-1489
140. LCRLAF C4LX64 C4LX64_ENTHI [tr] Uncharacterized protein 205 28-33
141. PAAPPP C4LVS4 C4LVS4_ENTHI [tr] Rap ran GTPase-activating protein putative 610 35-40
142. PPPCPY A0A175JFN0 A0A175JFN0_ENTHI [tr] Uncharacterized protein 512 85-90
143. PPPCPY C4LV01 C4LV01_ENTHI [tr] Uncharacterized protein 488 85-90
144. PYLDLE C4LYN0 C4LYN0_ENTHI [tr] Phospholipase b putative 552 519-524
145. PSDSED C4MAK5 C4MAK5_ENTHI [tr] Uncharacterized protein 232 57-62
146. SLGGAS C4M978 C4M978_ENTHI [tr] ABC transporter putative 1372 1038-1043
147. GASLGG C4M1H8 C4M1H8_ENTHI [tr] Sericin 1 putative 718 548-553
148. GASLGG O15607 O15607_ENTHI [tr] Sericin homologue (Fragment) 157 89-94
149. ASLELL A0A060N0Z0 A0A060N0Z0_ENTHI [tr] Phosphoglycerate kinase 415 395-400
150. ASLELL A0A060N142 A0A060N142_ENTHI [tr] Phosphoglycerate kinase 415 395-400
151. ASLELL A0A060N290 A0A060N290_ENTHI [tr] Phosphoglycerate kinase 415 395-400
152. ASLELL A0A060N4F3 A0A060N4F3_ENTHI [tr] Phosphoglycerate kinase 415 395-400
153. ASLELL A0A060N4R1 A0A060N4R1_ENTHI [tr] Phosphoglycerate kinase 415 395-400
154. ASLELL A0A060N5R3 A0A060N5R3_ENTHI [tr] Phosphoglycerate kinase 415 395-400
155. ASLELL A0A060N6J4 A0A060N6J4_ENTHI [tr] Phosphoglycerate kinase 415 395-400
156. ASLELL C4M192 C4M192_ENTHI [tr] Phosphoglycerate kinase 415 395-400
157. ASLELL S0AV80 S0AV80_ENTHI [tr] Phosphoglycerate kinase 415 395-400
158. ASLELL S0AVL3 S0AVL3_ENTHI [tr] Phosphoglycerate kinase 415 395-400
159. ASLELL S0AWR1 S0AWR1_ENTHI [tr] Phosphoglycerate kinase 415 395-400
160. ASLELL S0AWU3 S0AWU3_ENTHI [tr] Phosphoglycerate kinase 415 395-400
161. ASLELL S0AWV1 S0AWV1_ENTHI [tr] Phosphoglycerate kinase 415 395-400
162. ASLELL S0AWV2 S0AWV2_ENTHI [tr] Phosphoglycerate kinase 415 395-400
163. ASLELL S0AX04 S0AX04_ENTHI [tr] Phosphoglycerate kinase 415 395-400
164. ASLELL S0AX27 S0AX27_ENTHI [tr] Phosphoglycerate kinase 415 395-400
165. ASLELL S0AXA5 S0AXA5_ENTHI [tr] Phosphoglycerate kinase 415 395-400
166. ASLELL S0AZ74 S0AZ74_ENTHI [tr] Phosphoglycerate kinase 415 395-400
167. ASLELL S0AZU7 S0AZU7_ENTHI [tr] Phosphoglycerate kinase 415 395-400
168. ASLELL S0B0M3 S0B0M3_ENTHI [tr] Phosphoglycerate kinase 415 395-400
169. APPPPP A0A175JLR6 A0A175JLR6_ENTHI [tr] Uncharacterized protein 492 371-376, 386-391
170. APPPPP B1N5W5 B1N5W5_ENTHI [tr] Diaphanous protein, putative (Fragment) 448 92-97
171. APPPPP C4LVH1 C4LVH1_ENTHI [tr] Diaphanous protein, homolog 2, putative 986 540-545
172. APPPPP C4LYT8 C4LYT8_ENTHI [tr] Uncharacterized protein 338 186-191
173. APPPPP C4LZH0 C4LZH0_ENTHI [tr] Uncharacterized protein 612 365-370
174. APPPPP C4M0H3 C4M0H3_ENTHI [tr] Uncharacterized protein 468 347-352, 362-367
175. APPPPP C4M1G6 C4M1G6_ENTHI [tr] Diaphanous protein, homolog 1, putative 1212 612-617
176. APPPPP C4M2L6 C4M2L6_ENTHI [tr] Uncharacterized protein 271 186-191
177. APPPPP C4M2U9 C4M2U9_ENTHI [tr] Uncharacterized protein 540 355-360, 397-402, 426-431, 435-440
178. APPPPP C4M345 C4M345_ENTHI [tr] Uncharacterized protein 867 21-26
179. APPPPP C4M622 C4M622_ENTHI [tr] Diaphanous protein putative 1183 586-591
180. APPPPP C4M6C1 C4M6C1_ENTHI [tr] Uncharacterized protein 168 161-166
181. APPPPP C4MBT4 C4MBT4_ENTHI [tr] WH2 motif domain contaning protein 181 70-75, 84-89
182. APPPPP Q9NGX1 Q9NGX1_ENTHI [tr] Diaphanous protein (Fragment) 1096 474-479
183. APPPPP Q9NGX2 Q9NGX2_ENTHI [tr] Diaphanous protein 1209 586-591
184. APPPPP S0AXS3 S0AXS3_ENTHI [tr] Uncharacterized protein 318 186-191
185. PRASHR C4LZ51 C4LZ51_ENTHI [tr] Lipase putative 442 304-309
186. SLEDLS C4M4F7 C4M4F7_ENTHI [tr] Protein kinase domain containing protein 569 194-199
187. LEDLSS C4M4F7 C4M4F7_ENTHI [tr] Protein kinase domain containing protein 569 195-200
188. LEDLSS C4MAE3 C4MAE3_ENTHI [tr] F-box domain containing protein 799 442-447
189. EDLSSC C4MAE3 C4MAE3_ENTHI [tr] F-box domain containing protein 799 443-448
190. GGDLGT C4LWM6 C4LWM6_ENTHI [tr] Phospholipase b putative 520 165-170
191. TRRGSA C4M8Y2 C4M8Y2_ENTHI [tr] 5'-3' exonuclease domain containing protein 628 370-375
192. RRGSAH C4M935 C4M935_ENTHI [tr] Protein kinase, putative 686 488-493

**NMDA 3A**

1. LLSRVC A0A175JHL2 A0A175JHL2_ENTHI [tr] Uncharacterized protein 1229 1155-1160
2. LLSRVC C4LW41 C4LW41_ENTHI [tr] Uncharacterized protein 1229 1155-1160
3. LSRVCL A0A175JHL2 A0A175JHL2_ENTHI [tr] Uncharacterized protein 1229 1156-1161
4. LSRVCL C4LW41 C4LW41_ENTHI [tr] Uncharacterized protein 1229 1156-1161
5. VLAGVP C4M653 C4M653_ENTHI [tr] Histidyl-tRNA synthetase putative 506 204-209
6. LKRIGH C4M4B5 C4M4B5_ENTHI [tr] Uncharacterized protein 169 154-159
7. ALLFAV C4LTU9 C4LTU9_ENTHI [tr] Structural maintenance of chromosomes protein 1197 1113-1118
8. LNRVEG C4LXH4 C4LXH4_ENTHI [tr] Uncharacterized protein 540 376-381
9. RVEGLL A0A175JFI6 A0A175JFI6_ENTHI [tr] Protein phosphatase domain-containing protein 947 834-839
10. RVEGLL C4LUM6 C4LUM6_ENTHI [tr] Protein phosphatase domain-containing protein 786 673-678
11. RVEGLL C4M3X4 C4M3X4_ENTHI [tr] Protein phosphatase domain-containing protein 943 833-838
12. LPYNLS C4LSH6 C4LSH6_ENTHI [tr] Leucine rich repeat protein bspa family 1105 206-211
13. LSLEVV C4MB03 C4MB03_ENTHI [tr] Protein kinase putative 390 215-220
14. LGDLPL C4MA79 C4MA79_ENTHI [tr] RNA recognition motif domain containing protein 333 88-93
15. DLPLLP C4M613 C4M613_ENTHI [tr] Uncharacterized protein 640 512-517
16. PLLPFS C4LYN3 C4LYN3_ENTHI [tr] Uncharacterized protein 559 545-550
17. LLPFSS C4LZB5 C4LZB5_ENTHI [tr] Uncharacterized protein 163 146-151
18. FSSPSS A0A175JQ33 A0A175JQ33_ENTHI [tr] Uncharacterized protein 324 165-170
19. FSSPSS C4M396 C4M396_ENTHI [tr] Uncharacterized protein 199 40-45
20. PFSFLQ A0A175JEF4 A0A175JEF4_ENTHI [tr] Uncharacterized protein 321 146-151
21. PFSFLQ C4LTX0 C4LTX0_ENTHI [tr] Uncharacterized protein 244 146-151
22. FSFLQS A0A175JEF4 A0A175JEF4_ENTHI [tr] Uncharacterized protein 321 147-152
23. FSFLQS C4LTX0 C4LTX0_ENTHI [tr] Uncharacterized protein 244 147-152
24. LLAFPQ C4LSG6 C4LSG6_ENTHI [tr] PQ loop repeat protein 228 121-126
25. LDLVSL C4M7I3 C4M7I3_ENTHI [tr] ABC transporter putative 917 610-615
26. LVSLVL C4M8S1 C4M8S1_ENTHI [tr] Uncharacterized protein 710 67-72
27. QLSLEN C4M3H5 C4M3H5_ENTHI [tr] WD repeat protein 341 318-323
28. ENSLSS C4LW65 C4LW65_ENTHI [tr] Uncharacterized protein 209 102-107
29. ENSLSS C4M671 C4M671_ENTHI [tr] Uncharacterized protein 470 370-375
30. NSLSSD C4M210 C4M210_ENTHI [tr] Lipid phosphate phosphatase putative 255 228-233
31. NSLSSD C4M4B4 C4M4B4_ENTHI [tr] Protein kinase domain containing protein 1989 1386-1391
32. NSLSSD C4M671 C4M671_ENTHI [tr] Uncharacterized protein 470 371-376
33. TVSILT Q1EQ46 Q1EQ46_ENTHI [tr] EhSec24C 876 638-643
34. ILTMNN C4M5P6 C4M5P6_ENTHI [tr] Uncharacterized protein 579 154-159
35. FSLLLC C4LV96 C4LV96_ENTHI [tr] Uncharacterized protein 184 6-11
36. TDFLLL C4LSX8 C4LSX8_ENTHI [tr] Uncharacterized protein 243 97-102
37. TDFLLL C4M8P6 C4M8P6_ENTHI [tr] Helicase putative 1192 570-575
38. LLLTQN C4LXB6 C4LXB6_ENTHI [tr] Uncharacterized protein 519 511-516
39. LLLTQN C4LXH8 C4LXH8_ENTHI [tr] Uncharacterized protein 436 57-62
40. LLTQNN C4LZG3 C4LZG3_ENTHI [tr] Tagatose 6 phosphate kinase putative 285 56-61
41. LLTQNN C4M0Y2 C4M0Y2_ENTHI [tr] Uncharacterized protein 323 308-313
42. LGSIIN C4LST1 C4LST1_ENTHI [tr] Uncharacterized protein 1539 655-660
43. SIINIT A0A175JFD7 A0A175JFD7_ENTHI [tr] Uncharacterized protein 308 122-127
44. SIINIT C4LUI7 C4LUI7_ENTHI [tr] Uncharacterized protein 297 122-127
45. TQDLLS C4LSF9 C4LSF9_ENTHI [tr] Uncharacterized protein 412 337-342
46. LLSFLQ C4LVA7 C4LVA7_ENTHI [tr] Gamma-adaptin, putative 837 382-387
47. LLSFLQ Q1EQ24 Q1EQ24_ENTHI [tr] Gamma subunit isoform 1 837 382-387
48. LESIKN C4M9J6 C4M9J6_ENTHI [tr] Ras guanine nucleotide exchange factor putative 1166 965-970
49. LESIKN S0AW96 S0AW96_ENTHI [tr] Uncharacterized protein 359 37-42
50. ESIKNS C4M5T7 C4M5T7_ENTHI [tr] Uncharacterized protein 430 141-146
51. SIKNST C4LZC5 C4LZC5_ENTHI [tr] Leucine rich repeat protein bspa family 400 324-329
52. IKNSTP A0A060MZZ9 A0A060MZZ9_ENTHI [tr] Alcohol dehydrogenase 3, putative 384 318-323
53. IKNSTP A0A060N1U0 A0A060N1U0_ENTHI [tr] Alcohol dehydrogenase 3, putative 384 318-323
54. IKNSTP A0A060N1V7 A0A060N1V7_ENTHI [tr] Alcohol dehydrogenase 3, putative 384 318-323
55. IKNSTP A0A060N202 A0A060N202_ENTHI [tr] Alcohol dehydrogenase 3, putative 384 318-323
56. IKNSTP A0A060N283 A0A060N283_ENTHI [tr] Alcohol dehydrogenase 3, putative 384 318-323
57. IKNSTP A0A060N592 A0A060N592_ENTHI [tr] Alcohol dehydrogenase 3, putative 384 318-323
58. IKNSTP A0A060N638 A0A060N638_ENTHI [tr] Alcohol dehydrogenase 3, putative 384 318-323
59. IKNSTP C4LWS2 C4LWS2_ENTHI [tr] Alcohol dehydrogenase 3 putative 384 318-323
60. IKNSTP O15603 O15603_ENTHI [tr] NADH dependent alcohol dehydrogenase (Fragment) 122 56-61
61. IKNSTP Q24857 ADH3_ENTHI [sp] Alcohol dehydrogenase 3 395 329-334
62. IKNSTP S0AVC4 S0AVC4_ENTHI [tr] Alcohol dehydrogenase 3, putative 384 318-323
63. IKNSTP S0AVL1 S0AVL1_ENTHI [tr] Alcohol dehydrogenase 3, putative 384 318-323
64. IKNSTP S0AVP1 S0AVP1_ENTHI [tr] Alcohol dehydrogenase 3, putative 384 318-323
65. IKNSTP S0AW28 S0AW28_ENTHI [tr] Alcohol dehydrogenase 3, putative 384 318-323
66. IKNSTP S0AW50 S0AW50_ENTHI [tr] Alcohol dehydrogenase 3, putative 384 318-323
67. IKNSTP S0AW68 S0AW68_ENTHI [tr] Alcohol dehydrogenase 3, putative 382 316-321
68. IKNSTP S0AX44 S0AX44_ENTHI [tr] Alcohol dehydrogenase 3, putative 384 318-323
69. IKNSTP S0AYR1 S0AYR1_ENTHI [tr] Alcohol dehydrogenase 3, putative 384 318-323
70. IKNSTP S0B082 S0B082_ENTHI [tr] Alcohol dehydrogenase 3, putative 384 318-323
71. IKNSTP S0B1W5 S0B1W5_ENTHI [tr] Alcohol dehydrogenase 3, putative 384 318-323
72. TVVMFG C4M8C4 C4M8C4_ENTHI [tr] tRNA-methyltransferase catalytic subunit putative 285 13-18
73. ESIRRI A0A175JJZ5 A0A175JJZ5_ENTHI [tr] Uncharacterized protein 2653 1771-1776
74. ESIRRI C4LY79 C4LY79_ENTHI [tr] Uncharacterized protein 3652 1771-1776
75. IRRIFE A0A175JYY5 A0A175JYY5_ENTHI [tr] rRNA biogenesis protein RRP5 810 759-764
76. IRRIFE B1N3M7 B1N3M7_ENTHI [tr] S1 RNA binding domain-containing protein 799 748-753
77. IRRIFE B1N4H4 B1N4H4_ENTHI [tr] rRNA biogenesis protein RRP5, putative 517 466-471
78. IRRIFE B1N5X7 B1N5X7_ENTHI [tr] Uncharacterized protein 407 356-361
79. IRRIFE C4M2T6 C4M2T6_ENTHI [tr] S1 RNA binding domain-containing protein 1710 1659-1664
80. IRRIFE C4MAW0 C4MAW0_ENTHI [tr] rRNA biogenesis protein RRP5, putative 811 760-765
81. RIFEIT C4LVT0 C4LVT0_ENTHI [tr] Uncharacterized protein 1014 361-366
82. TTQSVF C4MA19 C4MA19_ENTHI [tr] DNA repair helicase putative 648 280-285
83. ELALIP B1N436 B1N436_ENTHI [tr] Uncharacterized protein 469 323-328
84. NLTSGQ C4M446 C4M446_ENTHI [tr] Tyrosine kinase putative 1348 184-189
85. FRGLSG C4LTB9 C4LTB9_ENTHI [tr] Uncharacterized protein 506 476-481
86. STIVSS A0A175JSH0 A0A175JSH0_ENTHI [tr] Uncharacterized protein 922 70-75
87. STIVSS C4M0J5 C4M0J5_ENTHI [tr] Uncharacterized protein 330 141-146
88. STIVSS C4M4L0 C4M4L0_ENTHI [tr] Uncharacterized protein 908 70-75
89. STIVSS S0AWD7 S0AWD7_ENTHI [tr] Uncharacterized protein 330 141-146
90. STIVSS S0AWR6 S0AWR6_ENTHI [tr] Uncharacterized protein 330 141-146
91. TLIEHP A0A060N1N1 A0A060N1N1_ENTHI [tr] Methionine gamma-lyase 389 338-343
92. TLIEHP C4MAS9 C4MAS9_ENTHI [tr] Methionine gamma-lyase 389 338-343
93. TLIEHP Q86D27 Q86D27_ENTHI [tr] Methionine gamma-lyase 392 341-346
94. TLIEHP Q86D28 Q86D28_ENTHI [tr] Methionine gamma-lyase 389 338-343
95. TLIEHP S0AW11 S0AW11_ENTHI [tr] Methionine gamma-lyase 389 338-343
96. IEHPFV C4M4X2 C4M4X2_ENTHI [tr] FHA domain protein, putative 180 75-80
97. PFVFTR C4M149 C4M149_ENTHI [tr] Uncharacterized protein 884 464-469
98. FTREVD A0A175JEP3 A0A175JEP3_ENTHI [tr] Uncharacterized protein 402 114-119
99. FTREVD B1N2I8 B1N2I8_ENTHI [tr] Uncharacterized protein 398 114-119
100. TREVDD A0A175JEP3 A0A175JEP3_ENTHI [tr] Uncharacterized protein 402 115-120
101. TREVDD B1N2I8 B1N2I8_ENTHI [tr] Uncharacterized protein 398 115-120
102. TNDSST A0A175JFQ8 A0A175JFQ8_ENTHI [tr] Uncharacterized protein 1597 217-222
103. TNDSST C4LUT6 C4LUT6_ENTHI [tr] Uncharacterized protein 1999 619-624
104. TNDSST C4M3Y6 C4M3Y6_ENTHI [tr] Uncharacterized protein 1598 217-222
105. DSSTLD C4M5P4 C4M5P4_ENTHI [tr] Uncharacterized protein 1225 192-197
106. TLDSLF C4LT26 C4LT26_ENTHI [tr] 4-alpha-glucanotransferase putative 898 586-591
107. LDSLFS A0A175JR33 A0A175JR33_ENTHI [tr] Uncharacterized protein 2474 864-869
108. LDSLFS C4M2V8 C4M2V8_ENTHI [tr] Uncharacterized protein 2111 1646-1651
109. LDSLFS C4M3H1 C4M3H1_ENTHI [tr] Uncharacterized protein 2457 847-852
110. FSSLHS C4M5W5 C4M5W5_ENTHI [tr] Uncharacterized protein 461 429-434
111. FSSLHS C4M8Y5 C4M8Y5_ENTHI [tr] Hydrolase alpha beta fold family domain containing 277 263-268
112. SLHSSN C4M5Q1 C4M5Q1_ENTHI [tr] Ras guanine nucleotide exchange factor putative 1214 1147-1152
113. PIKFKK C4LYK1 C4LYK1_ENTHI [tr] Rab GTPase activating protein putative 504 147-152
114. IDLLEK C4LY01 C4LY01_ENTHI [tr] Uncharacterized protein 828 746-751
115. YIVGDG C4M6I1 C4M6I1_ENTHI [tr] Uncharacterized protein 996 810-815
116. LVGDLL A0A175JV39 A0A175JV39_ENTHI [tr] GTP-binding protein Ras family 329 297-302
117. LVGDLL C4M7T6 C4M7T6_ENTHI [tr] GTP-binding protein, Ras family 314 297-302
118. TSFSIN A0A175JU64 A0A175JU64_ENTHI [tr] Dolichyl-diphosphooligosaccharide--protein glycosyltransferase subunit 1 435 309-314
119. TSFSIN C4M4R3 C4M4R3_ENTHI [tr] Uncharacterized protein 259 188-193
120. TSFSIN C4M718 C4M718_ENTHI [tr] Dolichyl-diphosphooligosaccharide--protein glycosyltransferase subunit 1 406 280-285
121. ARSQVI C4LTC5 C4LTC5_ENTHI [tr] Cell division cycle protein 48, putative 794 325-330
122. SQVIDF C4LVC5 C4LVC5_ENTHI [tr] Uncharacterized protein 1344 296-301
123. QVIDFT C4LVC5 C4LVC5_ENTHI [tr] Uncharacterized protein 1344 297-302
124. TSPFFS C4M9K2 C4M9K2_ENTHI [tr] Uncharacterized protein 372 200-205
125. STSLGI A0A175K1G8 A0A175K1G8_ENTHI [tr] Uncharacterized protein 213 142-147
126. STSLGI B1N5F9 B1N5F9_ENTHI [tr] Uncharacterized protein (Fragment) 224 142-147
127. STSLGI C4LZL0 C4LZL0_ENTHI [tr] Uncharacterized protein 790 142-147
128. TSLGIL A0A175JT85 A0A175JT85_ENTHI [tr] Leucine rich repeat protein phosphatase 2c domain containing protein 850 427-432
129. TSLGIL C4M699 C4M699_ENTHI [tr] Leucine rich repeat / protein phosphatase 2C domain containing protein 850 427-432
130. GIFVAL C4LTQ4 C4LTQ4_ENTHI [tr] UDP-N-acetylglucosamine transporter putative 318 253-258
131. GIFVAL C4M5Q1 C4M5Q1_ENTHI [tr] Ras guanine nucleotide exchange factor putative 1214 874-879
132. VALHIT Q9UAB5 Q9UAB5_ENTHI [tr] Grainin 1 215 14-19
133. ALHITA Q9UAB5 Q9UAB5_ENTHI [tr] Grainin 1 215 15-20
134. LYEWKS C4LVK6 C4LVK6_ENTHI [tr] WD domain containing protein 392 291-296
135. KSPFGL C4M2T0 C4M2T0_ENTHI [tr] Uncharacterized protein 593 63-68
136. YALLFG C4M1T7 C4M1T7_ENTHI [tr] Uncharacterized protein 210 24-29
137. ALLFGR C4M1T7 C4M1T7_ENTHI [tr] Uncharacterized protein 210 25-30
138. LLFGRT C4M1K8 C4M1K8_ENTHI [tr] Uncharacterized protein 494 355-360
139. LFGRTV A0A175JLD2 A0A175JLD2_ENTHI [tr] Uncharacterized protein 1011 666-671
140. LFGRTV B1N367 B1N367_ENTHI [tr] Uncharacterized protein 932 587-592
141. EKIYEE C4M704 C4M704_ENTHI [tr] Uncharacterized protein 1339 76-81
142. KIYEEL A0A175JIP0 A0A175JIP0_ENTHI [tr] Uncharacterized protein 3302 2534-2539
143. KIYEEL C4LX72 C4LX72_ENTHI [tr] Uncharacterized protein 3289 2521-2526
144. PATPDG A0A175K0T1 A0A175K0T1_ENTHI [tr] Maltose o-acetyltransferase putative 151 20-25
145. PATPDG B1N458 B1N458_ENTHI [tr] Maltose O-acetyltransferase, putative 191 20-25
146. PATPDG B1N487 B1N487_ENTHI [tr] Acetyltransferase putative 204 20-25
147. PATPDG B1N5B7 B1N5B7_ENTHI [tr] Acetyltransferase putative 204 20-25
148. PATPDG B1N5J1 B1N5J1_ENTHI [tr] Maltose O-acetyltransferase, putative 191 20-25
149. PATPDG C4M9L8 C4M9L8_ENTHI [tr] Acetyltransferase putative 204 20-25
150. PATPDG C4MB27 C4MB27_ENTHI [tr] Bacterial transferase hexapeptide family protein 176 20-25
151. ATPDGV A0A175K0T1 A0A175K0T1_ENTHI [tr] Maltose o-acetyltransferase putative 151 21-26
152. ATPDGV B1N458 B1N458_ENTHI [tr] Maltose O-acetyltransferase, putative 191 21-26
153. ATPDGV B1N487 B1N487_ENTHI [tr] Acetyltransferase putative 204 21-26
154. ATPDGV B1N5B7 B1N5B7_ENTHI [tr] Acetyltransferase putative 204 21-26
155. ATPDGV B1N5J1 B1N5J1_ENTHI [tr] Maltose O-acetyltransferase, putative 191 21-26
156. ATPDGV C4M9L8 C4M9L8_ENTHI [tr] Acetyltransferase putative 204 21-26
157. ATPDGV C4MB27 C4MB27_ENTHI [tr] Bacterial transferase hexapeptide family protein 176 21-26
158. IDADCK A0A175JMR6 A0A175JMR6_ENTHI [tr] Uncharacterized protein 1530 1493-1498
159. IDADCK C4M0K3 C4M0K3_ENTHI [tr] Uncharacterized protein 1513 1476-1481
160. LPPNSP C4LTU8 C4LTU8_ENTHI [tr] U1snRNP-specific protein, putative 232 151-156
161. TANISE C4M3A7 C4M3A7_ENTHI [tr] Uncharacterized protein 919 279-284
162. NISELI A0A175JHL2 A0A175JHL2_ENTHI [tr] Uncharacterized protein 1229 609-614
163. NISELI A0A175JKF8 A0A175JKF8_ENTHI [tr] Uncharacterized protein 304 213-218
164. NISELI C4LW41 C4LW41_ENTHI [tr] Uncharacterized protein 1229 609-614
165. NISELI C4LYL0 C4LYL0_ENTHI [tr] Uncharacterized protein 289 198-203
166. NISELI Q56AY2 Q56AY2_ENTHI [tr] CAF1 family ribonuclease, putative 311 69-74
167. ISELIS A0A175JKF8 A0A175JKF8_ENTHI [tr] Uncharacterized protein 304 214-219
168. ISELIS C4LYL0 C4LYL0_ENTHI [tr] Uncharacterized protein 289 199-204
169. ISELIS C4MAC6 C4MAC6_ENTHI [tr] Uncharacterized protein 2089 1455-1460
170. ELISQY A0A175K147 A0A175K147_ENTHI [tr] Regulator of nonsense transcripts putative 1147 831-836
171. ELISQY C4LT63 C4LT63_ENTHI [tr] Regulator of nonsense transcripts putative 966 818-823
172. ELISQY C4LUD0 C4LUD0_ENTHI [tr] Regulator of nonsense transcripts putative 965 817-822
173. ELISQY C4LUR8 C4LUR8_ENTHI [tr] Regulator of nonsense transcripts putative 915 759-764
174. ELISQY C4LZD1 C4LZD1_ENTHI [tr] Protein with RhoGEF and ArfGAP domains 1098 778-783
175. ELISQY C4M4V4 C4M4V4_ENTHI [tr] Regulator of nonsense transcripts putative 931 736-741
176. ELISQY C4M5S7 C4M5S7_ENTHI [tr] Regulator of nonsense transcripts putative 979 817-822
177. LISQYK C4LZD1 C4LZD1_ENTHI [tr] Protein with RhoGEF and ArfGAP domains 1098 779-784
178. LFVLLC A0A0D4ZXV0 A0A0D4ZXV0_ENTHI [tr] Amoebapore C (Fragment) 84 3-8
179. LFVLLC A1A492 A1A492_ENTHI [tr] Amoebapore C 101 3-8
180. LFVLLC C4LZ09 C4LZ09_ENTHI [tr] Amoebapore C 101 3-8
181. LFVLLC Q24825 PFPC_ENTHI [sp] Pore-forming peptide ameobapore C precursor 101 3-8
182. GFGLSI C4LUT3 C4LUT3_ENTHI [tr] Brefeldin a-inhibited guanine nucleotide-exchange protein putative 1476 557-562
183. SILTTI C4M9R7 C4M9R7_ENTHI [tr] Ras guanine nucleotide exchange factor putative 1190 177-182
184. ILTTIG C4LTY8 C4LTY8_ENTHI [tr] CDP-alcohol phosphatidyltransferase family protein 371 55-60
185. ILTTIG C4M447 C4M447_ENTHI [tr] Uncharacterized protein 777 688-693
186. IVYRLL A0A175JH31 A0A175JH31_ENTHI [tr] Rhogap domain containing protein 470 303-308
187. IVYRLL B1N2T0 B1N2T0_ENTHI [tr] RhoGAP domain containing protein 479 303-308
188. IKNKSK C4LT43 C4LT43_ENTHI [tr] Syntaxin putative 278 49-54
189. IKNKSK C4M4U2 C4M4U2_ENTHI [tr] Uncharacterized protein 1102 714-719
190. IKNKSK Q1EQ05 Q1EQ05_ENTHI [tr] EhSyntaxin 16 (Fragment) 278 49-54
191. TSFIEE C4LYS5 C4LYS5_ENTHI [tr] Uncharacterized protein 434 275-280
192. TSFIEE C4M6B0 C4M6B0_ENTHI [tr] Uncharacterized protein 535 441-446
193. SFIEEK C4M6B0 C4M6B0_ENTHI [tr] Uncharacterized protein 535 442-447
194. IEEKQQ B1N2M9 B1N2M9_ENTHI [tr] Uncharacterized protein 2880 1206-1211
195. IEEKQQ C4LSP1 C4LSP1_ENTHI [tr] Rho guanine nucleotide exchange factor putative 772 104-109
196. VEKRSN Q1EQ47 Q1EQ47_ENTHI [tr] EhSec24B 647 268-273
197. IPLPPR C4M435 C4M435_ENTHI [tr] DEAD/DEAH box helicase, putative 757 427-432
198. QELSEL A0A175JJB1 A0A175JJB1_ENTHI [tr] Uncharacterized protein 1598 1162-1167
199. QELSEL A0A175JUV0 A0A175JUV0_ENTHI [tr] DNA primase large subunit putative 526 97-102
200. QELSEL C4LY98 C4LY98_ENTHI [tr] Uncharacterized protein 1574 1138-1143
201. QELSEL C4M6L8 C4M6L8_ENTHI [tr] DNA primase large subunit, putative 523 94-99
202. ELEKQI A0A175JHK5 A0A175JHK5_ENTHI [tr] Uncharacterized protein 443 188-193
203. ELEKQI C4LWS8 C4LWS8_ENTHI [tr] Uncharacterized protein 364 109-114
204. ELEKQI C4M298 C4M298_ENTHI [tr] Proteasome regulatory subunit putative 448 381-386
205. ELEKQI C4M4A4 C4M4A4_ENTHI [tr] Uncharacterized protein 206 120-125
206. ELEKQI C4M8Q9 C4M8Q9_ENTHI [tr] Uncharacterized protein 397 174-179
207. LEKQIQ A0A060N6S9 A0A060N6S9_ENTHI [tr] 26S protease regulatory subunit, putative 410 160-165
208. LEKQIQ A0A175K147 A0A175K147_ENTHI [tr] Regulator of nonsense transcripts putative 1147 513-518
209. LEKQIQ C4LUR8 C4LUR8_ENTHI [tr] Regulator of nonsense transcripts putative 915 448-453
210. LEKQIQ C4LWL8 C4LWL8_ENTHI [tr] Cullin family protein 728 152-157
211. LEKQIQ C4M285 C4M285_ENTHI [tr] 26S protease regulatory subunit putative 410 160-165

**NMDA 3B**

1. GGSVRL B1N4J7 B1N4J7_ENTHI [tr] Uncharacterized protein 176 119-124
2. GGSVRL C4MAZ2 C4MAZ2_ENTHI [tr] Uncharacterized protein 266 209-214
3. RARAAL C4LT54 C4LT54_ENTHI [tr] Uncharacterized protein 1122 295-300
4. SLELVV C4M886 C4M886_ENTHI [tr] Leucine rich repeat protein bspa family 537 404-409
5. VAALLA A0A175JN60 A0A175JN60_ENTHI [tr] Uncharacterized protein 154 16-21
6. VAALLA A0A175K0X9 A0A175K0X9_ENTHI [tr] Uncharacterized protein 243 16-21
7. VAALLA B1N4E5 B1N4E5_ENTHI [tr] Uncharacterized protein 236 16-21
8. VAALLA B1N528 B1N528_ENTHI [tr] Uncharacterized protein 152 16-21
9. VAALLA B1N609 B1N609_ENTHI [tr] Uncharacterized protein 243 16-21
10. VAALLA B1N625 B1N625_ENTHI [tr] Uncharacterized protein (Fragment) 251 73-78
11. VAALLA C4M1R1 C4M1R1_ENTHI [tr] Uncharacterized protein 233 16-21
12. VAALLA C4M826 C4M826_ENTHI [tr] Uncharacterized protein 154 16-21
13. VAALLA C4MAM7 C4MAM7_ENTHI [tr] Uncharacterized protein 155 16-21
14. VAALLA C4MB85 C4MB85_ENTHI [tr] Uncharacterized protein 155 16-21
15. LLQLHF C4LTK1 C4LTK1_ENTHI [tr] Uncharacterized protein 319 227-232
16. LLQLHF C4M3G8 C4M3G8_ENTHI [tr] Uncharacterized protein 393 313-318
17. LQLHFL A0A175JI46 A0A175JI46_ENTHI [tr] Uncharacterized protein 2036 1081-1086
18. LQLHFL C4LX90 C4LX90_ENTHI [tr] Uncharacterized protein 2020 1065-1070
19. PVLSLL C4M2H9 C4M2H9_ENTHI [tr] Uncharacterized protein 132 27-32
20. PVLSLL C4M2I0 C4M2I0_ENTHI [tr] Uncharacterized protein 132 27-32
21. PLETLL C4M3G8 C4M3G8_ENTHI [tr] Uncharacterized protein 393 25-30
22. PLETLL C4M8E1 C4M8E1_ENTHI [tr] Centromere microtubule binding protein cbf5 putative 484 256-261
23. LETLLD C4M6D3 C4M6D3_ENTHI [tr] Uncharacterized protein 604 151-156
24. LLDVLV C4M6K0 C4M6K0_ENTHI [tr] Splicing factor Prp8, putative 2270 1227-1232
25. VLVAVL C4LUA6 C4LUA6_ENTHI [tr] Protein tyrosine phosphatase putative 218 83-88
26. PPQLVL C4LTH3 C4LTH3_ENTHI [tr] Uncharacterized protein 753 117-122
27. PPQLVL C4LUJ4 C4LUJ4_ENTHI [tr] Uncharacterized protein 762 118-123
28. QLVLDL C4M515 C4M515_ENTHI [tr] HEAT repeat domain containing protein 1071 662-667
29. GEAPVP C4M610 C4M610_ENTHI [tr] Viral A-type inclusion protein repeat, putative 1813 37-42
30. APVPAA C4MAN6 C4MAN6_ENTHI [tr] Uncharacterized protein 510 361-366
31. PVPAAV C4M7W1 C4M7W1_ENTHI [tr] Uncharacterized protein 1656 1388-1393
32. AAVLLG C4M722 C4M722_ENTHI [tr] Meiotic check point regulator putative 803 257-262
33. PLPPKA C4LTT3 C4LTT3_ENTHI [tr] Phospholipase patatin family protein 650 344-349
34. PLPPKA C4LWE2 C4LWE2_ENTHI [tr] Uncharacterized protein 407 247-252
35. GLPPGL C4LW03 C4LW03_ENTHI [tr] Heat shock protein 70 putative 672 604-609
36. GLPPGL C4LZD0 C4LZD0_ENTHI [tr] Heat shock protein 70 putative 669 601-606
37. GLPPGL C4M340 C4M340_ENTHI [tr] Heat shock protein 70 putative 572 504-509
38. LGEVAR C4LU65 C4LU65_ENTHI [tr] Vacuolar ATP synthase subunit H, putative 444 388-393
39. LEAAIH C4LVB5 C4LVB5_ENTHI [tr] Importin beta 3 family protein 964 206-211
40. GRFLAR A0A175JNY4 A0A175JNY4_ENTHI [tr] DNA polymerase putative 1278 686-691
41. GRFLAR B1N3D4 B1N3D4_ENTHI [tr] DNA polymerase, putative 1278 686-691
42. RFLARF A0A175JNY4 A0A175JNY4_ENTHI [tr] DNA polymerase putative 1278 687-692
43. RFLARF B1N3D4 B1N3D4_ENTHI [tr] DNA polymerase, putative 1278 687-692
44. SFQGRT C4LV42 C4LV42_ENTHI [tr] Myb-like DNA-binding domain containing protein 167 101-106
45. DPRGAP C4M901 C4M901_ENTHI [tr] Zinc finger domain containing protein 217 122-127
46. LLEHPF C4LVK5 C4LVK5_ENTHI [tr] Protein kinase putative 602 579-584
47. LLEHPF Q24848 Q24848_ENTHI [tr] P21 activated kinase 458 435-440
48. LEHPFV B1N4S9 B1N4S9_ENTHI [tr] Uncharacterized protein 214 181-186
49. LEHPFV C4LX33 C4LX33_ENTHI [tr] Uncharacterized protein 620 587-592
50. LDPGTN C4LT47 C4LT47_ENTHI [tr] Gal galnac lectin light subunit 270 179-184
51. LDPGTN C4M5G9 C4M5G9_ENTHI [tr] Galactose-inhibitable lectin putative 271 178-183
52. LDPGTN S0AWZ5 S0AWZ5_ENTHI [tr] Gal/GalNAc lectin light subunit 270 179-184
53. LDPGTN S0B094 S0B094_ENTHI [tr] Galactose-inhibitable lectin, putative 271 178-183
54. SATLDA C4M6S9 C4M6S9_ENTHI [tr] Helicase putative 953 445-450
55. APRALR C4LTT8 C4LTT8_ENTHI [tr] Uncharacterized protein 360 38-43
56. ERLAED B1N3D6 B1N3D6_ENTHI [tr] Elongation factor 2 841 594-599
57. FELYLV C4LYS2 C4LYS2_ENTHI [tr] Uncharacterized protein 260 223-228
58. ELYLVG C4LSL1 C4LSL1_ENTHI [tr] RhoGAP domain containing protein 1067 297-302
59. LYLVGD C4M519 C4M519_ENTHI [tr] Tyrosine kinase putative 1590 562-567
60. LVGDLL A0A175JV39 A0A175JV39_ENTHI [tr] GTP-binding protein Ras family 329 297-302
61. LVGDLL C4M7T6 C4M7T6_ENTHI [tr] GTP-binding protein, Ras family 314 297-302
62. VGDLLA A0A175JV39 A0A175JV39_ENTHI [tr] GTP-binding protein Ras family 329 298-303
63. VGDLLA A0A175JX25 A0A175JX25_ENTHI [tr] Nucleoside transporter putative 407 37-42
64. VGDLLA C4M7T6 C4M7T6_ENTHI [tr] GTP-binding protein, Ras family 314 298-303
65. VGDLLA C4M8V2 C4M8V2_ENTHI [tr] Nucleoside transporter, putative 379 9-14
66. VGDLLA Q24832 Q24832_ENTHI [tr] Cytochrome b-like protein 396 37-42
67. TSFSIN A0A175JU64 A0A175JU64_ENTHI [tr] Dolichyl-diphosphooligosaccharide--protein glycosyltransferase subunit 1 435 309-314
68. TSFSIN C4M4R3 C4M4R3_ENTHI [tr] Uncharacterized protein 259 188-193
69. TSFSIN C4M718 C4M718_ENTHI [tr] Dolichyl-diphosphooligosaccharide--protein glycosyltransferase subunit 1 406 280-285
70. SFSINS C4M2H1 C4M2H1_ENTHI [tr] Uncharacterized protein 509 79-84
71. VDFTSP C4LUI6 C4LUI6_ENTHI [tr] Uncharacterized protein 100 73-78
72. TSPFFS C4M9K2 C4M9K2_ENTHI [tr] Uncharacterized protein 372 200-205
73. STSLGI A0A175K1G8 A0A175K1G8_ENTHI [tr] Uncharacterized protein 213 142-147
74. STSLGI B1N5F9 B1N5F9_ENTHI [tr] Uncharacterized protein (Fragment) 224 142-147
75. STSLGI C4LZL0 C4LZL0_ENTHI [tr] Uncharacterized protein 790 142-147
76. TSLGIM A0A175JSH0 A0A175JSH0_ENTHI [tr] Uncharacterized protein 922 192-197
77. TSLGIM C4M4L0 C4M4L0_ENTHI [tr] Uncharacterized protein 908 192-197
78. SLGIMV Q5FZP2 Q5FZP2_ENTHI [tr] p21-activated kinase 467 375-380
79. VRARDT C4M791 C4M791_ENTHI [tr] Protein kinase with WD repeats 1301 38-43
80. ALHLTA C4LTF3 C4LTF3_ENTHI [tr] Arf GTPase activating protein putative 598 522-527
81. LTALFL A0A175JW48 A0A175JW48_ENTHI [tr] Uncharacterized protein 668 215-220
82. LTALFL C4LWQ6 C4LWQ6_ENTHI [tr] Uncharacterized protein 404 46-51
83. LTALFL C4M8R5 C4M8R5_ENTHI [tr] Uncharacterized protein 806 215-220
84. ALFLTV A0A175JJB2 A0A175JJB2_ENTHI [tr] Uncharacterized protein 465 10-15
85. ALFLTV A0A175JJE3 A0A175JJE3_ENTHI [tr] Uncharacterized protein 297 10-15
86. ALFLTV B1N311 B1N311_ENTHI [tr] Uncharacterized protein 297 10-15
87. ALFLTV C4LYC3 C4LYC3_ENTHI [tr] Uncharacterized protein 474 10-15
88. FLTVYE C4LSH9 C4LSH9_ENTHI [tr] WD domain containing protein 881 291-296
89. LTVYEW C4LSH9 C4LSH9_ENTHI [tr] WD domain containing protein 881 292-297
90. TPRGRN C4M3W1 C4M3W1_ENTHI [tr] Uncharacterized protein 196 129-134
91. TVFSYS C4LZL8 C4LZL8_ENTHI [tr] DENN domain protein 991 119-124
92. VFSYSS C4LZL8 C4LZL8_ENTHI [tr] DENN domain protein 991 120-125
93. YSSALN C4M5C8 C4M5C8_ENTHI [tr] Uncharacterized protein 2378 1064-1069
94. KTPKCP C4LUP3 C4LUP3_ENTHI [tr] Uncharacterized protein 1028 776-781
95. TPKCPT C4LUP3 C4LUP3_ENTHI [tr] Uncharacterized protein 1028 777-782
96. FCLLVL A0A175JTX4 A0A175JTX4_ENTHI [tr] Six tm domain protein 217 62-67
97. CLLVLS A0A175JJD2 A0A175JJD2_ENTHI [tr] Uncharacterized protein 256 211-216
98. CLLVLS C4LY03 C4LY03_ENTHI [tr] Uncharacterized protein 140 95-100
99. LLVLSS A0A175JJD2 A0A175JJD2_ENTHI [tr] Uncharacterized protein 256 212-217
100. LLVLSS C4LY03 C4LY03_ENTHI [tr] Uncharacterized protein 140 96-101
101. LVLSSY B1N4H0 B1N4H0_ENTHI [tr] Uncharacterized protein 578 235-240
102. VLSSYT C4LVP1 C4LVP1_ENTHI [tr] Uncharacterized protein 399 303-308
103. VGDKTF C4M1I7 C4M1I7_ENTHI [tr] LIM zinc finger domain containing protein 387 289-294
104. KTFEEL C4LSK5 C4LSK5_ENTHI [tr] HEAT repeat domain containing protein 1074 567-572
105. KLNAFI A0A175JGA3 A0A175JGA3_ENTHI [tr] Spindle pole body component 666 577-582
106. KLNAFI C4LVM1 C4LVM1_ENTHI [tr] Spindle pole body component 648 559-564
107. LNAFIM C4LU21 C4LU21_ENTHI [tr] Uncharacterized protein 357 324-329
108. MDKSLL C4LXB4 C4LXB4_ENTHI [tr] Microfibril-associated protein putative 242 206-211
109. DKSLLD C4M9A6 C4M9A6_ENTHI [tr] Uncharacterized protein 488 201-206
110. IDADCK A0A175JMR6 A0A175JMR6_ENTHI [tr] Uncharacterized protein 1530 1493-1498
111. IDADCK C4M0K3 C4M0K3_ENTHI [tr] Uncharacterized protein 1513 1476-1481
112. NSPLTS C4LYM6 C4LYM6_ENTHI [tr] WD domain containing protein 341 66-71
113. LTSNLS C4M876 C4M876_ENTHI [tr] Uncharacterized protein 1416 531-536
114. LSEFIS C4LZX7 C4LZX7_ENTHI [tr] Ras GTPase activating protein putative 604 234-239
115. SGFIDL C4M0A4 C4M0A4_ENTHI [tr] Eukaryotic translation initiation factor 2 alpha subunit putative 299 86-91
116. GFIDLL C4LSF5 C4LSF5_ENTHI [tr] Haloacid dehalogenase-like hydrolase domain-contain 227 91-96
117. LFVLLC A0A0D4ZXV0 A0A0D4ZXV0_ENTHI [tr] Amoebapore C (Fragment) 84 3-8
118. LFVLLC A1A492 A1A492_ENTHI [tr] Amoebapore C 101 3-8
119. LFVLLC C4LZ09 C4LZ09_ENTHI [tr] Amoebapore C 101 3-8
120. LFVLLC Q24825 PFPC_ENTHI [sp] Pore-forming peptide ameobapore C precursor 101 3-8
121. FVLLCL A0A175JI84 A0A175JI84_ENTHI [tr] Uncharacterized protein 2034 323-328
122. FVLLCL C4LWT5 C4LWT5_ENTHI [tr] Uncharacterized protein 2005 323-328
123. LGLGSA C4MBG1 C4MBG1_ENTHI [tr] Metal cation transporter zinc zn2-iron fe2 permease 298 157-162
124. GSALLS C4M6M7 C4M6M7_ENTHI [tr] Citrate transporter putative 784 768-773
125. IHRALN C4LXN9 C4LXN9_ENTHI [tr] Phosphatidylinositol 3-kinase putative 1413 1011-1016
126. NTEPPE C4LZV9 C4LZV9_ENTHI [tr] Uncharacterized protein 395 336-341
127. EQQQQQ A0A175K293 A0A175K293_ENTHI [tr] Uncharacterized protein 367 182-187
128. EQQQQQ B1N5Z1 B1N5Z1_ENTHI [tr] Uncharacterized protein (Fragment) 361 182-187
129. EQQQQQ C4LU21 C4LU21_ENTHI [tr] Uncharacterized protein 357 4-9
130. QQQQQQ A0A175JPV6 A0A175JPV6_ENTHI [tr] Sac3 ganp family protein 670 161-166, 162-167
131. QQQQQQ C4LU21 C4LU21_ENTHI [tr] Uncharacterized protein 357 5-10, 6-11
132. QQQQQQ C4LVK2 C4LVK2_ENTHI [tr] Uncharacterized protein 533 33-38
133. QQQQQQ C4M2B4 C4M2B4_ENTHI [tr] SAC3/GANP family protein 670 161-166, 162-167
134. QQQQQQ C4M431 C4M431_ENTHI [tr] Valyl-tRNA synthetase putative 1050 48-53
135. QQQQQQ C4M4D3 C4M4D3_ENTHI [tr] Uncharacterized protein 816 551-556, 552-557, 553-558
136. QQQQQQ C4M567 C4M567_ENTHI [tr] Uncharacterized protein 656 475-480, 476-481
137. RVRFLL C4LVL6 C4LVL6_ENTHI [tr] Uncharacterized protein 505 117-122
138. AEAEAA C4LYR5 C4LYR5_ENTHI [tr] Uncharacterized protein 410 316-321
139. ELQELE A0A175JMS5 A0A175JMS5_ENTHI [tr] Uncharacterized protein 83 71-76
140. IEVARE C4LT54 C4LT54_ENTHI [tr] Uncharacterized protein 1122 680-685
141. PRRLLQ C4MA58 C4MA58_ENTHI [tr] Phospholipid-transporting ATPase 1055 179-184
142. AAPAEA C4LY50 C4LY50_ENTHI [tr] 40S ribosomal protein S25, putative 137 118-123
