# Supplementary material for: From Toxoplasmosis to Schizophrenia via NMDA Dysfunction: Peptide Overlap between Toxoplasma gondii and N-Methyl-d-Aspartate Receptors As a Potential Mechanistic Link
Source: Front Psychiatry. 2017 Mar 15;8:37. doi: 10.3389/fpsyt.2017.00037 (PMC5350139; doi:10.3389/fpsyt.2017.00037)
Supplement: Supplementary file 3 [file Table_3.DOC]

Supplementary Table S3. Epitopic hexapeptides shared between the 7 human NMDAR subunits

and the *E. Histolytica*  proteome

| NMDA 1* | NMDA 2A | NMDA 2B | NMDA 2C | NMDA 2D | NMDA 3A | NMDA 3B |
| --- | --- | --- | --- | --- | --- | --- |
| AIQMAL | AAAEKG | LKKISK | AGVSSS | AGGGGS | ELISQY | AAPAEA |
| DLLIKL | ALNIAV | LKKLQS | LAQAQS | ALVLTP | ILTTIG | AEAEAA |
| EAIQAV | EELETL | LSSIES | LLLTSL | APPPPP | TREVDD | APVPAA |
| EDLISS | GIGILT | NILRLL | LSLRQK | ASLELL |  | DPRGAP |
| EEEEED | INNSTN | PVVSAL | PALLLT | AVAAAV |  | EQQQQQ |
| ELLEKE | PLHNEE | QEAIAQ |  | AVVARG |  | GEAPVP |
| HELLEK | SRSISL | SNILRL |  | GASLGG |  | GSALLS |
| IVNIGA | SSVILL | TTVTGQ |  | GGPGGG |  | LEHPFV |
| KIVNIG | TKSSSP | YSTTVT |  | GKKIDG |  | QQQQQQ |
| LLEERE | VYISEH |  |  | GLGLGL |  | RARAAL |
| LLNSGI |  |  |  | LGLGLG |  |  |
| NLAAFL |  |  |  | LLLLAL |  |  |
| SEEEEE |  |  |  | PAAPPP |  |  |
|  |  |  |  | PPPPPQ |  |  |
|  |  |  |  | RSLVLQ |  |  |

*NMDA 1 hexapeptides were common to the 7 isoforms
